# ChatGPT Versus DeepSeek for Breast Cancer Information Retrieval: Quantitative Comparative Study

**DOI:** 10.2196/72839

**Published:** 2026-02-27

**Authors:** Rima Hajjo, Dima A Sabbah, Sanaa K Bardaweel

**Affiliations:** 1 Department of Pharmacy, Faculty of Pharmacy, Al-Zaytoonah University of Jordan, Airport Street, P.O. Box 130, Amman, 11733, Jordan, 962 64291511; 2 Department of Pharmaceutical Sciences, School of Pharmacy, University of Jordan, Amman, Jordan

**Keywords:** artificial intelligence, breast cancer, ChatGPT, DeepSeek, large language models, AI, LLM

## Abstract

**Background:**

Artificial intelligence (AI) is increasingly used to generate medical content, yet its performance in delivering clinically relevant and reliable information remains underexplored, especially in complex areas such as breast cancer.

**Objective:**

This study aimed to compare ChatGPT-4.0 and DeepSeek-V3 in generating breast cancer information, focusing on readability, content quality, and citation reliability.

**Methods:**

On the basis of publicly available patient education materials, 10 frequently asked questions were selected. Each model generated 60 responses. Three expert reviewers rated each response using a 7-point Likert scale across 5 dimensions (ie, accuracy, completeness, clarity, depth and insight, and alignment with expert answers). Readability was assessed using Flesch-Kincaid Grade Level scores. Information reliability was evaluated through interrater agreement metrics, including Cohen κ and Fleiss κ. Paired *t* tests were used for statistical comparisons.

**Results:**

AI models produced significantly more readable content than expert references (mean Flesch-Kincaid Grade Level difference −2.60; *P*<.001). ChatGPT-4.0 responses were more stylistically consistent with a median Flesch-Kincaid Grade Level score of 10.66 (IQR 0.98), whereas DeepSeek-V3 showed greater variability with a median Flesch-Kincaid Grade Level score of 10.17 (IQR 1.41). Content quality scores were DeepSeek-V3 achieving a higher mean score than ChatGPT-4.0 (6.22 [SD 0.43] vs 6.01 [SD 0.49]). In the multiresponse analysis, DeepSeek-V3 demonstrated a statistically significant advantage in accuracy (*P*=.041), while differences across other criteria were not statistically significant (*P*>.05). Human raters showed almost perfect agreement when judging source reliability (Fleiss κ=0.842 for ChatGPT’s citations and 0.935 for DeepSeek’s citations). Agreement between each model’s citation reliability scores and the expert majority was substantial for ChatGPT (Cohen κ=0.665) and higher for DeepSeek (Cohen κ=0.782).

**Conclusions:**

Both models generated readable and clinically relevant content with comparable overall performance. ChatGPT provided more consistent readability, while DeepSeek offered more diverse references with stronger alignment to expert ratings. Continued evaluation and quality assurance are essential for the responsible clinical use of AI-generated content.

## Introduction

Breast cancer is the most commonly diagnosed cancer among women and remains a major global health burden. Despite advancements in detection and treatment, late-stage presentation continues to hinder outcomes [1-4]. The World Health Organization highlights the need for improved screening and care access [5], while socioeconomic factors such as education and income shape care-seeking behavior. Many women turn to the internet for information on early signs and symptoms [6,7], although online health content is often inaccurate or misleading [8,9].

In response, large language models (LLMs), such as ChatGPT and DeepSeek, have emerged as potential tools for generating medical information and answering patient queries [10-17]. Trained on extensive biomedical literature and general data sources, these generative artificial intelligence (AI) models can produce natural language responses to diverse health-related questions [18,19], potentially supporting patient education and access to general medical knowledge [20]. Promising results have been demonstrated across multiple areas, including medical imaging, disease detection, drug discovery, and personalized oncology, with some AI models surpassing traditional diagnostic accuracy [21-24]. In oncology, AI aids in drug delivery, genomics integration, and real-time monitoring to improve outcomes [2,5-8,25,26]. Nonetheless, concerns persist regarding the accuracy, readability, and reliability of AI-generated content, especially in complex domains such as breast cancer, necessitating further validation studies [27-30].

A recent work by Ye et al [31] systematically evaluated ChatGPT-3.5’s performance in responding to frequently asked questions (FAQs) on cervical and breast cancer. Their study used 10 validated FAQs developed through expert consensus and compared ChatGPT’s responses to those of experienced gynecologists and mammography specialists. Using a 7-point Likert scale, they assessed multiple dimensions of response quality, including accuracy, readability, consistency, reliability, efficiency, and relevance. While ChatGPT 3.5 demonstrated high accuracy, often comparable to or exceeding physician responses, it also exhibited limitations in readability, consistency, and the reliability of cited sources. Although foundational, this study did not evaluate more advanced generative AI models, such as ChatGPT-4.0 or DeepSeek-V3, whose capabilities remain largely unexplored [31-33]. Thus, further research is needed to assess how newer models compare in generating high-quality, clinically relevant patient education content.

Therefore, the aim of this study was to systematically evaluate and compare ChatGPT-4.0 and DeepSeek-V3 in their ability to generate high-quality, patient-facing breast cancer information. Using an established, expert-validated set of breast cancer FAQs, we assessed each model across multiple dimensions of response quality, including readability, accuracy, completeness, clarity, depth, insight, alignment with expert reference answers, and citation reliability, with the goal of determining whether these systems can provide trustworthy, clinically relevant educational content for patients.

## Methods

### Study Design and Workflow

**Figure 1.**
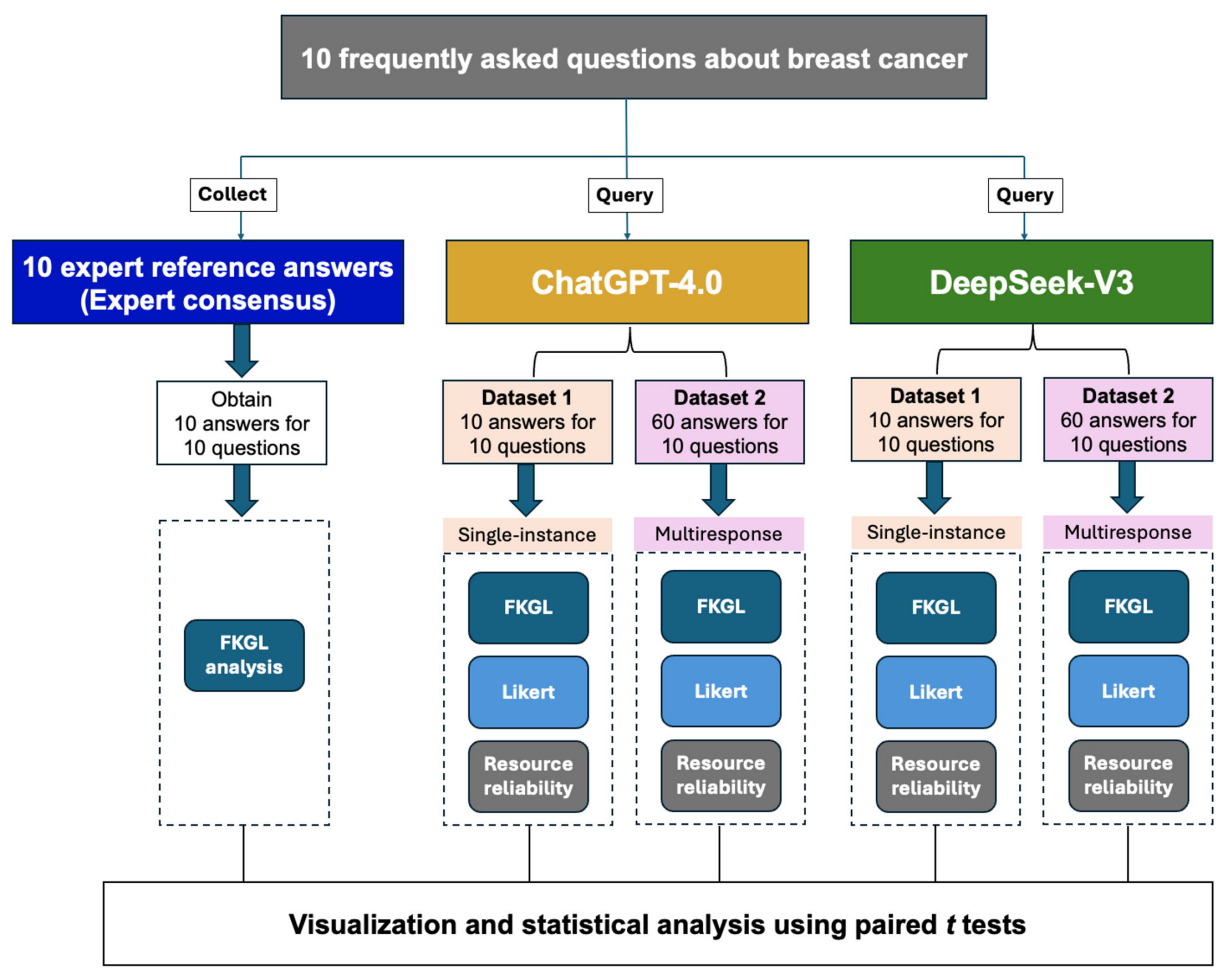
Workflow for evaluating the performance of ChatGPT and DeepSeek in retrieving and presenting medical information. FKGL: Flesch-Kincaid Grade Level.

This study evaluated the performance of generative AI models in answering 10 FAQs related to breast cancer as described in the workflow shown in Figure 1. The 10 validated questions and their corresponding expert reference answers were adopted from the study by Ye et al [31], which served as the benchmark dataset for this evaluation. In their foundational study, Ye et al [31] developed a list of FAQs regarding breast cancer and cervical cancer based on popular science materials and current hot topics related to tertiary prevention. A set of 10 questions was designed for each cancer type. Subsequently, experts were invited to review the proposed questions and to formulate expert consensus answers. The final set of questions and expert consensus answers were determined through several rounds of consultation and assessment.

### Data Collection and Processing

**Figure 2.**
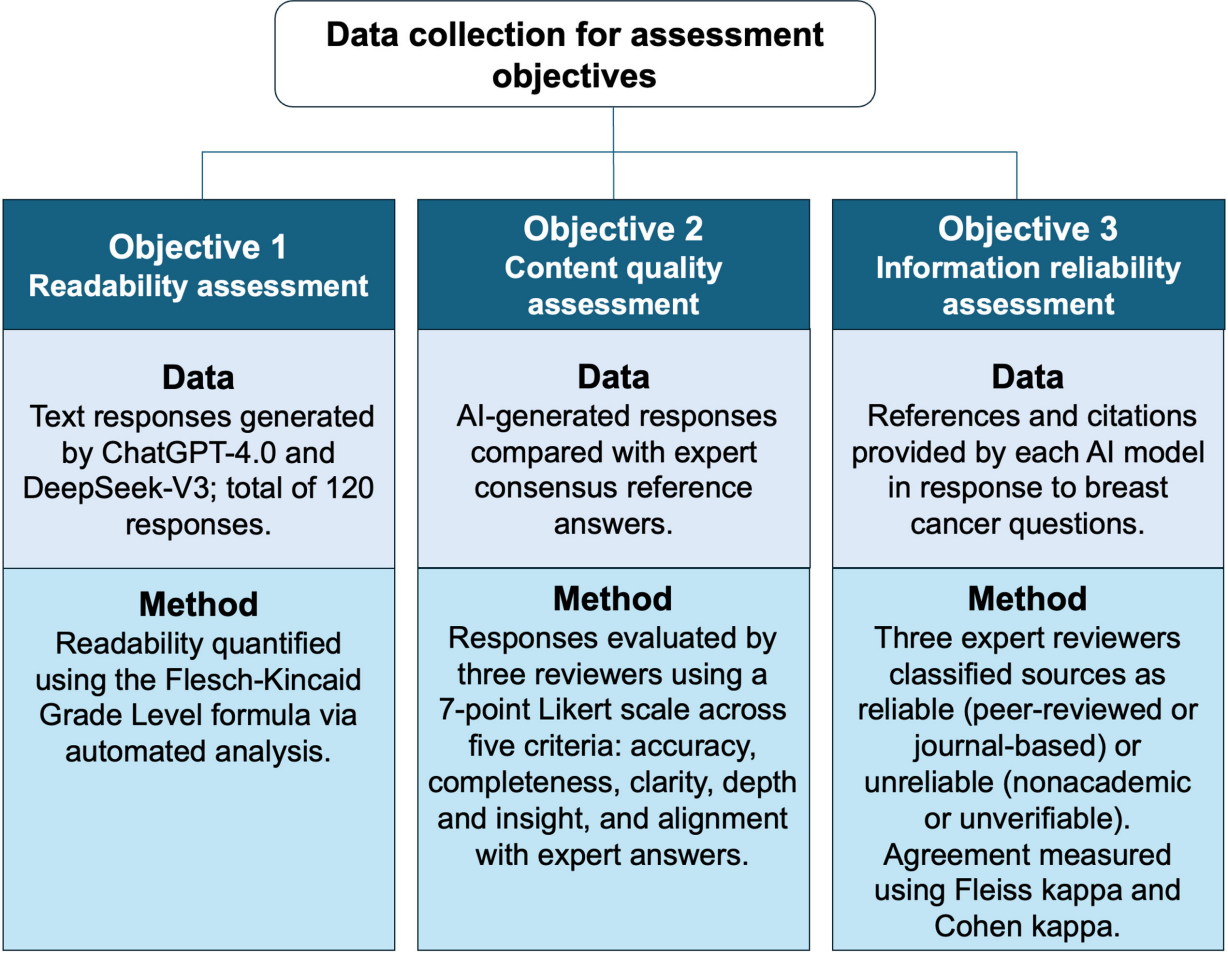
Workflow of data collection for assessment objectives. AI: artificial intelligence.

Figure 2 summarizes the data collection and assessment procedures used to evaluate the performance of ChatGPT-4.0 and DeepSeek-V3 across three core dimensions: readability, content quality, and information reliability. Each objective shown in Figure 2 was addressed using a tailored methodology aligned with specific types of data and evaluation criteria.

### AI Response Generation

To evaluate AI-generated content, we submitted 10 breast cancer FAQs to two generative AI models: ChatGPT-4.0 (OpenAI) and DeepSeek-V3 (DeepSeek). To ensure reproducibility and minimize bias, 3 independent researchers queried each model twice per question at different times, locations, and internet connections, yielding 6 responses per question per model (total: 60 responses per model and 120 responses overall).

### Expert Reference Answers

Expert reference answers for each FAQ were obtained from the consensus of 5 board-certified physicians specializing in mammography, as reported by Ye et al [31]. These physicians independently reviewed each FAQ and provided responses, which were then harmonized into a single consensus answer per question. This consensus served as the gold standard for evaluating AI performance.

The 10 breast cancer FAQs and the corresponding AI-generated answers from each independent researcher are provided in Tables S1-S3 in Multimedia Appendices 1-3, and the expert consensus reference answers are provided in Table S4 in Multimedia Appendix 4.

### Data Analysis

#### Readability Assessment

The readability evaluation was performed using the Flesch-Kincaid Grade Level (FKGL) analysis [34,35]. The textstat library (version 0.7.7; developed by Shivam Bansal, open-source project) [36] in Python was used to compute the FKGL scores for expert reference answers and responses generated by ChatGPT-4.0 and DeepSeek-V3. FKGL estimates the US school grade level required to understand a text, with higher scores indicating greater complexity. The FKGL score was calculated using the following formula:

$FKGL=0.39\left(\;\frac{Total\;words}{Total\;sentences}\;\right)+11.8\left(\;\frac{Total\;syllables}{Total\;words}\;\right)-15.59$

This analysis used two datasets: dataset 1, where expert reference answers were compared to the initial responses from ChatGPT-4.0 or DeepSeek-V3 for all questions (Q1-Q10); and dataset 2, where 3 researchers generated 2 responses each from ChatGPT-4.0 and DeepSeek-V3 for Q1-Q10, resulting in 60 responses per model. To evaluate statistical significance, paired *t* tests were conducted using the SciPy library (scipy.stats library; SciPy Developers, open-source project) within Python [37], as FKGL scores were normally distributed. These tests assessed mean differences between ChatGPT-4.0 and DeepSeek-V3 while ensuring paired comparisons across identical question sets.

#### Content Quality Assessment

Content quality was evaluated across five predefined dimensions: accuracy, completeness, clarity, depth and insight, and alignment with expert reference answers. These dimensions were adapted from established guidelines for assessing the quality of health information and AI-generated content [38,39]. Each AI-generated answer was independently scored by 3 expert reviewers using a 7-point Likert-type scale, where 1 represented poor performance and 7 represented excellent performance.

To compare the performance of ChatGPT-4.0 and DeepSeek-V3 across these dimensions, statistical analysis was performed using Python’s SciPy library (scipy.stats module, version 1.15.1; SciPy Developers, open-source project) [37]. The Shapiro-Wilk test was first applied to assess the normality of score distributions [40,41]. As the data met the normality assumptions, paired *t* tests [42] were conducted to compare mean scores across the 5 evaluation criteria. This approach enabled a robust and consistent comparison of content quality between the 2 models using identical question sets and standardized scoring methods.

#### Information Reliability Assessment

The information sources cited by ChatGPT-4.0 and DeepSeek-V3 were independently evaluated by 3 domain experts (RH, DAS, and SKB) with expertise in medicinal chemistry (all), molecular oncology (SKB), and computational drug discovery (RH and DAS). Each unique cited reference was assigned a binary reliability score: 1 for reliable sources (peer-reviewed journal articles, clinical guidelines, or official or authoritative bodies, such as national health agencies or major academic medical centers) and 0 for unreliable sources (commercial health media, foundation blogs, unsourced claims, or otherwise nonverifiable material). Complete source lists and scores are provided in Table S5 in Multimedia Appendix 5 (ChatGPT-4.0) and Table S6 in Multimedia Appendix 6 (DeepSeek-V3), and the full question-level reference data for both ChatGPT-4.0 and DeepSeek-V3 are provided in Table S7 in Multimedia Appendix 7.

For each AI model, we then performed 2 levels of agreement analysis. First, we assessed interrater reliability among the three human experts using Fleiss κ [43], and we also quantified the proportion of references with perfect agreement (all 3 raters assigned the same score), majority agreement (at least two raters agreed on the same score), and complete disagreement. Second, we evaluated how well each model aligned with human judgment by comparing the model’s score for each reference to the majority human score (the score agreed upon by ≥2 raters). Agreement between each AI model and this consensus was quantified using Cohen κ [44].

To further characterize disagreement, we identified references where ChatGPT or DeepSeek diverged from the expert majority score and summarized the frequency and nature of those disagreements. All scoring data (individual rater scores, AI-assigned scores, and majority scores) and all agreement calculations (Fleiss κ and Cohen κ) were generated in Python (pandas, NumPy, and scikit-learn) [45-47] and compiled into Microsoft Excel for audit trails, including disagreement tables and summary statistics for each model.

This evaluation framework allowed us to simultaneously measure (1) consistency among human raters when judging citation reliability and (2) how closely each AI system’s choices agreed with what experts considered acceptable evidence.

### Ethical Considerations

This study did not involve human participants, patient data, or intervention and therefore did not require review or approval by an institutional review board or research ethics committee. The analyses were based exclusively on AI-generated text responses and publicly available information sources, without collection of any personal, identifiable, or sensitive data. As no human subjects were recruited or interacted with, informed consent was not required, and no consent waiver was necessary. No compensation was provided. All data were handled in accordance with principles of data minimization and confidentiality, and no private or proprietary information was accessed or stored during the study.

## Results

### Study Design and Analytical Framework

To evaluate the performance of ChatGPT and DeepSeek, a structured analytical framework was implemented, as illustrated in Figure 1. A comparison of ChatGPT and DeepSeek was conducted using 10 FAQs about breast cancer, with responses evaluated against consensus expert reference answers as outlined in the Methods section. The workflow, illustrated in Figure 1, consisted of three key steps: data collection, model querying, and visual and statistical analysis. In the data collection phase, 10 common breast cancer questions were identified, and expert consensus answers were established. Three independent researchers then queried ChatGPT and DeepSeek, generating responses. Two datasets were created for each model: dataset 1, consisting of single-instance responses (1 response per question); and dataset 2, containing multiresponse outputs (multiple responses per question), resulting in 10 responses from each model for dataset 1 and 60 responses from each model for dataset 2Figure 1.

### Readability Assessment

**Figure 3.**
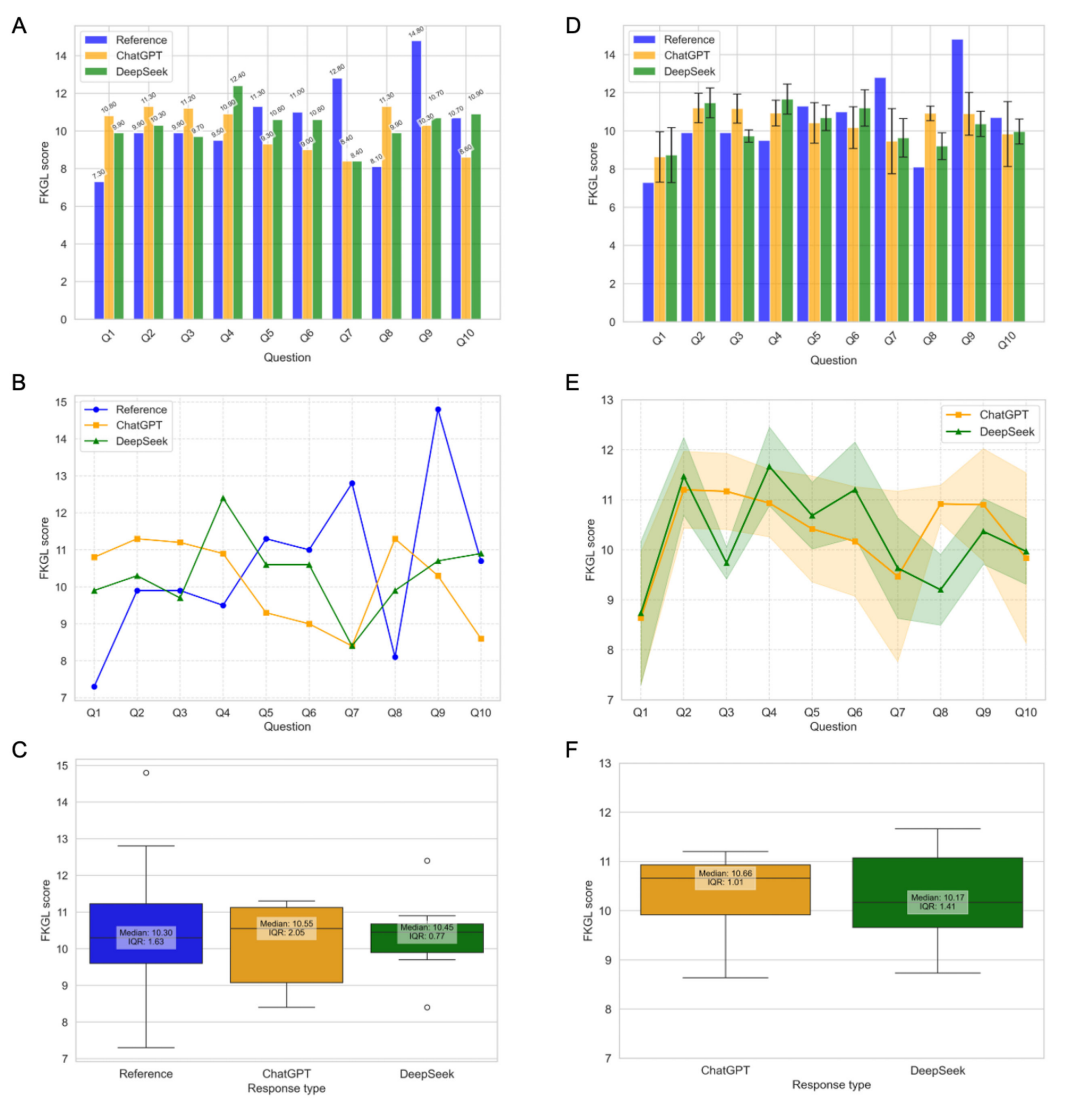
Readability assessment using Flesch-Kincaid Grade Level (FKGL) scores for expert reference answers, ChatGPT, and DeepSeek responses. (A) Bar plot comparing FKGL scores for 10 questions using 1 response per question from each platform. The bars represent the FKGL scores for reference, ChatGPT, and DeepSeek. (B) Line plot showing the overall trends of FKGL scores across all questions using 1 response per question from each platform. The lines represent the FKGL scores for reference, ChatGPT, and DeepSeek. (C) Box plot showing the distribution of FKGL scores using 1 response per question from each platform. The boxes represent the IQR, with the median annotated inside each box. Reference, ChatGPT, and DeepSeek are represented in blue, orange, and green, respectively. (D) Bar plot comparing FKGL scores for 10 questions using 6 responses per question per platform. The bars represent the average FKGL scores, with error bars indicating the SD. ChatGPT responses are shown in orange, and DeepSeek responses are shown in green. (E) Line plot showing the overall trends of FKGL scores for ChatGPT and DeepSeek responses across 10 questions using 6 responses per question per platform. The solid lines represent the mean FKGL scores, and the shaded regions indicate the SDs. (F) Box plot showing the distribution of FKGL scores for ChatGPT and DeepSeek responses across 10 questions using 6 responses per question per platform. The boxes represent the IQR, with the median annotated inside each box. The *y*-axis for all line and box plots ranges from 7 to 15 to ensure a clear comparison of FKGL variations.

The FKGL readability scores for responses generated by ChatGPT and DeepSeek, compared against expert reference answers, are presented in Tables1  2 and Figure 3. Two types of analyses were performed: single-instance analysis, where each AI model provided a single response per question; and multiresponse analysis, where multiple responses per question were collected by 3 researchers independently from both models.

**Table 1.** Comparison of readability scores of Flesch-Kincaid Grade Level (FKGL) of single-instance analysis for expert reference answers, ChatGPT-4.0, and DeepSeek-V3.

Question	Expert reference answer^a^	ChatGPT-4.0^b^		DeepSeek-V3^c^	
	FKGL score	FKGL score	Difference^d^	FKGL score	Difference^d^
Q1	7.30	10.80	3.50	9.90	2.60
Q2	9.90	11.30	1.40	10.30	0.40
Q3	9.90	11.20	1.30	9.70	−0.20
Q4	9.50	10.90	1.40	12.40	2.90
Q5	11.30	9.30	−2.00	10.60	−0.70
Q6	11.00	9.00	−2.00	10.60	−0.40
Q7	12.80	8.40	−4.40	8.40	−4.40
Q8	8.10	11.30	3.20	9.90	1.80
Q9	14.80	10.30	−4.50	10.70	−4.10
Q10	10.70	8.60	−2.10	10.90	0.20

a Statistics across all the responses for 10 questions are as follows: mean 10.53 (SD 2.17) and median 10.30 (IQR 1.63).

b Statistics across all the responses for 10 questions are as follows: mean 10.11 (SD 1.17) and median 10.55 (IQR 2.05).

c Statistics across all the responses for 10 questions are as follows: mean 10.53 (SD 1.02) and median 10.45 (IQR 0.77).

d Difference in comparison to reference. Positive difference indicates that the FKGL score was higher than that of reference, whereas negative difference means that the KFGL scores were lower than that of reference.

**Table 2.** Comparison of readability scores of Flesch-Kincaid Grade Level (FKGL) of multiresponse analysis for expert reference answers, ChatGPT-4.0, and DeepSeek-V3.

Question	Expert reference answer^a^	ChatGPT-4.0^b^			DeepSeek-V3^c^		
	FKGL score	FKGL score	Difference^d^	SD	FKGL score	Difference^d^	SD
Q1	7.30	8.63	1.33	1.32	8.73	1.43	1.43
Q2	9.90	11.20	1.30	0.77	11.47	1.57	0.78
Q3	9.90	11.17	1.27	0.76	9.73	−0.17	0.32
Q4	9.50	10.93	1.43	0.67	11.67	2.17	0.79
Q5	11.30	10.42	−0.88	1.06	10.68	−0.62	0.67
Q6	11.00	10.17	−0.83	1.09	11.2	0.20	0.96
Q7	12.80	9.47	−3.33	1.71	9.63	−3.17	1.00
Q8	8.10	10.92	2.82	0.38	9.20	1.10	0.71
Q9	14.80	10.90	−3.90	1.12	10.37	−4.43	0.66
Q10	10.70	9.83	−0.87	1.70	9.97	−0.73	0.66

a Statistics across all the responses for 10 questions are as follows: mean 10.53 (SD 2.17) and median 10.30 (IQR 1.63).

b Statistics across all the responses for 10 questions are as follows: mean 10.36 (SD 0.84) and median 10.66 (IQR 1.01).

c Statistics across all the responses for 10 questions are as follows: mean 10.27 (SD 0.98) and median 10.17 (IQR: 1.41).

d Difference in comparison to reference. Positive difference indicates that the FKGL score was higher than that of reference, whereas negative difference means that the FKGL scores were lower than that of reference.

In the single-instance analysis, ChatGPT’s mean FKGL score (10.11, SD 1.17) was slightly lower than the expert reference (10.53, SD 2.17), whereas DeepSeek’s mean score (10.53, SD 1.02) matched the expert reference answer exactly. In the multiresponse analysis, ChatGPT’s mean FKGL increased to 10.36 (SD 0.84), whereas DeepSeek’s mean FKGL slightly decreased to 10.27 (SD 0.98). ChatGPT demonstrated reduced variability in the multiresponse case (SD 0.84) compared to the single-instance case (SD 1.17), indicating greater consistency when averaging multiple responses. In contrast, DeepSeek’s variability remained relatively stable across both analyses (SD 1.02 in single instance and 0.98 in multiresponse).

In both analyses, DeepSeek’s median FKGL scores were slightly lower than ChatGPT’s, suggesting that DeepSeek’s responses may be more concise or readable. DeepSeek also exhibited less fluctuation in readability scores, as indicated by its smaller IQR 0.77 (median 10.45) in single instance and 1.41 (median 10.17) in multiresponse. In contrast, ChatGPT’s variability decreased in the multiresponse case, with its IQR narrowing from 2.05 (median 10.55) to 1.01 (median 10.66).

A detailed evaluation of FKGL readability scores reveals variations in how ChatGPT and DeepSeek generate responses compared to expert references. For some questions (eg, Q1, Q2, Q3, and Q4), both models produced more complex responses (higher FKGL scores), potentially reducing accessibility for general readers but could be more suitable for professionals and specialists seeking in-depth information.

In contrast, for other questions (eg, Q5, Q6, Q7, and Q9), DeepSeek generally remained closer to the reference readability (reference) but showed greater variability, whereas ChatGPT exhibited larger deviations, sometimes producing more complex or simpler responses (Figure 3A, B, D and E). The multiresponse analysis improved consistency (Figure 3F), smoothing out extreme deviations (eg, outliers evident in Figure 3C) and leading to more balanced readability. Full per-question FKGL values and paired comparisons for single-instance and multiresponse data are provided in Tables S8-S11 in MultimediaAppendices 8^11^.

A paired *t* test was conducted to assess the statistical significance of mean differences in FKGL scores among the expert reference answer, ChatGPT-4.0, and DeepSeek-V3 groups (Table 3). The Shapiro-Wilk test confirmed normality (*P*>.05 for all groups), validating the use of the *t* test. Results indicated no significant differences in readability scores between the expert reference answers and those generated by ChatGPT-4.0 or DeepSeek-V3, nor between ChatGPT-4.0 and DeepSeek-V3 (*P*=.61 for single instance and *P*=.73 for multiresponse). However, high variability in scores across questions was observed. Multiresponse analyses showed smaller differences between AI models and the reference, suggesting that averaging multiple responses yields more balanced readability. Normality was further confirmed by Shapiro-Wilk *P* values of .16 (ChatGPT-4.0) and .82 (DeepSeek-V3). The corresponding statistical summaries are provided in Tables S8 and S10 in MultimediaAppendices 8  10.

**Table 3.** Statistical comparison of readability (FKGL scores) among expert references, ChatGPT-4.0, and DeepSeek-V3 using paired *t* tests.

Comparison	Single-instance				Multiresponse			
	*t* test *(df)*^a^	*P* value^b^	95% CI^c^		*t* test *(df)*^a^	*P* value^b^	95% CI^c^	
ChatGPT-4.0 versus reference	−0.45 (9)	.66	−1.69 to 2.53		−0.24 (9)	.82	−1.40 to 1.74	
DeepSeek-V3 versus reference	−0.24 (9)	.81	−1.58 to 1.96		−0.40 (9)	.70	−1.25 to 1.78	
ChatGPT-4.0 versus DeepSeek-V3	0.52 (9)	.61	−1.23 to 0.77		0.35 (9)	.73	−0.53 to 0.72	

a *t* test *(df)*: measures the magnitude and direction of the difference between paired samples. A negative *t* value indicates that ChatGPT had lower scores than DeepSeek, while a positive *t* value indicates the opposite. *t* statistics are reported, all tests were 2-tailed paired *t* tests. Degrees of freedom shown in parentheses, and were calculated as n−1 for paired comparisons (*df*=9 for all single analyses).

b *P* value represents the probability of observing the test results under the null hypothesis. A *P* value ≤.05 is considered statistically significant.

c 95% CI for the mean difference.

### Content Quality Assessment

**Figure 4.**
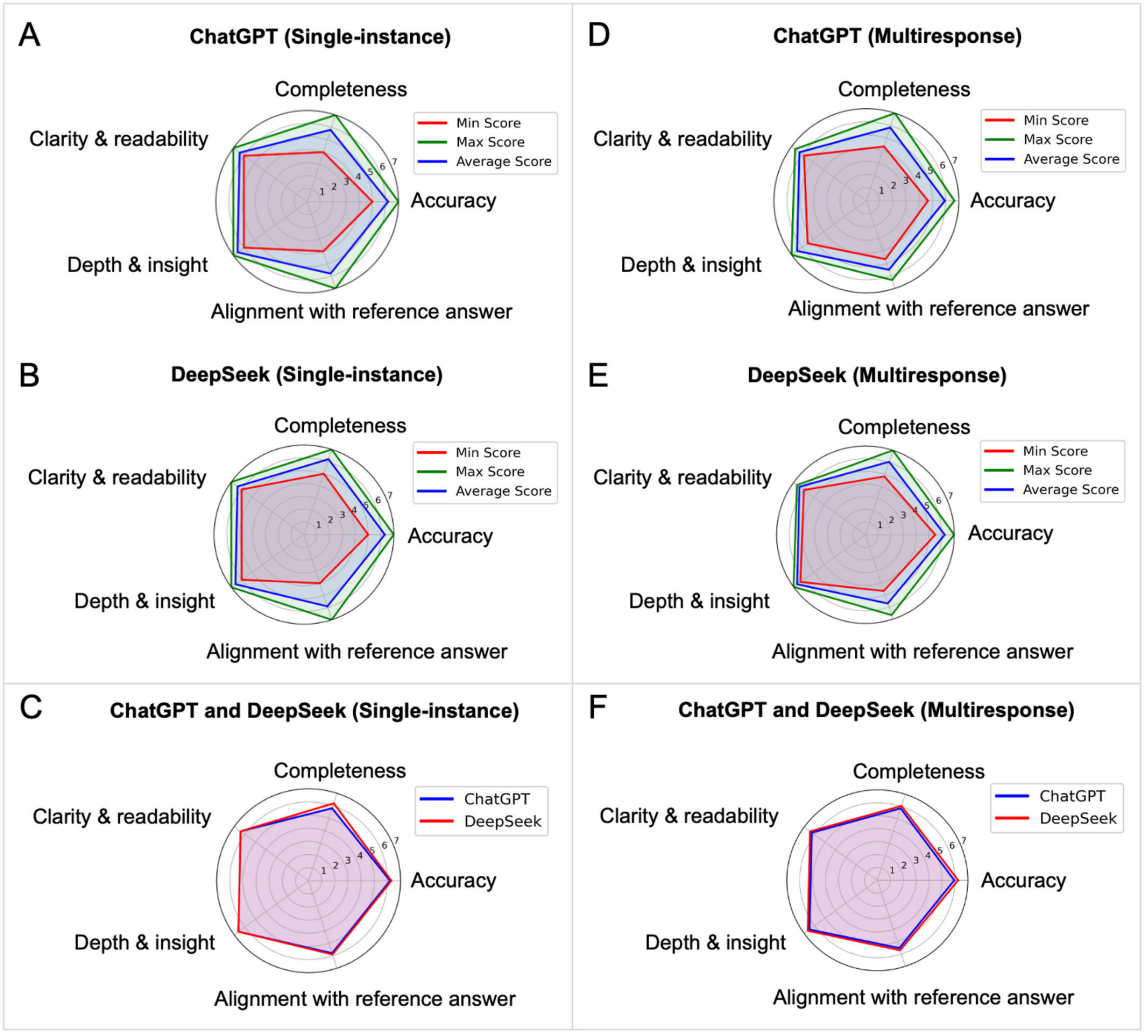
Content quality ratings for ChatGPT-4.0 and DeepSeek-V3 across 5 criteria. (A) ChatGPT response evaluation based on the average scores of 10 answers. (B) DeepSeek response evaluation based on average score of 10 answers. (C) ChatGPT versus DeepSeek comparison based on average scores of 10 answers. (D) ChatGPT response evaluation based on 60 answers. (E) DeepSeek response evaluation based on 60 answers. (F) ChatGPT versus DeepSeek comparison based on average scores of 10 answers.

Response quality was evaluated using a 7-point Likert scale across five criteria: accuracy, clarity and readability, completeness, depth and insight, and alignment with reference answers. In the single-response analysis, ChatGPT achieved an overall mean Likert score of 5.36 (SD 0.53), whereas DeepSeek had a slightly higher mean score of 5.60 (SD 0.45) across all criteria and questions. At the criterion level, ChatGPT scored higher in alignment and accuracy, whereas DeepSeek performed slightly better in the remaining criteria (Figure 4A–C).

In the multiresponse analysis (dataset 2), ChatGPT achieved a mean Likert score of 6.01 (SD 0.49), while DeepSeek achieved a comparable mean score of 6.22 (SD 0.43) across all criteria and responses (Figure 4D–F). Differences between the two models were minimal and not statistically significant (*P*>.05), with overlapping 95% CIs for completeness, clarity & readability, depth & insight, and alignment with the reference answer. However, DeepSeek outperformed ChatGPT based on accuracy (ChatGPT: 5.95 vs DeepSeek: 6.25). This difference was statistically significant (t₉ = −2.377, P = .041; 95% CI −0.585 to −0.015), indicating consistently higher accuracy scores for DeepSeek across the 10 questions.Overall, these findings indicate that both models provide comparably high-quality responses across the assessed dimensions. Detailed summary statistics are provided in Table 4, and full per-question and per-criterion Likert ratings for both the single-instance and multiresponse datasets are provided in Table S12 in Multimedia Appendix 12 and Table S13 in Multimedia Appendix 13.

**Table 4.** Comparison of ChatGPT-4.0 and DeepSeek-V3 content quality across 5 evaluation criteria using paired *t* tests for single- and multiresponse analyses.

Evaluation criterion	ChatGPT-4.0 score (mean)	DeepSeek-V3 score (mean)	*t* test (*df*)^a^	*P* value^b^	95% CI^c^	
Single-instance results						
Accuracy	6.20	6.30	−0.43 (9)	.68	−0.63 to 0.43	
Completeness	5.80	6.20	−1.50 (9)	.17	−1.00 to 0.20	
Clarity and readability	6.40	6.40	0.00 (9)	1.00	−0.34 to 0.34	
Depth and insight	6.60	6.60	0.00 (9)	1.00	−0.34 to 0.34	
Alignment with reference answer	5.80	5.90	−0.43 (9)	.68	−0.63 to 0.43	
Multiresponse results						
Accuracy	5.95	6.25	−2.38 (9)	.04	−0.59 to −0.01	
Completeness	5.85	6.05	−1.91 (9)	.09	−0.44 to 0.04	
Clarity and readability	6.27	6.43	−1.73 (9)	.12	−0.38 to 0.05	
Depth and insight	6.48	6.65	−1.79 (9)	.11	−0.38 to 0.04	
Alignment with reference answer	5.52	5.70	−1.49 (9)	.17	−0.46 to 0.09	

a *t* test: measures the magnitude and direction of the difference between paired samples. A negative *t* value indicates that ChatGPT had lower scores than DeepSeek, whereas a positive *t* value indicates the opposite, all tests were 2-tailed paired *t* tests Degrees of freedom shown in parentheses, and were calculated as n−1 for paired comparisons (*df*=9 for all analyses).

b *P* value represents the probability of observing the test results under the null hypothesis. A *P* value ≤.05 is considered statistically significant.

c 95% CI of the mean difference.

### Information Reliability Assessment

We analyzed the reliability of citations provided by ChatGPT-4.0 and DeepSeek-V3 in response to 10 clinically oriented breast cancer questions. For ChatGPT, 45 distinct references were extracted and scored by all 3 experts. Human raters showed very high internal consistency: Fleiss κ was 0.842, indicating almost perfect agreement. Of these 45 references, 40 (88.9%) received perfect agreement from all 3 raters, and 44 (97.8%) reached majority agreement (ie, at least two raters agreed on the same label). Only 1/45 (2.2%) reference showed complete disagreement among the raters. Detailed results are provided in Table S14 in Multimedia Appendix 14.

For DeepSeek, we evaluated 268 unique references. Interrater reliability among the three experts was even higher: Fleiss κ was 0.935, again in the *almost perfect* range. Across these 268 references, 260 (97.0%) had perfect agreement, and 267 (99.6%) had majority agreement. Only 1/268 references (0.4%) showed complete disagreement. These results confirm that, despite differences in style and depth of citation between the models, human experts were able to judge source reliability in a highly consistent way across both datasets. All details are provided in Table S15 in Multimedia Appendix 15.

We then assessed how well each AI model’s citation-level reliability scores aligned with human judgment (ie, each reference set was evaluated by both ChatGPT and DeepSeek as reported in Table S11 and Table S12 in MultimediaAppendices 11  12, respectively). For the ChatGPT reference set, agreement between ChatGPT’s scores and the human majority score was substantial (Cohen κ=0.665). For the DeepSeek reference set, agreement between DeepSeek’s scores and the human majority score was higher (κ=0.782). When disagreement occurred, ChatGPT differed from the human majority on 7 of its 45 references, whereas DeepSeek differed on 32 of its 268 references. These agreement metrics and disagreement profiles are summarized in Table S14 and Table 15 in MultimediaAppendices 7  14, respectively.

To enable direct comparison, we also evaluated each model on the other model’s reference set. In the ChatGPT set, DeepSeek’s scoring of those same sources achieved κ=0.800, which exceeded ChatGPT’s own κ=0.665*.* In the DeepSeek set, ChatGPT’s scoring of DeepSeek’s citations achieved κ=0.600, which was lower than DeepSeek’s κ=0.782. In other words, when both models were evaluated against the same expert consensus, DeepSeek was consistently closer to the human majority than ChatGPT.

Qualitatively, ChatGPT tended to cite large US medical organizations and public-facing health information portals (eg, national cancer societies, specialty associations, and patient education sites). These sources were commonly accepted as reliable when they represented established clinical authorities, but they occasionally triggered disagreement when they originated from advocacy foundations or commercial health media. DeepSeek more often cited recent peer-reviewed literature, systematic reviews, and oncology-specific clinical studies, which generally scored as reliable, although some entries contained incomplete metadata (eg, partial author or year strings or nonresolving links) that required manual adjudication.

Taken together, these results show 3 things. First, human expert scoring of reference quality was internally very stable for both models (Fleiss κ=0.842 for ChatGPT’s reference set and 0.935 for DeepSeek’s reference set) with majority agreement observed for 44/45 (97.8%) and 267/268 (99.6%) references, respectively, indicating that *reliable versus unreliable* source is a reproducible judgment in this context. Second, both AI systems demonstrated substantial alignment with expert consensus, but DeepSeek exhibited stronger agreement with the human majority than ChatGPT (κ up to 0.800 on the ChatGPT set and 0.782 on its own set, versus ChatGPT’s κ of 0.665 and 0.600*,* respectively). Third, the main source of residual disagreement was concentrated in borderline cases, such as advocacy organization content, institutional news posts, and partially specified citations, rather than in conventional peer-reviewed oncology literature (Table 5).

**Table 5.** Summary of citation reliability and agreement metrics for ChatGPT-4.0 and DeepSeek-V3.

Metric	ChatGPT-4.0	DeepSeek-V3
References evaluated, N	45	268
Reliability score (majority human; mean)	0.64	0.82
Fleiss κ (inter-rater agreement)	0.842	0.935
Perfect agreement (all 3 raters assigned the same label), n/N (%)	40/45 (88.9)	260/268 (97.0)
Majority agreement (≥2 of 3 raters assigned the same label), n/N (%)	44/45 (97.8)	267/268 (99.6)
Complete disagreement (all 3 raters assigned different labels), n/N (%)	1/45 (2.2)	1/268 (0.4)
Cohen κ (AI vs human majority)	0.665	0.782
Cross-evaluation κ (AI scoring the other model’s references)	ChatGPT on DeepSeek=0.600	DeepSeek on ChatGPT=0.800

The mean reliability score (majority human) is the mean binary reliability score (1=reliable and 0=unreliable) assigned by the human majority (≥2 of 3 expert raters) across all unique references cited by each model. “Fleiss κ” quantifies agreement among the 3 human experts for each model’s reference set. “Perfect agreement” is the proportion of references where all 3 experts independently gave the same reliability score. “Majority agreement” is the proportion of references where at least two of the 3 experts agreed. “Complete disagreement” refers to cases in which all 3 experts gave different scores. “Cohen κ (AI vs human majority score)” measures agreement between each model’s own reliability classification of its citations and the human majority classification for the same reference set. “Cross-evaluation κ” measures how well each model’s scoring of the other model’s references agreed with the human majority for that reference set (eg, “DeepSeek on ChatGPT”=DeepSeek’s κ when scoring the ChatGPT reference set). All underlying data are provided in Table S14 in Multimedia Appendix 7 (ChatGPT-4.0) and Table S15 in MultimediaAppendices 14  15 (DeepSeek-V3).

## Discussion

### Principal Findings

This study systematically evaluated the performance of ChatGPT-4.0 and DeepSeek-V3 in generating breast cancer information, focusing on readability, content quality, and reference quality. This multidimensional assessment approach (Figure 1), which is rarely addressed collectively in existing literature, offers valuable insights into the capabilities and limitations of generative AI models in health care. Both models generated clinically relevant and readable responses, with ChatGPT-4.0 showing greater consistency and overall readability, whereas DeepSeek-V3 offered more diverse citations but with greater variability and occasional technical issues.

### Readability Analysis

The FKGL readability scores revealed that AI-generated responses generally used simpler language structures than expert reference answers, which may enhance accessibility for general audiences. Expert responses had the highest FKGL scores (mean 10.53, SD 2.17), reflecting more complex vocabulary and sentence construction. In contrast, both ChatGPT and DeepSeek produced responses with lower FKGL scores, indicating simpler, more readable content.

This trend aligns with prior studies showing that LLMs often generate linguistically simplified outputs, which may benefit general users [38,39]. Notably, ChatGPT-4.0 exhibited more consistent readability across multiple outputs, with a median FKGL of 10.66 (IQR 0.98), compared to DeepSeek-V3, which showed greater variability with a median FKGL of 10.17 (IQR 1.41), suggesting greater stability with repeated querying.

For instance, ChatGPT-4.0 demonstrated improved readability consistency when averaging multiple responses, suggesting stability with repeated querying. In contrast, DeepSeek-V3’s responses were closer to expert reference readability levels in single-instance analysis but displayed greater variability in multiresponse cases (IQR 1.41).

However, statistical comparisons did not reveal significant differences in readability between models or relative to expert references. For instance, in the multiresponse setting, the paired *t* test for ChatGPT-4.0 versus reference yielded *t_9_*=−0.24; *P*=.82 (95% CI −1.40 to 1.74), and for DeepSeek-V3 versus reference, *t_9_*=−0.40; *P*=.70 (95% CI −1.25 to 1.78). The difference between ChatGPT and DeepSeek was also nonsignificant (*t*_9_=0.35; *P*=.73). Similar nonsignificant findings were observed in the single-instance analysis.

It is essential to emphasize that FKGL measures linguistic complexity, not clinical accuracy or information richness [35]. Lower FKGL scores do not necessarily indicate that critical medical details were omitted. Therefore, while improved readability may facilitate comprehension, further analysis is necessary to confirm that the content remains accurate, complete, and clinically appropriate.

### Content Quality Analysis

Beyond readability, content quality was assessed across 5 predefined evaluation criteria, including accuracy, completeness, clarity, depth and insight, and alignment with expert reference answers. This structured evaluation, based on established frameworks [38,39], allowed a more nuanced assessment of response quality. Across analyses, both models achieved high scores across all criteria. In the multiresponse dataset, DeepSeek achieved a higher overall mean score (6.22, SD 0.43) than ChatGPT (6.01, SD 0.49), with DeepSeek scoring numerically higher across all five criteria. Notably, DeepSeek demonstrated a statistically significant advantage in accuracy in the multiresponse analysis (t₉ = −2.377, *P* = .041; 95% CI −0.585 to −0.015), while differences for completeness, clarity and readability, depth and insight, and alignment with the reference answer did not reach statistical significance (P > .05). Interestingly, averaging multiple responses per question reduced response-level variability and revealed a more consistent performance advantage for DeepSeek, particularly for accuracy.

These findings expand on earlier studies that primarily focused on ChatGPT-3.5 [33] by demonstrating the evolving strengths and remaining limitations of newer generative AI models when applied to clinically relevant educational content. Unlike prior studies limited to single-response analysis, our multiresponse approach highlights how response variability remains an important factor when evaluating AI for health care education.

### Information Reliability Assessment

The reference reliability analysis revealed clear differences in both reference selection patterns and interrater scoring agreement for ChatGPT-4.0 and DeepSeek-V3 citations. ChatGPT tended to cite US-based medical institutions, national health organizations, and patient-facing educational resources, whereas DeepSeek more often cited recent peer-reviewed oncology studies and systematic reviews, although some DeepSeek citations were partially specified or contained inaccessible links. Human experts showed very consistent judgments of citation reliability for both models’ outputs: interrater reliability was almost perfect for both sets (Fleiss κ=0.842 for ChatGPT’s 45 references and 0.935 for DeepSeek’s 268 references), with ≥97% majority agreement and only one fully disputed reference in each set. When we compared each model’s scoring of source reliability to the majority expert score, agreement was substantial for ChatGPT and higher for DeepSeek. DeepSeek reached Cohen κ values up to 0.800 (for ChatGPT reference set) and 0.782 (for DeepSeek reference set), indicating strong alignment with expert consensus, whereas ChatGPT’s ability to assess references reached 0.665 for its own reference set and 0.600 when evaluated DeepSeek’s set.

Collectively, these results demonstrate that both models operate within the bounds of medically acceptable sourcing but use distinct strategies. DeepSeek’s output, favoring primary literature, more accurately reflects the evidence-based curation of oncology experts. Conversely, ChatGPT’s propensity for synthesized institutional guidance results in a higher frequency of citations to borderline sources that require expert judgment.

### Implications for Practice and Future Research

Collectively, these findings suggest that while generative AI models offer promising capabilities for generating patient education materials, important limitations remain. Oversimplification of complex medical information, variability in response consistency, and the reliability of cited sources remain persistent concerns. Given the growing interest in deploying AI tools for patient counseling, clinical decision support, and health care communication, these results underscore the need for careful validation of AI-generated content before clinical use [48]. Furthermore, while models such as DeepSeek demonstrate the potential for integrating primary literature more effectively, consistent quality control of both content and source reliability will be essential for safe application in clinical settings.

Finally, the structured multidimensional evaluation framework used in this study provides a replicable approach for future evaluations of AI models in health care. Future studies should continue to assess performance across broader clinical domains, expand evaluation across different languages and health literacy levels, and integrate clinical expert validation to further ensure the safety, accuracy, and equity of AI-generated medical information.

### Limitations and Future Directions

This study has several limitations. The FKGL test assessed structural readability but not contextual or clinical accuracy, and it is validated only for English, limiting generalizability across languages and literacy levels [20,34]. Additionally, the study was conducted in a controlled setting without patient or provider involvement, so real-world usability, comprehension, and clinical outcomes remain unexplored. Future work should include usability testing and assess impacts on decision-making and adherence [49]. Moreover, AI responses were not compared directly to clinical guidelines, and future studies should include validation against established standards before clinical integration. Reference accuracy remains a concern, as AI models may generate outdated or fabricated citations, requiring expert oversight [50].

Furthermore, the risk of bias in training data may lead to inaccuracies for underrepresented populations, highlighting the importance of ongoing monitoring and alignment with validated medical sources [38,48,49]. Evidence from clinical research, including variability in therapeutic outcomes across patient subgroups and molecular subtypes, further emphasizes the necessity for such validation [51,52]. Finally, ethical and regulatory considerations are essential for safe deployment of AI in health care; compliance with frameworks from regulatory bodies such as the Food and Drug Administration and the European Medicines Agency will be critical to ensure safety, transparency, and public trust [53]. As these technologies continue to evolve, continuous evaluation, comparative benchmarking, and expert oversight will be necessary to guide their responsible implementation.

### Conclusions

This comparative analysis of ChatGPT-4.0 and DeepSeek-V3 demonstrated that both AI platforms effectively retrieve and present medical information on breast cancer, each excelling in different areas. ChatGPT produced more polished, detailed, and readable responses, with strong performance in clarity and completeness, whereas DeepSeek outperformed ChatGPT on accuracy, and provided more comprehensive, globally diverse references with in-text citations resembling academic articles. However, DeepSeek faced challenges such as untagged links, occasional downtime, and corrupted references, which impacted the user experience. Despite these limitations, DeepSeek demonstrated superior citation efficiency and closely aligned with expert consensus answers. Moreover, interrater reliability analyses confirmed high levels of agreement among human evaluators across both models, with Fleiss κ scores exceeding 0.84 and Cohen κ values indicating substantial to almost perfect agreement. These findings support the overall quality of reference sources provided by the models and lend credibility to the evaluation framework used in this study. Furthermore, statistical analysis revealed no significant differences between the models across most evaluation criteria; however, in the multiresponse dataset (i.e., larger datasets), DeepSeek demonstrated a statistically significant advantage in accuracy, underscoring the importance of rigorous evaluation of AI-generated medical information to ensure accuracy, accessibility, and reliability. To optimize their effectiveness in health care applications, future improvements should focus on enhancing platform stability, response consistency, and overall user accessibility.

## Supplementary material

10.2196/72839 Multimedia Appendix 1 Frequently asked questions about breast cancer and answers generated by artificial intelligence models (ChatGPT and DeepSeek) by Researcher 1.

10.2196/72839 Multimedia Appendix 2 Frequently asked questions about breast cancer and answers generated by artificial intelligence models (ChatGPT and DeepSeek) by Researcher 2.

10.2196/72839 Multimedia Appendix 3 Frequently asked questions about breast cancer and answers generated by artificial intelligence models (ChatGPT and DeepSeek) by Researcher 3.

10.2196/72839 Multimedia Appendix 4 Frequently asked questions about breast cancer and expert consensus answers obtained from Ye et al.

10.2196/72839 Multimedia Appendix 5 Human experts and artificial intelligence reliability scores for references cited by ChatGPT-4.0.

10.2196/72839 Multimedia Appendix 6 Human experts and artificial intelligence reliability scores for references cited by DeepSeek-V3.

10.2196/72839 Multimedia Appendix 7 Information sources for ChatGPT-4.0 and DeepSeek-V3.

10.2196/72839 Multimedia Appendix 8 Average Flesch-Kincaid Grade Level scores for the single-response analysis and results of the paired *t* test.

10.2196/72839 Multimedia Appendix 9 Detailed Flesch-Kincaid Grade Level scores for the single-response analysis.

10.2196/72839 Multimedia Appendix 10 Average Flesch-Kincaid Grade Level scores for the multiresponse analysis and results of the paired *t* test.

10.2196/72839 Multimedia Appendix 11 Detailed Flesch-Kincaid Grade Level scores for the multiresponse analysis.

10.2196/72839 Multimedia Appendix 12 Likert scores for the single-instance analysis.

10.2196/72839 Multimedia Appendix 13 Likert scores for the multiresponse analysis.

10.2196/72839 Multimedia Appendix 14 Interrater evaluation of ChatGPT-4.0 references for breast cancer information based on ratings by 3 human experts and alignment with artificial intelligence models.

10.2196/72839 Multimedia Appendix 15 Interrater evaluation of DeepSeek-V3 references for breast cancer information based on ratings by three human experts and alignment with artificial intelligence models.

## Abbreviations:

AI: artificial intelligence
FAQ: frequently asked question
FKGL: Flesch-Kincaid Grade Level
LLM: large language model

## References

[1] Sung H, Ferlay J, Siegel RL (2021). Global cancer statistics 2020: GLOBOCAN estimates of incidence and mortality worldwide for 36 cancers in 185 countries. CA Cancer J Clin.

[2] Sinha A, Naskar M, Pandey M, Rautaray SS (2024). Challenges to the early diagnosis of breast cancer: current scenario and the challenges ahead. SN COMPUT SCI.

[3] Sunoqrot S, Abusulieh S, Abusara OH (2023). Identifying synergistic combinations of doxorubicin-loaded polyquercetin nanoparticles and natural products: implications for breast cancer therapy. Int J Pharm.

[4] Sweidan K, Elfadel H, Sabbah DA (2022). Novel derivatives of 4,6‐dihydroxy‐2‐quinolone‐3‐carboxamides as potential PI3Kα inhibitors. ChemistrySelect.

[5] (2023). Global Breast Cancer Initiative Implementation Framework: Assessing, Strengthening and Scaling-up of Services for the Early Detection and Management of Breast Cancer.

[6] Kasper G, Momen M, Sorice KA (2024). Effect of neighborhood and individual-level socioeconomic factors on breast cancer screening adherence in a multiethnic study. BMC Public Health.

[7] Chen J, Duan Y, Xia H, Xiao R, Cai T, Yuan C (2025). Online health information seeking behavior among breast cancer patients and survivors: a scoping review. BMC Womens Health.

[8] Loeb S, Langford AT, Bragg MA, Sherman R, Chan JM (2024). Cancer misinformation on social media. CA Cancer J Clin.

[9] Johnson SB, Parsons M, Dorff T (2022). Cancer misinformation and harmful information on Facebook and other social media: a brief report. J Natl Cancer Inst.

[10] Walker HL, Ghani S, Kuemmerli C (2023). Reliability of medical information provided by ChatGPT: assessment against clinical guidelines and patient information quality instrument. J Med Internet Res.

[11] Dave T, Athaluri SA, Singh S (2023). ChatGPT in medicine: an overview of its applications, advantages, limitations, future prospects, and ethical considerations. Front Artif Intell.

[12] Choi J, Kim JW, Lee YS (2024). Availability of ChatGPT to provide medical information for patients with kidney cancer. Sci Rep.

[13] Peng Y, Malin BA, Rousseau JF (2025). From GPT to DeepSeek: significant gaps remain in realizing AI in health care. J Biomed Inform.

[14] Kincaid JP, Braby R, Mears JE (1988). Electronic authoring and delivery of technical information. J Instr Dev.

[15] Sullivan GM, Artino AR (2013). Analyzing and interpreting data from likert-type scales. J Grad Med Educ.

[16] AlZu’bi S, Zreiqat A, Radi W, Mughaid A, Abualigah L (2024). An intelligent health care monitoring system-based novel deep learning approach for detecting covid-19 from x-rays images. Multimed Tools Appl.

[17] Muhairat M, Alzyadat W, Shaheen A, Alhroob A, Asfour AN (2024). Leveraging machine learning for predictive pathways in higher education: a case study at Al-Zaytoonah University of Jordan. SSRG-IJECE.

[18] Jarab AS, Al-Qerem W, Al-Hajjeh DM (2025). Artificial intelligence utilization in the health care setting: perceptions of the public in the UAE. Int J Environ Health Res.

[19] Buch VH, Ahmed I, Maruthappu M (2018). Artificial intelligence in medicine: current trends and future possibilities. Br J Gen Pract.

[20] Rajpurkar P, Chen E, Banerjee O, Topol EJ (2022). AI in health and medicine. Nat Med.

[21] Dilsizian SE, Siegel EL (2014). Artificial intelligence in medicine and cardiac imaging: harnessing big data and advanced computing to provide personalized medical diagnosis and treatment. Curr Cardiol Rep.

[22] Malik P, Pathania M, Rathaur VK (2019). Overview of artificial intelligence in medicine. J Family Med Prim Care.

[23] Sahu M, Gupta R, Ambasta RK, Kumar P (2022). Artificial intelligence and machine learning in precision medicine: a paradigm shift in big data analysis. Prog Mol Biol Transl Sci.

[24] Hajjo R, Sabbah DA, Al Bawab AQ (2022). Unlocking the potential of the human microbiome for identifying disease diagnostic biomarkers. Diagnostics (Basel).

[25] Boscardin CK, Gin B, Golde PB, Hauer KE (2024). ChatGPT and generative artificial intelligence for medical education: potential impact and opportunity. Acad Med.

[26] Hajjo R, Sabbah DA, Bardaweel SK, Tropsha A (2021). Identification of tumor-specific MRI biomarkers using machine learning (ML). Diagnostics (Basel).

[27] Wenderott K, Krups J, Weigl M, Wooldridge AR (2025). Facilitators and barriers to implementing AI in routine medical imaging: systematic review and qualitative analysis. J Med Internet Res.

[28] Yang YM, Wang T, Chan HY, Huang YM (2025). Key elements and theoretical foundations for the design and delivery of text messages to boost medication adherence in patients with diabetes, hypertension, and hyperlipidemia: scoping review. J Med Internet Res.

[29] Lai CC, Chen CY, Chang TH (2025). Predicting pathological complete response following neoadjuvant therapy in patients with breast cancer: development of machine learning-based prediction models in a retrospective study. JMIR Cancer.

[30] Xu F, Yang Y, Luo Y (2025). Efficacy of Fuzheng Quxie formula against postoperative metastasis of lung cancer in stage IIA-IIIA with negative driver genes: protocol for a multicenter, double-blind, randomized controlled trial. JMIR Res Protoc.

[31] Ye Z, Zhang B, Zhang K (2024). An assessment of ChatGPT’s responses to frequently asked questions about cervical and breast cancer. BMC Womens Health.

[32] A shocking chinese AI advancement called deepseek is sending US stocks plunging. CNN Business.

[33] Korea biomedical review. The DeepSeek dilemma: navigating innovation and security concerns in Korea’s health care industry.

[34] FLESCH R (1948). A new readability yardstick. J Appl Psychol.

[35] Badarudeen S, Sabharwal S (2008). Readability of patient education materials from the American Academy of Orthopaedic Surgeons and Pediatric Orthopaedic Society of North America web sites. J Bone Joint Surg Am.

[36] Textstat. Python Package Index.

[37] Virtanen P, Gommers R, Oliphant TE (2020). SciPy 1.0: fundamental algorithms for scientific computing in Python. Nat Methods.

[38] Johnson SB, King AJ, Warner EL, Aneja S, Kann BH, Bylund CL (2023). Using ChatGPT to evaluate cancer myths and misconceptions: artificial intelligence and cancer information. JNCI Cancer Spectr.

[39] Haver HL, Ambinder EB, Bahl M, Oluyemi ET, Jeudy J, Yi PH (2023). Appropriateness of breast cancer prevention and screening recommendations provided by ChatGPT. Radiology.

[40] Shapiro SS, Wilk MB (1965). An analysis of variance test for normality (complete samples). Biometrika.

[41] Arnastauskaitė J, Ruzgas T, Bražėnas M (2021). An exhaustive power comparison of normality tests. Mathematics.

[42] Ruxton GD (2006). The unequal variance t-test is an underused alternative to Student’s t-test and the Mann–Whitney U test. Behav Ecol.

[43] Fleiss JL (1971). Measuring nominal scale agreement among many raters. Psychol Bull.

[44] Cohen J (1960). A coefficient of agreement for nominal scales. Educ Psychol Meas.

[45] Harris CR, Millman KJ, van der Walt SJ (2020). Array programming with NumPy. Nature New Biol.

[46] Scikit-learn: machine learning in python — scikit-learn 170 documentation. Scikit-learn.

[47] Python data analysis library. pandas.

[48] Vellido A (2019). Societal issues concerning the application of artificial intelligence in medicine. Kidney Dis (Basel).

[49] Kitsios F, Kamariotou M, Syngelakis AI, Talias MA (2023). Recent advances of artificial intelligence in health care: a systematic literature review. Appl Sci (Basel).

[50] Lee P, Bubeck S, Petro J (2023). Benefits, limits, and risks of GPT-4 as an AI chatbot for medicine. N Engl J Med.

[51] Hajjo R, Sabbah DA, Bardaweel SK, Zhong HA (2024). Targeting the EGFR/RAS/RAF signaling pathway in anticancer research: a recent update on inhibitor design and clinical trials (2020-2023). Expert Opin Ther Pat.

[52] Sabbah DA, Hajjo R, Bardaweel SK, Zhong HA (2024). Targeting the PI3K/AKT signaling pathway in anticancer research: a recent update on inhibitor design and clinical trials (2020-2023). Expert Opin Ther Pat.

[53] Pasas-Farmer S, Jain R (2025). From discovery to delivery: governance of AI in the pharmaceutical industry. Green Analytical Chemistry.

[54] Hajjo R AI-models [github repository]. GitHub.

