# Supplementary material for: ChatGPT Versus DeepSeek for Breast Cancer Information Retrieval: Quantitative Comparative Study
Source: JMIR Cancer. 2026 Feb 27;12:e72839. doi: 10.2196/72839 (PMC12954694; doi:10.2196/72839)
Supplement: Multimedia Appendix 1 — Frequently asked questions about breast cancer and answers generated by artificial intelligence models (ChatGPT and DeepSeek) by Researcher 1. [file cancer-v12-e72839-s001.docx]

**Supporting Information: Supplementary Table 1**

**ChatGPT vs. DeepSeek: A Comparative Analysis of AI Models for Breast Cancer Information Retrieval**

Rima Hajjo^1,2*^, Dima A. Sabbah^1^, Sanaa K. Bardaweel^3^

*^1^Department of Pharmacy, Faculty of Pharmacy, Al-Zaytoonah University of Jordan, P.O. Box 130, Amman 11733, Jordan.*

*^2^Laboratory for Molecular Modeling, Division of Chemical Biology and Medicinal Chemistry, Eshelman School of Pharmacy, The University of North Carolina at Chapel Hill, Chapel Hill, NC, USA.*

*^3^Department of Pharmaceutical Sciences, School of Pharmacy, University of Jordan, Amman 11942, Jordan.*

*Correspondence

Rima Hajjo

E-mail: rhajjo@gmail.com; [r.hajjo@zuj.edu.jo](mailto:r.hajjo@zuj.edu.jo)

ORCID: https://orcid.org/0000-0002-7090-5425

**Supplementary Table 1.** Frequently asked questions about breast cancer and answers generated by artificial intelligence models (ChatGPT and DeepSeek) by Researcher 1.

| **ChatGPT 4.0** | | |
| --- | --- | --- |
|  | **Question** | **Answer** |
| 1 | What are the common symptoms of breast cancer? | Breast cancer symptoms can vary depending on the type, stage, and individual factors. Here are the most common symptoms to watch for:  1. Lump or Mass in the Breast or Underarm  A painless, hard lump with irregular edges (most common symptom)  Some lumps may be soft, tender, or painful  Can appear in the breast or armpit (axillary lymph nodes)  2. Changes in Breast Shape or Size  One breast becoming larger, swollen, or misshapen  Asymmetry (one breast looking different from the other)  3. Skin Changes on the Breast  Dimpling or puckering (like an orange peel, known as "peau d'orange")  Redness, warmth, or dark patches (may indicate inflammation)  Scaly or thickened skin on the breast or nipple  4. Nipple Changes  Inverted nipple (suddenly pulling inward)  Discharge (clear, bloody, or unusual fluid from the nipple)  Crusting, peeling, or flaking around the nipple area  5. Persistent Breast Pain or Tenderness  Ongoing pain not related to menstrual cycles  Burning or aching sensations in the breast or nipple  6. Swelling or Lumps in the Armpit or Collarbone Area  May indicate spread to lymph nodes  Swollen or firm lumps that don't go away  7. Unexplained Weight Loss or Fatigue (Advanced Cases)  Feeling tired or weak without a clear reason  Losing weight unintentionally  When to See a Doctor?  If you feel a new lump or notice any unusual changes in your breasts  If symptoms persist for more than two weeks  If you have a family history of breast cancer  Early detection saves lives! Regular breast self-exams, clinical exams, and mammograms are crucial for early diagnosis.  Would you like recommendations for breast cancer screening tests? |
|  | Repeat | The common symptoms of breast cancer include:  1. Changes in the Breast or Nipple  A lump or thickening in the breast or underarm (most common symptom)  Change in the size or shape of the breast  Swelling in part of the breast (even without a lump)  Dimpling or puckering of the breast skin (may look like an orange peel)  2. Changes in the Nipple  Nipple retraction (turning inward)  Discharge from the nipple, especially if bloody or clear  Pain or tenderness in the nipple or breast (though most breast cancers are painless)  3. Skin Changes  Redness, scaling, or thickening of the nipple or breast skin  Itchy, scaly rash (may be a sign of Paget’s disease, a rare breast cancer)  4. Pain or Discomfort  Persistent breast or nipple pain (though breast pain is more commonly due to benign causes)  5. Unexplained Symptoms  Swollen lymph nodes in the armpit or collarbone area (may indicate the cancer has spread)  Unexplained weight loss or fatigue (in more advanced cases)  If any of these symptoms are present, it’s important to see a doctor for evaluation. Many breast changes are not cancer, but early detection improves outcomes significantly.  Would you like information on risk factors or screening methods as well? |
|  | Repeat | Common symptoms of breast cancer can vary, but here are some of the most frequent signs to watch for:  1. Lump or Thickening in the Breast or Underarm  A new, painless or painful lump that feels different from surrounding tissue.  Swelling in or around the breast, collarbone, or armpit.  2. Changes in Breast Shape or Size  Unexplained increase or decrease in breast size.  Uneven or sudden distortion of breast shape.  3. Skin Changes on the Breast  Dimpling or puckering, resembling an orange peel (peau d'orange).  Redness, scaling, or thickening of the skin or nipple.  Ulcers or sores that don’t heal.  4. Nipple Changes  Inversion (nipple pulling inward) or flattening.  Discharge (bloody, clear, or yellow fluid) from the nipple.  Persistent pain or itchiness in the nipple area.  5. Breast Pain or Discomfort  Persistent, localized pain that doesn’t go away.  Tenderness in one breast without an obvious reason.  6. Swelling in Lymph Nodes  Swollen lymph nodes in the armpit or around the collarbone.  When to See a Doctor?  If you notice any of these symptoms, it's essential to get a medical evaluation as soon as possible. Many breast changes are not cancerous, but early detection significantly improves treatment outcomes.  Would you like information on screening tests or risk factors? |
| 2 | What are the risk factors for breast cancer? | Risk Factors for Breast Cancer  Breast cancer risk is influenced by a combination of genetic, hormonal, lifestyle, and environmental factors. Some factors are modifiable, while others are beyond control.  1. Non-Modifiable Risk Factors (Cannot Be Changed)  Age  The risk increases with age, especially after 50.  Gender  Women are at a much higher risk than men, though men can also develop breast cancer.  Genetics & Family History  Having first-degree relatives (mother, sister, daughter) with breast cancer increases risk.  Inherited gene mutations, such as BRCA1 and BRCA2, significantly raise the risk.  Personal History of Breast Cancer or Certain Breast Diseases  If you've had breast cancer before, the risk of developing it again is higher.  Conditions like atypical hyperplasia or lobular carcinoma in situ (LCIS) increase risk.  Hormonal Factors  Early menstruation (before age 12) or late menopause (after age 55) increases risk due to prolonged estrogen exposure.  Never having children or having the first child after 30 slightly increases risk.  2. Modifiable Risk Factors (Can Be Changed)  Hormone Therapy  Long-term use of hormone replacement therapy (HRT) (especially estrogen + progesterone) after menopause raises risk.  Certain birth control pills may slightly increase risk.  Lifestyle & Environmental Factors  Alcohol consumption (even moderate drinking) increases breast cancer risk.  Obesity, especially after menopause, raises estrogen levels.  Lack of physical activity is linked to higher risk.  Smoking is associated with an increased risk, especially in premenopausal women.  Radiation Exposure  Prior radiation therapy to the chest (e.g., for Hodgkin’s lymphoma) increases breast cancer risk.  Dietary Factors  High intake of processed foods, unhealthy fats, and red meat may contribute to increased risk.  3. Emerging & Controversial Risk Factors  Night shift work (possible link due to hormone disruption).  Exposure to endocrine-disrupting chemicals (e.g., BPA in plastics, parabens in cosmetics).  Dense breast tissue (makes tumors harder to detect on mammograms and may increase risk).  Can You Reduce Your Risk?  Yes! While some risk factors can't be changed, you can lower your risk by:  ✅Maintaining a healthy weight  ✅ Exercising regularly (150 minutes per week)  ✅ Limiting alcohol and smoking  ✅ Eating a balanced diet rich in fruits, vegetables, and lean proteins  ✅ Breastfeeding (if possible)  ✅ Considering alternative hormone therapy options after menopause  Would you like recommendations on breast cancer screening or genetic testing? |
|  | Repeat | The risk factors for breast cancer can be categorized into modifiable (lifestyle-related) and non-modifiable (biological/genetic) factors.  1. Non-Modifiable Risk Factors (Biological/Genetic)  These are factors you cannot change, but they may increase your risk.  Age: Risk increases with age, especially after 50.  Gender: Women are at a much higher risk than men.  Family History & Genetics:  A close relative (mother, sister, daughter) with breast cancer.  Inherited BRCA1 or BRCA2 gene mutations (increase risk significantly).  Personal History of Breast Cancer or Certain Non-Cancerous Breast Conditions:  Previous breast cancer increases risk of recurrence.  Conditions like atypical hyperplasia or lobular carcinoma in situ (LCIS).  Hormonal Factors:  Early menstruation (before age 12) or late menopause (after age 55).  Never having children or having the first child after 30.  Hormone replacement therapy (HRT) (especially estrogen-progesterone combination).  Dense Breast Tissue: Makes it harder to detect tumors on mammograms and increases risk.  2. Modifiable Risk Factors (Lifestyle-Related)  These are factors you can change to lower your risk.  Obesity & Weight Gain: Especially after menopause, as fat cells produce estrogen.  Alcohol Consumption: Even one drink per day increases risk.  Lack of Physical Activity: Regular exercise reduces risk.  Smoking: Increases risk, especially in premenopausal women.  Dietary Factors: High-fat and processed food diets may contribute to risk.  Radiation Exposure: Prior radiation therapy to the chest area (e.g., for Hodgkin’s lymphoma).  3. Environmental & Other Factors  Night Shift Work: Linked to higher risk due to disruptions in circadian rhythms.  Exposure to Chemicals & Pollutants: Some endocrine disruptors in plastics, cosmetics, and pesticides may play a role.  Reducing Your Risk  Maintain a healthy weight.  Exercise regularly (150 minutes per week of moderate activity).  Limit alcohol intake (or avoid it).  Avoid smoking.  Breastfeed if possible (linked to a reduced risk).  Get regular screenings and mammograms.  Would you like guidance on breast cancer screening recommendations? |
| 3 | How to prevent breast cancer? | Breast Cancer Prevention Strategies  While breast cancer cannot always be prevented, there are several lifestyle changes and medical interventions that can significantly reduce the risk.  1. Maintain a Healthy Lifestyle  ✔️ Stay Physically Active  Aim for at least 150 minutes of moderate exercise or 75 minutes of vigorous exercise per week.  Activities like walking, jogging, yoga, and strength training can help reduce risk.  ✔️ Maintain a Healthy Weight  Obesity, especially after menopause, increases estrogen levels, raising the risk.  A balanced diet and regular exercise can help with weight management.  ✔️ Eat a Nutritious Diet  Increase intake of fruits, vegetables, whole grains, and lean proteins.  Reduce consumption of processed foods, red meat, and unhealthy fats.  Include anti-inflammatory and antioxidant-rich foods like berries, leafy greens, and nuts.  Consume omega-3 fatty acids from sources like salmon, flaxseeds, and walnuts.  ✔️ Limit Alcohol Consumption  Even moderate alcohol consumption increases breast cancer risk.  Limit intake to no more than one drink per day, or avoid alcohol completely.  ✔️ Avoid Smoking  Smoking has been linked to an increased risk of breast cancer, especially in premenopausal women.  2. Hormonal & Reproductive Factors  ✔️ Breastfeed, If Possible  Breastfeeding for at least six months has been shown to reduce breast cancer risk.  ✔️ Limit Hormone Replacement Therapy (HRT)  If HRT is necessary for menopause symptoms, use the lowest effective dose for the shortest possible time.  Discuss non-hormonal alternatives with your doctor.  ✔️ Consider Birth Control Choices Carefully  Oral contraceptives may slightly increase risk, though the risk returns to normal after stopping use.  Weigh the benefits and risks with your healthcare provider.  3. Regular Screening & Early Detection  ✔️ Perform Regular Breast Self-Exams (BSE)  Become familiar with how your breasts normally look and feel.  Check for lumps, skin changes, or nipple discharge and report any unusual changes to your doctor.  ✔️ Get Regular Mammograms  Women aged 40+ should discuss mammogram screening frequency with their doctor.  Women at higher risk (family history, genetic mutations) may need earlier or more frequent screening.  ✔️ Consider Genetic Testing & Preventive Measures  If you have a strong family history of breast cancer, genetic testing for BRCA1, BRCA2, or other mutations may be recommended.  Preventive options like risk-reducing medications (Tamoxifen, Raloxifene) or preventive mastectomy (for high-risk individuals) can be discussed with a specialist.  4. Reduce Environmental & Workplace Exposures  Avoid exposure to harmful chemicals found in plastics, pesticides, and cosmetics (e.g., BPA, parabens, phthalates).  Limit radiation exposure (only undergo medical imaging like X-rays and CT scans when necessary).  Key Takeaway  ✅ Live an active, healthy lifestyle.  ✅ Get regular check-ups and screenings.  ✅ Be mindful of hormone therapy and birth control.  ✅ Avoid harmful substances like alcohol, tobacco, and toxic chemicals.  Would you like a personalized risk assessment or more details on genetic testing? 😊 |
|  | Repeat | Breast Cancer Prevention Strategies  While not all cases of breast cancer can be prevented, certain lifestyle changes and risk-reducing strategies can help lower the risk. Here’s what you can do:  1. Maintain a Healthy Lifestyle  ✅ Stay Physically Active  Aim for at least 150 minutes of moderate exercise or 75 minutes of vigorous activity per week.  Activities like brisk walking, jogging, swimming, or yoga can help.  ✅ Maintain a Healthy Weight  Obesity, especially after menopause, increases breast cancer risk due to higher estrogen levels.  A balanced diet and regular exercise can help maintain a healthy weight.  ✅ Eat a Healthy Diet  Focus on fruits, vegetables, whole grains, and lean proteins.  Limit processed foods, red meat, and saturated fats.  Mediterranean diet (rich in healthy fats like olive oil and nuts) may help lower risk.  ✅ Limit Alcohol Intake  Even one drink per day can increase risk.  Best choice: Avoid alcohol or limit to less than 3 drinks per week.  ✅ Avoid Smoking  Smoking is linked to increased breast cancer risk, especially in younger women.  2. Hormone Regulation & Reproductive Health  ✅ Breastfeed, if possible  Women who breastfeed for at least 6 months have a lower risk.  ✅ Limit Hormone Therapy (HRT & Birth Control)  Hormone Replacement Therapy (HRT) used during menopause increases risk.  If needed, use the lowest effective dose for the shortest time.  Oral contraceptives (birth control pills) slightly increase risk but decrease risk of ovarian and endometrial cancer. Discuss pros and cons with your doctor.  3. Reduce Environmental Exposures  ✅ Avoid Exposure to Toxins & Radiation  Reduce exposure to pesticides, industrial chemicals, and plastics with hormone-disrupting chemicals.  Limit unnecessary radiation exposure (X-rays, CT scans, etc.).  ✅ Work on a Healthy Sleep Cycle  Night shift work and disruptions in circadian rhythms may increase risk.  Try to maintain consistent sleep patterns.  4. Regular Screening & Early Detection  ✅ Get Regular Mammograms  Women 40+ (or earlier if high-risk) should get regular mammograms.  Self-exams help detect changes, but screening is key.  ✅ Know Your Family History  If you have a strong family history or a BRCA1/BRCA2 mutation, talk to your doctor about genetic counseling and preventive strategies.  ✅ Consider Preventive Medications or Surgery (For High-Risk Individuals)  Tamoxifen & Raloxifene (for women at high risk) can reduce breast cancer chances.  Preventive Mastectomy (for BRCA1/BRCA2 carriers) is an option for very high-risk individuals.  Key Takeaway  Healthy lifestyle choices significantly lower risk.  Early detection through screening is crucial.  Know your personal risk and take steps accordingly.  Would you like recommendations for a personalized risk assessment? 😊 |
| 4 | Can breast cancer be inherited? | Can Breast Cancer Be Inherited?  Yes, breast cancer can be inherited, but only about 5-10% of cases are directly linked to genetic mutations passed down in families. The majority of breast cancer cases are sporadic (occur due to aging and environmental factors).  1. Genetic Mutations & Inherited Risk  ✔️ BRCA1 and BRCA2 Mutations  The most well-known inherited mutations are in the BRCA1 and BRCA2 genes.  These genes normally help repair DNA damage and prevent cancer.  Mutations in BRCA1 or BRCA2 significantly increase the risk of:  Breast cancer (up to 70% lifetime risk)  Ovarian cancer (up to 45% lifetime risk for BRCA1, 17% for BRCA2)  Increased risk for pancreatic, prostate, and male breast cancer  ✔️ Other Inherited Gene Mutations  Besides BRCA1/2, other gene mutations linked to breast cancer include:  TP53 (Li-Fraumeni Syndrome) – Causes early-onset breast cancer.  CHEK2, ATM, PALB2 – Moderate increase in breast cancer risk.  PTEN (Cowden Syndrome) – Increases risk of breast and other cancers.  ✔️ Family History & Risk  If a first-degree relative (mother, sister, daughter) had breast cancer, your risk doubles.  If multiple family members (on either parent's side) had breast cancer, the risk is even higher.  If a male relative had breast cancer, genetic factors may be involved.  2. How to Know if You Have an Inherited Risk?  ✔️ Consider Genetic Testing if You Have:  ✅ A strong family history of breast or ovarian cancer  ✅ A relative diagnosed with breast cancer before age 50  ✅ A family member with BRCA1/2 or another genetic mutation  ✅ A history of male breast cancer in the family  ✅ Ashkenazi Jewish ancestry, as BRCA1/2 mutations are more common  ✔️ What If You Test Positive?  Increased screening: More frequent mammograms, MRIs starting at a younger age.  Preventive medications: Risk-reducing drugs like Tamoxifen or Raloxifene.  Preventive surgery: Some women choose prophylactic mastectomy (removal of breasts) or oophorectomy (removal of ovaries).  3. What If You Don’t Have a Family History?  Most breast cancer cases (90-95%) are NOT inherited.  Lifestyle factors, aging, and environmental exposures play a bigger role.  Regular screenings and healthy habits can still significantly reduce your risk.  Would you like guidance on genetic testing options or preventive measures? 😊 |
|  | Repeat | Can Breast Cancer Be Inherited?  Yes, breast cancer can be inherited, but only about 5-10% of cases are due to genetic mutations passed down in families. The majority of breast cancer cases are sporadic (not inherited) and result from lifestyle, environmental, and age-related factors.  1. Genetic Mutations Linked to Hereditary Breast Cancer  The most well-known inherited genetic mutations that increase breast cancer risk are:  ✅ BRCA1 & BRCA2 Mutations  BRCA1 and BRCA2 genes help suppress tumors, but when mutated, they increase the risk of breast and ovarian cancer.  Women with BRCA1 mutations have up to a 72% lifetime risk of breast cancer.  Women with BRCA2 mutations have up to a 69% lifetime risk.  Men with BRCA mutations also have a higher risk of male breast cancer, prostate cancer, and pancreatic cancer.  ✅ Other Less Common Gene Mutations  TP53 (Li-Fraumeni Syndrome) → High risk of early breast cancer.  PALB2 → Second highest risk after BRCA mutations.  CHEK2, ATM, PTEN, CDH1, and STK11 → Moderate increase in risk.  2. Signs That Breast Cancer May Be Inherited in a Family  If any of the following apply to you, consider genetic testing for inherited mutations:  ✅ Multiple close relatives (mother, sister, aunt, grandmother) had breast or ovarian cancer.  ✅ A relative was diagnosed with breast cancer before age 50.  ✅ Family history of male breast cancer.  ✅ Bilateral breast cancer (cancer in both breasts).  ✅ A family member has a known BRCA mutation.  ✅ Family history of other cancers (ovarian, pancreatic, prostate).  ✅ Ashkenazi Jewish ancestry, as BRCA mutations are more common.  3. What to Do If You Have a Family History?  ✅ Genetic Testing: If you have a strong family history, a genetic test can help assess your risk.  ✅ Increased Screening: Women with BRCA mutations should begin mammograms and MRIs earlier (age 25-30).  ✅ Preventive Medications: Drugs like Tamoxifen or Raloxifene may help lower risk.  ✅ Preventive Surgery: Some high-risk individuals choose prophylactic mastectomy or oophorectomy (removal of ovaries) to reduce cancer risk.  Key Takeaways  🔹 Inherited genetic mutations cause only 5-10% of breast cancers.  🔹 BRCA1, BRCA2, and other gene mutations significantly increase risk.  🔹 A strong family history may indicate a higher inherited risk.  🔹 Genetic testing and preventive strategies can help high-risk individuals.  Would you like guidance on genetic testing options or how to assess your personal risk? 😊 |
| 5 | Why is breast cancer screening important? | Why Is Breast Cancer Screening Important?  Breast cancer screening is crucial for early detection, which significantly improves treatment success and survival rates. Here’s why it matters:  1. Detects Cancer Early, Before Symptoms Appear  Early-stage breast cancer is often small and treatable before it spreads.  Many tumors don’t cause symptoms in the beginning, so screening can catch cancer before you feel a lump.  Impact of Early Detection:  Stage 1 breast cancer: 99% survival rate.  Stage 4 breast cancer: Survival drops to about 30%.  2. Improves Treatment Outcomes & Options  Smaller tumors may be treated with less aggressive therapy (e.g., lumpectomy instead of mastectomy).  Less need for chemotherapy if cancer is caught early.  Higher chance of breast-conserving surgery (avoiding full removal of the breast).  3. Reduces the Risk of Death from Breast Cancer  Regular mammograms lower breast cancer mortality by 30-40% in women over 50.  Detecting cancer early allows for less invasive treatments and a higher chance of cure.  4. Helps Identify High-Risk Individuals  Screening can detect precancerous changes (like ductal carcinoma in situ – DCIS) before they become invasive.  Women with dense breast tissue (which increases cancer risk) may need additional screening.  High-risk individuals (e.g., BRCA1/2 carriers) can take preventive measures earlier.  5. Supports Better Health Monitoring  Routine screenings track changes over time, making it easier to detect abnormalities.  If a benign lump is found, doctors can monitor it without unnecessary surgery.  Types of Breast Cancer Screening Tests  ✅ Mammogram – Gold standard; detects tumors before they are felt.  ✅ Breast MRI – For high-risk women (e.g., genetic mutations, strong family history).  ✅ Ultrasound – Used when mammograms are unclear (especially for dense breasts).  ✅ Clinical Breast Exam (CBE) – Performed by a doctor, recommended with mammograms.  ✅ Breast Self-Exam (BSE) – Helps you become familiar with your breasts and notice changes.  Who Should Get Screened?  Risk Level Screening Recommendations  Average Risk Mammogram every 1-2 years starting at age 40-50 (depending on guidelines).  High Risk (BRCA mutation, strong family history, radiation exposure) Annual mammogram + MRI starting at age 25-30.  Dense Breasts Mammogram + Ultrasound/MRI, depending on doctor’s advice.  Any Age with Symptoms Immediate medical evaluation, regardless of screening schedule.  Key Takeaways  ✅ Early detection = better survival rates  ✅ Less aggressive treatment needed when caught early  ✅ Reduces breast cancer mortality  ✅ Especially important for women at high risk  Would you like help determining your personal risk level or screening options near you? 😊 |
|  | Repeat | Why Is Breast Cancer Screening Important?  Breast cancer screening is crucial because it helps detect cancer early, often before symptoms appear, leading to better treatment outcomes and higher survival rates. Early detection can prevent cancer from spreading and reduce the need for aggressive treatments.  1. Detects Cancer Early  ✅ Before Symptoms Appear – Many breast cancers show no symptoms in early stages.  ✅ Easier to Treat – Early-stage breast cancer is more likely to respond to treatment with less aggressive options.  ✅ Higher Survival Rates – The 5-year survival rate is 99% for localized breast cancer (found early).  2. Improves Treatment Options & Reduces Mortality  Less invasive treatments (lumpectomy instead of mastectomy).  Less need for chemotherapy if detected early.  Reduces breast cancer death rates by up to 40% in screened populations.  3. Helps Identify High-Risk Individuals  Detects precancerous changes (like atypical hyperplasia or DCIS).  Identifies women with dense breast tissue, who may need additional screening (like MRI or ultrasound).  Genetic screening can help guide BRCA-positive women toward preventive steps.  4. Screening Guidelines by Age  Women at Average Risk  ✅ Ages 40-49: Discuss with doctor; some recommend starting mammograms at 40.  ✅ Ages 50-74: Mammogram every 1-2 years (gold standard).  ✅ 75+: Continue if in good health and life expectancy is 10+ years.  High-Risk Women (e.g., BRCA mutation, strong family history)  ✅ Start screening earlier (age 25-30) with mammograms & MRIs.  ✅ May need genetic testing and closer monitoring.  5. Types of Breast Cancer Screening  📌 Mammogram – The best screening tool, detects tumors before they can be felt.  📌 Breast MRI – Used for high-risk women (e.g., BRCA carriers).  📌 Ultrasound – Used when mammograms are unclear, especially in dense breasts.  📌 Breast Self-Exam & Clinical Exam – Can help detect changes, but not a substitute for mammograms.  Key Takeaways  ✔ Early detection = Better survival + Less invasive treatment.  ✔ Regular mammograms save lives by reducing mortality.  ✔ High-risk women need earlier & more frequent screenings.  Would you like help in understanding when to start screening based on your personal risk factors? 😊 |
| 6 | What are the risks of breast cancer screening? | Risks of Breast Cancer Screening  While breast cancer screening saves lives, it does come with some risks and limitations. Understanding these risks can help in making informed decisions about screening.  1. False Positives (Unnecessary Anxiety & Tests)  A false positive occurs when a mammogram detects an abnormality that is not cancer.  Leads to unnecessary follow-up tests (additional mammograms, ultrasounds, or biopsies).  More common in younger women or those with dense breast tissue.  Can cause stress, anxiety, and emotional distress.  How to Reduce False Positives?  Follow screening guidelines based on your risk level.  Ask your doctor about 3D mammography (tomosynthesis), which reduces false positives.  2. False Negatives (Missed Cancer Cases)  A false negative happens when a mammogram fails to detect cancer, leading to delayed diagnosis.  Occurs in about 10-20% of cases, especially in dense breasts.  May give a false sense of security, causing some women to ignore symptoms.  How to Reduce False Negatives?  Women with dense breasts should consider breast ultrasound or MRI in addition to mammograms.  Always report new symptoms (lumps, skin changes) even if your last mammogram was normal.  3. Overdiagnosis (Detecting Non-Life-Threatening Cancers)  Some detected cancers (like ductal carcinoma in situ – DCIS) may never spread or cause harm.  Leads to unnecessary treatments (surgery, radiation, hormone therapy) for cancers that might not have become dangerous.  It is impossible to predict which cancers will progress, so most detected cancers are treated aggressively.  How to Reduce Overdiagnosis?  Discuss the risks and benefits of treatment for non-invasive cancers (DCIS, LCIS).  Get a second opinion before deciding on aggressive treatment.  4. Radiation Exposure from Mammograms  Mammograms use low-dose radiation, which slightly increases cancer risk over time.  The risk is very small compared to the benefits of early detection.  Risk is higher for young women (under 30), so screening is usually not recommended unless high-risk.  How to Reduce Radiation Exposure?  Follow the recommended screening schedule – don’t over-screen unnecessarily.  Ask about 3D mammography (digital tomosynthesis), which may require fewer follow-ups.  5. Unnecessary Biopsies & Procedures  If an abnormality is found, a biopsy may be needed to confirm if it's cancer.  Many biopsies (about 80%) turn out to be benign (not cancer).  Can lead to scarring, pain, and emotional distress.  How to Reduce Unnecessary Biopsies?  Consider MRI or ultrasound before jumping to a biopsy.  Get a second opinion if unsure about biopsy results.  Should You Still Get Screened?  ✅ Yes! The benefits outweigh the risks for most women, especially as they age.  ✅ Screening guidelines should be personalized based on your risk level.  ✅ Always discuss concerns with your doctor to make informed decisions.  Would you like help understanding your personal screening schedule based on your risk factors? 😊 |
|  | Repeat | Risks of Breast Cancer Screening  While breast cancer screening, especially mammograms, is highly effective in detecting cancer early, it does come with some risks and limitations. Understanding these risks can help individuals make informed decisions about screening.  1. False Positives (Overdiagnosis)  📌 What it means: A test result suggests cancer when there is none.  📌 Impact:  Leads to unnecessary stress, anxiety, and additional tests (biopsies, ultrasounds).  More common in younger women or those with dense breast tissue.  📌 How to Reduce It?  Follow recommended screening intervals (to avoid excessive testing).  Use 3D mammography (tomosynthesis) for more accurate results.  2. False Negatives (Missed Cancer)  📌 What it means: Cancer is present but not detected by the mammogram.  📌 Impact:  Can delay diagnosis and treatment.  Happens in about 1 in 5 cases, especially in women with dense breasts.  📌 How to Reduce It?  Women with dense breasts may need breast ultrasound or MRI in addition to a mammogram.  3. Overdiagnosis & Overtreatment  📌 What it means: Detecting small, slow-growing cancers that may never cause harm in a person’s lifetime.  📌 Impact:  Unnecessary treatments (surgery, radiation, chemotherapy).  Some cases of ductal carcinoma in situ (DCIS) may never develop into invasive cancer.  📌 How to Reduce It?  Discuss watchful waiting vs. immediate treatment with a doctor if diagnosed with DCIS or other early-stage conditions.  4. Exposure to Radiation  📌 What it means: Mammograms use low-dose X-rays, which expose the breast to radiation.  📌 Impact:  The radiation risk is very low, but frequent mammograms over decades could slightly increase cancer risk.  📌 How to Reduce It?  Only get mammograms as recommended (not too frequently).  High-risk individuals can consider MRI screening instead of X-rays.  5. Pain & Discomfort  📌 What it means: Breast compression during a mammogram can be uncomfortable.  📌 Impact:  Some women experience mild to moderate pain during the test.  📌 How to Reduce It?  Schedule the test after your period (breasts are less tender).  Take a mild pain reliever before the exam if needed.  6. Psychological Impact  📌 What it means: Waiting for results or dealing with abnormal findings can cause anxiety.  📌 Impact:  Emotional stress can be high, especially with false positives.  📌 How to Reduce It?  Understand that most abnormal results are not cancer (only 10% of mammogram callbacks lead to cancer diagnosis).  Key Takeaways  ✔ Screening saves lives, but it has risks like false positives and overdiagnosis.  ✔ Women with dense breasts may need additional tests (MRI, ultrasound).  ✔ Radiation risk is very low, but screenings should be done only as recommended.  ✔ Discuss benefits vs. risks with a doctor based on age, family history, and risk factors.  Would you like help in determining which screening method is best for your risk level? 😊 |
| 7 | What are the common screening methods for breast cancer? | Common Breast Cancer Screening Methods  Breast cancer screening aims to detect cancer early, before symptoms appear, when treatment is most effective. Here are the most commonly used screening methods:  1. Mammography (Gold Standard)  🔹 What is it?  A low-dose X-ray that detects abnormal growths in the breast.  Can identify tumors before they can be felt.  🔹 Who should get it?  Women aged 40-49: Discuss with a doctor (screening may start earlier if high risk).  Women aged 50-74: Mammograms every 1-2 years (depending on risk factors).  🔹 Pros  ✅ Proven to reduce breast cancer mortality  ✅ Effective for detecting early-stage cancer  ✅ Widely available & relatively quick  🔹 Cons  ❌ Can miss some cancers (false negatives, especially in dense breasts)  ❌ May detect harmless tumors (overdiagnosis)  ❌ Can lead to false positives, requiring extra tests (biopsies, ultrasounds)  🔹 Types of Mammograms  ✔ 2D Mammogram – Standard X-ray imaging of the breast.  ✔ 3D Mammogram (Tomosynthesis) – Provides clearer, layered images, reducing false positives.  2. Breast Ultrasound  🔹 What is it?  Uses sound waves to create images of breast tissue.  Helps distinguish solid tumors (potential cancer) from fluid-filled cysts (non-cancerous).  🔹 Who should get it?  Women with dense breast tissue (mammograms may not be as clear).  Used as a follow-up test after an abnormal mammogram.  🔹 Pros  ✅ No radiation exposure  ✅ Useful for women with dense breasts  ✅ Good for distinguishing between benign & cancerous lumps  🔹 Cons  ❌ Not a standalone screening test (usually a follow-up test).  ❌ Can’t detect microcalcifications (early cancer signs seen on mammograms).  3. Breast MRI (Magnetic Resonance Imaging)  🔹 What is it?  Uses powerful magnets & contrast dye to create detailed images.  Detects cancer even in dense breast tissue.  🔹 Who should get it?  Women at high risk (BRCA1/2 mutations, strong family history).  Used alongside mammograms for high-risk individuals.  🔹 Pros  ✅ Highly sensitive – detects even small tumors  ✅ Best for women with very dense breasts or genetic risk  🔹 Cons  ❌ Expensive & not always covered by insurance  ❌ More false positives (leading to unnecessary biopsies)  ❌ Requires contrast dye, which some may react to  4. Clinical Breast Exam (CBE)  🔹 What is it?  A doctor or trained healthcare provider examines the breasts and underarms for lumps or abnormalities.  🔹 Who should get it?  Women in their 20s and 30s: Recommended every 1-3 years.  Women over 40: Often part of an annual check-up.  🔹 Pros  ✅ No radiation, non-invasive  ✅ Can detect lumps that a mammogram might miss  🔹 Cons  ❌ Not highly reliable (can miss deep or small tumors)  ❌ Should always be combined with imaging tests  5. Breast Self-Exam (BSE)  🔹 What is it?  A woman examines her own breasts to detect changes (lumps, swelling, nipple discharge).  Not a replacement for medical screenings but helps with breast awareness.  🔹 Who should do it?  All women should be familiar with how their breasts normally look & feel.  🔹 Pros  ✅ No cost, easy to do at home  ✅ Helps you recognize changes early  🔹 Cons  ❌ Not a proven screening tool (many lumps are non-cancerous).  ❌ Can lead to unnecessary anxiety and false alarms.  Which Screening Test is Right for You?  Risk Level Recommended Screening  Average risk (no family history, no genetic mutations) Mammogram every 1-2 years (age 40+), CBE every 1-3 years  High risk (BRCA mutation, family history, dense breasts) Annual mammogram + MRI starting at age 25-30  Women under 40 Breast self-awareness, CBE if needed  Any age with symptoms Immediate medical evaluation  Key Takeaways  ✅ Mammograms are the gold standard for early detection.  ✅ Ultrasound & MRI are useful for high-risk women and dense breasts.  ✅ Clinical exams & self-exams help with breast awareness.  ✅ Early detection = better survival & less aggressive treatment.  Would you like help determining your personal screening plan or finding a screening center near you? 😊 |
|  | Repeat | Common Screening Methods for Breast Cancer  Breast cancer screening involves different methods to detect cancer early, often before symptoms appear. The right screening test depends on age, breast density, and individual risk factors.  1. Mammogram (Gold Standard)  ✅ Best for: Women 40+ or earlier if high-risk.  ✅ How it works:  Uses low-dose X-rays to detect tumors or abnormalities.  3D mammography (Tomosynthesis) provides a clearer image, reducing false positives.  ✅ Pros:  Detects cancer before lumps are felt.  Reduces breast cancer mortality by up to 40%.  ✅ Cons:  False positives (especially in younger women or dense breasts).  Radiation exposure, though minimal.  📌 Recommended:  Ages 40-49: Discuss with doctor (some start at 40, others at 45-50).  Ages 50-74: Mammogram every 1-2 years.  High-risk women: Start screening earlier (age 25-30) with MRI + mammogram.  2. Breast MRI (Magnetic Resonance Imaging)  ✅ Best for:  Women at high risk (BRCA mutations, strong family history).  Dense breast tissue where mammograms may miss cancer.  ✅ How it works:  Uses magnetic fields & contrast dye for a highly detailed image.  ✅ Pros:  More sensitive than a mammogram.  No radiation exposure.  ✅ Cons:  More false positives, leading to unnecessary biopsies.  Expensive & not recommended for average-risk women.  📌 Recommended:  High-risk women: MRI + mammogram yearly starting at age 25-30.  3. Breast Ultrasound  ✅ Best for:  Women with dense breasts or inconclusive mammograms.  Pregnant women (safe alternative to X-rays).  ✅ How it works:  Uses sound waves to create breast images.  ✅ Pros:  No radiation exposure.  Good for detecting lumps missed on mammograms.  ✅ Cons:  Cannot detect microcalcifications (an early cancer sign).  More false positives.  📌 Recommended:  As a follow-up test for abnormal mammograms.  Supplementary screening for dense breast tissue.  4. Clinical Breast Exam (CBE)  ✅ Best for: Women 20+ as part of a routine check-up.  ✅ How it works:  A doctor manually checks for lumps or other changes.  ✅ Pros:  Can help detect lumps.  ✅ Cons:  Less effective than imaging tests.  📌 Recommended:  Every 1-3 years for women in their 20s & 30s.  Annually for women 40+.  5. Breast Self-Exam (BSE)  ✅ Best for: Women who want to stay familiar with their breasts.  ✅ How it works:  Women check their own breasts for lumps, changes, or discharge.  ✅ Pros:  Helps women detect changes between screenings.  ✅ Cons:  Not a reliable screening tool alone.  📌 Recommended:  Not a substitute for mammograms.  Women should still be aware of any changes and report them.  Which Screening Method Should You Choose?  Screening Method Best For Frequency Radiation?  Mammogram (2D/3D) Women 40+ (Gold Standard) Every 1-2 years (age 40+) Yes (low dose)  Breast MRI High-risk women (BRCA, dense breasts) Annually (with mammogram) No  Ultrasound Dense breasts, pregnancy, unclear mammogram As needed No  Clinical Breast Exam Routine check-ups (20+) Every 1-3 years (20-39), annually (40+) No  Self-Exam All women (awareness) Monthly No  Key Takeaways  ✔ Mammograms are the best screening tool for most women.  ✔ MRI & ultrasound help in high-risk cases or dense breasts.  ✔ Self-exams & clinical exams are useful but not substitutes for imaging.  ✔ High-risk women need earlier, more frequent screenings.  Would you like help in determining which screening schedule fits your personal risk factors? 😊 |
| 8 | What is the survival rate of breast cancer? | Survival Rate of Breast Cancer  Breast cancer survival rates depend on factors like cancer stage, type, treatment, and individual health conditions. The survival rate is typically measured as the 5-year relative survival rate, which compares breast cancer patients to people without cancer.  1. Overall Breast Cancer Survival Rates  5-year survival rate: 90%  10-year survival rate: 84%  15-year survival rate: 80%  🔹 These numbers mean that, on average, 90% of women diagnosed with breast cancer are still alive 5 years after diagnosis.  2. Survival Rate by Cancer Stage  Stage 5-Year Survival Rate  Stage 0 (Non-invasive/DCIS) 99-100%  Stage I (Early-stage, localized tumor) 99%  Stage II (Larger tumor, may involve lymph nodes) 86-98%  Stage III (Locally advanced, spread to nearby lymph nodes) 66-72%  Stage IV (Metastatic, spread to distant organs) 30%  🔹 Key Takeaway: Early detection (Stage 0/I) has a nearly 100% survival rate, while advanced-stage cancer survival drops significantly.  3. Factors That Affect Survival Rates  ✔️ Tumor Type & Characteristics  Hormone receptor-positive (ER+/PR+) tumors respond better to treatment.  HER2-positive tumors have targeted therapies that improve survival.  Triple-negative breast cancer (TNBC) is more aggressive but responds well to chemotherapy.  ✔️ Age & Overall Health  Younger women often have more aggressive breast cancer but may respond better to treatment.  Older women may have other health conditions that impact survival.  ✔️ Response to Treatment  Surgery (mastectomy/lumpectomy), chemotherapy, radiation, and targeted therapy all improve outcomes.  Hormonal therapy (Tamoxifen, Aromatase inhibitors) improves survival for ER+/PR+ cancers.  ✔️ Access to Care & Screening  Regular screenings (mammograms) help detect cancer early, improving survival.  Genetic testing & preventive measures (for BRCA1/BRCA2 carriers) lower mortality.  4. Survival Rate for Male Breast Cancer  Male breast cancer is rare but has a 5-year survival rate of ~84%.  Prognosis depends on early detection and treatment.  🔹 Key Takeaways  ✅ Early-stage breast cancer has a 99% survival rate.  ✅ Stage IV (metastatic) breast cancer has a lower survival rate (30%), but treatment is improving outcomes.  ✅ Regular screening, early detection, and advanced treatments improve survival.  Would you like to know about treatment options or new research on survival improvement? 😊 |
|  | Repeat | Survival Rate of Breast Cancer  Breast cancer survival rates depend on stage at diagnosis, tumor type, and treatment effectiveness. The earlier it is detected, the higher the survival rate.  1. Overall 5-Year Survival Rates  Localized (Stage 0 & 1, confined to the breast) → 99%  Regional (Stage 2 & 3, spread to nearby lymph nodes) → 86%  Distant (Stage 4, metastatic, spread to other organs) → 30%  Overall (all stages combined) → 90%  📌 This means that, on average, 90% of women diagnosed with breast cancer will live at least 5 years after diagnosis.  2. Survival Rates by Breast Cancer Stage  Stage Extent of Spread 5-Year Survival Rate  Stage 0 Non-invasive (DCIS) ~100%  Stage 1 Small tumor, no lymph node spread 99%  Stage 2 Tumor larger, limited lymph node involvement 90-95%  Stage 3 Cancer spread to multiple lymph nodes 70-86%  Stage 4 Metastatic (spread to lungs, liver, bones) 30%  🔹 Early detection significantly improves survival chances.  🔹 Metastatic breast cancer (Stage 4) has lower survival, but new treatments are improving outcomes.  3. Factors That Affect Survival Rates  ✅ Tumor Type & Hormone Receptor Status  Hormone-positive (ER+/PR+) cancers → Better response to treatment, higher survival.  HER2-positive cancers → More aggressive but improved with targeted therapy (e.g., Herceptin).  Triple-negative breast cancer (TNBC) → More aggressive, lower survival (77% for localized TNBC).  ✅ Age & Overall Health  Younger women (under 40) tend to have more aggressive cancers.  Older women may have other health conditions affecting survival.  ✅ Response to Treatment  Surgery + chemotherapy + radiation + targeted therapy improves outcomes.  Immunotherapy & newer treatments are increasing survival rates for aggressive cancers.  4. Long-Term Survival (10-Year & 15-Year Rates)  10-year survival: ~84%  15-year survival: ~80%  Metastatic breast cancer (Stage 4) can be managed as a chronic disease in some cases.  📌 Survivors require regular follow-ups to monitor for recurrence or long-term effects of treatment.  5. How to Improve Survival Chances?  ✅ Early detection (mammograms, self-exams, doctor visits).  ✅ Following treatment plans (surgery, chemo, hormone therapy, radiation).  ✅ Healthy lifestyle (diet, exercise, stress management).  ✅ Genetic testing for high-risk individuals (BRCA mutations).  Key Takeaways  ✔ Early-stage breast cancer (Stage 0-1) has a near 100% survival rate.  ✔ Stage 4 (metastatic) breast cancer has a lower survival rate (30%), but new treatments are improving outcomes.  ✔ Hormone receptor-positive cancers have better survival rates than triple-negative breast cancer.  ✔ Early detection, personalized treatment, and lifestyle changes can significantly impact survival.  Would you like help understanding treatment options based on breast cancer type? 😊 |
| 9 | What are the treatment methods of breast cancer? | Treatment Methods for Breast Cancer  Breast cancer treatment depends on factors such as cancer stage, tumor type, hormone receptor status, and overall health. Treatment often involves a combination of therapies to remove, shrink, or prevent the spread of cancer.  1. Surgery (Primary Treatment for Early-Stage Cancer)  ✔️ Lumpectomy (Breast-Conserving Surgery)  Removes the tumor and a small margin of surrounding tissue while preserving most of the breast.  Often followed by radiation therapy to prevent recurrence.  Best for small, localized tumors.  ✔️ Mastectomy  Removes the entire breast to eliminate cancer.  Types:  Simple/Total Mastectomy – Removes the whole breast but not lymph nodes.  Modified Radical Mastectomy – Removes breast and some lymph nodes.  Radical Mastectomy – Removes breast, lymph nodes, and chest muscles (rarely used today).  May be followed by breast reconstruction surgery.  ✔️ Lymph Node Removal (Sentinel or Axillary Node Biopsy)  If cancer has spread to lymph nodes, they may need to be removed to prevent further spread.  2. Radiation Therapy (Destroys Remaining Cancer Cells)  🔹 Uses high-energy X-rays to kill cancer cells left after surgery.  ✔️ External Beam Radiation  The most common type; directs radiation beams at the breast area.  ✔️ Internal Radiation (Brachytherapy)  Radioactive implants are placed inside the breast near the tumor site.  🔹 Best for:  ✅ After lumpectomy (to reduce recurrence risk).  ✅ Locally advanced breast cancer.  🔹 Side Effects:  ❌ Fatigue, skin irritation, and swelling.  3. Chemotherapy (Kills Fast-Growing Cancer Cells)  🔹 Powerful drugs kill cancer cells throughout the body.  ✔️ Neoadjuvant Chemotherapy (Before Surgery)  Shrinks tumors, making surgery easier.  ✔️ Adjuvant Chemotherapy (After Surgery)  Kills remaining cancer cells to prevent recurrence.  ✔️ Chemotherapy for Metastatic Breast Cancer  Helps control the spread but not a cure for Stage IV cancer.  🔹 Common Drugs:  Doxorubicin (Adriamycin), Paclitaxel (Taxol), Cyclophosphamide (Cytoxan).  🔹 Side Effects:  ❌ Hair loss, nausea, fatigue, low immunity.  4. Hormone Therapy (For Hormone-Sensitive Cancers)  🔹 Blocks estrogen or progesterone, which fuels some breast cancers (ER+/PR+ tumors).  ✔️ Selective Estrogen Receptor Modulators (SERMs)  Tamoxifen – Blocks estrogen in premenopausal women.  ✔️ Aromatase Inhibitors (AIs)  Anastrozole, Letrozole, Exemestane – Reduce estrogen in postmenopausal women.  ✔️ Ovarian Suppression (For Premenopausal Women)  Medications (e.g., Lupron, Zoladex) or ovary removal to stop estrogen production.  🔹 Best for:  ✅ Hormone receptor-positive breast cancer (ER+/PR+).  🔹 Side Effects:  ❌ Menopause symptoms (hot flashes, bone loss, mood changes).  5. Targeted Therapy (For HER2-Positive Breast Cancer)  🔹 Specifically attacks HER2-positive cancer cells without harming normal cells.  ✔️ Monoclonal Antibodies  Trastuzumab (Herceptin), Pertuzumab (Perjeta) – Block HER2 protein to stop tumor growth.  ✔️ HER2-Targeted Tyrosine Kinase Inhibitors  Lapatinib, Neratinib – Used in advanced cases.  🔹 Best for:  ✅ HER2-positive breast cancer (about 20% of cases).  🔹 Side Effects:  ❌ Heart problems, fatigue, diarrhea.  6. Immunotherapy (Boosts the Immune System to Fight Cancer)  🔹 Helps the immune system recognize & destroy cancer cells.  ✔️ Checkpoint Inhibitors  Atezolizumab (Tecentriq), Pembrolizumab (Keytruda) – Used in triple-negative breast cancer (TNBC).  🔹 Best for:  ✅ Triple-negative breast cancer (TNBC).  🔹 Side Effects:  ❌ Autoimmune reactions, fatigue, skin rashes.  7. Bone-Directed Therapy (For Metastatic Cancer)  🔹 Prevents bone damage in cases where breast cancer has spread to bones.  🔹 Common Drugs:  Bisphosphonates (Zoledronic acid, Pamidronate) – Strengthen bones.  Denosumab (Xgeva) – Prevents bone fractures.  🔹 Side Effects:  ❌ Joint pain, flu-like symptoms.  Which Treatment is Right for You?  Stage Recommended Treatments  Stage 0 (DCIS) Surgery (Lumpectomy or Mastectomy) + Radiation (if needed)  Stage I & II Surgery + Radiation + (Chemo/Hormone/Targeted Therapy depending on tumor type)  Stage III Neoadjuvant Chemo → Surgery → Radiation + Targeted Therapy (if HER2+)  Stage IV (Metastatic) Chemo, Hormone Therapy, Targeted Therapy, Immunotherapy, Palliative Care  Key Takeaways  ✅ Surgery, radiation, and chemotherapy are the main treatments.  ✅ Targeted therapy & hormone therapy improve outcomes for specific types of cancer.  ✅ Immunotherapy is a breakthrough for triple-negative breast cancer.  ✅ Early detection leads to less aggressive treatment and better survival.  Would you like more details on personalized treatment options or clinical trials? 😊 |
|  | Repeat | Breast Cancer Treatment Methods  Breast cancer treatment depends on factors like cancer type, stage, hormone receptor status (ER/PR), HER2 status, and overall health. The main treatment options include:  1. Surgery  Surgery is often the first step in treating breast cancer, aimed at removing the tumor.  Types of Surgery:  Lumpectomy (Breast-Conserving Surgery): Removes only the tumor and some surrounding tissue.  Mastectomy: Removes the entire breast.  Simple/Total Mastectomy: Whole breast removal.  Radical Mastectomy: Entire breast, chest muscles, and lymph nodes removed (rarely done now).  Modified Radical Mastectomy: Breast and lymph nodes removed but chest muscles preserved.  Sentinel Lymph Node Biopsy (SLNB): Removes the first lymph node(s) to check for cancer spread.  Axillary Lymph Node Dissection (ALND): More extensive removal of lymph nodes if cancer has spread.  2. Radiation Therapy  Uses high-energy X-rays to kill cancer cells, often after surgery to reduce recurrence.  Types of Radiation:  External Beam Radiation Therapy (EBRT): Most common; targets the breast area from outside the body.  Brachytherapy (Internal Radiation): Radioactive material is placed inside the breast near the tumor site.  3. Chemotherapy  Uses drugs to kill cancer cells, often used before (neoadjuvant) or after (adjuvant) surgery.  Common Chemotherapy Drugs for Breast Cancer:  Anthracyclines (e.g., Doxorubicin, Epirubicin)  Taxanes (e.g., Paclitaxel, Docetaxel)  Cyclophosphamide  Fluorouracil (5-FU)  Platinum-based drugs (e.g., Carboplatin, Cisplatin)  Targeted Chemotherapy (for triple-negative breast cancer, like Sacituzumab govitecan)  4. Hormone (Endocrine) Therapy  For hormone receptor-positive (ER+/PR+) breast cancers, hormone therapy blocks estrogen or progesterone.  Types of Hormone Therapy:  Selective Estrogen Receptor Modulators (SERMs)  Example: Tamoxifen (blocks estrogen in breast tissue)  Aromatase Inhibitors (AIs) (for postmenopausal women)  Examples: Anastrozole, Letrozole, Exemestane (reduce estrogen production)  Ovarian Suppression (for premenopausal women)  LHRH Agonists (e.g., Leuprolide, Goserelin) suppress estrogen by shutting down ovaries.  Oophorectomy (surgical removal of ovaries) is a permanent option.  5. Targeted Therapy  For cancers with specific molecular markers like HER2-positive or BRCA mutations.  Common Targeted Therapies:  HER2-Positive Breast Cancer:  Trastuzumab (Herceptin)  Pertuzumab (Perjeta)  Ado-Trastuzumab Emtansine (Kadcyla)  Lapatinib (Tykerb)  BRCA-Mutated Breast Cancer:  PARP Inhibitors (e.g., Olaparib, Talazoparib)  PI3K Inhibitors (for PIK3CA mutations):  Alpelisib (Piqray)  6. Immunotherapy  Uses the immune system to fight cancer, especially for triple-negative breast cancer (TNBC).  FDA-Approved Immunotherapy:  Atezolizumab (Tecentriq)  Pembrolizumab (Keytruda)  These are often combined with chemotherapy for advanced-stage or metastatic TNBC.  7. Bone-Directed Therapy  For metastatic breast cancer that has spread to bones, bone-strengthening drugs reduce complications.  Bisphosphonates (e.g., Zoledronic acid, Pamidronate)  Denosumab (Xgeva) (a monoclonal antibody)  8. Clinical Trials & Experimental Therapies  Ongoing research explores new drugs, personalized medicine, CAR-T cell therapy, and vaccine-based treatments. If standard treatments don’t work, clinical trials may offer novel options.  Personalized Treatment Plan  Treatment is usually multimodal, meaning a combination of methods is used.  For example:  Early-stage breast cancer: Surgery → Radiation → Hormone or Chemotherapy (if needed)  HER2-positive breast cancer: Surgery → Chemotherapy + HER2-targeted therapy  Triple-negative breast cancer: Chemotherapy + Immunotherapy → Surgery  Metastatic breast cancer: Targeted therapy, immunotherapy, or palliative care  Would you like help understanding which treatment approach is most effective for specific breast cancer subtypes? |
| 10 | Can breast cancer recur? | Can Breast Cancer Recur?  Yes, breast cancer can recur even after successful treatment. Recurrence happens when some cancer cells survive treatment and grow back later. The risk depends on factors like cancer stage, tumor type, treatment effectiveness, and individual biology.  1. Types of Breast Cancer Recurrence  ✔️ Local Recurrence (Same Breast or Chest Wall)  Cancer comes back in the same area where it was originally found.  Symptoms may include:  A new lump or thickening in the breast or chest.  Skin changes (redness, swelling, or dimpling).  Nipple changes or discharge.  Treatment: Surgery, radiation, or systemic therapy (chemo, hormone therapy, targeted therapy).  ✔️ Regional Recurrence (Nearby Lymph Nodes)  Cancer spreads to nearby lymph nodes (armpit, collarbone, or neck).  Symptoms may include:  Swelling or lumps in the lymph nodes.  Pain or tightness in the armpit or chest area.  Treatment: Surgery, radiation, chemotherapy, or targeted therapy.  ✔️ Distant Recurrence (Metastatic Breast Cancer – Stage IV)  Cancer spreads to other organs (bones, lungs, liver, brain).  Symptoms depend on where cancer spreads:  Bones: Bone pain, fractures.  Lungs: Persistent cough, shortness of breath.  Liver: Jaundice, abdominal pain.  Brain: Headaches, seizures, confusion.  Treatment: Chemotherapy, targeted therapy, immunotherapy, palliative care.  2. What Increases the Risk of Recurrence?  ✔️ Tumor Characteristics  Larger tumors or lymph node involvement = Higher recurrence risk.  Triple-negative breast cancer (TNBC) has a higher risk of early recurrence.  HER2-positive tumors can be aggressive but respond well to targeted therapy.  ✔️ Stage at Diagnosis  Stage 0 & I: Low recurrence risk.  Stage II & III: Higher risk, especially if lymph nodes were involved.  ✔️ Incomplete Treatment  Skipping radiation, chemo, or hormone therapy increases recurrence risk.  Stopping hormone therapy (Tamoxifen, Aromatase Inhibitors) too early raises risk for ER+/PR+ cancers.  ✔️ Lifestyle Factors  Obesity, smoking, alcohol, and lack of exercise increase recurrence risk.  Chronic stress and poor diet may also play a role.  3. How to Reduce Recurrence Risk?  ✔️ Follow the Full Treatment Plan  Complete all prescribed treatments (radiation, chemo, hormone therapy).  Continue hormone therapy for 5-10 years if ER+/PR+ (as recommended).  ✔️ Maintain a Healthy Lifestyle  Exercise regularly (150 min/week).  Eat a balanced diet (rich in fruits, veggies, lean proteins).  Limit alcohol & avoid smoking.  Maintain a healthy weight (especially after menopause).  ✔️ Regular Follow-Ups & Screening  Mammograms, blood tests, and imaging help detect recurrence early.  Self-exams and awareness of symptoms are essential.  4. What Are the Chances of Recurrence?  Cancer Type/Stage Recurrence Rate (Approx.)  Stage 0 (DCIS) <10% (with treatment)  Stage I 5-10%  Stage II 15-30%  Stage III 30-50%  Triple-Negative Breast Cancer (TNBC) Up to 40% (within 3 years)  HER2-Positive Breast Cancer Higher, but reduced with targeted therapy  🔹 Recurrence risk is highest in the first 2-5 years after treatment.  Key Takeaways  ✅ Breast cancer can recur, but early detection and proper treatment reduce the risk.  ✅ Local recurrence is treatable; metastatic recurrence is managed with long-term therapy.  ✅ Following a full treatment plan and adopting a healthy lifestyle lowers recurrence risk.  ✅ Regular follow-ups and screenings are critical for early detection.  Would you like help understanding your recurrence risk or prevention strategies? 😊 |
|  | Repeat | Can Breast Cancer Recur?  Yes, breast cancer can recur, even after successful treatment. Recurrence happens when cancer cells that survived initial treatment start to grow again, sometimes months, years, or even decades later.  The risk of recurrence depends on several factors, including:  Cancer type and stage at diagnosis  Tumor characteristics (e.g., hormone receptor status, HER2 status)  Effectiveness of initial treatment  Lymph node involvement  Lifestyle and genetic factors  Types of Breast Cancer Recurrence  Breast cancer recurrence is classified into three main types:  1. Local Recurrence (Same Breast or Chest Wall)  Cancer returns in the same breast, mastectomy scar, or chest wall.  Symptoms:  A new lump or thickening in the breast or chest area.  Changes in breast skin (redness, swelling, dimpling).  Nipple discharge or retraction (in cases of breast-conserving surgery).  Pain or irritation in the same area.  2. Regional Recurrence (Nearby Lymph Nodes)  Cancer reappears in the lymph nodes near the original tumor site, such as under the arm (axillary), near the collarbone (supraclavicular), or near the breastbone (internal mammary nodes).  Symptoms:  Swelling or lumps in the armpit, collarbone, or neck area.  Persistent pain or swelling in the chest.  3. Distant (Metastatic) Recurrence (Stage 4)  Cancer spreads to other organs, such as the bones, liver, lungs, or brain.  Symptoms depend on the affected organ:  Bone metastasis: Bone pain, fractures.  Lung metastasis: Chronic cough, shortness of breath.  Liver metastasis: Jaundice, swelling in the abdomen.  Brain metastasis: Headaches, vision problems, seizures.  Risk of Breast Cancer Recurrence  The risk of recurrence depends on several factors:  Stage at Diagnosis: Higher stages (Stage 2-3) have a greater risk.  Lymph Node Involvement: Cancer in multiple lymph nodes increases recurrence chances.  Hormone Receptor Status:  ER+/PR+ (Hormone Receptor-Positive) cancers can recur even after many years.  HER2+ (HER2-Positive) cancers have a higher recurrence rate but respond well to targeted therapy.  Triple-Negative Breast Cancer (TNBC) has a higher early recurrence risk (within the first 3 years).  Treatment Completion: Incomplete treatment increases recurrence risk.  How to Reduce the Risk of Recurrence  While recurrence cannot always be prevented, the following strategies help lower the risk:  ✅ Follow-Up Care: Regular check-ups, blood tests, and imaging (e.g., mammograms, MRIs).  ✅ Hormone Therapy (if ER+/PR+): Taking Tamoxifen or Aromatase Inhibitors as prescribed.  ✅ Targeted Therapy (if HER2+): Using Trastuzumab (Herceptin) and other HER2 inhibitors.  ✅ Lifestyle Changes:  Healthy diet (fruits, vegetables, lean proteins).  Regular exercise (150 minutes per week).  Maintain a healthy weight.  Limit alcohol consumption. ✅ Medication Adherence: Completing prescribed therapies like chemotherapy, radiation, or immunotherapy.  When to See a Doctor?  If you experience any new lumps, pain, or unusual symptoms, consult a doctor immediately. Early detection of recurrence improves treatment outcomes.  Would you like more information on personalized risk assessment or new treatment options? 🚀 |
| **DeepSeek** | | |
|  | **Question** | **Answer** |
| 1 | What are the common symptoms of breast cancer? | Breast cancer symptoms can vary widely, and some individuals may not experience any symptoms at all, especially in the early stages. However, common signs and symptoms of breast cancer include:  Lump or Thickening: A new lump or mass in the breast or underarm (armpit) is one of the most common signs. The lump may feel hard, irregular in shape, and different from the surrounding tissue.  Changes in Breast Size or Shape: Swelling, shrinkage, or asymmetry of the breast, especially if it occurs on one side only.  Skin Changes: Dimpling, puckering, or redness of the breast skin, sometimes resembling the texture of an orange peel (known as peau d'orange).  Nipple Changes:  Inversion (nipple turning inward).  Nipple discharge that is not breast milk, which may be clear, bloody, or another color.  Pain or tenderness in the nipple area.  Breast Pain: Persistent pain or tenderness in the breast, though this is less common and can also be caused by non-cancerous conditions.  Swelling or Lump in the Armpit: Enlarged lymph nodes under the arm or near the collarbone.  Redness or Scaling: Redness, flaking, or thickening of the skin on the breast or nipple.  Warmth or Itching: Unusual warmth or itching in the breast.  It’s important to note that many of these symptoms can also be caused by benign (non-cancerous) conditions, such as cysts or infections. However, if you notice any persistent or unusual changes in your breasts, it’s essential to consult a healthcare professional for evaluation and diagnosis. Early detection and treatment significantly improve outcomes for breast cancer. Regular self-exams, clinical breast exams, and mammograms (as recommended by your doctor) are key to early detection. |
|  | Repeat | The common symptoms of breast cancer can vary depending on the stage and type of cancer, as well as individual factors. It's important to note that early-stage breast cancer often has no symptoms, which is why regular screening (e.g., mammograms) is crucial for early detection. However, as the cancer progresses, the following symptoms may occur:  Common Symptoms of Breast Cancer  Lump or Mass in the Breast or Underarm  A new, painless lump or thickening in the breast or underarm (armpit) is one of the most common signs.  The lump may feel hard, irregular in shape, and different from the surrounding tissue.  Changes in Breast Size or Shape  Swelling, shrinkage, or asymmetry of the breast, especially if it occurs on one side only.  Visible changes in the contour or shape of the breast.  Skin Changes on the Breast  Dimpling, puckering, or redness of the breast skin, sometimes resembling the texture of an orange peel (known as peau d'orange).  Scaly, thickened, or flaky skin on the breast or nipple.  Nipple Changes  Inversion (nipple turning inward).  Nipple discharge that is not breast milk, which may be clear, bloody, or another color.  Crusting, peeling, or flaking around the nipple area.  Breast Pain or Tenderness  Persistent pain or tenderness in the breast or nipple, though this is less common and can also be caused by non-cancerous conditions.  Swelling or Lumps in the Armpit or Collarbone Area  Enlarged lymph nodes under the arm or near the collarbone, which may indicate the spread of cancer.  Redness or Warmth  Unusual redness, warmth, or itching in the breast, which may indicate inflammation or infection.  Important Notes  Many of these symptoms can also be caused by benign (non-cancerous) conditions, such as cysts, infections, or hormonal changes.  If you notice any persistent or unusual changes in your breasts, it’s essential to consult a healthcare professional for evaluation and diagnosis.  Early detection through regular self-exams, clinical breast exams, and mammograms significantly improves outcomes for breast cancer.  If you have concerns or a family history of breast cancer, talk to your doctor about appropriate screening and preventive measures. |
| 2 | What are the risk factors for breast cancer? | Breast cancer symptoms can vary widely, and some individuals may not experience any symptoms at all, especially in the early stages. However, common signs and symptoms of breast cancer include:  Lump or Thickening: A new lump or mass in the breast or underarm (armpit) is one of the most common signs. The lump may feel hard, irregular in shape, and different from the surrounding tissue.  Changes in Breast Size or Shape: Swelling, shrinkage, or asymmetry of the breast, especially if it occurs on one side only.  Skin Changes: Dimpling, puckering, or redness of the breast skin, sometimes resembling the texture of an orange peel (known as peau d'orange).  Nipple Changes:  Inversion (nipple turning inward).  Nipple discharge that is not breast milk, which may be clear, bloody, or another color.  Pain or tenderness in the nipple area.  Breast Pain: Persistent pain or tenderness in the breast, though this is less common and can also be caused by non-cancerous conditions.  Swelling or Lump in the Armpit: Enlarged lymph nodes under the arm or near the collarbone.  Redness or Scaling: Redness, flaking, or thickening of the skin on the breast or nipple.  Warmth or Itching: Unusual warmth or itching in the breast.  It’s important to note that many of these symptoms can also be caused by benign (non-cancerous) conditions, such as cysts or infections. However, if you notice any persistent or unusual changes in your breasts, it’s essential to consult a healthcare professional for evaluation and diagnosis. Early detection and treatment significantly improve outcomes for breast cancer. Regular self-exams, clinical breast exams, and mammograms (as recommended by your doctor) are key to early detection.  What are the risk factors for breast cancer?  Breast cancer risk factors are characteristics or exposures that increase the likelihood of developing the disease. Some risk factors are modifiable (related to lifestyle or environment), while others are non-modifiable (such as genetics or age). Here are the key risk factors for breast cancer:  Non-Modifiable Risk Factors  Gender: Women are at a much higher risk of developing breast cancer than men, though men can also develop breast cancer.  Age: The risk of breast cancer increases with age, with most cases diagnosed in women over 50.  Family History: A close relative (mother, sister, or daughter) with breast cancer increases your risk, especially if they were diagnosed at a young age.  Genetic Mutations: Inherited mutations in genes such as BRCA1 and BRCA2 significantly increase the risk of breast and ovarian cancer.  Personal History of Breast Cancer: If you’ve had breast cancer in one breast, you have a higher risk of developing it in the other breast or a different part of the same breast.  Dense Breast Tissue: Women with denser breast tissue (more glandular and fibrous tissue and less fatty tissue) have a higher risk.  Reproductive History:  Early menstruation (before age 12).  Late menopause (after age 55).  Radiation Exposure: Previous radiation therapy to the chest area, especially during adolescence or young adulthood, increases risk.  Race and Ethnicity: White women are slightly more likely to develop breast cancer than women of other races, though Black women are more likely to develop aggressive forms and die from the disease.  Modifiable Risk Factors  Hormone Replacement Therapy (HRT): Long-term use of combined estrogen and progesterone hormone therapy after menopause increases risk.  Oral Contraceptives: Some studies suggest a slight increase in risk for women who use birth control pills, though the risk decreases after stopping.  Alcohol Consumption: Regular alcohol consumption is linked to an increased risk of breast cancer.  Obesity: Being overweight or obese, especially after menopause, increases risk due to higher estrogen levels produced by fat tissue.  Physical Inactivity: A sedentary lifestyle is associated with a higher risk of breast cancer.  Not Having Children or Having Children Later: Women who have never given birth or had their first child after age 30 may have a slightly higher risk.  Not Breastfeeding: Breastfeeding for 1.5 to 2 years may slightly reduce breast cancer risk.  Smoking: Long-term smoking, especially if started at a young age, is linked to a higher risk of breast cancer.  Other Factors  Diethylstilbestrol (DES) Exposure: Women who took DES (a drug given to prevent miscarriage) during pregnancy or were exposed to it in utero have a slightly higher risk.  Environmental Factors: Exposure to certain chemicals or pollutants may increase risk, though research is ongoing.  Important Notes  Having one or more risk factors does not mean you will develop breast cancer, and many women with breast cancer have no known risk factors.  Regular screenings (mammograms, clinical breast exams, and self-exams) and lifestyle changes (e.g., maintaining a healthy weight, limiting alcohol, and exercising) can help reduce risk and improve early detection.  If you have concerns about your risk factors, consult a healthcare provider for personalized advice and screening recommendations. |
|  | Repeat | Breast cancer risk factors are conditions or behaviors that increase the likelihood of developing the disease. While having one or more risk factors does not guarantee that someone will develop breast cancer, understanding these factors can help individuals take preventive measures and make informed decisions about screening. Here are the common risk factors for breast cancer:  Non-Modifiable Risk Factors (Cannot Be Changed)  Gender  Women are at a much higher risk of developing breast cancer than men, though men can also develop breast cancer.  Age  The risk of breast cancer increases with age, with most cases diagnosed in women over 50.  Family History of Breast Cancer  Having a close relative (e.g., mother, sister, daughter) with breast cancer increases your risk, especially if they were diagnosed at a young age.  Inherited Genetic Mutations  Mutations in certain genes, such as BRCA1 and BRCA2, significantly increase the risk of breast and ovarian cancers.  Personal History of Breast Cancer  If you’ve had breast cancer in one breast, you have a higher risk of developing it in the other breast or a different part of the same breast.  Dense Breast Tissue  Women with dense breast tissue (more glandular and fibrous tissue than fatty tissue) have a higher risk of breast cancer.  Radiation Exposure  Previous radiation therapy to the chest area (e.g., for Hodgkin’s lymphoma) increases the risk of breast cancer.  Reproductive History  Early menstruation (before age 12) or late menopause (after age 55) increases lifetime exposure to hormones, raising breast cancer risk.  Modifiable Risk Factors (Can Be Changed or Managed)  Physical Inactivity  A sedentary lifestyle is associated with a higher risk of breast cancer.  Obesity or Excess Weight  Being overweight or obese, especially after menopause, increases the risk of breast cancer due to higher estrogen levels.  Alcohol Consumption  Regular alcohol consumption is linked to an increased risk of breast cancer. The more alcohol consumed, the higher the risk.  Hormone Replacement Therapy (HRT)  Long-term use of combined estrogen and progesterone hormone therapy during menopause increases breast cancer risk.  Oral Contraceptives  Some studies suggest a slight increase in breast cancer risk for women who use oral contraceptives, though the risk decreases after stopping use.  Not Breastfeeding  Women who do not breastfeed may have a slightly higher risk of breast cancer compared to those who do.  Smoking  Smoking, especially long-term or heavy smoking, is associated with a higher risk of breast cancer.  Unhealthy Diet  A diet high in processed foods, red meat, and saturated fats may contribute to an increased risk.  Other Risk Factors  Race and Ethnicity  White women are slightly more likely to develop breast cancer than women of other races, though Black women are more likely to develop aggressive forms of breast cancer and die from the disease.  Diethylstilbestrol (DES) Exposure  Women who took the drug DES (given to prevent miscarriage in the mid-20th century) or whose mothers took it have a slightly higher risk.  What You Can Do to Reduce Risk  Maintain a healthy weight through diet and exercise.  Limit alcohol consumption.  Avoid smoking.  Breastfeed, if possible.  Discuss the risks and benefits of hormone therapy with your doctor.  Get regular breast cancer screenings (e.g., mammograms) as recommended by your healthcare provider.  If you have a family history or genetic predisposition, consider genetic counseling and testing.  While some risk factors cannot be changed, adopting a healthy lifestyle and staying informed about screening options can help reduce your risk of breast cancer. If you have concerns about your risk factors, consult your healthcare provider for personalized advice. |
| 3 | How to prevent breast cancer? | While there is no guaranteed way to prevent breast cancer, certain lifestyle changes and proactive measures can help reduce your risk. Here are evidence-based strategies for lowering the likelihood of developing breast cancer:  Lifestyle Changes  Maintain a Healthy Weight:  Obesity, especially after menopause, increases breast cancer risk. Aim for a healthy weight through a balanced diet and regular exercise.  Exercise Regularly:  Engage in moderate physical activity (e.g., brisk walking, swimming, or cycling) for at least 150 minutes per week. Regular exercise helps regulate hormones and boosts immunity.  Limit Alcohol Consumption:  Alcohol is a known risk factor for breast cancer. If you drink, limit intake to no more than one drink per day.  Avoid Smoking:  Smoking is linked to many cancers, including breast cancer. If you smoke, seek help to quit.  Breastfeed if Possible:  Breastfeeding for 1.5 to 2 years (cumulatively) may slightly reduce breast cancer risk by reducing estrogen exposure.  Dietary Choices  Eat a Balanced Diet:  Focus on fruits, vegetables, whole grains, and lean proteins. Include foods rich in antioxidants and fiber.  Limit Processed and Red Meat:  High consumption of processed and red meats may increase cancer risk. Opt for plant-based proteins or fish instead.  Choose Healthy Fats:  Replace saturated and trans fats with healthy fats like those found in olive oil, nuts, seeds, and fatty fish.  Limit Sugar and Refined Carbs:  High sugar intake and refined carbohydrates can contribute to obesity and inflammation, which may increase cancer risk.  Medical and Preventive Measures  Regular Screenings:  Follow recommended guidelines for mammograms and clinical breast exams. Early detection can prevent advanced-stage breast cancer.  Know Your Family History:  If you have a strong family history of breast or ovarian cancer, consider genetic counseling and testing for mutations like BRCA1 or BRCA2.  Limit Hormone Therapy:  Avoid long-term use of combined estrogen and progesterone hormone replacement therapy (HRT) after menopause. Discuss alternatives with your doctor.  Consider Preventive Medications:  For women at high risk, medications like tamoxifen or raloxifene may reduce the risk of developing breast cancer. Consult your doctor to see if this is an option for you.  Prophylactic Surgery:  In rare cases, women with a very high genetic risk (e.g., BRCA mutations) may consider preventive mastectomy or removal of the ovaries to reduce risk.  Environmental and Behavioral Factors  Reduce Exposure to Environmental Toxins:  Limit exposure to chemicals that may disrupt hormones, such as certain pesticides, plastics, and industrial pollutants.  Manage Stress:  Chronic stress may weaken the immune system. Practice stress-reducing techniques like yoga, meditation, or mindfulness.  Get Enough Sleep:  Poor sleep and irregular sleep patterns may disrupt hormone levels. Aim for 7-9 hours of quality sleep per night.  Breast Awareness  Perform Regular Self-Exams:  Familiarize yourself with the normal look and feel of your breasts. Report any changes (e.g., lumps, pain, or discharge) to your doctor promptly.  Stay Informed:  Keep up-to-date with the latest breast cancer prevention guidelines and research.  Important Note  While these strategies can help reduce your risk, they cannot eliminate it entirely. Some risk factors, like genetics and age, cannot be changed. Regular check-ups and open communication with your healthcare provider are essential for personalized prevention and early detection.  If you have a family history of breast cancer or other concerns, consult a healthcare professional for tailored advice and screening recommendations. |
|  | Repeat | While there is no guaranteed way to prevent breast cancer, certain lifestyle changes and proactive measures can significantly reduce your risk. Here are evidence-based strategies to help lower your chances of developing breast cancer:  1. Maintain a Healthy Weight  Why: Being overweight or obese, especially after menopause, increases breast cancer risk due to higher estrogen levels.  How: Aim for a balanced diet and regular physical activity to achieve and maintain a healthy weight.  2. Stay Physically Active  Why: Regular exercise helps regulate hormones, boosts the immune system, and reduces inflammation, all of which can lower breast cancer risk.  How: Aim for at least 150 minutes of moderate-intensity exercise (e.g., brisk walking) or 75 minutes of vigorous exercise (e.g., running) per week.  3. Limit Alcohol Consumption  Why: Alcohol increases estrogen levels and damages DNA, raising breast cancer risk.  How: If you drink alcohol, limit it to no more than one drink per day (for women) or avoid it altogether.  4. Avoid Smoking  Why: Smoking is linked to many cancers, including breast cancer, due to the harmful chemicals in tobacco.  How: Quit smoking and avoid secondhand smoke. Seek support from healthcare professionals or smoking cessation programs if needed.  5. Eat a Healthy Diet  Why: A nutrient-rich diet can help maintain a healthy weight and reduce inflammation, lowering cancer risk.  How: Focus on:  Fruits and vegetables (rich in antioxidants).  Whole grains (e.g., oats, brown rice).  Lean proteins (e.g., fish, poultry, beans).  Healthy fats (e.g., olive oil, nuts, avocados).  Limit processed foods, red meat, and sugary drinks.  6. Breastfeed, If Possible  Why: Breastfeeding reduces lifetime exposure to estrogen and may help lower breast cancer risk.  How: Aim to breastfeed for at least several months, if not longer.  7. Limit Hormone Replacement Therapy (HRT)  Why: Long-term use of combined estrogen and progesterone HRT increases breast cancer risk.  How: If you need HRT for menopausal symptoms, discuss the risks and benefits with your doctor and use the lowest effective dose for the shortest time possible.  8. Get Regular Screenings  Why: Early detection through screening can help catch breast cancer at its most treatable stage.  How: Follow your doctor’s recommendations for:  Mammograms (typically starting at age 40–50, depending on risk factors).  Clinical breast exams (performed by a healthcare provider).  Breast self-exams (to become familiar with your breasts and notice any changes).  9. Know Your Family History and Genetic Risk  Why: A family history of breast cancer or inherited genetic mutations (e.g., BRCA1, BRCA2) significantly increases risk.  How: If you have a family history, consider genetic counseling and testing. If you test positive for a high-risk gene, discuss preventive options with your doctor, such as:  Increased surveillance (e.g., more frequent screenings).  Preventive medications (e.g., tamoxifen, raloxifene).  Prophylactic surgery (e.g., mastectomy, oophorectomy) in extreme cases.  10. Reduce Exposure to Environmental Toxins  Why: Some chemicals in the environment may increase breast cancer risk.  How: Minimize exposure by:  Avoiding endocrine-disrupting chemicals (e.g., BPA, phthalates) found in plastics and personal care products.  Using natural cleaning and beauty products.  Limiting exposure to pesticides and industrial chemicals.  11. Manage Stress and Mental Health  Why: Chronic stress and poor mental health may weaken the immune system and contribute to cancer risk.  How: Practice stress-reducing techniques such as:  Meditation or mindfulness.  Yoga or tai chi.  Regular exercise and adequate sleep.  12. Consider Preventive Medications (for High-Risk Individuals)  Why: Certain medications can reduce breast cancer risk in women at high risk.  How: Talk to your doctor about options like:  Tamoxifen or raloxifene (for pre- and postmenopausal women).  Aromatase inhibitors (for postmenopausal women).  13. Stay Informed and Advocate for Your Health  Why: Knowledge is power when it comes to prevention and early detection.  How: Stay updated on breast cancer research, screening guidelines, and lifestyle recommendations. Advocate for your health by asking questions and seeking second opinions if needed.  Key Takeaway  While some risk factors (e.g., genetics, age) cannot be changed, adopting a healthy lifestyle, staying informed, and getting regular screenings can significantly reduce your risk of breast cancer. If you have concerns about your risk, consult your healthcare provider for personalized advice and a prevention plan tailored to your needs. |
| 4 | Can breast cancer be inherited? | Yes, breast cancer can be inherited, but it’s important to note that only a small percentage of breast cancer cases (about 5-10%) are directly linked to inherited genetic mutations. Most breast cancers are caused by a combination of environmental, lifestyle, and random genetic factors.  Inherited Genetic Mutations  Certain inherited gene mutations can significantly increase the risk of breast cancer. The most well-known are:  BRCA1 and BRCA2 Mutations:  These are the most common genes associated with hereditary breast cancer.  Women with a BRCA1 mutation have a 55-65% lifetime risk of developing breast cancer, while those with a BRCA2 mutation have a 45% lifetime risk.  These mutations also increase the risk of ovarian cancer and other cancers.  Other Gene Mutations:  Mutations in genes like TP53, PTEN, PALB2, CHEK2, and ATM can also increase breast cancer risk, though they are less common than BRCA mutations.  Family History and Risk  A strong family history of breast or ovarian cancer can indicate an inherited risk. Red flags include:  Multiple relatives with breast or ovarian cancer.  Breast cancer diagnosed at a young age (under 50).  A family member with cancer in both breasts or male breast cancer.  A family history of other cancers, such as pancreatic or prostate cancer.  Genetic Testing and Counseling  If you have a family history of breast cancer, you may consider:  Genetic Counseling:  A genetic counselor can assess your family history and determine if genetic testing is appropriate.  Genetic Testing:  Testing can identify mutations in genes like BRCA1, BRCA2, and others. This information can help guide prevention and screening strategies.  Prevention for Those with Inherited Risk  If you have an inherited mutation or a strong family history, you can take steps to reduce your risk:  Increased Screening:  More frequent mammograms, MRIs, or clinical breast exams.  Preventive Medications:  Drugs like tamoxifen or raloxifene may reduce risk.  Prophylactic Surgery:  Some women choose to undergo preventive mastectomy or removal of the ovaries to reduce risk.  Lifestyle Changes:  Maintain a healthy weight, exercise regularly, limit alcohol, and avoid smoking.  Important Note  Inherited mutations do not guarantee you will develop breast cancer, but they do increase your risk. If you have concerns about your family history or genetic risk, consult a healthcare provider or genetic counselor for personalized advice. Early detection and proactive management can significantly improve outcomes. |
|  | Repeat | Yes, breast cancer can be inherited, but it’s important to note that only a small percentage of breast cancer cases (about 5–10%) are directly linked to inherited genetic mutations. Most breast cancers are caused by a combination of genetic, environmental, and lifestyle factors. Here’s what you need to know about inherited breast cancer:  Inherited Genetic Mutations and Breast Cancer  Certain inherited genetic mutations can significantly increase the risk of breast cancer. The most well-known genes associated with inherited breast cancer are:  BRCA1 and BRCA2  These genes normally help repair DNA damage and suppress tumor growth.  Mutations in BRCA1 or BRCA2 significantly increase the risk of breast and ovarian cancers.  Women with a BRCA1 mutation have a 55–72% lifetime risk of developing breast cancer, while those with a BRCA2 mutation have a 45–69% lifetime risk.  Men with BRCA mutations also have an increased risk of breast cancer.  Other Genes  Mutations in other genes, such as TP53, PTEN, PALB2, CHEK2, and ATM, can also increase breast cancer risk, though to a lesser extent than BRCA mutations.  How Inherited Breast Cancer Works  These genetic mutations are passed down from parents to children in an autosomal dominant pattern, meaning only one copy of the mutated gene (from either parent) is needed to increase cancer risk.  If one parent carries a mutation, there is a 50% chance of passing it on to their children.  Who Is at Risk of Inherited Breast Cancer?  You may be at higher risk of inherited breast cancer if you have:  A family history of breast, ovarian, or other cancers (especially if diagnosed at a young age).  A relative with a known genetic mutation (e.g., BRCA1, BRCA2).  A personal history of breast cancer diagnosed at a young age (before 50).  A family member with cancer in both breasts or multiple cancers (e.g., breast and ovarian).  Male relatives with breast cancer.  What Should You Do If You’re at Risk?  If you suspect you may have an inherited risk of breast cancer, consider the following steps:  Genetic Counseling and Testing  A genetic counselor can assess your family history and recommend testing for mutations in BRCA1, BRCA2, or other genes.  Testing involves a blood or saliva sample to analyze your DNA.  Increased Surveillance  If you have a genetic mutation, your doctor may recommend more frequent screenings, such as:  Mammograms starting at an earlier age.  Breast MRI for more detailed imaging.  Regular clinical breast exams.  Preventive Medications  Medications like tamoxifen or raloxifene can reduce breast cancer risk in high-risk individuals.  Prophylactic Surgery  Some high-risk individuals choose to undergo preventive mastectomy (removal of one or both breasts) or oophorectomy (removal of the ovaries) to significantly reduce their risk.  Lifestyle Changes  Adopting a healthy lifestyle (e.g., maintaining a healthy weight, limiting alcohol, exercising regularly) can help reduce overall cancer risk.  Key Points to Remember  Inherited breast cancer is caused by specific genetic mutations, such as BRCA1 and BRCA2.  Only a small percentage of breast cancer cases are linked to inherited mutations.  If you have a family history of breast or ovarian cancer, genetic counseling and testing can help assess your risk.  Early detection and preventive measures can significantly reduce the risk of developing breast cancer in high-risk individuals.  If you’re concerned about your risk of inherited breast cancer, consult a healthcare provider or genetic counselor for personalized advice and testing options. |
| 5 | Why is breast cancer screening important? | Breast cancer screening is crucial because it can detect cancer at an early stage, often before symptoms develop. Early detection significantly improves the chances of successful treatment and survival. Here are the key reasons why breast cancer screening is important:  1. Early Detection Saves Lives  Breast cancer is most treatable when caught early. Screening tests like mammograms can identify tumors that are too small to be felt during a physical exam.  Early-stage breast cancer has a 5-year survival rate of over 99%, compared to advanced-stage cancer, which has a much lower survival rate.  2. Reduces the Need for Aggressive Treatment  Detecting breast cancer early often means less aggressive treatment options, such as lumpectomy (removal of the tumor) instead of mastectomy (removal of the entire breast).  Early detection may also reduce the need for chemotherapy or radiation therapy.  3. Identifies Abnormalities Before Symptoms Appear  Many breast cancers do not cause noticeable symptoms in their early stages. Screening can detect abnormalities, such as lumps or calcifications, before they become symptomatic.  4. Helps Monitor High-Risk Individuals  For women with a family history of breast cancer or genetic mutations (e.g., BRCA1 or BRCA2), regular screening is essential for early detection and monitoring.  5. Provides Peace of Mind  Regular screening can reassure women that they are taking proactive steps to protect their health. If an abnormality is found, early intervention can reduce anxiety and improve outcomes.  Types of Breast Cancer Screening  Mammograms:  The most common screening tool, mammograms use low-dose X-rays to detect tumors or abnormalities in the breast.  Recommended for women aged 40 and older, though guidelines vary based on individual risk factors.  Clinical Breast Exams (CBE):  A healthcare provider examines the breasts and underarms for lumps or other changes.  Breast Self-Exams (BSE):  Women can regularly check their own breasts for changes, though this is not a substitute for mammograms or clinical exams.  MRI (Magnetic Resonance Imaging):  Used for women at high risk (e.g., those with BRCA mutations) or to further evaluate abnormalities found during a mammogram.  Screening Guidelines  Screening recommendations vary based on age, risk factors, and guidelines from health organizations. Here are general guidelines:  Average Risk Women:  Start annual or biennial mammograms at age 40-50 and continue until age 74.  High-Risk Women:  Begin screening earlier (e.g., age 30) and include additional tests like MRIs.  Why Screening Matters  Breast cancer is the second leading cause of cancer death in women (after lung cancer). Screening can reduce mortality by detecting cancer early.  Even if you feel healthy, screening is important because breast cancer can develop without symptoms.  Take Action  Talk to your healthcare provider about when to start screening and how often to get tested based on your age, family history, and risk factors.  If you notice any changes in your breasts (e.g., lumps, pain, or discharge), seek medical attention promptly, even if you’ve had a recent screening.  By prioritizing breast cancer screening, you are taking a proactive step toward protecting your health and well-being. |
|  | Repeat | Breast cancer screening is crucial because it can detect cancer early, often before symptoms develop, when treatment is most effective. Early detection significantly improves survival rates and reduces the need for aggressive treatments. Here are the key reasons why breast cancer screening is important:  1. Early Detection Saves Lives  Breast cancer is most treatable when caught in its early stages.  Screening can identify tumors that are too small to be felt during a physical exam, allowing for earlier intervention.  Studies show that regular mammograms can reduce breast cancer mortality by 20–40% in women aged 40–74.  2. Improves Treatment Options  Early-stage breast cancer often requires less aggressive treatment (e.g., lumpectomy instead of mastectomy, less chemotherapy).  Early detection increases the likelihood of successful treatment with fewer side effects.  3. Identifies Cancer Before Symptoms Appear  Many breast cancers, especially in the early stages, do not cause noticeable symptoms.  Screening can detect cancer before it spreads to other parts of the body, improving outcomes.  4. Reduces the Risk of Advanced Cancer  Regular screening helps catch cancer before it progresses to advanced stages, which are harder to treat and more likely to be fatal.  5. Provides Peace of Mind  Regular screenings can reassure individuals that they are taking proactive steps to monitor their health.  If cancer is detected early, patients often feel more in control of their treatment and recovery.  6. Helps Identify High-Risk Individuals  Screening can reveal abnormalities (e.g., dense breast tissue, benign tumors) that may indicate a higher risk of developing breast cancer.  High-risk individuals can then take additional preventive measures, such as more frequent screenings, genetic testing, or preventive medications.  7. Encourages Awareness and Education  Screening programs often include education about breast health, risk factors, and lifestyle changes that can reduce cancer risk.  This empowers individuals to take charge of their health and make informed decisions.  Types of Breast Cancer Screening  Mammograms  The most common and effective screening tool for breast cancer.  Uses low-dose X-rays to detect abnormalities in breast tissue.  Recommended for women aged 40 and older, with frequency depending on risk factors (e.g., annual or biennial screening).  Clinical Breast Exams (CBE)  Performed by a healthcare provider to check for lumps or other changes in the breast.  Often done during routine health checkups.  Breast Self-Exams (BSE)  Regular self-exams help individuals become familiar with their breasts and notice any changes.  While not a substitute for mammograms, BSE can help detect changes that warrant further investigation.  Breast MRI  Used for high-risk individuals (e.g., those with BRCA mutations or a strong family history).  Provides detailed images of breast tissue and is often used in addition to mammograms.  Who Should Get Screened?  Average-Risk Women:  Start mammograms at age 40–50 and continue annually or biennially until age 74, depending on guidelines and individual risk factors.  High-Risk Women:  Begin screening earlier (e.g., age 30) and consider additional tests like breast MRI.  High-risk factors include family history, genetic mutations (e.g., BRCA1/BRCA2), or previous breast cancer.  Key Takeaway  Breast cancer screening is a vital tool for early detection, which can save lives, improve treatment outcomes, and reduce the need for aggressive therapies. If you’re unsure about when to start screening or which tests are right for you, consult your healthcare provider to create a personalized screening plan based on your age, risk factors, and medical history. Regular screening, combined with a healthy lifestyle, is one of the best ways to protect your breast health. |
| 6 | What are the risks of breast cancer screening? | While breast cancer screening is a vital tool for early detection and saving lives, it is not without risks or limitations. Understanding these risks can help you make informed decisions about screening. Here are the potential risks and downsides:  1. False Positives  What it is: A false positive occurs when a screening test suggests cancer, but further testing reveals no cancer.  Risks:  Can cause unnecessary anxiety and stress.  Leads to additional tests, such as diagnostic mammograms, ultrasounds, or biopsies, which can be uncomfortable and costly.  Frequency: About 10% of women screened annually for 10 years will experience a false positive.  2. Overdiagnosis and Overtreatment  What it is: Overdiagnosis occurs when screening detects cancers that would never have caused symptoms or become life-threatening (e.g., slow-growing or non-invasive cancers like ductal carcinoma in situ, or DCIS).  Risks:  May lead to unnecessary treatments, such as surgery, radiation, or chemotherapy, which can have physical and emotional side effects.  Exposes women to risks of treatment without clear benefits.  3. False Negatives  What it is: A false negative occurs when screening misses a cancer that is present.  Risks:  Can lead to a false sense of security and delay diagnosis and treatment.  More common in women with dense breast tissue, as tumors can be harder to detect on mammograms.  4. Radiation Exposure  What it is: Mammograms use low-dose X-rays, which involve minimal radiation exposure.  Risks:  While the radiation dose is very low, repeated exposure over many years could slightly increase cancer risk.  The benefits of early detection generally outweigh the small risk of radiation.  5. Discomfort or Pain  What it is: Mammograms require compressing the breast to get a clear image, which can be uncomfortable or painful for some women.  Risks:  Temporary discomfort during the procedure.  May discourage some women from returning for regular screenings.  6. Psychological Stress  What it is: The screening process, including waiting for results or dealing with false positives, can cause anxiety and stress.  Risks:  Emotional distress from worrying about potential cancer.  Impact on mental health, especially for women with a history of anxiety or cancer.  7. Financial Costs  What it is: Screening tests, follow-up procedures, and treatments can be expensive.  Risks:  Costs may be a barrier for some women, especially if they lack insurance coverage.  Additional tests for false positives can increase financial burden.  8. Limited Effectiveness in Younger Women  What it is: Breast cancer is less common in younger women, and their breast tissue is often denser, making mammograms less effective.  Risks:  Higher rates of false positives and false negatives in women under 40.  Screening may not be recommended for average-risk women under 40 unless they have specific risk factors.  Balancing Risks and Benefits  While these risks exist, the benefits of breast cancer screening—particularly early detection and reduced mortality—generally outweigh the downsides for most women. The key is to make informed decisions based on your individual risk factors, age, and preferences.  What You Can Do  Discuss with Your Doctor:  Talk to your healthcare provider about your personal risk factors and the best screening plan for you.  Understand Your Breast Density:  If you have dense breasts, ask about additional screening options, such as ultrasound or MRI.  Follow Guidelines:  Adhere to recommended screening schedules based on your age and risk level.  Stay Informed:  Keep up-to-date with the latest research and recommendations on breast cancer screening.  By understanding the risks and benefits, you can make empowered decisions about your health and screening options. |
|  | Repeat | While breast cancer screening is a vital tool for early detection and saving lives, it is not without risks. Understanding these risks can help individuals make informed decisions about screening. Here are the potential risks and limitations of breast cancer screening:  1. False Positives  What It Means: A false positive occurs when a screening test (e.g., mammogram) suggests cancer, but further testing reveals no cancer is present.  Risks:  Can cause anxiety and stress.  Leads to additional tests, such as diagnostic mammograms, ultrasounds, or biopsies, which can be invasive and costly.  About 10% of women undergoing mammograms will have a false positive result, and this risk increases with more frequent screenings.  2. Overdiagnosis and Overtreatment  What It Means: Overdiagnosis occurs when screening detects cancers that would never have caused symptoms or become life-threatening (e.g., slow-growing or non-invasive cancers like ductal carcinoma in situ, or DCIS).  Risks:  May lead to unnecessary treatments, such as surgery, radiation, or chemotherapy, which can have physical and emotional side effects.  Estimates suggest that 10–20% of breast cancers detected through screening may be overdiagnosed.  3. False Negatives  What It Means: A false negative occurs when a screening test fails to detect cancer that is present.  Risks:  Can provide a false sense of security, delaying diagnosis and treatment.  False negatives are more common in women with dense breast tissue, as both tumors and dense tissue appear white on mammograms.  4. Radiation Exposure  What It Means: Mammograms use low-dose X-rays, which involve minimal radiation exposure.  Risks:  While the radiation dose is very low, repeated exposure over many years could slightly increase the risk of cancer.  The benefits of early detection generally outweigh the small risk of radiation-induced cancer.  5. Discomfort or Pain During Screening  What It Means: Mammograms involve compressing the breast between two plates to obtain clear images, which can cause temporary discomfort or pain.  Risks:  Some women find the procedure uncomfortable or painful, though it lasts only a few seconds.  Fear of discomfort may discourage some women from getting screened.  6. Psychological Impact  What It Means: The screening process, including false positives or waiting for results, can cause anxiety, stress, or fear.  Risks:  Emotional distress can affect mental health and quality of life, even if the final diagnosis is negative.  Overdiagnosis and overtreatment can also lead to long-term psychological effects.  7. Financial Costs  What It Means: Screening tests, follow-up exams, and treatments for false positives or overdiagnosed cancers can be expensive.  Risks:  Costs may include co-pays, deductibles, or out-of-pocket expenses, depending on insurance coverage.  Financial burden can be a barrier to accessing screening or follow-up care.  8. Limited Effectiveness in Younger Women  What It Means: Breast cancer is less common in younger women, and their breast tissue is often denser, making mammograms less effective.  Risks:  Higher rates of false positives and false negatives in women under 40.  Screening is generally not recommended for average-risk women under 40 unless they have specific risk factors.  9. Potential for Incidental Findings  What It Means: Screening may detect unrelated abnormalities that require further investigation but are ultimately harmless.  Risks:  Can lead to unnecessary tests, procedures, and anxiety.  Balancing Risks and Benefits  While breast cancer screening has risks, the benefits of early detection generally outweigh these concerns for most women, especially those at average or high risk. To minimize risks:  Follow evidence-based screening guidelines tailored to your age, risk factors, and medical history.  Discuss the pros and cons of screening with your healthcare provider to make an informed decision.  If you have dense breasts, ask about supplemental screening options (e.g., ultrasound or MRI).  Stay informed about advances in screening technology and guidelines.  Key Takeaway  Breast cancer screening is a powerful tool for early detection, but it is not perfect. Understanding the risks—such as false positives, overdiagnosis, and radiation exposure—can help you make informed decisions about screening. If you have concerns, talk to your healthcare provider about the best screening plan for your individual needs. |
| 7 | What are the common screening methods for breast cancer? | While breast cancer screening is a vital tool for early detection and saving lives, it is not without risks or limitations. Understanding these risks can help you make informed decisions about screening. Here are the potential risks and downsides:  1. False Positives  What it is: A false positive occurs when a screening test suggests cancer, but further testing reveals no cancer.  Risks:  Can cause unnecessary anxiety and stress.  Leads to additional tests, such as diagnostic mammograms, ultrasounds, or biopsies, which can be uncomfortable and costly.  Frequency: About 10% of women screened annually for 10 years will experience a false positive.  2. Overdiagnosis and Overtreatment  What it is: Overdiagnosis occurs when screening detects cancers that would never have caused symptoms or become life-threatening (e.g., slow-growing or non-invasive cancers like ductal carcinoma in situ, or DCIS).  Risks:  May lead to unnecessary treatments, such as surgery, radiation, or chemotherapy, which can have physical and emotional side effects.  Exposes women to risks of treatment without clear benefits.  3. False Negatives  What it is: A false negative occurs when screening misses a cancer that is present.  Risks:  Can lead to a false sense of security and delay diagnosis and treatment.  More common in women with dense breast tissue, as tumors can be harder to detect on mammograms.  4. Radiation Exposure  What it is: Mammograms use low-dose X-rays, which involve minimal radiation exposure.  Risks:  While the radiation dose is very low, repeated exposure over many years could slightly increase cancer risk.  The benefits of early detection generally outweigh the small risk of radiation.  5. Discomfort or Pain  What it is: Mammograms require compressing the breast to get a clear image, which can be uncomfortable or painful for some women.  Risks:  Temporary discomfort during the procedure.  May discourage some women from returning for regular screenings.  6. Psychological Stress  What it is: The screening process, including waiting for results or dealing with false positives, can cause anxiety and stress.  Risks:  Emotional distress from worrying about potential cancer.  Impact on mental health, especially for women with a history of anxiety or cancer.  7. Financial Costs  What it is: Screening tests, follow-up procedures, and treatments can be expensive.  Risks:  Costs may be a barrier for some women, especially if they lack insurance coverage.  Additional tests for false positives can increase financial burden.  8. Limited Effectiveness in Younger Women  What it is: Breast cancer is less common in younger women, and their breast tissue is often denser, making mammograms less effective.  Risks:  Higher rates of false positives and false negatives in women under 40.  Screening may not be recommended for average-risk women under 40 unless they have specific risk factors.  Balancing Risks and Benefits  While these risks exist, the benefits of breast cancer screening—particularly early detection and reduced mortality—generally outweigh the downsides for most women. The key is to make informed decisions based on your individual risk factors, age, and preferences.  What You Can Do  Discuss with Your Doctor:  Talk to your healthcare provider about your personal risk factors and the best screening plan for you.  Understand Your Breast Density:  If you have dense breasts, ask about additional screening options, such as ultrasound or MRI.  Follow Guidelines:  Adhere to recommended screening schedules based on your age and risk level.  Stay Informed:  Keep up-to-date with the latest research and recommendations on breast cancer screening.  By understanding the risks and benefits, you can make empowered decisions about your health and screening options.  What are the common screening methods for breast cancer?  The server is busy. Please try again later.  What are the common screening methods for breast cancer?  Breast cancer screening involves methods to detect cancer before symptoms appear. Early detection improves treatment outcomes and survival rates. Here are the most common screening methods:  1. Mammogram  What it is: A mammogram is an X-ray of the breast tissue. It is the most widely used and effective screening tool for breast cancer.  How it works: The breast is compressed between two plates to spread out the tissue, and images are taken from different angles.  Who it’s for:  Women aged 40 and older (guidelines vary; some recommend starting at 50).  Women at average risk or higher risk of breast cancer.  Benefits:  Can detect tumors that are too small to be felt.  Reduces breast cancer mortality by catching cancer early.  Limitations:  May produce false positives or false negatives.  Less effective in women with dense breast tissue.  2. Clinical Breast Exam (CBE)  What it is: A physical examination of the breasts and underarms by a healthcare provider.  How it works: The provider checks for lumps, changes in size or shape, or other abnormalities.  Who it’s for:  Women of all ages, often as part of a routine check-up.  Benefits:  Can identify changes that may not show up on a mammogram.  Provides an opportunity to discuss breast health with a provider.  Limitations:  Less effective than mammograms for detecting early-stage cancer.  Relies on the skill and experience of the provider.  3. Breast Self-Exam (BSE)  What it is: A woman examines her own breasts for changes, such as lumps, pain, or discharge.  How it works: Regularly check your breasts for any unusual changes and report them to a doctor.  Who it’s for:  Women of all ages, as a way to stay familiar with their breast tissue.  Benefits:  Encourages breast awareness and early reporting of changes.  Limitations:  Not a substitute for mammograms or clinical exams.  May lead to unnecessary anxiety if changes are detected.  4. Breast MRI (Magnetic Resonance Imaging)  What it is: An MRI uses magnetic fields and radio waves to create detailed images of the breast.  How it works: A contrast dye is injected into the bloodstream to highlight abnormalities.  Who it’s for:  Women at high risk (e.g., BRCA mutations, strong family history).  Used alongside mammograms for women with dense breast tissue.  Benefits:  Highly sensitive and can detect cancers missed by mammograms.  Limitations:  Higher rate of false positives.  Expensive and not routinely used for average-risk women.  5. Ultrasound  What it is: An ultrasound uses sound waves to create images of the breast tissue.  How it works: A handheld device (transducer) is moved over the breast to capture images.  Who it’s for:  Often used as a follow-up to mammograms or clinical exams.  Helpful for women with dense breast tissue or to evaluate lumps.  Benefits:  Can distinguish between solid masses and fluid-filled cysts.  No radiation exposure.  Limitations:  Not a primary screening tool for average-risk women.  May miss small tumors.  6. 3D Mammography (Tomosynthesis)  What it is: An advanced form of mammography that creates a 3D image of the breast.  How it works: Multiple images are taken from different angles and reconstructed into a 3D image.  Who it’s for:  Women of all ages, especially those with dense breast tissue.  Benefits:  Reduces false positives and improves detection of small tumors.  Limitations:  May involve higher radiation exposure than standard mammograms (though still within safe limits).  Not always available or covered by insurance.  7. Genetic Testing  What it is: Testing for mutations in genes like BRCA1 and BRCA2 that increase breast cancer risk.  How it works: A blood or saliva sample is analyzed for specific genetic mutations.  Who it’s for:  Women with a strong family history of breast or ovarian cancer.  Benefits:  Identifies high-risk individuals who may benefit from enhanced screening or preventive measures.  Limitations:  Does not detect cancer itself; only assesses risk.  Can cause anxiety or uncertainty about results.  Screening Guidelines  Screening recommendations vary based on age, risk factors, and guidelines from health organizations. Here are general guidelines:  Average-Risk Women:  Start mammograms at age 40-50 and continue every 1-2 years until age 74.  High-Risk Women:  Begin screening earlier (e.g., age 30) and include additional tests like MRI.  Takeaway  Breast cancer screening is a powerful tool for early detection and saving lives. Talk to your healthcare provider to determine the best screening plan for you based on your age, risk factors, and preferences. Regular screening, combined with breast awareness, can significantly improve outcomes. |
|  | Repeat | Breast cancer screening involves methods to detect cancer before symptoms appear, allowing for early intervention and better outcomes. Here are the common screening methods for breast cancer:  1. Mammogram  What It Is: A mammogram is an X-ray of the breast tissue used to detect abnormalities, such as lumps or calcifications.  How It Works: The breast is compressed between two plates to spread out the tissue and obtain clear images.  Types:  Screening Mammogram: Used for routine checkups in women without symptoms.  Diagnostic Mammogram: Used to investigate abnormalities found during a screening mammogram or physical exam.  Who Should Get It:  Average-risk women: Start at age 40–50 and continue annually or biennially until age 74, depending on guidelines.  High-risk women: May start earlier and have more frequent screenings.  Benefits:  Reduces breast cancer mortality by detecting cancer early.  Effective for finding small tumors that cannot be felt.  Limitations:  Can produce false positives or false negatives.  Less effective in women with dense breast tissue.  2. Clinical Breast Exam (CBE)  What It Is: A physical examination of the breasts performed by a healthcare provider to check for lumps or other changes.  How It Works: The provider uses their hands to feel for abnormalities in the breast and underarm areas.  Who Should Get It:  Often done during routine health checkups for women in their 20s and 30s.  May be part of screening for high-risk women.  Benefits:  Complements mammograms and self-exams.  Helps detect changes that may not show up on imaging.  Limitations:  Less effective than mammograms for detecting early-stage cancer.  Dependent on the skill of the examiner.  3. Breast Self-Exam (BSE)  What It Is: A regular check performed by individuals to become familiar with their breasts and notice any changes.  How It Works: Individuals use their hands to feel for lumps, thickening, or other changes in the breast tissue.  Who Should Do It:  Recommended for all women as part of breast awareness, though not a substitute for mammograms.  Benefits:  Helps individuals detect changes early and report them to a healthcare provider.  Encourages proactive health monitoring.  Limitations:  Not a reliable standalone screening method.  Can lead to unnecessary anxiety if changes are detected.  4. Breast MRI (Magnetic Resonance Imaging)  What It Is: A imaging technique that uses magnetic fields and radio waves to create detailed images of the breast.  How It Works: A contrast dye is injected into the bloodstream to highlight abnormalities in the breast tissue.  Who Should Get It:  High-risk women, such as those with BRCA mutations, a strong family history, or dense breast tissue.  Often used in addition to mammograms, not as a replacement.  Benefits:  Highly sensitive for detecting cancer, especially in dense breast tissue.  Useful for monitoring high-risk individuals.  Limitations:  Higher rate of false positives compared to mammograms.  Expensive and not routinely recommended for average-risk women.  5. Breast Ultrasound  What It Is: An imaging technique that uses sound waves to create pictures of the breast tissue.  How It Works: A handheld device (transducer) is moved over the breast to capture images.  Who Should Get It:  Often used as a follow-up to mammograms or clinical exams to investigate abnormalities.  Helpful for women with dense breast tissue or those who cannot undergo MRI.  Benefits:  Non-invasive and does not use radiation.  Useful for distinguishing between solid masses (e.g., tumors) and fluid-filled cysts.  Limitations:  Not a standalone screening tool for average-risk women.  Less effective than mammograms for detecting early-stage cancer.  6. Tomosynthesis (3D Mammography)  What It Is: An advanced form of mammography that creates 3D images of the breast by taking multiple X-ray pictures from different angles.  How It Works: Similar to a traditional mammogram but provides more detailed images.  Who Should Get It:  Increasingly used for routine screening, especially in women with dense breast tissue.  Benefits:  Reduces false positives and improves cancer detection rates compared to 2D mammograms.  Better at detecting small tumors in dense tissue.  Limitations:  May involve slightly higher radiation exposure than 2D mammograms (though still within safe limits).  Not universally available.  7. Thermography  What It Is: A non-invasive imaging technique that detects heat patterns and blood flow in the breast.  How It Works: An infrared camera is used to measure temperature differences in the breast tissue.  Who Should Get It:  Not recommended as a standalone screening tool.  Sometimes marketed as an alternative to mammograms, but its effectiveness is not well-supported by evidence.  Benefits:  Non-invasive and does not use radiation.  Limitations:  Not as accurate as mammograms for detecting cancer.  Not endorsed by major medical organizations for routine screening.  Key Takeaway  The most common and effective screening method for breast cancer is the mammogram, especially for women aged 40 and older. Additional methods, such as clinical breast exams, breast MRI, and ultrasound, may be used for high-risk individuals or as follow-up tests. While no screening method is perfect, regular screening can significantly improve early detection and outcomes. Talk to your healthcare provider to determine the best screening plan based on your age, risk factors, and medical history. |
| 8 | What is the survival rate of breast cancer? | The survival rate for breast cancer varies depending on the stage at diagnosis, the type of breast cancer, and individual factors such as age, overall health, and access to treatment. Here’s an overview of breast cancer survival rates based on the stage of diagnosis:  5-Year Relative Survival Rates  The 5-year relative survival rate compares the survival of people with breast cancer to the survival of the general population. It is often used to describe the likelihood of living at least 5 years after diagnosis.  Localized (Early-Stage) Breast Cancer:  Cancer is confined to the breast.  5-year survival rate: 99%.  Early detection through screening significantly improves outcomes.  Regional Breast Cancer:  Cancer has spread to nearby lymph nodes or tissues.  5-year survival rate: 86%.  Distant (Metastatic) Breast Cancer:  Cancer has spread to distant organs (e.g., lungs, liver, bones).  5-year survival rate: 31%.  All Stages Combined:  The overall 5-year survival rate for breast cancer is 91%.  10-Year Relative Survival Rates  For longer-term outlook, the 10-year relative survival rate is also considered:  Localized: 84%.  Regional: 63%.  Distant: 13%.  Factors Affecting Survival Rates  Stage at Diagnosis:  Early detection through screening (e.g., mammograms) dramatically improves survival rates.  Type of Breast Cancer:  Hormone receptor-positive (ER+/PR+) cancers generally have better outcomes.  Triple-negative breast cancer (TNBC) and HER2-positive cancers may have lower survival rates, though advances in treatment are improving outcomes.  Age and Overall Health:  Younger, healthier patients often have better survival rates.  Access to Treatment:  Timely and effective treatment, including surgery, chemotherapy, radiation, and targeted therapies, improves survival.  Lifestyle Factors:  Maintaining a healthy weight, exercising, and avoiding smoking can improve outcomes.  Improving Survival Rates  Early Detection: Regular mammograms and breast self-exams can help catch cancer at an early, more treatable stage.  Advances in Treatment: New therapies, such as immunotherapy and targeted drugs, are improving survival rates, even for advanced-stage cancers.  Personalized Medicine: Tailoring treatment based on the specific characteristics of the cancer (e.g., hormone receptors, genetic mutations) is leading to better outcomes.  Takeaway  While breast cancer survival rates have improved significantly over the past few decades, early detection and access to effective treatment remain critical. If you have concerns about breast cancer risk or symptoms, consult a healthcare provider for personalized advice and screening recommendations. Regular check-ups and proactive health measures can make a significant difference in outcomes. |
|  | Repeat | The survival rate for breast cancer varies widely depending on factors such as the stage at diagnosis, the type of breast cancer, the patient's age, and the effectiveness of treatment. Survival rates are typically expressed as 5-year relative survival rates, which compare the likelihood of survival for people with breast cancer to that of the general population. Here’s an overview of breast cancer survival rates:  Overall Survival Rates  All Stages Combined:  The 5-year relative survival rate for breast cancer is 90%. This means that, on average, women with breast cancer are 90% as likely to live for at least 5 years after diagnosis as women without breast cancer.  The 10-year relative survival rate is approximately 84%.  Survival Rates by Stage  Breast cancer survival rates are strongly influenced by the stage at diagnosis. The stages are based on the size of the tumor, whether it has spread to lymph nodes, and whether it has metastasized (spread to distant organs).  Stage 0 (Carcinoma in Situ):  5-year survival rate: Nearly 100%.  This is the earliest stage, where cancer cells are confined to the milk ducts (ductal carcinoma in situ, or DCIS) or lobules (lobular carcinoma in situ, or LCIS) and have not invaded surrounding tissue.  Stage I:  5-year survival rate: 98–100%.  The tumor is small (≤2 cm) and has not spread to lymph nodes or distant sites.  Stage II:  5-year survival rate: 90–93%.  The tumor is larger (2–5 cm) or has spread to a few nearby lymph nodes but not to distant organs.  Stage III:  5-year survival rate: 72–75%.  The cancer is locally advanced, with larger tumors (>5 cm) and/or significant spread to lymph nodes but no distant metastasis.  Stage IV (Metastatic):  5-year survival rate: 29–31%.  The cancer has spread to distant organs, such as the bones, liver, lungs, or brain. While not curable, treatments can help manage the disease and improve quality of life.  Survival Rates by Breast Cancer Type  Hormone Receptor-Positive (HR+):  Generally has a better prognosis because it responds well to hormone therapy.  5-year survival rate: Approximately 90%.  HER2-Positive:  Historically had a poorer prognosis, but targeted therapies (e.g., trastuzumab) have significantly improved outcomes.  5-year survival rate: Approximately 85–90%.  Triple-Negative Breast Cancer (TNBC):  More aggressive and harder to treat due to the lack of hormone receptors and HER2 protein.  5-year survival rate: Approximately 77%.  Factors Influencing Survival Rates  Early Detection:  Survival rates are significantly higher when breast cancer is detected early, before it spreads to lymph nodes or distant organs.  Age and Overall Health:  Younger women and those in good health tend to have better outcomes.  Type and Subtype of Breast Cancer:  Some types (e.g., HR+) are more treatable than others (e.g., TNBC).  Access to Treatment:  Timely access to surgery, chemotherapy, radiation, and targeted therapies improves survival.  Lifestyle Factors:  Maintaining a healthy weight, exercising, and avoiding smoking can improve outcomes.  Improving Survival Rates  Regular Screening: Mammograms and other screening methods can detect cancer early, when it is most treatable.  Advances in Treatment: Targeted therapies, immunotherapy, and personalized medicine are improving outcomes for many patients.  Healthy Lifestyle: A balanced diet, regular exercise, and avoiding alcohol and smoking can reduce the risk of recurrence and improve overall health.  Key Takeaway  The survival rate for breast cancer is generally high, especially when detected early. The 5-year relative survival rate is 90% for all stages combined, but this varies significantly by stage, type, and individual factors. Early detection through regular screening and advances in treatment are key to improving survival rates. If you have concerns about breast cancer, consult your healthcare provider for personalized advice and screening recommendations. |
| 9 | What are the treatment methods of breast cancer? | Breast cancer treatment depends on the type, stage, and characteristics of the cancer, as well as the patient’s overall health and preferences. A combination of treatments is often used for the best outcomes. Here are the primary treatment methods for breast cancer:  1. Surgery  Surgery is often the first step in treating breast cancer and involves removing the tumor and some surrounding tissue.  Lumpectomy (Breast-Conserving Surgery):  Removes the tumor and a small margin of healthy tissue.  Often followed by radiation therapy.  Suitable for early-stage breast cancer.  Mastectomy:  Removes the entire breast. Types include:  Simple mastectomy: Removal of the breast tissue.  Modified radical mastectomy: Removal of the breast tissue and lymph nodes.  Skin-sparing or nipple-sparing mastectomy: Preserves the skin or nipple for reconstruction.  May be recommended for larger tumors or multiple tumors.  Lymph Node Removal:  Sentinel lymph node biopsy: Removes a few lymph nodes to check for cancer spread.  Axillary lymph node dissection: Removes multiple lymph nodes if cancer is detected.  Breast Reconstruction:  Can be done during or after mastectomy to restore the breast’s appearance.  2. Radiation Therapy  Uses high-energy beams to kill cancer cells or prevent them from growing.  Often used after lumpectomy or mastectomy to reduce the risk of recurrence.  Types include:  External beam radiation: Targets the breast or chest wall.  Brachytherapy: Places radioactive material inside the breast near the tumor.  3. Chemotherapy  Uses drugs to kill cancer cells or stop their growth.  May be used:  Before surgery (neoadjuvant therapy): To shrink tumors.  After surgery (adjuvant therapy): To kill remaining cancer cells.  For advanced or metastatic breast cancer.  Common drugs include anthracyclines, taxanes, and platinum-based agents.  4. Hormone Therapy  Used for hormone receptor-positive (ER+ or PR+) breast cancers.  Blocks hormones (estrogen or progesterone) that fuel cancer growth.  Common treatments include:  Tamoxifen: Blocks estrogen receptors.  Aromatase inhibitors (e.g., letrozole, anastrozole): Reduce estrogen production in postmenopausal women.  Ovarian suppression: Shuts down estrogen production in premenopausal women.  5. Targeted Therapy  Targets specific molecules or pathways involved in cancer growth.  Common targeted therapies include:  HER2-targeted drugs (e.g., trastuzumab, pertuzumab): For HER2-positive breast cancer.  CDK4/6 inhibitors (e.g., palbociclib, ribociclib): For hormone receptor-positive, HER2-negative breast cancer.  PARP inhibitors (e.g., olaparib): For BRCA-mutated breast cancer.  6. Immunotherapy  Boosts the immune system to fight cancer.  Checkpoint inhibitors (e.g., pembrolizumab) are used for triple-negative breast cancer (TNBC) that expresses PD-L1.  7. Treatment by Stage  Stage 0 (DCIS):  Lumpectomy + radiation or mastectomy.  Hormone therapy may be recommended for hormone receptor-positive DCIS.  Stage I-II (Early-Stage):  Surgery (lumpectomy or mastectomy) + radiation.  Chemotherapy, hormone therapy, or targeted therapy based on tumor characteristics.  Stage III (Locally Advanced):  Neoadjuvant chemotherapy to shrink the tumor, followed by surgery and radiation.  Adjuvant hormone therapy or targeted therapy.  Stage IV (Metastatic):  Focuses on controlling cancer growth and improving quality of life.  Treatments include chemotherapy, hormone therapy, targeted therapy, immunotherapy, and radiation for symptom relief.  8. Palliative Care  Focuses on relieving symptoms and improving quality of life for advanced-stage patients.  May include pain management, nutritional support, and emotional counseling.  Emerging Treatments  CAR-T Cell Therapy: A type of immunotherapy being studied for breast cancer.  Vaccines: Experimental vaccines aim to prevent recurrence or treat advanced breast cancer.  Liquid Biopsies: Blood tests to monitor cancer and guide treatment decisions.  Personalized Treatment Plans  Breast cancer treatment is highly individualized. Factors considered include:  Tumor size, grade, and stage.  Hormone receptor and HER2 status.  Genetic mutations (e.g., BRCA1/2).  Patient age, overall health, and preferences.  Takeaway  Breast cancer treatment has advanced significantly, offering many options for patients. If you or a loved one is diagnosed, work closely with a multidisciplinary team (oncologists, surgeons, radiologists) to develop a personalized treatment plan. Early detection and timely treatment are key to improving outcomes. |
|  | Repeat | Breast cancer treatment depends on the type, stage, and characteristics of the cancer, as well as the patient’s overall health and preferences. Treatment often involves a combination of therapies to achieve the best outcomes. Here are the common treatment methods for breast cancer:  1. Surgery  Surgery is often the first line of treatment for breast cancer and aims to remove the tumor and surrounding tissue.  Types of Surgery:  Lumpectomy (Breast-Conserving Surgery):  Removes the tumor and a small margin of healthy tissue.  Often followed by radiation therapy to kill any remaining cancer cells.  Suitable for early-stage breast cancer.  Mastectomy:  Removes the entire breast.  Types include:  Simple Mastectomy: Removal of the breast tissue only.  Modified Radical Mastectomy: Removal of the breast tissue and lymph nodes.  Double Mastectomy: Removal of both breasts, often done for high-risk individuals or bilateral cancer.  Sentinel Lymph Node Biopsy:  Removes a few lymph nodes to check for cancer spread.  Helps determine if further lymph node removal is needed.  Axillary Lymph Node Dissection:  Removes multiple lymph nodes if cancer is found in the sentinel nodes.  Reconstructive Surgery:  Restores the shape of the breast after mastectomy or lumpectomy.  Can be done immediately after surgery or later.  2. Radiation Therapy  Uses high-energy beams (e.g., X-rays or protons) to kill cancer cells or prevent them from growing.  Often used after lumpectomy or mastectomy to reduce the risk of recurrence.  May also be used to treat metastatic breast cancer in specific areas (e.g., bones, brain).  Types of Radiation Therapy:  External Beam Radiation: Delivered from outside the body.  Brachytherapy (Internal Radiation): Places radioactive material inside the breast near the tumor.  3. Chemotherapy  Uses drugs to kill cancer cells or stop their growth.  Can be given before surgery (neoadjuvant chemotherapy) to shrink tumors or after surgery (adjuvant chemotherapy) to kill remaining cancer cells.  Also used for advanced or metastatic breast cancer.  Common Chemotherapy Drugs:  Anthracyclines (e.g., doxorubicin).  Taxanes (e.g., paclitaxel, docetaxel).  Platinum-based drugs (e.g., carboplatin).  4. Hormone Therapy  Used for hormone receptor-positive (HR+) breast cancers, which grow in response to estrogen or progesterone.  Blocks hormones or lowers their levels to prevent cancer growth.  Types of Hormone Therapy:  Selective Estrogen Receptor Modulators (SERMs):  E.g., tamoxifen, raloxifene.  Blocks estrogen receptors in breast tissue.  Aromatase Inhibitors (AIs):  E.g., letrozole, anastrozole, exemestane.  Lowers estrogen levels in postmenopausal women.  Ovarian Suppression:  Shuts down the ovaries (e.g., with drugs or surgery) to reduce estrogen production in premenopausal women.  5. Targeted Therapy  Uses drugs or other substances to specifically target cancer cells without harming normal cells.  Often used for HER2-positive or advanced breast cancers.  Types of Targeted Therapy:  HER2-Targeted Drugs:  E.g., trastuzumab (Herceptin), pertuzumab (Perjeta), ado-trastuzumab emtansine (Kadcyla).  Blocks the HER2 protein, which promotes cancer growth.  CDK4/6 Inhibitors:  E.g., palbociclib (Ibrance), ribociclib (Kisqali).  Used for HR+, HER2-negative advanced breast cancer.  PARP Inhibitors:  E.g., olaparib (Lynparza), talazoparib (Talzenna).  Used for BRCA-mutated breast cancer.  PI3K Inhibitors:  E.g., alpelisib (Piqray).  Used for HR+, HER2-negative breast cancer with a PIK3CA mutation.  6. Immunotherapy  Boosts the body’s immune system to fight cancer.  Used for triple-negative breast cancer (TNBC) or advanced breast cancer.  Types of Immunotherapy:  Checkpoint Inhibitors:  E.g., pembrolizumab (Keytruda), atezolizumab (Tecentriq).  Helps the immune system recognize and attack cancer cells.  7. Bone-Directed Therapy  Used to treat or prevent bone metastases (spread of cancer to bones).  Helps strengthen bones and reduce pain.  Types of Bone-Directed Therapy:  Bisphosphonates: E.g., zoledronic acid.  Denosumab: A monoclonal antibody that prevents bone breakdown.  8. Palliative Care  Focuses on relieving symptoms and improving quality of life for patients with advanced or metastatic breast cancer.  Can be used alongside other treatments.  Treatment by Stage  Stage 0 (DCIS/LCIS): Lumpectomy + radiation or mastectomy.  Stage I–II: Surgery (lumpectomy or mastectomy) + radiation, chemotherapy, or hormone therapy.  Stage III: Neoadjuvant chemotherapy, surgery, radiation, and/or targeted therapy.  Stage IV (Metastatic): Systemic therapies (chemotherapy, hormone therapy, targeted therapy, immunotherapy) + palliative care.  Key Takeaway  Breast cancer treatment is highly personalized and may involve a combination of surgery, radiation, chemotherapy, hormone therapy, targeted therapy, and immunotherapy. The choice of treatment depends on the cancer’s characteristics, stage, and the patient’s preferences. Early detection and advances in treatment have significantly improved outcomes for breast cancer patients. If you or a loved one is diagnosed with breast cancer, work closely with your healthcare team to develop a treatment plan tailored to your needs. |
| 10 | Can breast cancer recur? | Yes, breast cancer can recur, even after successful treatment. Recurrence happens when cancer cells that were not completely eliminated during initial treatment begin to grow again. The risk of recurrence depends on factors such as the stage and type of breast cancer, the treatments received, and individual health factors. Here’s an overview of breast cancer recurrence:  Types of Breast Cancer Recurrence  Local Recurrence:  Cancer returns in the same breast (after lumpectomy) or in the chest wall or skin (after mastectomy).  Often detected early through regular follow-up exams or imaging.  Regional Recurrence:  Cancer returns in nearby lymph nodes (e.g., under the arm or near the collarbone).  May require additional surgery, radiation, or systemic therapy.  Distant Recurrence (Metastatic):  Cancer spreads to distant organs, such as the bones, liver, lungs, or brain.  Treated as stage IV (metastatic) breast cancer, with a focus on controlling growth and improving quality of life.  Risk Factors for Recurrence  Several factors can increase the risk of breast cancer recurrence:  Cancer Stage at Diagnosis:  Higher-stage cancers (e.g., stage III) are more likely to recur than early-stage cancers (e.g., stage I).  Tumor Characteristics:  Larger tumors, high-grade cancers, and cancers with lymph node involvement have a higher risk of recurrence.  Triple-negative breast cancer (TNBC) and HER2-positive cancers are associated with a higher risk of recurrence.  Incomplete Initial Treatment:  Skipping or delaying treatments like surgery, radiation, chemotherapy, or hormone therapy can increase recurrence risk.  Age and Health:  Younger women (under 35) and those with certain genetic mutations (e.g., BRCA1/2) may have a higher risk.  Lifestyle Factors:  Obesity, smoking, and lack of physical activity can increase recurrence risk.  Signs and Symptoms of Recurrence  Symptoms depend on where the cancer recurs:  Local or Regional Recurrence:  A new lump in the breast, chest wall, or underarm.  Skin changes, such as redness, dimpling, or thickening.  Pain or swelling in the breast or nearby areas.  Distant Recurrence:  Persistent pain (e.g., in bones, back, or joints).  Shortness of breath or a chronic cough (lung involvement).  Jaundice, abdominal pain, or swelling (liver involvement).  Headaches, dizziness, or seizures (brain involvement).  Reducing the Risk of Recurrence  While recurrence cannot always be prevented, the following strategies can help lower the risk:  Complete Initial Treatment:  Follow your treatment plan, including surgery, radiation, chemotherapy, hormone therapy, or targeted therapy.  Hormone Therapy:  For hormone receptor-positive cancers, taking hormone therapy (e.g., tamoxifen, aromatase inhibitors) for 5-10 years can significantly reduce recurrence risk.  Lifestyle Changes:  Maintain a healthy weight, exercise regularly, limit alcohol, and avoid smoking.  Regular Follow-Up Care:  Attend all scheduled check-ups, mammograms, and imaging tests to detect recurrence early.  Genetic Testing and Counseling:  If you have a family history or genetic mutation (e.g., BRCA1/2), consider preventive measures like prophylactic surgery or enhanced screening.  Treatment for Recurrent Breast Cancer  Treatment depends on the type and location of recurrence:  Local or Regional Recurrence:  Surgery, radiation, or systemic therapy (chemotherapy, hormone therapy, or targeted therapy).  Distant Recurrence:  Focuses on controlling cancer growth and improving quality of life.  Treatments include chemotherapy, hormone therapy, targeted therapy, immunotherapy, and radiation for symptom relief.  Emotional Support  A breast cancer recurrence can be emotionally challenging. Seek support from:  Healthcare providers.  Support groups or counseling.  Family and friends.  Takeaway  While breast cancer can recur, advances in treatment and early detection have improved outcomes for many patients. Staying vigilant with follow-up care, adhering to treatment plans, and making healthy lifestyle choices can help reduce the risk of recurrence and improve long-term survival. If you notice any new symptoms or changes, contact your healthcare provider promptly. |
|  | Repeat | Yes, breast cancer can recur, even after successful treatment. Recurrence happens when cancer cells that were not completely eliminated during initial treatment begin to grow again. The risk of recurrence depends on factors such as the stage and type of breast cancer, the effectiveness of initial treatment, and individual health and lifestyle factors. Here’s what you need to know about breast cancer recurrence:  Types of Breast Cancer Recurrence  Local Recurrence  Cancer returns in the same area as the original tumor (e.g., the breast, chest wall, or surgical scar).  More common in women who had breast-conserving surgery (lumpectomy) rather than mastectomy.  Symptoms may include a new lump, skin changes, or nipple discharge.  Regional Recurrence  Cancer returns in nearby lymph nodes or tissues (e.g., under the arm or near the collarbone).  Symptoms may include swelling or a lump in the lymph nodes.  Distant Recurrence (Metastatic Recurrence)  Cancer spreads to distant organs, such as the bones, liver, lungs, or brain.  This is the most serious type of recurrence and is considered stage IV breast cancer.  Symptoms depend on the affected organ (e.g., bone pain, shortness of breath, headaches).  Risk Factors for Recurrence  Several factors can increase the risk of breast cancer recurrence:  Cancer Stage at Diagnosis:  Higher-stage cancers (e.g., stage III) are more likely to recur than early-stage cancers (e.g., stage I).  Tumor Characteristics:  Triple-negative breast cancer (TNBC) and HER2-positive breast cancer have higher recurrence rates.  Larger tumors and cancer spread to lymph nodes increase the risk.  Treatment Received:  Incomplete treatment (e.g., skipping radiation or hormone therapy) increases the risk.  Lack of access to advanced treatments may also play a role.  Age at Diagnosis:  Younger women (under 35) have a higher risk of recurrence.  Lifestyle Factors:  Obesity, smoking, alcohol use, and lack of physical activity can increase recurrence risk.  Genetic Mutations:  BRCA1 or BRCA2 mutations are associated with a higher risk of recurrence.  Reducing the Risk of Recurrence  While recurrence cannot always be prevented, the following strategies can help reduce the risk:  Complete Initial Treatment:  Follow your treatment plan, including surgery, radiation, chemotherapy, hormone therapy, or targeted therapy.  Hormone Therapy:  For hormone receptor-positive (HR+) breast cancer, taking hormone therapy (e.g., tamoxifen, aromatase inhibitors) for 5–10 years can significantly reduce recurrence risk.  Lifestyle Changes:  Maintain a healthy weight.  Exercise regularly (e.g., 150 minutes of moderate activity per week).  Limit alcohol consumption and avoid smoking.  Eat a balanced diet rich in fruits, vegetables, and whole grains.  Regular Follow-Up Care:  Attend all scheduled checkups, mammograms, and other screenings.  Report any new symptoms (e.g., lumps, pain, or changes in the breast) to your doctor immediately.  Targeted Therapy:  For HER2-positive breast cancer, HER2-targeted drugs (e.g., trastuzumab) can reduce recurrence risk.  For BRCA-mutated breast cancer, PARP inhibitors (e.g., olaparib) may be recommended.  Monitoring for Recurrence  After completing treatment, regular follow-up care is essential to detect recurrence early. This may include:  Clinical Breast Exams: Every 3–6 months for the first few years, then annually.  Mammograms: Annually or as recommended by your doctor.  Other Imaging Tests: MRI, ultrasound, or CT scans if needed.  Blood Tests: To monitor tumor markers (e.g., CA 15-3, CEA).  Bone Scans or PET Scans: If symptoms suggest distant recurrence.  Treatment for Recurrent Breast Cancer  If breast cancer recurs, treatment options depend on the type and location of recurrence:  Local or Regional Recurrence: Surgery, radiation, or systemic therapy (chemotherapy, hormone therapy, targeted therapy).  Distant Recurrence (Metastatic): Systemic therapies to control the cancer and improve quality of life.  Palliative Care: To manage symptoms and provide emotional support.  Key Takeaway  Breast cancer can recur, but the risk varies depending on the cancer’s characteristics, treatment, and lifestyle factors. Completing your initial treatment, making healthy lifestyle choices, and attending regular follow-up appointments can help reduce the risk of recurrence. If you have concerns about recurrence, talk to your healthcare provider about personalized strategies for monitoring and prevention. Early detection of recurrence improves treatment outcomes and quality of life. |
