# Supplementary material for: ChatGPT Versus DeepSeek for Breast Cancer Information Retrieval: Quantitative Comparative Study
Source: JMIR Cancer. 2026 Feb 27;12:e72839. doi: 10.2196/72839 (PMC12954694; doi:10.2196/72839)
Supplement: Multimedia Appendix 2 — Frequently asked questions about breast cancer and answers generated by artificial intelligence models (ChatGPT and DeepSeek) by Researcher 2. [file cancer-v12-e72839-s002.docx]

**Supporting Information: Supplementary Table 2**

**ChatGPT vs. DeepSeek: A Comparative Analysis of AI Models for Breast Cancer Information Retrieval**

Rima Hajjo^1,2*^, Dima A. Sabbah^1^, Sanaa K. Bardaweel^3^

*^1^Department of Pharmacy, Faculty of Pharmacy, Al-Zaytoonah University of Jordan, P.O. Box 130, Amman 11733, Jordan.*

*^2^Laboratory for Molecular Modeling, Division of Chemical Biology and Medicinal Chemistry, Eshelman School of Pharmacy, The University of North Carolina at Chapel Hill, Chapel Hill, NC, USA.*

*^3^Department of Pharmaceutical Sciences, School of Pharmacy, University of Jordan, Amman 11942, Jordan.*

*Correspondence

Rima Hajjo

E-mail: rhajjo@gmail.com; [r.hajjo@zuj.edu.jo](mailto:r.hajjo@zuj.edu.jo)

ORCID: https://orcid.org/0000-0002-7090-5425

**Supplementary Table 2.** Frequently asked questions about breast cancer and answers generated by artificial intelligence models (ChatGPT and DeepSeek) by Researcher 2.

| **ChatGPT 4.0** | | | |
| --- | --- | --- | --- |
|  | **Question** | **Answer** | |
| 1 | What are the common symptoms of breast cancer? | Common symptoms of breast cancer can vary, but here are some of the most typical signs to look out for:   1. **Lump in the breast**: A new lump or mass in the breast that feels hard or different from the surrounding tissue is one of the most common signs. 2. **Changes in the size, shape, or appearance of the breast**: This can include the breast becoming enlarged or having an unusual shape. 3. **Unexplained pain or tenderness**: Pain in the breast or nipple that doesn’t go away could be a symptom. 4. **Changes in the skin over the breast**: Skin dimpling or puckering, similar to the texture of an orange peel, might occur. 5. **Nipple discharge**: Unexplained discharge from the nipple, other than milk (especially if it’s bloody or clear), can be a red flag. 6. **Swelling in the armpit or around the collarbone**: Swollen lymph nodes can sometimes be noticed in these areas if the cancer has spread. 7. **Redness or warmth**: The skin of the breast may become red or warm to the touch.   Not all of these symptoms necessarily mean cancer, but if someone experiences them, it’s important to consult a healthcare provider for further evaluation. Early detection plays a big role in effective treatment.  Have you or someone you know been dealing with any of these symptoms, or are you just exploring this topic out of curiosity? | |
|  | Repeat1 | Common symptoms of breast cancer can vary, but some typical signs to watch for include:   1. **Changes in the Breast or Nipple** 2. **Lump or thickening:** A new lump or mass in the breast or underarm is often the most common symptom. It may feel hard, irregular, or different from the rest of the tissue. 3. **Change in size or shape:** One breast may appear larger or have an unusual contour. 4. **Nipple discharge:** Any fluid (other than breast milk) from the nipple, especially if bloody or clear. 5. **Nipple inversion or retraction:** Turning inward of the nipple. 6. **Changes to the skin on the breast:** Redness, dimpling, puckering, or skin that looks like an orange peel (peau d'orange). 7. **Itching, flaking, or scaling of the nipple or breast skin.**   **Other Symptoms**   1. **Pain:** Persistent pain in the breast or nipple (though breast cancer is often painless). 2. **Swelling:** Even without a distinct lump, swelling of part or all of the breast can occur. 3. **Lymph node changes:** Swelling or a lump in the underarm area.   **Note:**  These symptoms do not always indicate breast cancer, as other benign conditions may cause similar changes. However, it is essential to seek medical evaluation if you notice any unusual changes. Early detection improves treatment outcomes.  Would you like to know more about risk factors or screening guidelines? | |
| 2 | What are the risk factors for breast cancer? | The risk factors for breast cancer can be divided into categories based on modifiable and non-modifiable factors. Here's an overview   1. **Non-Modifiable Risk Factors**   **Gender:** Women are at much higher risk than men.  **Age:** The risk increases with age, particularly after age 50.  **Family History:** Having a first-degree relative (mother, sister, or daughter) with breast cancer increases risk.  **Genetic Mutations:** BRCA1, BRCA2, and other genetic mutations significantly raise the risk.  **Personal History:** A previous diagnosis of breast cancer or certain non-cancerous breast diseases increases the risk.  **Menstrual History:** Early menstruation (before age 12) and late menopause (after age 55) increase exposure to estrogen, raising the risk.  **Dense Breast Tissue:** Women with dense breast tissue have a higher risk and may have less accurate mammogram results.  **Race and Ethnicity:** White women have a slightly higher risk than women of other ethnicities.  **Radiation Exposure:** Previous radiation therapy to the chest, especially during adolescence, increases risk.   1. **Modifiable Risk Factors**   **Hormone Replacement Therapy (HRT):** Long-term use of combined estrogen and progesterone therapy can increase the risk.  **Oral Contraceptives:** Some studies suggest a slight increase in risk with prolonged use of birth control pills.  **Alcohol Consumption:** Even moderate alcohol intake increases the risk.  **Obesity and Weight Gain:** Being overweight, especially after menopause, raises the risk.  **Physical Inactivity:** Lack of regular exercise is associated with higher risk.  **Diet:** Diets high in saturated fats may contribute to increased risk.  **Smoking:** Some evidence suggests smoking is linked to a higher risk, especially in premenopausal women.  **Reproductive History:** Having children at a later age or never having children can increase risk. Breastfeeding appears to reduce risk.   1. **Emerging Factors (Potential but Less Established)**   **Environmental Exposures:** Chemicals such as BPA and pollutants are being investigated.  **Night Shift Work:** Disruption of circadian rhythms may increase the risk.  **Stress:** While difficult to quantify, chronic stress may contribute to hormonal imbalances.  Would you like more information on prevention strategies or specific risk factor details? | |
|  | Repeat 1 | Several factors can increase the risk of developing breast cancer. While having one or more of these risk factors does not guarantee that a person will develop breast cancer, being aware of them is essential for early detection and prevention. Here's a comprehensive list:  **Lifestyle and Environmental Factors**  **Alcohol consumption:** Higher alcohol intake increases risk.  **Obesity:** Especially after menopause.  **Physical inactivity:** Regular exercise can reduce risk.  **Diet:** High-fat diets may contribute to an increased risk.  **Smoking:** Associated with a higher risk, particularly in premenopausal women.  **Reproductive and Hormonal Factors**  **Early menstruation:** Before age 12.  **Late menopause:** After age 55.  **Late or no pregnancy:** First pregnancy after age 30 or never being pregnant.  **Not breastfeeding:** Breastfeeding can lower the risk.  **Use of hormone replacement therapy (HRT):** Long-term use, especially combined estrogen-progestin therapy.  **Birth control pills:** Slightly increased risk while taking them.  **Genetic Factors**  **Family history:** Having a first-degree relative (mother, sister, or daughter) with breast cancer increases risk.  **Inherited gene mutations:** BRCA1 and BRCA2 mutations significantly increase risk.  **Medical and Radiation Factors**  **Previous breast cancer or benign breast conditions:** History of certain non-cancerous breast conditions or breast cancer.  **Radiation exposure:** Radiation therapy to the chest, particularly during adolescence or early adulthood.  **Dense breast tissue:** Makes it harder to detect tumors and may independently increase risk.  **Age and Gender Factors**  **Gender:** Women are at a much higher risk than men.  **Age:** Risk increases with age, particularly after 50.  **Ethnic and Racial Factors**  **Race/Ethnicity:** White women are slightly more likely to develop breast cancer, but African American women are more likely to develop aggressive forms and have higher mortality rates.  **Other Factors**  **Diethylstilbestrol (DES) exposure:** Women who took DES during pregnancy and their daughters may have an increased risk.  **Night shift work:** Disruption of the body’s natural circadian rhythms may be linked to an increased risk.  Would you like more detailed information on any specific factor or recommendations for screening and prevention? | |
| 3 | How to prevent breast cancer? | While there's no guaranteed way to prevent breast cancer, adopting certain lifestyle changes and staying informed can significantly reduce the risk. Here are evidence-based recommendations:  1. **Maintain a Healthy Lifestyle**  **Exercise regularly:** Aim for at least 150 minutes of moderate activity or 75 minutes of vigorous activity weekly.  **Healthy diet:** Include plenty of fruits, vegetables, whole grains, and lean proteins. The Mediterranean diet is often recommended.  **Maintain a healthy weight:** Excess body fat, especially after menopause, increases breast cancer risk.  2. **Limit Alcohol and Avoid Smoking**  **Limit alcohol:** Keep alcohol intake to no more than one drink per day, if at all.  **Quit smoking:** Smoking has been linked to an increased risk of breast cancer, especially in younger and premenopausal women.  3. **Hormonal and Environmental Factors**  **Limit hormone therapy:** Discuss alternatives or the shortest possible duration for hormone replacement therapy (HRT) with your doctor.  **Avoid unnecessary radiation exposure:** Only undergo medical scans when necessary.  4. **Self-Awareness and Regular Screenings**  **Breast self-exams:** Familiarize yourself with your breast tissue and look out for any changes.  **Clinical breast exams and mammograms:** Follow guidelines based on age and family history. Early detection improves outcomes.  5. **Know Your Family History**  **Genetic testing:** If you have a family history of breast cancer, consider consulting a genetic counselor. BRCA1 and BRCA2 gene mutations significantly increase the risk.  6. **Consider Preventive Medications or Surgery (High-Risk Individuals)**  Medications like **tamoxifen** or **raloxifene** may be prescribed for women at high risk.  Prophylactic mastectomy or oophorectomy can be an option for those with a significant genetic risk.  7. **Minimize Exposure to Toxins**  Avoid exposure to endocrine disruptors found in certain plastics, cosmetics, and pesticides. Choose products labeled free from harmful chemicals.  Do you want more specific details on any of these points? | |
|  | Repeat 1 | Preventing breast cancer involves a combination of lifestyle choices, regular screenings, and awareness of risk factors. While no method guarantees prevention, you can reduce your risk by adopting the following strategies:  **1. Maintain a Healthy Lifestyle**  **Exercise Regularly:** Aim for at least 150 minutes of moderate activity or 75 minutes of vigorous activity per week.  **Healthy Diet:** Include plenty of fruits, vegetables, whole grains, and lean proteins. Limit processed foods and added sugars.  **Healthy Weight:** Maintain a healthy body weight, especially after menopause, as obesity increases breast cancer risk.  **2. Limit Alcohol and Quit Smoking**  **Alcohol Consumption:** Limit alcohol to no more than one drink per day or avoid it altogether.  **Avoid Smoking:** Smoking has been linked to various cancers, including breast cancer.  **3. Hormonal and Medication Awareness**  **Limit Hormone Therapy:** If hormone replacement therapy (HRT) is necessary, use the lowest dose for the shortest duration possible.  **Preventive Medications:** In high-risk individuals, medications like tamoxifen or raloxifene may be prescribed by doctors.  **4. Breastfeeding**  Breastfeeding, when possible, has been associated with a reduced risk of breast cancer.  **5. Environmental Exposure**  **Avoid Radiation and Pollution:** Limit exposure to high-dose radiation when possible.  **Choose Safer Personal Care Products:** Some chemicals in cosmetics and cleaning products may act as hormone disruptors.  **6. Genetic and Family History**  **Know Your Family History:** Inform your healthcare provider if you have a family history of breast or ovarian cancer.  **Genetic Counseling:** If you are at high risk, consider genetic counseling and testing for mutations in BRCA1, BRCA2, and other genes.  **7. Regular Screenings and Self-Awareness**  **Mammograms:** Follow screening recommendations based on your age and risk level (usually starting at age 40 or earlier if at high risk).  **Clinical Breast Exams:** Consult your healthcare provider for the recommended frequency.  **Self-Examinations:** Be familiar with how your breasts normally look and feel to detect any unusual changes early.  Would you like a checklist or more personalized advice based on age or family history? | |
| 4 | Can breast cancer be inherited? | Yes, breast cancer can be inherited in some cases. While most breast cancers are not linked to family history, approximately 5% to 10% of cases are hereditary. In these cases, genetic mutations passed down from parents can increase the risk of developing breast cancer.   1. Common Genetic Mutations   **BRCA1 and BRCA2:** Mutations in these genes significantly increase the risk of breast and ovarian cancer.  **PALB2, TP53, and CHEK2:** Mutations in these genes are also linked to an increased risk of breast cancer.   1. Family History Indicators   You may be at higher risk if  Multiple family members have had breast or ovarian cancer, especially at a young age.  Male breast cancer is present in your family.  A known genetic mutation exists within your family.   1. Genetic Testing   If you have a strong family history, genetic testing can help identify whether you carry a mutation associated with a higher risk of breast cancer. Genetic counseling is often recommended before and after testing to understand the results and implications.  Would you like more information on genetic testing or risk-reducing strategies? | |
|  | Repeat 1 | Yes, breast cancer can be inherited in some cases, but most instances are not directly related to family history.   1. **Inherited Risk Factors**   About 5-10% of breast cancer cases are hereditary, often due to inherited gene mutations, particularly in the following genes  **BRCA1 and BRCA2:** Mutations in these genes significantly increase the risk of breast and ovarian cancer.  **PALB2, TP53, and CHEK2:** These are other gene mutations that can also elevate the risk of breast cancer.   1. **Family History**   You may have a higher risk if:  Close relatives (mother, sister, or daughter) have been diagnosed with breast cancer, especially at a young age.  Multiple family members on the same side have had breast or ovarian cancer.  There's a known presence of BRCA gene mutations in your family.   1. **Genetic Testing**   If you have a strong family history, genetic counseling and testing can help assess your risk and inform preventive steps.   1. **Non-Hereditary Factors**   The majority of breast cancer cases are due to non-genetic factors like age, hormonal influences, lifestyle, and environmental factors.  Would you like information on genetic testing or ways to reduce risk? | |
| 5 | Why is breast cancer screening important? | Breast cancer screening is crucial because it helps detect breast cancer at an early stage, often before symptoms appear. Early detection increases the chances of successful treatment and improves survival rates. Here’s why screening is essential:  **1. Early Detection Saves Lives**  When breast cancer is caught early, treatment options are more effective, less invasive, and have better outcomes.  Survival rates are significantly higher for early-stage breast cancer compared to late-stage diagnoses.  **2. Identifying Cancer Before Symptoms Develop**  Screening can detect abnormalities in breast tissue that may not be felt during a physical exam or cause noticeable symptoms.  **3. Improved Treatment Options**  Early detection may allow for less aggressive treatments, such as breast-conserving surgery instead of mastectomy.  **4. Better Quality of Life**  Catching cancer early can reduce the physical and emotional impact of more advanced disease and intensive treatments.  **5. Risk Reduction for High-Risk Individuals**  Women with a family history of breast cancer or genetic predispositions (like BRCA1 or BRCA2 mutations) can benefit significantly from regular screenings.  **Types of Screening**  **Mammography:** The most common and effective screening tool.  **Clinical Breast Exam:** Performed by a healthcare professional.  **Breast MRI:** Recommended for high-risk individuals.  **Self-Exams:** Encourage familiarity with your own body to notice changes.  Would you like more information on the recommended screening guidelines based on age or risk factors? | |
|  | Repeat 1 | Breast cancer screening is essential because it helps detect breast cancer early, often before any symptoms develop. Early detection significantly increases the chances of successful treatment and can lead to better outcomes. Here are some key reasons why breast cancer screening is important:  1. **Early Detection Saves Lives**  Detecting breast cancer at an early stage increases the likelihood of effective treatment and lowers the risk of death.  2. **More Treatment Options**  Early-stage breast cancer often has more treatment options, including less aggressive treatments that may preserve more breast tissue.  3. **Improved Prognosis**  Early diagnosis is associated with higher survival rates and a better long-term prognosis.  4. **Less Invasive Treatment**  Cancers found early may be treated with lumpectomy (removing only the tumor) rather than a full mastectomy, and may not require chemotherapy.  5. **Monitoring for High-Risk Individuals**  Regular screening is especially important for those with a family history or genetic predisposition to breast cancer.  6. **Peace of Mind**  Regular screening can provide reassurance and reduce anxiety for many women by confirming the absence of cancer.  **Common Screening Methods:**  **Mammogram:** The most common and effective method for early detection.  **Breast MRI:** Recommended for high-risk individuals.  **Clinical Breast Exam:** Performed by a healthcare provider.  **Self-Exams:** Awareness of changes in the breasts can prompt further testing.  Would you like information on recommended screening guidelines or how to schedule a screening? | |
| 6 | What are the risks of breast cancer screening? | Breast cancer screening, typically involving mammography, can be a vital tool for early detection. However, like any medical procedure, it comes with certain risks. Here are the primary ones  1. **False Positives**  **What it is:** When a screening detects an abnormality that appears suspicious but turns out to be non-cancerous.  **Risk:** Can lead to unnecessary stress, further tests (like biopsies or additional imaging), and follow-up procedures.  2. **False Negatives**  **What it is:** When a screening misses cancer that is actually present.  **Risk:** May provide a false sense of security and delay proper diagnosis and treatment.  3. **Overdiagnosis and Overtreatment**  **What it is:** Detection of cancers that would not have caused symptoms or harm during a person's lifetime.  **Risk:** Can lead to unnecessary treatments, such as surgery, radiation, or hormone therapy, which come with their own side effects.  4. **Radiation Exposure**  **What it is:** Mammograms use low-dose X-rays.  **Risk:** Repeated exposure over time may slightly increase the risk of developing cancer, although the risk is minimal.  5. **Psychological Impact**  **What it is:** Anxiety and emotional distress related to the screening process, waiting for results, or receiving abnormal findings.  **Risk:** Can impact mental health, even when results are ultimately negative.  6. **Physical Discomfort**  **What it is:** Compression of the breast during mammography.  **Risk:** Can cause temporary pain or discomfort.  7. **Cost and Accessibility**  **What it is:** Depending on insurance coverage and healthcare systems, screening can be costly or logistically challenging.  **Risk:** May result in financial stress or reduced access to care.  How to Mitigate Risks  **Informed Decision-Making:** Discuss the pros and cons of screening with a healthcare provider.  **Individualized Screening Plans:** Tailor screening frequency and type based on personal and family risk factors.  **Stay Informed:** Keep up with guidelines and emerging technologies that might reduce these risks.  Would you like information on the latest guidelines or more details on reducing specific risks? | |
|  | Repeat | Breast cancer screening, such as mammograms, is generally beneficial for early detection and treatment of breast cancer. However, it also comes with some risks and limitations. Below are the main risks associated with breast cancer screening:  1. **False Positives**  A screening may indicate the presence of cancer when none exists.  This can lead to anxiety, additional tests (like biopsies), and unnecessary stress.  2. **False Negatives**  Sometimes cancer is present but not detected by the screening.  This can delay diagnosis and treatment.  3. **Overdiagnosis**  Some detected cancers may be slow-growing and would never cause harm during the patient's lifetime.  This can lead to unnecessary treatments such as surgery, radiation, or chemotherapy.  4. **Radiation Exposure**  Mammograms involve exposure to low-dose radiation.  Although the risk is small, repeated exposure over time may slightly increase the risk of developing cancer.  5. **Psychological Stress**  The process of waiting for results or undergoing follow-up tests can cause anxiety.  6. **Discomfort or Pain**  Mammograms can be physically uncomfortable or painful as the breast tissue is compressed during the procedure.  7. **False Assurance**  Negative screening results may give a false sense of security, possibly leading to ignoring future breast changes or symptoms.  8. **Financial and Time Costs**  Follow-up tests and treatments can be costly and time-consuming.  Balancing Risks and Benefits  The benefits of early detection often outweigh these risks for most people, especially those at higher risk for breast cancer. Guidelines typically recommend that women discuss their individual risk factors and preferences with their healthcare provider to determine an appropriate screening schedule.  Would you like details on screening guidelines or alternative methods? | |
| 7 | What are the common screening methods for breast cancer? | Breast cancer screening involves various methods to detect cancer early, often before symptoms arise, thereby improving treatment outcomes. The most common screening methods include:  1. Mammography: mammography is the most common screening test for breast cancer.t uses low-dose X-rays to create images of the breast, allowing for the detection of tumors that cannot be felt regular screening mammograms can reduce breast cancer deaths, especially among women aged 50 to 69. Cite turn search8  2. Clinical Breast Exam (CBE): clinical breast exam is performed by a healthcare professional who checks for lumps or other physical changes in the breasts while evidence is limited on its effectiveness in reducing breast cancer mortality, it remains a common practice in many healthcare settings. Cite turn search6  3. Breast Self-Exam (BSE): breast self-exam involves a woman examining her own breasts for lumps, changes, or abnormalities while BSEs can help women become familiar with their breasts and identify changes, they have not been shown to reduce breast cancer mortality. Cite turn0search6  4. Magnetic Resonance Imaging (MRI): RI uses magnetic fields to create detailed images of the breast and may be used to screen women who have a high risk of breast cancer. It's more sensitive than mammography but also has a higher false-positive rate, leading to additional testing. Cite turns earch1 5. Ultrasound: Breast ultrasound uses sound waves to produce images of the breast tissue. It’s often used as a supplemental tool to mammography, especially in women with dense breast tissue, where mammograms may be less effective. Cite turn0search11 it's important to consult with a healthcare provider to determine the most appropriate screening strategy based on individual risk factors, such as age, family history, and breast density. Regular screenings and awareness of personal breast health are key components in the early detection of breast cancer. | |
|  | Repeat | Common screening methods for breast cancer include:  1. **Mammography**  **Description:** X-ray imaging of the breast to detect abnormalities.  **Purpose:** Often considered the gold standard for breast cancer screening.  **Recommended Frequency:** Annually or biennially for women aged 40 and older, depending on individual risk factors.  2. **Breast Ultrasound**  **Description:** Uses sound waves to create images of breast tissue.  **Purpose:** Commonly used for women with dense breast tissue or to further evaluate abnormalities found in a mammogram.  3. **Magnetic Resonance Imaging (MRI)**  **Description:** Uses magnetic fields and radio waves to produce detailed images of the breast.  **Purpose:** Recommended for high-risk individuals, such as those with a strong family history or genetic predisposition (like BRCA mutations).  4. **Clinical Breast Exam (CBE)**  **Description:** A physical examination performed by a healthcare provider to feel for lumps or abnormalities.  **Purpose:** Often part of routine check-ups.  5. **Self-Breast Examination (SBE)**  **Description:** Self-examination of the breasts to detect lumps or changes.  **Purpose:** Helps women become familiar with their breast tissue, though it's less emphasized as a formal screening method by many medical guidelines.  Emerging Methods:  **3D Mammography (Tomosynthesis):** Provides a more detailed view by taking multiple X-ray images from different angles.  **Molecular Breast Imaging (MBI):** Uses a radioactive tracer to detect cancer, particularly in dense breast tissue.  The choice of screening method depends on factors like age, breast density, personal and family medical history, and genetic predisposition. Regular discussions with a healthcare provider are essential to determine the most appropriate screening plan. | |
| 8 | What is the survival rate of breast cancer? | Breast cancer survival rates vary based on factors such as the cancer's stage at diagnosis, tumor characteristics, and individual health conditions .In the United States, the overall 5-year relative survival rate for breast cancer is approximately 91%. cite turn0search5 survival rates are often categorized by the extent of cancer spread at the time of diagnosis:  Localized: cancer confined to the breast.  Regional: cancer spread to nearby lymph nodes or tissues.  Distant: cancer spread to distant organs. The 5-year relative survival rates for these categories are:  Localized: 9%  Regional: 6%  Distant: 1%  cite turn0search1  These statistics are based on data from the U.S. and may differ in other countries .It's important to note that survival rates are averages and can vary depending on individual circumstances. Advancements in early detection and treatment have contributed to improved survival rates over time or the most accurate and personalized information, it's recommended to consult with healthcare professionals who can provide guidance based on specific medical conditions. | |
|  | Repeat | Breast cancer survival rates vary based on factors such as the cancer's stage at diagnosis, tumor characteristics, and individual health conditions. In the United States, the overall 5-year relative survival rate for breast cancer is approximately 91%. Cite turn0 search5 survival rates are often categorized by the extent of cancer spread at the time of diagnosis:  Localized: Cancer confined to the breast. Regional: Cancer spread to nearby structures or lymph nodes.  - Distant: Cancer spread to distant organs. The 5-year relative survival rates for these categories are:  Localized: 9%- Regional: 6%- Distant: 1% cite turn0search1  These statistics are based on data from the U.S. and may differ in other countries. It's important to note that survival rates are averages and can vary depending on individual circumstances. Early detection through regular screenings and advancements in treatment have significantly improved survival rates over the past few decades. Navlist Recent Insights on Breast Cancer Trends turn0news15,turn0news20 | |
| 9 | What are the treatment methods of breast cancer? | The treatment of breast cancer depends on several factors, including the stage, type, hormone receptor status, genetic factors, and overall health of the patient. Common treatment methods include:  1. **Surgery**  **Lumpectomy:** Removal of the tumor and a small margin of surrounding tissue, preserving the rest of the breast.  **Mastectomy:** Removal of the entire breast. Variants include total mastectomy and modified radical mastectomy.  **Lymph Node Surgery:** Removal of lymph nodes in the armpit to check for cancer spread.  2. **Radiation Therapy**  **External Beam Radiation:** High-energy X-rays are directed at the breast tissue.  **Brachytherapy (Internal Radiation):** Radioactive seeds are placed inside or near the tumor site.  3. **Chemotherapy**  Use of powerful drugs to destroy cancer cells or shrink tumors before surgery (neoadjuvant therapy) or after surgery (adjuvant therapy).  Commonly administered intravenously or orally.  4. **Hormone Therapy (Endocrine Therapy)**  For hormone receptor-positive breast cancers:  **Tamoxifen:** Blocks estrogen receptors.  **Aromatase Inhibitors (Anastrozole, Letrozole, Exemestane):** Reduce estrogen production in postmenopausal women.  **Ovarian Suppression:** Shutting down estrogen production from the ovaries.  5. **Targeted Therapy**  **HER2-Targeted Therapy:** For HER2-positive cancers (e.g., Trastuzumab, Pertuzumab).  **CDK4/6 Inhibitors:** (e.g., Palbociclib, Ribociclib).  **PI3K Inhibitors:** (e.g., Alpelisib).  **Antibody-Drug Conjugates:** (e.g., T-DM1).  6. **Immunotherapy**  Used for triple-negative breast cancer.  **Immune Checkpoint Inhibitors:** (e.g., Atezolizumab, Pembrolizumab).  7. **Clinical Trials**  Participation in clinical trials to access experimental treatments under development.  8. **Supportive and Palliative Care**  Pain management, mental health support, and nutritional counseling.  Treatment plans are typically customized by a team of oncologists based on individual patient factors. Would you like detailed information on any specific method or its side effects? | |
|  | Repeat | Breast cancer treatment depends on factors such as the type, stage, hormone receptor status, genetic factors, and the patient's overall health. Below are common treatment methods:  **1. Surgery**  **Lumpectomy (Breast-Conserving Surgery):** Removes the tumor and a small margin of surrounding tissue.  **Mastectomy:** Removes the entire breast; can be followed by breast reconstruction.  **Lymph Node Removal:** Sentinel or axillary lymph node dissection may be done to check for cancer spread.  **2. Radiation Therapy**  Uses high-energy rays to target and destroy cancer cells.  **External Beam Radiation:** Most common form, directed at the breast from outside the body.  **Brachytherapy:** Internal radiation where radioactive seeds are placed inside or near the tumor site.  **3. Chemotherapy**  Uses drugs to kill or slow the growth of cancer cells.  Can be administered before surgery (neoadjuvant) or after surgery (adjuvant).  Sometimes used to treat advanced or metastatic breast cancer.  **4. Hormone Therapy (Endocrine Therapy)**  Used for hormone receptor-positive (ER/PR-positive) breast cancers to block the effects of estrogen or reduce estrogen production.  **Medications:** Tamoxifen, aromatase inhibitors, and ovarian suppression treatments.  **5. Targeted Therapy**  Focuses on specific molecules involved in cancer growth and spread.  Examples include:  **HER2-targeted therapies:** Trastuzumab (Herceptin), Pertuzumab.  **CDK4/6 inhibitors:** Palbociclib (Ibrance).  **PARP inhibitors:** Olaparib (Lynparza) for BRCA-mutated cancers.  **6. Immunotherapy**  Stimulates the immune system to recognize and attack cancer cells.  Example: Atezolizumab combined with chemotherapy for certain triple-negative breast cancers.  **7. Clinical Trials**  Offer access to new treatments not yet widely available.  May involve novel drugs, combinations, or advanced therapies.  **8. Palliative Care**  Focuses on relieving symptoms and improving quality of life for advanced cancer patients.  Would you like more details on any particular treatment, or guidance on the pros and cons of each option? | |
| 10 | Can breast cancer recur? | Yes, breast cancer can recur. Recurrence means that the cancer returns after treatment and a period of being cancer-free. Breast cancer recurrence can happen months, years, or even decades after the initial treatment.  Types of Recurrence:  **Local Recurrence:** Cancer returns in the same breast, chest wall, or nearby tissue where the original tumor was located.  **Regional Recurrence:** Cancer reappears in nearby lymph nodes.  **Distant (Metastatic) Recurrence:** Cancer spreads to distant parts of the body, such as the bones, liver, lungs, or brain.  Factors Influencing Recurrence:  **Stage of cancer at diagnosis:** Higher stages have a greater risk of recurrence.  **Hormone receptor status:** Estrogen-positive cancers may have late recurrences.  **Lymph node involvement:** More lymph node involvement increases recurrence risk.  **Tumor size and grade:** Larger or higher-grade tumors may be more aggressive.  **Type of treatment received:** Incomplete or less effective treatments may contribute to recurrence.  Preventive Measures:  **Adjuvant therapy:** Treatments like hormone therapy, chemotherapy, and radiation help reduce recurrence risks.  **Lifestyle changes:** Maintaining a healthy weight, regular exercise, and limiting alcohol consumption may reduce risk.  **Regular follow-up:** Routine check-ups and mammograms help monitor for early signs of recurrence.  Would you like more information on symptoms to watch for or steps to reduce the risk of recurrence? | |
|  | Repeat | Yes, breast cancer can recur even after successful treatment. Recurrence can happen months, years, or even decades after the initial treatment. The likelihood of recurrence depends on various factors, including the type of breast cancer, stage at diagnosis, and the treatments received.  Types of Recurrence  **Local recurrence:** Cancer returns in the same breast, chest wall, or surgical scar.  **Regional recurrence:** Cancer returns in nearby lymph nodes.  **Distant (metastatic) recurrence:** Cancer spreads to other parts of the body, such as bones, liver, lungs, or brain.  Signs of Recurrence  A new lump in the breast or chest wall  Changes to the skin of the breast or around the scar  Swelling in the lymph nodes near the armpit or collarbone  Persistent bone pain, cough, or unexplained weight loss (for distant recurrence)  Reducing the Risk  Follow-up care and regular check-ups  Hormone therapy, if indicated  Maintaining a healthy lifestyle (diet, exercise, avoiding smoking)  Adherence to prescribed medications, such as Tamoxifen or aromatase inhibitors  If there's a specific concern or recent development, I can provide more tailored information or guide you on what to discuss with a healthcare provider. | |
| **DeepSeek** | | |  |
|  | Question | Answer |  |
| 1 | What are the common symptoms of breast cancer? | Breast cancer symptoms can vary, and some individuals may not experience any symptoms at all, especially in the early stages. However, common signs and symptoms of breast cancer include:  1. **Lump in the Breast or Underarm**: A new lump or mass that feels different from the surrounding tissue is one of the most common signs. It may be hard, painless, and have irregular edges, but some lumps can be soft, tender, or rounded.  2. **Changes in Breast Size or Shape**: Swelling, shrinkage, or asymmetry of the breast, especially if it occurs on one side.  3. **Skin Changes**: Dimpling, puckering, or redness of the breast skin, resembling the texture of an orange peel (peau d'orange).  4. **Nipple Changes**: Inversion of the nipple (turning inward), nipple discharge (other than breast milk, which may be clear, bloody, or another color), or scaling, crusting, or peeling of the nipple or surrounding skin.  5. **Pain**: Persistent pain in the breast or nipple, though pain alone is not usually a sign of breast cancer and can be caused by other conditions.  6. **Swelling**: Swelling in part or all of the breast, even if no distinct lump is felt.  7. **Redness or Thickening**: Redness, thickening, or warmth of the breast skin.  8. **Lymph Node Changes**: Swelling or a lump in the lymph nodes under the arm or near the collarbone.  It's important to note that these symptoms can also be caused by non-cancerous conditions, such as cysts or infections. However, if you notice any persistent changes in your breasts, it's essential to consult a healthcare professional for evaluation and appropriate testing, such as a mammogram, ultrasound, or biopsy. Early detection and treatment significantly improve outcomes for breast cancer. |  |
|  | Repeat | Breast cancer symptoms can vary, and some individuals may not experience any symptoms at all, especially in the early stages. However, common signs and symptoms of breast cancer include:  1. **Lump or Thickening**: A new lump or mass in the breast or underarm area. It may feel hard, irregular, and different from surrounding tissue.  2. **Changes in Breast Size or Shape**: Swelling, shrinkage, or asymmetry of the breast.  3. **Skin Changes**: Dimpling, puckering, or redness of the breast skin, resembling the texture of an orange peel (peau d'orange).  4. **Nipple Changes**:  - Inversion (turning inward) of the nipple.  - Nipple discharge (other than breast milk), which may be bloody or clear.  5. **Pain**: Persistent pain or tenderness in the breast or nipple area.  6. **Swelling**: Swelling in part or all of the breast, even if no distinct lump is felt.  7. **Redness or Scaling**: Redness, flakiness, or thickening of the nipple or breast skin.  8. **Lymph Node Changes**: Swelling or lumps in the lymph nodes under the arm or near the collarbone.  It's important to note that these symptoms can also be caused by non-cancerous conditions. However, if you notice any persistent or unusual changes in your breasts, it's essential to consult a healthcare professional for evaluation and diagnosis. Early detection and treatment significantly improve outcomes for breast cancer. Regular screenings, such as mammograms, are also crucial for early detection, especially for individuals with risk factors. |  |
| 2 | What are the risk factors for breast cancer? | Breast cancer is a complex disease with multiple risk factors, some of which are modifiable while others are not. Here are the key risk factors:  ### Non-Modifiable Risk Factors:  1. **Gender**: Women are at a much higher risk of developing breast cancer compared to men.  2. **Age**: The risk increases with age, particularly after 50.  3. **Family History**: A family history of breast cancer, especially in first-degree relatives (mother, sister, daughter), increases risk.  4. **Genetic Mutations**: Inherited mutations in genes such as BRCA1 and BRCA2 significantly increase the risk.  5. **Personal History**: A previous diagnosis of breast cancer or certain non-cancerous breast diseases can increase the risk.  6. **Race and Ethnicity**: White women are slightly more likely to develop breast cancer than women of other races, though African American women are more likely to develop more aggressive forms.  7. **Dense Breast Tissue**: Women with denser breast tissue have a higher risk.  8. **Reproductive History**: Early menstruation (before age 12) and late menopause (after age 55) increase risk due to longer exposure to hormones.  ### Modifiable Risk Factors:  1. **Lifestyle Factors**:  - **Alcohol Consumption**: Regular alcohol consumption increases risk.  - **Smoking**: Smoking, especially long-term, is associated with increased risk.  - **Physical Inactivity**: Lack of regular physical activity can contribute to higher risk.  - **Diet**: A diet high in saturated fats and low in fruits and vegetables may increase risk.  2. **Hormonal Factors**:  - **Hormone Replacement Therapy (HRT)**: Long-term use of combined estrogen and progesterone HRT increases risk.  - **Oral Contraceptives**: Some studies suggest a slight increase in risk with long-term use.  3. **Weight and Body Composition**:  - **Obesity**: Being overweight or obese, particularly after menopause, increases risk due to higher levels of estrogen produced by fat tissue.  4. **Reproductive Factors**:  - **Childbearing**: Having no children or having the first child after age 30 increases risk.  - **Breastfeeding**: Not breastfeeding or breastfeeding for a shorter duration may slightly increase risk.  ### Other Risk Factors:  1. **Radiation Exposure**: Previous radiation therapy to the chest area, especially during adolescence, increases risk.  2. **Environmental Factors**: Exposure to certain chemicals and pollutants may contribute to increased risk, though this is less well understood.  ### Summary:  While some risk factors like genetics and age cannot be changed, others related to lifestyle and environment can be modified to reduce risk. Regular screening and early detection are crucial for managing breast cancer risk effectively. Always consult with healthcare professionals for personalized advice and risk assessment. |  |
|  | Repeat | Breast cancer is a complex disease with multiple risk factors, some of which are modifiable while others are not. Here are the key risk factors:  ### Non-Modifiable Risk Factors:  1. **Gender**: Women are at a much higher risk of developing breast cancer compared to men.  2. **Age**: Risk increases with age, particularly after 50.  3. **Family History**: A family history of breast cancer, especially in first-degree relatives (mother, sister, daughter), increases risk.  4. **Genetic Mutations**: Inherited mutations in genes such as BRCA1 and BRCA2 significantly increase risk.  5. **Personal History**: A previous diagnosis of breast cancer or certain non-cancerous breast diseases (e.g., atypical hyperplasia) increases risk.  6. **Race and Ethnicity**: White women are slightly more likely to develop breast cancer than African American women, though African American women are more likely to die from the disease. Ashkenazi Jewish women have a higher risk due to BRCA mutations.  7. **Dense Breast Tissue**: Women with denser breast tissue have a higher risk.  8. **Reproductive History**: Early menstruation (before age 12) and late menopause (after age 55) increase risk due to longer exposure to hormones.  ### Modifiable Risk Factors:  1. **Hormone Replacement Therapy (HRT)**: Long-term use of combined estrogen and progesterone HRT increases risk.  2. **Oral Contraceptives**: Some studies suggest a slight increase in risk with current or recent use.  3. **Alcohol Consumption**: Regular alcohol consumption increases risk, with higher consumption correlating with higher risk.  4. **Obesity**: Being overweight or obese, particularly after menopause, increases risk.  5. **Physical Inactivity**: Lack of regular physical activity is associated with higher risk.  6. **Diet**: A diet high in saturated fats and low in fruits and vegetables may increase risk.  7. **Smoking**: Smoking, especially long-term and heavy smoking, is linked to increased risk.  8. **Radiation Exposure**: Previous radiation therapy to the chest area, especially during adolescence, increases risk.  ### Other Factors:  1. **Pregnancy History**: Never having been pregnant or having a first pregnancy after age 30 increases risk.  2. **Breastfeeding**: Not breastfeeding or breastfeeding for a shorter duration may slightly increase risk.  3. **Environmental Factors**: Exposure to certain chemicals and pollutants may contribute to risk, though this is less well understood.  ### Summary:  While some risk factors like genetics and age cannot be changed, lifestyle modifications such as maintaining a healthy weight, limiting alcohol, staying physically active, and avoiding smoking can help reduce the risk of breast cancer. Regular screenings and discussions with healthcare providers are crucial for early detection and management. |  |
| 3 | How to prevent breast cancer? | Preventing breast cancer involves a combination of lifestyle changes, regular screenings, and awareness of risk factors. While not all cases of breast cancer can be prevented, the following strategies can help reduce your risk:  ### 1. **Maintain a Healthy Lifestyle**  - **Healthy Diet**: Focus on a diet rich in fruits, vegetables, whole grains, and lean proteins. Limit processed foods, red meat, and sugary drinks.  - **Regular Exercise**: Aim for at least 150 minutes of moderate-intensity exercise or 75 minutes of vigorous exercise per week.  - **Maintain a Healthy Weight**: Obesity, especially after menopause, increases the risk of breast cancer. Work toward a healthy weight through diet and exercise.  - **Limit Alcohol**: Alcohol consumption is linked to an increased risk of breast cancer. If you drink, limit it to no more than one drink per day.  ### 2. **Avoid Smoking**  - Smoking is associated with an increased risk of many cancers, including breast cancer. If you smoke, seek help to quit.  ### 3. **Breastfeed if Possible**  - Breastfeeding for several months may reduce the risk of breast cancer, especially if done for a year or more.  ### 4. **Be Aware of Hormone Therapy**  - Long-term use of hormone replacement therapy (HRT) during menopause can increase breast cancer risk. Discuss alternatives with your doctor if you’re considering HRT.  ### 5. **Know Your Family History**  - If you have a family history of breast cancer or carry genetic mutations (e.g., BRCA1 or BRCA2), consult a healthcare provider about additional preventive measures, such as:  - **Genetic Testing**: To assess your risk.  - **Preventive Medications**: Such as tamoxifen or raloxifene for high-risk individuals.  - **Prophylactic Surgery**: In extreme cases, some women opt for preventive mastectomy.  ### 6. **Regular Screenings**  - **Mammograms**: Follow recommended guidelines for mammograms based on your age and risk factors. Early detection can improve outcomes.  - **Clinical Breast Exams**: Have regular check-ups with your healthcare provider.  - **Self-Exams**: Be familiar with how your breasts look and feel, and report any changes (e.g., lumps, pain, or discharge) to your doctor.  ### 7. **Limit Exposure to Radiation and Environmental Toxins**  - Minimize unnecessary exposure to radiation (e.g., medical imaging) and avoid environmental pollutants linked to cancer.  ### 8. **Stay Informed**  - Keep up-to-date with the latest research and recommendations on breast cancer prevention.  ### 9. **Manage Stress**  - While stress itself is not directly linked to breast cancer, chronic stress can weaken the immune system. Practice stress-reducing techniques like yoga, meditation, or mindfulness.  ### 10. **Consider Vaccinations**  - Some studies suggest a link between certain viruses and breast cancer. Stay up-to-date on vaccinations, such as the HPV vaccine, which may indirectly reduce cancer risks.  ### When to Consult a Doctor  If you have a family history of breast cancer or other risk factors, talk to your doctor about personalized prevention strategies. They may recommend more frequent screenings or other interventions.  While these steps can reduce your risk, they cannot guarantee prevention. Regular check-ups and early detection remain critical for managing breast cancer effectively. |  |
|  | Repeat | Preventing breast cancer involves a combination of lifestyle changes, regular screenings, and awareness of risk factors. While not all cases of breast cancer can be prevented, the following strategies can help reduce your risk:  ### 1. **Maintain a Healthy Weight**  - Obesity, especially after menopause, increases the risk of breast cancer. Aim for a healthy weight through a balanced diet and regular exercise.  ### 2. **Exercise Regularly**  - Engage in at least 150 minutes of moderate-intensity or 75 minutes of high-intensity exercise per week. Physical activity helps regulate hormones and boosts the immune system.  ### 3. **Limit Alcohol Consumption**  - Alcohol is linked to an increased risk of breast cancer. If you drink, limit yourself to no more than one drink per day.  ### 4. **Eat a Healthy Diet**  - Focus on a diet rich in fruits, vegetables, whole grains, and lean proteins. Limit processed foods, red meat, and sugary drinks.  - Some studies suggest that a Mediterranean diet may reduce breast cancer risk.  ### 5. **Avoid Smoking**  - Smoking is associated with many cancers, including breast cancer. If you smoke, seek help to quit.  ### 6. **Breastfeed if Possible**  - Breastfeeding for at least several months may reduce the risk of breast cancer, especially if done for a year or more.  ### 7. **Limit Hormone Therapy**  - Long-term use of hormone replacement therapy (HRT) during menopause can increase breast cancer risk. Discuss alternatives with your doctor.  ### 8. **Be Aware of Family History**  - If you have a family history of breast cancer or carry genetic mutations (e.g., BRCA1 or BRCA2), consult a doctor about preventive measures, such as increased screening or prophylactic medications.  ### 9. **Regular Screenings**  - Follow recommended guidelines for mammograms and clinical breast exams. Early detection can significantly improve outcomes.  - Women at average risk should typically start mammograms at age 40–50, depending on guidelines.  ### 10. **Self-Exams**  - Perform monthly breast self-exams to become familiar with how your breasts normally look and feel. Report any changes to your doctor.  ### 11. **Reduce Exposure to Environmental Toxins**  - Limit exposure to radiation and environmental pollutants, such as certain chemicals found in plastics and pesticides.  ### 12. **Consider Preventive Medications or Surgery (for High-Risk Individuals)**  - Women at very high risk may consider medications like tamoxifen or raloxifene, or even preventive mastectomy, after consulting with a healthcare provider.  ### 13. **Manage Stress**  - Chronic stress may weaken the immune system. Practice stress-reducing techniques like yoga, meditation, or mindfulness.  ### 14. **Get Enough Sleep**  - Poor sleep patterns may disrupt hormone levels. Aim for 7–9 hours of quality sleep per night.  ### 15. **Stay Informed**  - Keep up-to-date with the latest research and recommendations on breast cancer prevention.  ### Important Notes:  - **Risk factors you can't control:** Age, gender, family history, and genetics play a role in breast cancer risk. Even if you follow all preventive measures, you may still develop breast cancer.  - **Consult a doctor:** Always discuss your personal risk factors and prevention strategies with a healthcare professional.  By adopting these healthy habits and staying proactive about your health, you can lower your risk of developing breast cancer. |  |
| 4 | Can breast cancer be inherited? | Yes, breast cancer can be inherited, but it's important to understand that only a small percentage of breast cancer cases are directly linked to inherited genetic mutations. The majority of breast cancers are considered sporadic, meaning they occur by chance without a clear hereditary cause.  ### Inherited Breast Cancer  Inherited breast cancer is primarily associated with mutations in specific genes, the most well-known being **BRCA1** and **BRCA2**. These genes normally help suppress tumor growth by repairing DNA damage, but mutations in these genes can increase the risk of developing breast cancer, ovarian cancer, and other cancers.  - **BRCA1 and BRCA2 mutations**: Women with a BRCA1 or BRCA2 mutation have a significantly higher lifetime risk of developing breast cancer (up to 70-80% for BRCA1 and 45-70% for BRCA2) compared to the general population.  - Other genes, such as **PALB2**, **TP53**, **PTEN**, **CHEK2**, and **ATM**, are also associated with an increased risk of breast cancer, though they are less common.  ### Family History and Risk  A strong family history of breast or ovarian cancer can indicate a higher likelihood of an inherited genetic mutation. Red flags include:  - Multiple relatives with breast or ovarian cancer.  - Breast cancer diagnosed at a young age (under 50).  - Cases of male breast cancer in the family.  - Ashkenazi Jewish ancestry (due to a higher prevalence of BRCA mutations).  ### Genetic Testing  If there is a concern about inherited breast cancer, genetic testing can be performed to identify mutations in BRCA1, BRCA2, or other genes. This testing is typically recommended for individuals with a strong family history of breast or ovarian cancer.  ### Prevention and Management  For those with inherited mutations, risk-reducing strategies may include:  - Increased surveillance (e.g., more frequent mammograms, MRIs).  - Chemoprevention (medications to reduce risk).  - Prophylactic surgeries (e.g., mastectomy, oophorectomy).  If you have concerns about inherited breast cancer, consult a genetic counselor or healthcare provider to discuss your family history and potential need for genetic testing. |  |
|  | Repeat | Yes, breast cancer can be inherited, but it's important to understand that only a small percentage of breast cancer cases are directly linked to inherited genetic mutations. The majority of breast cancers are considered sporadic, meaning they occur by chance without a clear hereditary cause.  ### Inherited Breast Cancer  Inherited breast cancer is primarily associated with mutations in specific genes, the most well-known being **BRCA1** and **BRCA2**. These genes normally help suppress tumor growth by repairing DNA damage, but mutations in these genes can increase the risk of breast and ovarian cancers.  - **BRCA1 and BRCA2 mutations**: Women with these mutations have a significantly higher lifetime risk of developing breast cancer (up to 70% or more) compared to the general population.  - Other genes, such as **TP53**, **PTEN**, **PALB2**, **CHEK2**, and **ATM**, can also increase the risk of breast cancer when mutated.  ### Family History  A strong family history of breast or ovarian cancer can suggest an inherited predisposition. Red flags include:  - Multiple relatives with breast or ovarian cancer.  - Breast cancer diagnosed at a young age (under 50).  - Cases of male breast cancer in the family.  - Ashkenazi Jewish ancestry (higher prevalence of BRCA mutations).  ### Genetic Testing  If there is a concern about inherited breast cancer, genetic testing can identify mutations in high-risk genes. This information can guide screening, prevention, and treatment decisions.  ### Prevention and Management  For individuals with inherited mutations, proactive measures may include:  - Increased surveillance (e.g., more frequent mammograms, MRIs).  - Risk-reducing medications (e.g., tamoxifen).  - Preventive surgeries (e.g., mastectomy, oophorectomy).  While inherited mutations play a role in some cases, most breast cancers are influenced by a combination of genetic, environmental, and lifestyle factors. If you have a family history of breast cancer, consult a healthcare provider or genetic counselor to assess your risk. |  |
| 5 | Why is breast cancer screening important? | Breast cancer screening is crucial for several reasons:  1. **Early Detection**: Screening can detect breast cancer at an early stage, often before any symptoms are noticeable. Early detection is key to successful treatment and can significantly improve the prognosis.  2. **Improved Survival Rates**: When breast cancer is found early, the chances of successful treatment and survival are much higher. Early-stage cancers are generally easier to treat and may require less aggressive therapy.  3. **More Treatment Options**: Early detection often means more treatment options are available. These options may include less invasive surgeries, targeted therapies, and a better overall response to treatment.  4. **Reduced Mortality**: Regular screening has been shown to reduce the mortality rate from breast cancer. By catching the disease early, the risk of it spreading to other parts of the body is minimized.  5. **Monitoring High-Risk Individuals**: For women with a family history of breast cancer or other risk factors, regular screening is essential for ongoing monitoring and early intervention if necessary.  6. **Peace of Mind**: Regular screenings can provide peace of mind for women, knowing that they are taking proactive steps to monitor their health.  7. **Public Health Impact**: On a broader scale, widespread screening programs contribute to public health by reducing the overall burden of breast cancer through early detection and treatment.  Common screening methods include mammograms, clinical breast exams, and self-exams. The specific recommendations for screening can vary based on age, risk factors, and guidelines from health organizations. It's important for individuals to discuss their personal risk factors and screening options with their healthcare provider to determine the best approach for their situation. |  |
|  | Repeat | Breast cancer screening is crucial for several reasons:  1. **Early Detection**: Screening can detect breast cancer at an early stage, often before any symptoms are noticeable. Early detection is key to successful treatment and can significantly improve the prognosis.  2. **Improved Survival Rates**: When breast cancer is found early, the chances of successful treatment and survival are much higher. Early-stage cancers are generally easier to treat and may require less aggressive therapy.  3. **More Treatment Options**: Early detection often means more treatment options are available. These options may include less invasive surgeries, targeted therapies, and the possibility of avoiding chemotherapy in some cases.  4. **Reduced Mortality**: Regular screening has been shown to reduce the mortality rate from breast cancer. Mammograms, for example, can identify tumors that are too small to be felt, leading to earlier intervention.  5. **Monitoring High-Risk Individuals**: For women with a family history of breast cancer or other risk factors, regular screening is essential for monitoring their health and catching any developments early.  6. **Peace of Mind**: Regular screenings can provide peace of mind for women, knowing that they are taking proactive steps to monitor their health.  7. **Public Health Impact**: On a broader scale, widespread screening can lead to better public health outcomes by reducing the overall incidence of advanced breast cancer and the associated healthcare costs.  ### Common Screening Methods:  - **Mammography**: The most common screening tool, which uses X-rays to create images of the breast.  - **Clinical Breast Exam (CBE)**: A physical examination performed by a healthcare provider.  - **Breast Self-Exams (BSE)**: Regular self-examinations to check for any changes or lumps.  - **MRI**: Used for women at high risk of breast cancer, often in conjunction with mammography.  ### Guidelines:  Screening guidelines can vary based on age, risk factors, and medical history. It's important for women to discuss their individual risk factors with their healthcare provider to determine the most appropriate screening schedule.  In summary, breast cancer screening is a vital tool in the early detection and effective treatment of breast cancer, ultimately saving lives and improving the quality of life for many women. |  |
| 6 | What are the risks of breast cancer screening? | Breast cancer screening, primarily through mammography, is a valuable tool for early detection of breast cancer, which can significantly improve treatment outcomes. However, like any medical procedure, it carries certain risks and limitations. Here are some of the key risks associated with breast cancer screening:  1. **False Positives**:  - A false positive occurs when a screening test suggests the presence of cancer when there is none. This can lead to additional tests, such as biopsies, and unnecessary anxiety and stress for the patient.  2. **False Negatives**:  - A false negative occurs when the screening test fails to detect cancer that is actually present. This can lead to a false sense of security and delay in diagnosis and treatment.  3. **Overdiagnosis and Overtreatment**:  - Screening can detect cancers that are slow-growing or would never cause symptoms or death during a person's lifetime. This can lead to overdiagnosis, where women are treated for cancers that would not have harmed them, exposing them to the side effects and risks of treatments like surgery, radiation, and chemotherapy.  4. **Radiation Exposure**:  - Mammograms involve exposure to low doses of radiation. While the risk of radiation-induced cancer from mammography is very low, repeated exposure over many years can potentially increase the risk of cancer.  5. **Psychological Stress**:  - The process of screening and the possibility of false positives can cause significant anxiety and stress. The waiting period for further tests or results can be emotionally taxing.  6. **Physical Discomfort**:  - Mammograms can be uncomfortable or even painful for some women due to the compression of the breast tissue required to obtain clear images.  7. **Financial Costs**:  - Screening tests and follow-up procedures can be costly, and not all costs may be covered by insurance, leading to financial burden for some individuals.  8. **Limited Benefit for Certain Groups**:  - The benefits of screening may be limited for certain groups, such as women with a low risk of breast cancer or those with a limited life expectancy due to age or other health conditions. For these groups, the risks may outweigh the benefits.  It's important for individuals to discuss the potential risks and benefits of breast cancer screening with their healthcare provider to make an informed decision based on their personal health history and risk factors. |  |
|  | Repeat | Breast cancer screening, primarily through mammography, is a valuable tool for early detection of breast cancer, which can significantly improve treatment outcomes. However, like any medical procedure, it carries certain risks and limitations. Here are some of the key risks associated with breast cancer screening:  1. **False Positives**:  - A false positive occurs when a screening test suggests the presence of cancer when there is none. This can lead to additional tests, such as more mammograms, ultrasounds, or biopsies, which can cause anxiety, discomfort, and additional costs.  2. **Overdiagnosis and Overtreatment**:  - Overdiagnosis happens when screening detects cancers that would never have caused symptoms or become life-threatening. This can lead to overtreatment, including surgeries, radiation, and chemotherapy, which may not have been necessary and can have significant side effects.  3. **False Negatives**:  - A false negative occurs when a screening test fails to detect cancer that is present. This can provide false reassurance and delay diagnosis and treatment.  4. **Radiation Exposure**:  - Mammograms involve exposure to low levels of radiation. While the risk from this exposure is small, repeated screenings over time can potentially increase the risk of developing cancer, although this risk is generally considered to be very low compared to the benefits of early detection.  5. **Anxiety and Stress**:  - The screening process, including waiting for results and dealing with false positives, can cause significant anxiety and stress for some women.  6. **Physical Discomfort**:  - Mammograms can be uncomfortable or even painful for some women due to the compression of the breast required to obtain clear images.  7. **Financial Costs**:  - Screening tests and any follow-up procedures can be costly, and not all costs may be covered by insurance, leading to financial strain for some individuals.  8. **Limited Benefit for Certain Groups**:  - The benefits of screening may be limited for certain groups, such as older women or those with a short life expectancy, where the risks of screening may outweigh the potential benefits.  It's important for individuals to discuss these risks and benefits with their healthcare provider to make an informed decision about breast cancer screening based on their personal health history, risk factors, and preferences. |  |
| 7 | What are the common screening methods for breast cancer? | Breast cancer screening aims to detect cancer early, often before symptoms appear. Common screening methods include:  1. **Mammography**:  - **Standard Mammogram**: Uses X-rays to create images of the breast. It is the most common screening tool and can detect tumors that are too small to feel.  - **Digital Mammogram**: Converts X-rays into digital images, allowing for enhanced visualization and storage.  - **3D Mammography (Tomosynthesis)**: Creates a 3D image of the breast, improving detection rates and reducing false positives.  2. **Clinical Breast Exam (CBE)**:  - A physical examination performed by a healthcare provider to check for lumps or other changes in the breast.  3. **Breast Self-Exam (BSE)**:  - Individuals check their own breasts for lumps or changes. While not a substitute for clinical exams or mammograms, it helps with awareness.  4. **Breast Ultrasound**:  - Uses sound waves to produce images of the breast. It is often used as a supplementary tool, especially for dense breast tissue.  5. **Magnetic Resonance Imaging (MRI)**:  - Uses magnetic fields and radio waves to create detailed images. It is recommended for high-risk individuals, such as those with a strong family history or genetic mutations (e.g., BRCA1/BRCA2).  6. **Genetic Testing**:  - Identifies mutations in genes like BRCA1 and BRCA2, which increase breast cancer risk. This is not a routine screening tool but is used for individuals with a family history of breast or ovarian cancer.  7. **Thermography**:  - Detects heat patterns and blood flow in the breast. However, it is not widely recommended as a standalone screening tool due to limited evidence of effectiveness.  8. **Ductal Lavage**:  - A minimally invasive procedure to collect cells from the milk ducts, where some breast cancers originate. It is not commonly used for routine screening.  ### Screening Guidelines:  - **Average-Risk Women**: Mammograms every 1-2 years starting at age 40-50, depending on guidelines from organizations like the American Cancer Society (ACS) or U.S. Preventive Services Task Force (USPSTF).  - **High-Risk Women**: Earlier and more frequent screening, often including MRI in addition to mammography.  Consult a healthcare provider to determine the best screening plan based on individual risk factors. |  |
|  | Repeat | Common screening methods for breast cancer include:  1. **Mammography**: The most widely used screening tool, mammograms are X-ray images of the breast that can detect tumors too small to be felt. Regular mammograms are recommended for women over 40 or 50, depending on guidelines.  2. **Clinical Breast Exam (CBE)**: A physical examination performed by a healthcare provider to check for lumps or other changes in the breast.  3. **Breast Self-Exams (BSE)**: Regular self-checks to familiarize oneself with the normal look and feel of the breasts, helping to identify any unusual changes.  4. **Magnetic Resonance Imaging (MRI)**: Used for women at high risk of breast cancer (e.g., those with BRCA mutations or strong family history). MRI is often used in addition to mammography.  5. **Ultrasound**: Used to further evaluate abnormalities found during a mammogram or physical exam, especially in dense breast tissue.  6. **Digital Breast Tomosynthesis (3D Mammography)**: An advanced form of mammography that creates a 3D image of the breast, improving detection in some cases.  7. **Genetic Testing**: For individuals with a family history of breast cancer, genetic testing can identify mutations in genes like BRCA1 and BRCA2, which increase cancer risk.  Screening recommendations vary based on age, risk factors, and guidelines from health organizations. Always consult a healthcare provider for personalized advice. |  |
| 8 | What is the survival rate of breast cancer? | The survival rate for breast cancer varies significantly based on several factors, including the stage at diagnosis, the type of breast cancer, the patient's age, overall health, and the treatments available. Here are some general statistics based on stages:  1. **Stage 0 (DCIS - Ductal Carcinoma In Situ):** Nearly 100% survival rate at 5 years, as this is a non-invasive cancer.  2. **Stage I:** The 5-year relative survival rate is about 98-100%.  3. **Stage II:** The 5-year relative survival rate is approximately 90-93%.  4. **Stage III:** The 5-year relative survival rate is around 72%.  5. **Stage IV (Metastatic Breast Cancer):** The 5-year relative survival rate drops to about 22%.  These statistics are based on data from large populations and can vary for individual cases. Advances in treatment and early detection continue to improve survival rates over time. Always consult with healthcare professionals for the most accurate and personalized information. |  |
|  | Repeat | The survival rate for breast cancer varies depending on several factors, including the stage at diagnosis, the type of breast cancer, the patient's age, overall health, and the treatments received. Here are some general statistics based on the stage of breast cancer:  1. **Stage 0 (DCIS - Ductal Carcinoma In Situ):** The 5-year survival rate is close to 100% because this is a non-invasive cancer that has not spread beyond the milk ducts.  2. **Stage I:** The 5-year survival rate is also close to 100%. At this stage, the cancer is small and has not spread to the lymph nodes.  3. **Stage II:** The 5-year survival rate is about 93%. This stage includes larger tumors or cancer that has spread to a few nearby lymph nodes.  4. **Stage III:** The 5-year survival rate is about 72%. This stage includes larger tumors that may have spread to several lymph nodes or nearby tissues.  5. **Stage IV (Metastatic Breast Cancer):** The 5-year survival rate is about 22%. At this stage, the cancer has spread to distant organs such as the bones, liver, lungs, or brain.  It's important to note that these statistics are based on historical data and may not reflect the outcomes of patients who are diagnosed and treated today. Advances in early detection, personalized treatment, and targeted therapies continue to improve survival rates.  Always consult with healthcare professionals for the most accurate and personalized information regarding breast cancer prognosis and treatment options. |  |
| 9 | What are the treatment methods of breast cancer? | Breast cancer treatment depends on the cancer type, stage, patient health, and preferences. Common methods include:  ### 1. **Surgery**  - **Lumpectomy**: Removes the tumor and some surrounding tissue.  - **Mastectomy**: Removes the entire breast, sometimes including nearby tissues.  - **Sentinel Lymph Node Biopsy**: Removes a few lymph nodes to check for cancer spread.  - **Axillary Lymph Node Dissection**: Removes more lymph nodes if cancer is detected.  ### 2. **Radiation Therapy**  - Uses high-energy rays to kill cancer cells, often after surgery to eliminate remaining cells.  ### 3. **Chemotherapy**  - Uses drugs to kill or slow cancer cells, administered orally or intravenously, often before or after surgery.  ### 4. **Hormone Therapy**  - Blocks hormones that fuel certain breast cancers, using drugs like tamoxifen or aromatase inhibitors.  ### 5. **Targeted Therapy**  - Targets specific cancer cell molecules, such as HER2 inhibitors for HER2-positive breast cancer.  ### 6. **Immunotherapy**  - Boosts the immune system to fight cancer, used for certain types like triple-negative breast cancer.  ### 7. **Combination Therapy**  - Often combines treatments like surgery with radiation or chemotherapy for better outcomes.  ### 8. **Palliative Care**  - Focuses on symptom relief and improving quality of life, used alongside other treatments.  ### Factors Influencing Treatment:  - Cancer stage and type  - Hormone receptor and HER2 status  - Patient age and overall health  - Personal preferences  ### Follow-Up Care:  - Regular check-ups, imaging, and tests to monitor recovery and detect recurrence.  Always consult a healthcare provider for a personalized treatment plan. |  |
|  | Repeat | Breast cancer treatment depends on the cancer type, stage, patient health, and preferences. Common methods include:  ### 1. **Surgery**  - **Lumpectomy**: Removes the tumor and some surrounding tissue.  - **Mastectomy**: Removes the entire breast, sometimes including nearby tissues.  - **Sentinel Lymph Node Biopsy**: Removes a few lymph nodes to check for cancer spread.  - **Axillary Lymph Node Dissection**: Removes multiple lymph nodes if cancer is detected.  ### 2. **Radiation Therapy**  - Uses high-energy rays to kill cancer cells, often after surgery to eliminate remaining cells.  ### 3. **Chemotherapy**  - Uses drugs to kill or slow cancer cells, administered orally or intravenously, often before or after surgery.  ### 4. **Hormone Therapy**  - Blocks hormones that fuel certain breast cancers, using drugs like tamoxifen or aromatase inhibitors.  ### 5. **Targeted Therapy**  - Targets specific cancer cell molecules, such as HER2 inhibitors for HER2-positive breast cancer.  ### 6. **Immunotherapy**  - Boosts the immune system to fight cancer, used for certain types like triple-negative breast cancer.  ### 7. **Combination Therapy**  - Uses multiple treatments (e.g., surgery with chemotherapy or radiation) for better outcomes.  ### 8. **Palliative Care**  - Focuses on symptom relief and improving quality of life, often used in advanced stages.  ### Factors Influencing Treatment:  - **Cancer Stage**: Early-stage may require less aggressive treatment.  - **Tumor Characteristics**: Hormone receptor status and HER2 levels guide therapy.  - **Patient Health**: Overall health and preferences impact treatment choice.  ### Follow-Up Care:  - Regular monitoring is essential to detect recurrence and manage side effects.  Treatment plans are personalized, so patients should discuss options with their healthcare team. |  |
| 10 | Can breast cancer recur? | Yes, breast cancer can recur. Recurrence means that the cancer has returned after a period of time during which it could not be detected. Breast cancer recurrence can happen in several ways:  1. **Local Recurrence**: This occurs when cancer comes back in the same place as the original tumor, such as in the remaining breast tissue or the chest wall after a mastectomy.  2. **Regional Recurrence**: This happens when cancer returns in the nearby lymph nodes or tissues close to the original cancer site.  3. **Distant Recurrence (Metastatic)**: This is when cancer spreads to distant parts of the body, such as the bones, liver, lungs, or brain. This is also known as metastatic breast cancer.  The risk of recurrence depends on various factors, including the stage and type of the original cancer, the treatments received, and individual health factors. Regular follow-up care, including physical exams, imaging tests, and sometimes blood tests, is important for monitoring for signs of recurrence. Early detection of recurrence can improve the chances of successful treatment. |  |
|  | Repeat | Yes, breast cancer can recur. Recurrence happens when cancer cells that were not eliminated by initial treatment survive and later develop into a new tumor. Breast cancer recurrence can be categorized into several types:  1. **Local Recurrence**: Cancer reappears in the same area where it was first diagnosed, such as the breast or chest wall.  2. **Regional Recurrence**: Cancer returns in nearby lymph nodes or tissues.  3. **Distant Recurrence (Metastatic)**: Cancer spreads to distant parts of the body, such as the bones, liver, lungs, or brain.  The risk of recurrence depends on factors like the stage and type of the original cancer, the treatments received, and individual health factors. Regular follow-up care, including screenings and monitoring, is essential to detect and manage recurrence early. Advances in treatment have improved outcomes for many patients with recurrent breast cancer. |  |
