# Supplementary material for: ChatGPT Versus DeepSeek for Breast Cancer Information Retrieval: Quantitative Comparative Study
Source: JMIR Cancer. 2026 Feb 27;12:e72839. doi: 10.2196/72839 (PMC12954694; doi:10.2196/72839)
Supplement: Multimedia Appendix 3 — Frequently asked questions about breast cancer and answers generated by artificial intelligence models (ChatGPT and DeepSeek) by Researcher 3. [file cancer-v12-e72839-s003.docx]

**Supporting Information: Supplementary Table 3**

**ChatGPT vs. DeepSeek: A Comparative Analysis of AI Models for Breast Cancer Information Retrieval**

Rima Hajjo^1,2*^, Dima A. Sabbah^1^, Sanaa K. Bardaweel^3^

*^1^Department of Pharmacy, Faculty of Pharmacy, Al-Zaytoonah University of Jordan, P.O. Box 130, Amman 11733, Jordan.*

*^2^Laboratory for Molecular Modeling, Division of Chemical Biology and Medicinal Chemistry, Eshelman School of Pharmacy, The University of North Carolina at Chapel Hill, Chapel Hill, NC, USA.*

*^3^Department of Pharmaceutical Sciences, School of Pharmacy, University of Jordan, Amman 11942, Jordan.*

*Correspondence

Rima Hajjo

E-mail: rhajjo@gmail.com; [r.hajjo@zuj.edu.jo](mailto:r.hajjo@zuj.edu.jo)

ORCID: https://orcid.org/0000-0002-7090-5425

**Supplementary Table 3.** Frequently asked questions about breast cancer and answers generated by artificial intelligence models (ChatGPT and DeepSeek) by Researcher 3.

| **ChatGPT 4.0** | | | |
| --- | --- | --- | --- |
|  | **Question** | **Answer** | |
| 1 | What are the common symptoms of breast cancer? | The common symptoms of breast cancer include:   1. **Lump or Mass** – A new lump in the breast or underarm that feels different from surrounding tissue. 2. **Changes in Breast Size or Shape** – Unexplained swelling, shrinkage, or asymmetry. 3. **Skin Changes** – Redness, dimpling (peau d'orange), or thickening of the breast skin. 4. **Nipple Changes** – Retraction (pulling inward), discharge (especially bloody or clear), or scaling/crusting. 5. **Persistent Breast Pain** – Not related to the menstrual cycle. 6. **Swelling in the Armpit or Collarbone Area** – May indicate lymph node involvement.   If any of these symptoms persist, it’s important to consult a healthcare professional for further evaluation. | |
|  | Repeat | Common symptoms of breast cancer include:   1. **Lump in the Breast or Underarm** – A new lump or mass that feels different from surrounding tissue. 2. **Change in Breast Size or Shape** – Unexplained swelling, shrinkage, or asymmetry. 3. **Skin Changes** – Dimpling, redness, or puckering of the skin (resembling an orange peel). 4. **Nipple Changes** – Inversion, retraction, or discharge (other than breast milk), sometimes bloody. 5. **Breast Pain or Tenderness** – Though breast cancer is often painless, some cases may cause discomfort. 6. **Swelling in the Armpit or Collarbone Area** – Could indicate lymph node involvement. 7. **Persistent Itching or Rash** – Particularly around the nipple, possibly linked to rare forms like Paget’s disease. 8. **Changes in Skin Texture** – Thickening of breast tissue or peeling of nipple skin.   If you notice any of these symptoms, consult a healthcare professional for further evaluation. Early detection improves treatment outcomes. | |
| 2 | What are the risk factors for breast cancer? | The risk factors for breast cancer can be categorized into **non-modifiable** and **modifiable** factors:  **Non-Modifiable Risk Factors:**   1. **Gender** – Women are at a much higher risk than men. 2. **Age** – Risk increases with age, especially after 50. 3. **Family History & Genetics** – Having close relatives with breast cancer, especially mutations in **BRCA1** or **BRCA2** genes. 4. **Personal History of Breast Cancer** – Previous breast cancer increases the risk of recurrence. 5. **Dense Breast Tissue** – Makes it harder to detect cancer on mammograms. 6. **Early Menstruation (Before Age 12) or Late Menopause (After 55)** – Prolonged estrogen exposure increases risk. 7. **Previous Radiation Therapy to the Chest** – Especially during childhood or young adulthood.   **Modifiable Risk Factors:**   1. **Hormone Replacement Therapy (HRT)** – Long-term use of estrogen and progesterone. 2. **Alcohol Consumption** – Higher alcohol intake increases risk. 3. **Obesity** – Especially after menopause due to increased estrogen production in fat tissue. 4. **Lack of Physical Activity** – A sedentary lifestyle contributes to risk. 5. **Poor Diet** – High in processed foods and unhealthy fats. 6. **Reproductive Factors** – Having first pregnancy after 30, never having children, or not breastfeeding.   **Emerging Risk Factors:**   - **Exposure to Environmental Toxins** (e.g., endocrine disruptors). - **Chronic Stress & Poor Sleep Patterns** (linked to hormonal imbalances).   Although some risk factors are beyond control, adopting a healthy lifestyle can help reduce the overall risk of breast cancer. | |
|  | Repeat | Several factors can increase the risk of developing breast cancer. These include:  **1. Biological and Genetic Factors**   - **Gender** – Women are at higher risk than men. - **Age** – Risk increases with age, especially after 50. - **Family History** – Having a close relative (mother, sister, or daughter) with breast cancer. - **Genetic Mutations** – BRCA1, BRCA2, and other gene mutations significantly increase risk. - **Personal History of Breast Cancer** – Previous breast cancer increases the chance of recurrence. - **Dense Breast Tissue** – Makes detection harder and is linked to higher risk.   **2. Hormonal and Reproductive Factors**   - **Early Menstruation (Before 12) or Late Menopause (After 55)** – Longer exposure to estrogen. - **Late or No Pregnancy** – Women who have their first child after 30 or never give birth have a higher risk. - **Hormone Replacement Therapy (HRT)** – Long-term use of estrogen and progesterone therapy increases risk. - **Birth Control Pills** – Some studies suggest a slight increase in risk, but it decreases after stopping use.   **3. Lifestyle Factors**   - **Alcohol Consumption** – Even moderate drinking can raise risk. - **Obesity** – Especially after menopause, due to increased estrogen production. - **Lack of Physical Activity** – Regular exercise helps lower risk. - **Poor Diet** – High-fat diets and processed foods may contribute. - **Radiation Exposure** – Previous radiation therapy to the chest (e.g., for Hodgkin’s lymphoma).   **4. Environmental and Other Factors**   - **Exposure to Chemicals** – Some endocrine disruptors (like BPA and pesticides) may contribute. - **Night Shift Work** – Some research suggests that disrupted circadian rhythms may increase risk.   While some risk factors (like genetics) can’t be changed, lifestyle modifications can help lower overall risk. If concerned, regular screenings and doctor consultations are key for early detection. | |
| 3 | How to prevent breast cancer? | Preventing breast cancer involves a combination of lifestyle choices, early detection strategies, and, for high-risk individuals, medical interventions. Here are some key approaches:  **1. Maintain a Healthy Lifestyle**   - **Healthy Diet:** Eat a balanced diet rich in fruits, vegetables, whole grains, and lean proteins. Limit processed foods, red meat, and saturated fats. - **Regular Exercise:** Engage in at least 150 minutes of moderate-intensity exercise (e.g., brisk walking) or 75 minutes of vigorous-intensity exercise (e.g., running) per week. - **Maintain a Healthy Weight:** Obesity, especially after menopause, increases the risk of breast cancer. - **Limit Alcohol Consumption:** Even small amounts of alcohol can increase breast cancer risk. Limit intake to no more than one drink per day. - **Avoid Smoking:** Smoking is linked to an increased risk of many cancers, including breast cancer.   **2. Hormonal and Reproductive Factors**   - **Breastfeed if Possible:** Breastfeeding has been shown to reduce the risk of breast cancer. - **Limit Hormone Therapy:** Long-term use of hormone replacement therapy (HRT), especially combined estrogen-progestin therapy, increases breast cancer risk. If needed, use the lowest dose for the shortest time possible. - **Consider Timing of Pregnancy:** Women who have their first full-term pregnancy at an earlier age and have multiple children may have a slightly lower risk.   **3. Regular Screening and Early Detection**   - **Self-Breast Exams:** Be familiar with your breasts' normal look and feel. Report any changes to a doctor. - **Clinical Breast Exams:** Have a doctor check your breasts regularly. - **Mammograms:** Women over 40 (or earlier if high risk) should have regular mammograms to detect cancer early.   **4. Reduce Environmental Exposures**   - **Limit Exposure to Radiation & Pollutants:** Avoid unnecessary medical imaging (like CT scans) and limit exposure to environmental toxins. - **Use Safe Personal Care Products:** Some cosmetics and plastics contain endocrine-disrupting chemicals that may impact breast cancer risk.   **5. Genetic and Medical Considerations**   - **Know Your Family History:** If you have a family history of breast cancer, consider genetic counseling and testing for BRCA1/BRCA2 mutations. - **Consider Preventive Medications:** For high-risk individuals, doctors may prescribe tamoxifen or raloxifene to reduce the risk. - **Preventive Surgery (in High-Risk Cases):** In extreme cases, women with BRCA mutations may consider preventive mastectomy or oophorectomy.   Would you like any specific details on one of these prevention strategies? | |
|  | Repeat | While there is no guaranteed way to prevent breast cancer, several lifestyle changes and medical interventions can help lower your risk. Here are some key strategies:  **1. Maintain a Healthy Lifestyle**  ✅ **Exercise Regularly** – Aim for at least **150 minutes of moderate** or **75 minutes of vigorous** physical activity per week. ✅ **Maintain a Healthy Weight** – Being overweight, especially after menopause, increases risk. ✅ **Eat a Balanced Diet** – Focus on **fruits, vegetables, whole grains, and lean proteins** while limiting processed foods and red meat. ✅ **Limit Alcohol Consumption** – No more than **one drink per day** (or none) is best. ✅ **Avoid Smoking** – Smoking is linked to an increased risk of breast and other cancers.  **2. Be Mindful of Hormones**  🔸 **Limit Hormone Replacement Therapy (HRT)** – If necessary, use the lowest dose for the shortest time. 🔸 **Use Birth Control Wisely** – Oral contraceptives may slightly increase risk, but risk decreases after stopping.  **3. Breastfeeding (if possible)**  🔹 Breastfeeding for several months or longer can slightly reduce breast cancer risk.  **4. Regular Screening & Early Detection**  🔹 **Perform Monthly Breast Self-Exams** – Be familiar with how your breasts normally look and feel. 🔹 **Get Regular Mammograms** – Follow screening guidelines based on your age and risk level:   - **Ages 40-44**: Optional annual mammogram. - **Ages 45-54**: Annual mammogram recommended. - **Ages 55+**: Mammograms every 1-2 years, depending on risk factors. 🔹 **Consult a Doctor for Genetic Testing** – If you have a strong family history, testing for **BRCA1, BRCA2**, or other mutations may help with risk assessment.   **5. Reduce Environmental Exposures**  🛑 **Minimize Exposure to Radiation & Toxins** – Avoid unnecessary X-rays and limit exposure to endocrine-disrupting chemicals (like BPA and pesticides).  For those at **high risk**, doctors may recommend **preventive medications (like tamoxifen or raloxifene)** or, in extreme cases, **prophylactic mastectomy (preventive breast removal).**  **Bottom Line**  While some risk factors like genetics cannot be changed, adopting a healthy lifestyle, staying informed, and getting regular screenings can significantly **reduce** your risk of breast cancer. | |
| 4 | Can breast cancer be inherited? | Yes, breast cancer can be inherited, but most cases are not. Only about **5-10% of breast cancer cases** are linked to inherited gene mutations.  **1. Inherited Gene Mutations**  Certain gene mutations can increase the risk of breast cancer. The most well-known are:   - **BRCA1 and BRCA2 mutations**: These significantly increase the risk of breast and ovarian cancer. Women with BRCA1 mutations have a **55-72% lifetime risk**, while those with BRCA2 mutations have a **45-69% lifetime risk** of developing breast cancer. - **Other genetic mutations**: Less common mutations in genes like **TP53, PTEN, PALB2, CHEK2, and ATM** also contribute to inherited breast cancer risk.   **2. Family History & Risk**  You may have a higher risk of inherited breast cancer if:   - A **first-degree relative** (mother, sister, or daughter) has had breast cancer, especially before age 50. - Multiple family members on **either side** (mother’s or father’s family) have had breast or ovarian cancer. - A male relative has had breast cancer. - There is a family history of **triple-negative breast cancer** or **bilateral breast cancer** (cancer in both breasts). - A family member has tested **positive for a BRCA1/BRCA2 mutation**.   **3. Genetic Testing & Counseling**   - If you have a strong family history, you can consider **genetic testing** to check for mutations in BRCA1, BRCA2, and other related genes. - **Genetic counseling** can help you understand your risk and guide prevention strategies.   **4. Preventive Measures for High-Risk Individuals**   - **Regular screening**: More frequent mammograms and MRI scans. - **Preventive medication**: Tamoxifen or raloxifene can reduce risk. - **Lifestyle changes**: A healthy diet, regular exercise, and avoiding smoking and alcohol can help. - **Preventive surgery**: Some high-risk individuals choose a prophylactic mastectomy (removal of breasts) or oophorectomy (removal of ovaries) to reduce risk.   Would you like guidance on genetic testing or risk reduction strategies? | |
|  | Repeat | Yes, breast cancer can be **inherited**, but only about **5–10% of cases** are directly linked to genetic mutations passed down in families.  **How is Breast Cancer Inherited?**  Inherited breast cancer is mainly associated with mutations in the **BRCA1** and **BRCA2** genes, which normally help repair damaged DNA. Mutations in these genes increase the risk of breast, ovarian, and other cancers.   - **BRCA1 Mutation** – Raises lifetime breast cancer risk to **55–72%**. - **BRCA2 Mutation** – Raises lifetime breast cancer risk to **45–69%**.   Other genes, such as **TP53, PTEN, PALB2, CHEK2, and ATM**, can also contribute to hereditary breast cancer but are less common.  **Who Should Consider Genetic Testing?**  You may want to consider genetic testing if you have: ✔ **A strong family history** of breast or ovarian cancer (especially in close relatives like mother, sister, or daughter). ✔ **Multiple family members** diagnosed with breast cancer at a young age (before 50). ✔ **Male breast cancer** in the family. ✔ **Ashkenazi Jewish ancestry**, as BRCA mutations are more common in this group. ✔ **A personal history** of breast cancer, especially triple-negative breast cancer.  **What Can You Do If You Have a Genetic Mutation?**  If you test positive for a BRCA or other high-risk mutation, your doctor may recommend: 🔹 **Increased Screening** – More frequent mammograms, MRIs, and clinical exams. 🔹 **Preventive Medications** – Such as **tamoxifen** or **raloxifene** to reduce risk. 🔹 **Risk-Reducing Surgery** – Preventive mastectomy (removal of breasts) or oophorectomy (removal of ovaries) may be considered in high-risk cases.  **Bottom Line**  While most breast cancer cases are **not inherited**, genetic mutations can significantly increase risk. If you have a family history of breast cancer, **talk to a doctor about genetic counseling and testing** to determine your risk and prevention options. | |
| 5 | Why is breast cancer screening important? | Breast cancer screening is essential because it **detects cancer early**, often before symptoms appear, improving the chances of successful treatment and survival. Here’s why screening matters:  **1. Early Detection Saves Lives**   - When breast cancer is found early (at **Stage 0 or I**), the **5-year survival rate is nearly 99%**. - Advanced-stage breast cancer (Stage IV) has a much lower survival rate. - Early detection allows for **less aggressive treatments** and better outcomes.   **2. Detects Cancer Before Symptoms Appear**   - Most early-stage breast cancers **don’t cause pain or noticeable lumps**. - Mammograms can detect tumors **years before they are felt**, enabling treatment at an earlier stage.   **3. Increases Treatment Options**   - Early-stage cancers can often be treated with **breast-conserving surgery (lumpectomy)** rather than a mastectomy. - Less aggressive treatments (like targeted therapy or hormone therapy) may be possible instead of chemotherapy.   **4. Reduces Breast Cancer Mortality**   - Studies show that regular screening reduces breast cancer deaths by **25-30%** in women aged 40-74.   **5. Identifies High-Risk Individuals**   - Screening also detects **precancerous conditions** (like ductal carcinoma in situ, DCIS), allowing preventive measures. - Women with dense breast tissue or genetic risks may need additional imaging (like MRI).   **Who Should Get Screened?**   - **Ages 40-49**: Screening is recommended based on individual risk factors (discuss with a doctor). - **Ages 50-74**: **Mammograms every 1-2 years** (as recommended by most guidelines). - **High-Risk Women (BRCA mutations, family history, dense breasts, etc.)**: May need **earlier and more frequent screening** with **MRI + mammograms**.   Would you like recommendations on a screening schedule based on risk factors? | |
|  | Repeat | Breast cancer screening is **crucial** because it helps detect cancer **early**, often before symptoms appear. Early detection increases the chances of **successful treatment** and improves survival rates.  **Key Benefits of Breast Cancer Screening**  ✅ **Detects Cancer Early** – When caught in its early stages, breast cancer is more treatable and less likely to spread.  ✅ **Increases Survival Rates** – The **5-year survival rate** for localized (early-stage) breast cancer is **99%**, compared to lower survival rates for advanced stages.  ✅ **Reduces the Need for Aggressive Treatment** – Early-stage cancer may require **less invasive treatments**, such as lumpectomy instead of mastectomy or less aggressive chemotherapy.  ✅ **Identifies Precancerous Changes** – Screening can detect **abnormal cells** (like ductal carcinoma in situ, DCIS) before they develop into invasive cancer.  ✅ **Provides Peace of Mind** – Regular screening can help reduce anxiety by confirming no signs of cancer or catching it early if present.  **Types of Breast Cancer Screening**  🔹 **Mammogram** – The **gold standard** for breast cancer screening; recommended annually or biennially depending on age and risk.  🔹 **Clinical Breast Exam (CBE)** – Performed by a doctor to check for lumps or abnormalities.  🔹 **Breast Self-Exam (BSE)** – While not a replacement for medical screening, it helps individuals become familiar with their breasts to detect changes.  🔹 **Breast MRI or Ultrasound** – Used for **high-risk women** or when mammograms need further evaluation.  **Who Should Get Screened?**  📌 **Ages 40-44** – Optional mammograms based on personal choice. 📌 **Ages 45-54** – Annual mammograms recommended. 📌 **Ages 55+** – Mammograms every **1-2 years**, depending on risk factors. 📌 **High-Risk Individuals** – May need earlier and more frequent screenings (e.g., starting at age 30 with MRI and mammograms).  **Bottom Line**  Breast cancer screening is a **lifesaving tool** that helps detect cancer **early**, when it’s most treatable. Regular screenings, combined with a healthy lifestyle and awareness of risk factors, can significantly improve **outcomes and survival rates**. | |
| 6 | What are the risks of breast cancer screening? | While breast cancer screening (especially mammograms) is crucial for early detection, it does come with some risks. Here are the main ones to consider:  **1. False Positives (Overdiagnosis)**   - A mammogram may detect something suspicious that **is not cancer**, leading to unnecessary stress and additional tests. - About **10% of women** undergoing mammography may get a false-positive result, especially younger women with dense breast tissue. - Follow-up procedures like **biopsies** can cause anxiety, discomfort, and added costs.   **2. Overdiagnosis & Overtreatment**   - Screening may detect **slow-growing or non-life-threatening cancers** (such as ductal carcinoma in situ, DCIS) that might never cause harm. - Some women undergo **surgery, radiation, or chemotherapy** for cancers that might not have progressed.   **3. Radiation Exposure**   - Mammograms use **low-dose X-rays**, and while the radiation exposure is minimal, repeated screening over decades carries a **very small risk** of contributing to cancer. - The benefits of early detection **far outweigh** this risk, but younger women (under 40) should avoid unnecessary mammograms unless at high risk.   **4. False Negatives (Missed Cancers)**   - Mammograms **miss about 10-15% of breast cancers**, especially in women with dense breasts. - Some aggressive cancers may develop between screenings (interval cancers).   **5. Psychological Stress**   - Waiting for results, undergoing additional testing, and potential false positives can cause anxiety and emotional distress.   **6. Pain or Discomfort**   - The breast compression during a mammogram can be **uncomfortable or mildly painful**, but it lasts only a few seconds.   **How to Minimize Risks?**   - **Personalized screening plans**: High-risk women may need MRI in addition to mammograms. - **Discuss screening frequency** with a doctor, especially for women over 40. - **Use advanced imaging techniques** (like 3D mammograms) for better accuracy.   Would you like recommendations on the best screening method based on risk factors? | |
|  | Repeat | While breast cancer screening has significant benefits, it also comes with some potential risks. It’s important to understand these risks to make an informed decision about screening.  **1. False Positives (Unnecessary Anxiety & Tests)**  🔹 A **false positive** occurs when a mammogram detects an abnormality that turns out **not to be cancer**. 🔹 This can lead to **unnecessary stress**, additional tests (like biopsies), and emotional distress. 🔹 False positives are more common in **younger women** and those with **dense breast tissue**.  **2. False Negatives (Missed Cancer Cases)**  🔹 A **false negative** occurs when a mammogram **fails to detect breast cancer**, delaying diagnosis. 🔹 This happens more often in women with **dense breast tissue**. 🔹 A missed diagnosis can lead to a **false sense of security** and delayed treatment.  **3. Overdiagnosis & Overtreatment**  🔹 Some detected cancers (e.g., **DCIS or very slow-growing tumors**) may **never cause harm** in a person’s lifetime. 🔹 These cases may lead to **unnecessary treatments** like surgery, radiation, or chemotherapy that could have been avoided. 🔹 Overdiagnosis is more common with **increased screening frequency** and **older age**.  **4. Exposure to Radiation**  🔹 Mammograms use **low-dose X-rays**, but repeated exposure over many years **slightly increases cancer risk**. 🔹 The radiation risk is **very low** compared to the benefit of early cancer detection.  **5. Unnecessary Biopsies & Procedures**  🔹 If an abnormality is found, further tests (like biopsies) may be needed. 🔹 While most biopsies turn out **benign (non-cancerous)**, the procedure can cause **pain, scarring, and emotional distress**.  **Should You Still Get Screened?**  For **most women**, the **benefits outweigh the risks**. Early detection improves survival rates and allows for less aggressive treatment. However, it's essential to discuss personal **risk factors, age, and preferences** with a doctor to determine the best screening schedule. | |
| 7 | What are the common screening methods for breast cancer? | Breast cancer screening involves different imaging techniques and clinical evaluations to detect cancer early, often before symptoms appear. Here are the most commonly used screening methods:  **1. Mammography (Gold Standard)**  ✅ **What it is:**   - A low-dose X-ray imaging of the breast. - Detects small tumors, calcifications, or abnormal growths.   ✅ **Types:**   - **2D Mammography:** Standard X-ray images from two angles. - **3D Mammography (Tomosynthesis):** Creates multiple images for a clearer, more detailed view, reducing false positives.   ✅ **Who should get it?**   - **Women 40-49**: Based on individual risk and doctor’s recommendation. - **Women 50-74**: Recommended every **1-2 years**. - **High-risk women** (family history, BRCA mutations): **Earlier & more frequent screening** advised.   ✅ **Pros:** ✔️ Detects tumors **years before symptoms appear**. ✔️ Reduces breast cancer mortality. ✔️ Widely available and relatively quick.  ✅ **Cons:** ❌ May **miss** some cancers (false negatives). ❌ Can lead to **false positives & unnecessary biopsies**. ❌ **Radiation exposure** (though very low).  **2. Breast Ultrasound**  ✅ **What it is:**   - Uses sound waves to create breast images, often used as a follow-up to abnormal mammograms.   ✅ **Who should get it?**   - Women with **dense breasts** (where mammograms may be less effective). - To distinguish **solid tumors from fluid-filled cysts**. - High-risk women who cannot undergo mammography.   ✅ **Pros:** ✔️ No radiation. ✔️ Better for **dense breasts**. ✔️ Can guide **needle biopsies**.  ✅ **Cons:** ❌ **Less effective than mammograms** for detecting early cancers. ❌ Can lead to **false positives**.  **3. Magnetic Resonance Imaging (MRI)**  ✅ **What it is:**   - Uses magnets and contrast dye to create highly detailed breast images.   ✅ **Who should get it?**   - **High-risk women** (BRCA1/BRCA2 mutations, strong family history). - Women with **dense breasts** who need additional screening.   ✅ **Pros:** ✔️ More sensitive than mammograms. ✔️ Detects **small, aggressive cancers**.  ✅ **Cons:** ❌ Expensive and not widely available. ❌ **May detect non-cancerous abnormalities**, leading to unnecessary tests.  **4. Clinical Breast Exam (CBE) & Self-Breast Exam (SBE)**  ✅ **What they are:**   - **CBE:** A healthcare provider manually examines the breasts for lumps or abnormalities. - **SBE:** Women examine their own breasts for changes in size, shape, or feel.   ✅ **Who should do it?**   - **CBE:** Part of routine checkups for women in their **20s & 30s**. - **SBE:** No longer officially recommended for screening but helps **awareness of normal breast changes**.   ✅ **Pros:** ✔️ **Easy & accessible**. ✔️ Encourages breast health awareness.  ✅ **Cons:** ❌ **Less effective** in detecting early cancer. ❌ May lead to unnecessary **anxiety & biopsies**.  **Which Screening Method Is Best for You?**   \| **Risk Level** \| **Recommended Screening** \| \| --- \| --- \| \| **Average Risk** (no family history, no BRCA mutations) \| **Mammogram every 1-2 years (starting at 40 or 50)** \| \| **High Risk** (BRCA mutations, strong family history) \| **Mammogram + MRI annually** \| \| **Dense Breasts** \| **Mammogram + Ultrasound or MRI** \| \| **Young Women (<40) with family history** \| **MRI or ultrasound (not mammogram)** \|   Would you like help determining your best screening plan based on your risk factors? 😊 | |
|  | Repeat | Several screening methods help detect breast cancer early, increasing the chances of successful treatment.  **1. Mammogram (X-ray of the Breast) – Gold Standard**  ✔ Uses **low-dose X-rays** to detect tumors or abnormal growths. ✔ Can detect cancer **before symptoms appear** (up to 2-3 years early). ✔ Recommended **annually or biennially**, depending on age and risk.  🔹 **Best for:** Women **40+ years old** (earlier if high risk). 🔹 **Limitations:** May miss tumors in **dense breast tissue** (false negatives).  **2. Breast Ultrasound**  ✔ Uses **sound waves** to create breast images. ✔ Helps determine if a lump is **solid (potential cancer) or fluid-filled (likely benign cyst)**. ✔ Often used as a follow-up to an **abnormal mammogram**.  🔹 **Best for:** Women with **dense breasts**, younger women, or those at high risk. 🔹 **Limitations:** Cannot detect **microcalcifications** (tiny early cancer signs).  **3. Breast MRI (Magnetic Resonance Imaging)**  ✔ Uses **magnetic fields and contrast dye** to produce detailed images. ✔ More sensitive than mammograms, detecting cancers **missed on X-rays**. ✔ Recommended for **high-risk women** (e.g., BRCA mutation carriers).  🔹 **Best for:** Women with **very high genetic risk** or prior radiation exposure. 🔹 **Limitations:** Can lead to **false positives**, requiring unnecessary biopsies.  **4. Clinical Breast Exam (CBE)**  ✔ Performed by a **doctor or nurse** during a routine check-up. ✔ Checks for **lumps, skin changes, or nipple abnormalities**.  🔹 **Best for:** Women of **all ages**, especially those with no access to imaging. 🔹 **Limitations:** Less effective than imaging in detecting early-stage cancer.  **5. Breast Self-Exam (BSE)**  ✔ Involves **self-checking** for lumps or changes in size, shape, or texture. ✔ Helps women become familiar with their **normal breast feel**.  🔹 **Best for:** Women who want to monitor their breast health. 🔹 **Limitations:** Not a replacement for mammograms; may lead to false alarms.  **Which Screening is Right for You?**  📌 **Ages 40-44** – Optional annual mammograms. 📌 **Ages 45-54** – Annual mammograms recommended. 📌 **Ages 55+** – Mammograms every 1-2 years. 📌 **High-Risk Women** – May need **MRI + mammograms** starting at age 30.  🔹 **Bottom Line:** Mammograms remain the **most effective** screening tool, but ultrasounds, MRIs, and exams help in specific cases. Regular screenings save lives by detecting breast cancer **early**. | |
| 8 | What is the survival rate of breast cancer? | The survival rate of breast cancer depends on several factors, including the **stage at diagnosis, tumor type, hormone receptor status, and overall health of the patient**. Below are the general survival rates based on stage.  **1. Overall 5-Year Survival Rate**   - **90%** for all breast cancer stages combined. - This means that 90 out of 100 women diagnosed with breast cancer survive at least **five years** after diagnosis.   **2. Survival Rate by Stage**   \| **Stage** \| **5-Year Survival Rate** \| \| --- \| --- \| \| **Localized (Stage 0 & I)** – Cancer is confined to the breast \| **99%** \| \| **Regional (Stage II & III)** – Cancer has spread to nearby lymph nodes \| **86%** \| \| **Distant (Stage IV – Metastatic)** – Cancer has spread to other organs \| **30%** \|  - **Localized breast cancer** has the highest survival rate because it is detected early. - **Metastatic breast cancer** (Stage IV) has a lower survival rate, but advancements in treatment are improving outcomes.   **3. Factors Affecting Survival**   - **Tumor Type:** **Hormone receptor-positive (ER/PR+) and HER2+ breast cancers** often respond better to treatment than **triple-negative breast cancer (TNBC)**. - **Age & Overall Health:** Younger patients with no other health issues tend to have better survival rates. - **Treatment Response:** Early detection and aggressive treatment improve survival chances. - **Genetic Mutations (BRCA1/BRCA2):** Some genetic mutations can affect prognosis and treatment options.   **4. 10-Year & 15-Year Survival Rates**   - **10-Year Survival Rate:** Around **84%** for all breast cancers. - **15-Year Survival Rate:** Around **80%**, but it varies by stage.   **5. Improvements in Survival Over Time**  Thanks to advancements in **targeted therapy, immunotherapy, and early detection**, survival rates for breast cancer have improved significantly over the last few decades.  Would you like information on treatment options to improve survival rates? 😊 | |
|  | Repeat | Breast cancer survival rates depend on **the stage at diagnosis, tumor type, and treatment effectiveness**. The **5-year survival rate** is a commonly used measure to estimate the percentage of patients who survive at least **five years** after diagnosis.  **Overall Survival Rates (U.S.)**   - **Localized (Early-stage, No Spread)** – **99%** - **Regional (Spread to Nearby Lymph Nodes)** – **86%** - **Distant (Metastatic, Spread to Other Organs)** – **30%** - **Overall 5-Year Survival Rate** – **90%**   **Survival Rates by Stage**   \| **Cancer Stage** \| **5-Year Survival Rate** \| \| --- \| --- \| \| **Stage 0** (DCIS, non-invasive) \| ~100% \| \| **Stage 1** (Small tumor, no lymph spread) \| ~99% \| \| **Stage 2** (Larger tumor or some lymph node involvement) \| ~93% \| \| **Stage 3** (More lymph node spread) \| ~72% \| \| **Stage 4** (Metastatic, distant spread) \| ~30% \|   **Factors Affecting Survival Rates**  ✔ **Early Detection** – Cancers detected early have a much higher survival rate. ✔ **Tumor Type & Grade** – Aggressive subtypes (e.g., triple-negative breast cancer) may have lower survival. ✔ **Age & Health** – Younger patients and those in good health respond better to treatment. ✔ **Treatment Advances** – Targeted therapies and immunotherapy improve outcomes. ✔ **Hormone Receptor Status** – **ER+/PR+ cancers** respond well to hormone therapy, improving survival.  **Improving Survival Chances**  ✔ **Regular Screening** – Detects cancer early, improving treatment success. ✔ **Timely Treatment** – Surgery, radiation, chemotherapy, and targeted therapy help control cancer. ✔ **Healthy Lifestyle** – Exercise, diet, and avoiding smoking/alcohol can support recovery.  **Bottom Line**  Breast cancer survival rates are **high**, especially when detected early. **Regular screenings, early treatment, and advances in medicine** continue to improve survival outcomes. | |
| 9 | What are the treatment methods of breast cancer? | The treatment of breast cancer depends on several factors, including **cancer stage, type, hormone receptor status (ER/PR), HER2 status, and overall patient health**. Below are the main treatment options:  **1. Surgery 🏥**  Surgery is often the first step in treating breast cancer, especially for early-stage cases.  **🔹 Breast-Conserving Surgery (Lumpectomy)**   - Removes only the tumor and a small margin of surrounding tissue. - Preserves most of the breast. - Usually followed by **radiation therapy** to kill remaining cancer cells. - Best for **early-stage (Stage I or II) tumors**.   **🔹 Mastectomy**   - Removes the entire breast. - Types:   - **Simple (Total) Mastectomy** – Removes the breast but leaves lymph nodes.   - **Modified Radical Mastectomy** – Removes the breast and lymph nodes.   - **Double Mastectomy** – Both breasts removed (often for high-risk patients, like those with BRCA mutations). - Sometimes followed by **breast reconstruction surgery**.   **🔹 Lymph Node Surgery**   - **Sentinel Lymph Node Biopsy (SLNB):** Checks if cancer has spread to the first few lymph nodes. - **Axillary Lymph Node Dissection:** Removes multiple lymph nodes if cancer has spread.   **2. Radiation Therapy 🔥**  Uses high-energy rays to kill cancer cells after surgery.  **🔹 External Beam Radiation**   - A machine directs radiation at the breast. - Usually given **daily for 3-6 weeks**.   **🔹 Internal Radiation (Brachytherapy)**   - Radioactive seeds or pellets are placed inside the breast. - Used in some **lumpectomy cases**.   ✅ **Best for:** ✔️ After **lumpectomy** to prevent recurrence. ✔️ Some cases of **mastectomy**, especially if cancer spread to lymph nodes.  ❌ **Side Effects:** Skin irritation, fatigue, swelling, and changes in breast texture.  **3. Chemotherapy 💊💉**  Drugs are used to **kill cancer cells or stop them from growing**.  ✅ **Used for:** ✔️ **Early-stage (before or after surgery)** – To shrink tumors (neoadjuvant) or prevent recurrence (adjuvant). ✔️ **Advanced/metastatic cancer** – To slow cancer spread and relieve symptoms.  **🔹 Common Chemo Drugs:**   - **Doxorubicin (Adriamycin)** - **Cyclophosphamide (Cytoxan)** - **Paclitaxel (Taxol)** - **Carboplatin** (for triple-negative breast cancer)   ❌ **Side Effects:** Hair loss, nausea, fatigue, infection risk, early menopause.  **4. Hormone (Endocrine) Therapy 🌿**  For **hormone receptor-positive (ER+/PR+)** breast cancer.  ✅ **How It Works:** Blocks estrogen or progesterone to stop tumor growth.  **🔹 Common Hormone Therapy Drugs:**   - **Tamoxifen** (for premenopausal & postmenopausal women). - **Aromatase Inhibitors (AIs)**: Anastrozole (Arimidex), Letrozole (Femara), Exemestane (Aromasin) – for postmenopausal women. - **Ovarian Suppression (with GnRH agonists)**: Stops ovaries from making estrogen.   ❌ **Side Effects:** Hot flashes, joint pain, bone loss, blood clots.  **5. Targeted Therapy 🎯**  Attacks specific molecules in cancer cells.  ✅ **Best for:** HER2-positive and some triple-negative breast cancers.  **🔹 HER2-Targeted Drugs**  For HER2-positive cancers (**20-25% of cases**).   - **Trastuzumab (Herceptin)** – Standard treatment. - **Pertuzumab (Perjeta)** – Often combined with Herceptin. - **T-DM1 (Kadcyla)** – Used after chemo.   **🔹 Other Targeted Drugs**   - **CDK4/6 Inhibitors** (Palbociclib, Ribociclib) – For hormone receptor-positive cancers. - **PARP Inhibitors** (Olaparib, Talazoparib) – For BRCA-mutated cancers.   ❌ **Side Effects:** Heart problems (HER2 drugs), low blood counts (CDK4/6 inhibitors).  **6. Immunotherapy 🛡️**  ✅ **Best for:** **Triple-negative breast cancer (TNBC)**.  **🔹 Common Immunotherapy Drugs:**   - **Atezolizumab (Tecentriq)** - **Pembrolizumab (Keytruda)**   ❌ **Side Effects:** Fatigue, nausea, immune system overreaction.  **7. Bone-Directed Therapy (For Metastatic Breast Cancer) 🦴**  For breast cancer that has spread to the bones.   - **Bisphosphonates (Zoledronic acid, Pamidronate)** – Strengthen bones. - **Denosumab (Xgeva)** – Prevents bone damage.   **Choosing the Right Treatment Plan**   \| **Breast Cancer Type** \| **Recommended Treatment** \| \| --- \| --- \| \| **Early-Stage (I & II)** \| Surgery + Radiation ± Chemo/Hormone Therapy \| \| **Locally Advanced (III)** \| Chemo + Surgery + Radiation ± Targeted Therapy \| \| **Metastatic (IV)** \| Chemo + Targeted Therapy ± Hormone Therapy ± Immunotherapy \| \| **HER2-Positive** \| HER2-Targeted Therapy + Chemo \| \| **Triple-Negative (TNBC)** \| Chemo + Immunotherapy (if eligible) \|   Would you like help understanding which treatment might be best based on specific factors? 😊 | |
|  | Repeat | Breast cancer treatment depends on factors like **cancer stage, type, hormone receptor status, and overall health**. Treatment may involve one or a combination of the following methods:  **1. Surgery (Removing the Tumor or Breast)**  🔹 **Lumpectomy (Breast-Conserving Surgery)** – Removes only the tumor and some surrounding tissue; best for early-stage cancer. 🔹 **Mastectomy** – Removes one or both breasts; recommended for larger tumors, multiple tumors, or high-risk cases. 🔹 **Lymph Node Removal (Axillary Dissection or Sentinel Node Biopsy)** – Checks if cancer has spread to lymph nodes.  ✅ **Best for:** Early-stage and localized cancer. ✅ **Goal:** Remove as much cancer as possible.  **2. Radiation Therapy (Killing Cancer Cells with High-Energy Beams)**  🔹 Often used **after lumpectomy or mastectomy** to destroy remaining cancer cells. 🔹 Can be external (X-rays) or internal (brachytherapy).  ✅ **Best for:** Early-stage cancer, large tumors, or lymph node involvement. ✅ **Goal:** Prevent recurrence.  **3. Chemotherapy (Killing Cancer Cells with Drugs)**  🔹 Uses **powerful drugs** (via IV or pills) to kill fast-growing cancer cells. 🔹 Given **before surgery (neoadjuvant)** to shrink tumors or **after surgery (adjuvant)** to prevent recurrence.  ✅ **Best for:** Advanced, aggressive, or lymph node-positive cancers. ✅ **Goal:** Destroy cancer cells throughout the body.  ⛔ **Side Effects:** Hair loss, nausea, fatigue, infection risk.  **4. Hormone Therapy (Blocking Estrogen/Progesterone to Stop Cancer Growth)**  🔹 Used for **hormone receptor-positive (ER+/PR+) breast cancers**. 🔹 **Common drugs:**   - **Tamoxifen** – For premenopausal & postmenopausal women. - **Aromatase Inhibitors (Anastrozole, Letrozole, Exemestane)** – For postmenopausal women.   ✅ **Best for:** ER+/PR+ breast cancer. ✅ **Goal:** Reduce cancer recurrence & growth.  ⛔ **Side Effects:** Menopause-like symptoms, bone loss, blood clots.  **5. Targeted Therapy (Attacking Specific Cancer Proteins)**  🔹 Used for **HER2-positive breast cancer** to block HER2 protein signals. 🔹 **Common drugs:**   - **Trastuzumab (Herceptin)** - **Pertuzumab (Perjeta)** - **Lapatinib, T-DM1 (Kadcyla)**   ✅ **Best for:** HER2+ breast cancer. ✅ **Goal:** Stop cancer growth with fewer side effects than chemotherapy.  ⛔ **Side Effects:** Heart problems, fatigue, diarrhea.  **6. Immunotherapy (Boosting the Immune System to Fight Cancer)**  🔹 Used for **triple-negative breast cancer (TNBC)**, which lacks hormone & HER2 receptors. 🔹 **Common drug:** **Atezolizumab (Tecentriq)**.  ✅ **Best for:** Advanced/metastatic TNBC. ✅ **Goal:** Help the immune system recognize and attack cancer.  ⛔ **Side Effects:** Autoimmune reactions, fatigue, inflammation.  **7. Bone-Directed Therapy (For Cancer Spread to Bones)**  🔹 **Bisphosphonates (Zoledronic Acid, Pamidronate)** – Strengthen bones & reduce fractures. 🔹 **Denosumab (Xgeva, Prolia)** – Helps prevent bone complications.  ✅ **Best for:** Metastatic breast cancer affecting bones.  **Which Treatment is Right for You?**  📌 **Early-Stage Breast Cancer:** Surgery + Radiation ± Hormone/Targeted Therapy. 📌 **Locally Advanced Cancer:** Chemotherapy + Surgery + Radiation + Hormone/Targeted Therapy. 📌 **Metastatic Cancer:** Chemotherapy + Targeted/Immunotherapy + Palliative Care.  🔹 **Bottom Line:** The best treatment plan depends on **cancer type, stage, and personal health factors**. A multidisciplinary team (oncologists, surgeons, radiologists) customizes the best approach for each patient. | |
| 10 | Can breast cancer recur? | Yes, **breast cancer can recur** even after successful treatment. Recurrence happens when cancer cells survive initial treatment and later grow again. It can occur **months to years** after treatment, depending on the type and stage of the original cancer.  **Types of Breast Cancer Recurrence**  **1. Local Recurrence (Same Breast or Surgery Site)**   - Cancer reappears in the **same breast or chest wall** after a lumpectomy or mastectomy. - Symptoms:   - A new **lump** or thickening in the same area.   - **Redness, swelling, or skin changes** (like dimpling or ulceration).   - **Changes in the surgical scar** or persistent pain.   ✅ **Treatment Options:** ✔️ **Surgery** (Mastectomy if lumpectomy was done before). ✔️ **Radiation therapy** (if not used previously). ✔️ **Hormone therapy**, **targeted therapy**, or **chemotherapy** if needed.  **2. Regional Recurrence (Nearby Lymph Nodes)**   - Cancer spreads to **lymph nodes in the armpit, collarbone, or chest wall**. - Symptoms:   - **Swollen lymph nodes** in the armpit or neck.   - **Pain or swelling** in the chest or arm.   ✅ **Treatment Options:** ✔️ **Surgery** to remove affected lymph nodes. ✔️ **Radiation therapy** to target remaining cancer cells. ✔️ **Systemic treatments** (chemo, hormone therapy, or targeted therapy).  **3. Distant (Metastatic) Recurrence (Stage IV)**   - Cancer spreads to **distant organs** (lungs, liver, bones, brain). - **Most common sites:** **Bones, lungs, liver, brain**. - Symptoms depend on the affected organ:   - **Bones**: Bone pain, fractures.   - **Lungs**: Persistent cough, shortness of breath.   - **Liver**: Yellow skin (jaundice), swelling in the abdomen.   - **Brain**: Headaches, vision problems, seizures.   ✅ **Treatment Options:** ✔️ **Hormone therapy** (if ER/PR+). ✔️ **Targeted therapy** (if HER2+ or BRCA mutation present). ✔️ **Chemotherapy & immunotherapy**. ✔️ **Radiation or surgery** for symptom relief.  **Risk Factors for Recurrence 🚨**   - **Tumor Size & Stage**: Larger tumors and **Stage III cancers** have a higher risk. - **Lymph Node Involvement**: The more **lymph nodes affected**, the higher the risk. - **Hormone Receptor Status**: **Triple-negative breast cancer (TNBC)** has a higher recurrence rate. - **HER2 Status**: HER2+ cancers can recur, but **targeted therapy (Herceptin, Perjeta)** reduces this risk. - **Incomplete Treatment**: Missing treatments (like radiation after lumpectomy) increases risk.   **How to Reduce the Risk of Recurrence? 🛡️**  ✔️ **Follow-up Care**: Regular **mammograms, blood tests, and scans**. ✔️ **Hormone Therapy**: Take **Tamoxifen or Aromatase Inhibitors** for ER/PR+ cancers. ✔️ **Healthy Lifestyle**: Exercise, maintain a healthy weight, and eat a balanced diet. ✔️ **Avoid Smoking & Alcohol**: Reduces inflammation and recurrence risk. ✔️ **Take Targeted Therapy (if eligible)**: **Herceptin (HER2+), CDK4/6 inhibitors, or PARP inhibitors** for high-risk patients.  Would you like guidance on recurrence prevention based on a specific breast cancer type? | |
|  | Repeat | Yes, **breast cancer can recur** after treatment, even if the original tumor was completely removed. Recurrence happens when cancer cells remain undetected in the body and later start growing again.  **Types of Breast Cancer Recurrence**  **1. Local Recurrence (Same Breast or Surgery Area)**  📍 Cancer returns in the **same breast, chest wall, or surgical scar**. 🔹 May cause a **new lump, skin changes, or swelling** near the original site. 🔹 Treated with **surgery, radiation, or systemic therapies (hormone, chemo, targeted therapy)**.  **2. Regional Recurrence (Nearby Lymph Nodes)**  📍 Cancer spreads to **lymph nodes in the armpit, collarbone, or neck**. 🔹 Symptoms include **lumps, swelling, or pain in the affected area**. 🔹 Treated with **surgery, radiation, chemotherapy, or targeted therapy**.  **3. Distant (Metastatic) Recurrence (Stage 4 Breast Cancer)**  📍 Cancer spreads to distant organs like the **bones, lungs, liver, or brain**. 🔹 Symptoms depend on the affected organ (e.g., bone pain, breathing issues, headaches). 🔹 Treated with **chemotherapy, hormone therapy, immunotherapy, or targeted therapy** to control spread.  **Risk Factors for Recurrence**  Certain factors increase the likelihood of breast cancer returning: ✔ **Lymph Node Involvement** – More affected lymph nodes = higher recurrence risk. ✔ **Tumor Size & Aggressiveness** – Larger and high-grade tumors are more likely to return. ✔ **Incomplete Treatment** – Skipping radiation, chemo, or hormone therapy raises risk. ✔ **Hormone Receptor Status** – **Triple-negative breast cancer (TNBC)** has a higher recurrence risk. ✔ **Delayed or No Follow-up Care** – Regular check-ups help detect recurrence early.  **How to Reduce the Risk of Recurrence?**  ✅ **Complete Recommended Treatments** – Finish chemotherapy, radiation, hormone, or targeted therapy as prescribed. ✅ **Maintain a Healthy Lifestyle** – Eat a **balanced diet, exercise regularly, and avoid smoking/alcohol**. ✅ **Take Hormone Therapy (if prescribed)** – If ER+/PR+, continuing **tamoxifen or aromatase inhibitors** for 5-10 years lowers recurrence risk. ✅ **Attend Regular Follow-Ups** – Get **mammograms, blood tests, and scans** as recommended. ✅ **Manage Stress & Get Support** – Emotional well-being helps overall recovery.  **Bottom Line**  Breast cancer can **recur months or years after treatment**, but **early detection and preventive measures** can help lower the risk and improve survival. If you notice **new lumps, pain, or unusual symptoms**, consult your doctor immediately. | |
| **DeepSeek** | | |  |
|  | Question | Answer |  |
| 1 | What are the common symptoms of breast cancer? | The common symptoms of breast cancer can vary, but some of the most frequently reported signs include:   1. **Lump in the Breast or Underarm**: A new lump or mass that feels hard, irregular, and painless is one of the most common signs. However, some lumps may be tender or soft. 2. **Changes in Breast Size or Shape**: Unexplained swelling, shrinkage, or asymmetry of the breast. 3. **Skin Changes**: Dimpling, puckering, or redness of the breast skin, resembling an orange peel (peau d'orange). 4. **Nipple Changes**: Inversion of the nipple, unusual discharge (especially if bloody), or scaling/crusting around the nipple. 5. **Breast Pain**: Persistent pain or tenderness in a specific area of the breast. 6. **Swelling**: Swelling in part or all of the breast, even if no distinct lump is felt. 7. **Redness or Warmth**: Unexplained redness, warmth, or darkening of the breast skin. 8. **Lymph Node Changes**: Swelling or lumps in the lymph nodes under the arm or near the collarbone.   It’s important to note that these symptoms can also be caused by benign (non-cancerous) conditions. However, if any of these signs persist, it is crucial to consult a healthcare professional for further evaluation. Early detection through regular screenings, such as mammograms, and self-exams can significantly improve outcomes.  For more information, refer to trusted sources like the **American Cancer Society** (cancer.org) or the **National Cancer Institute** (cancer.gov). |  |
|  | Repeat | Breast cancer symptoms can vary, but some common signs include:   1. **Lump in the Breast or Underarm**: A new lump or mass that feels hard and is painless (though some may be tender or soft). 2. **Changes in Breast Size or Shape**: Swelling, shrinkage, or asymmetry of the breast. 3. **Skin Changes**: Dimpling, puckering, redness, or thickening of the skin (like an orange peel). 4. **Nipple Changes**: Inversion (turning inward), discharge (other than breast milk), or scaling/crusting. 5. **Pain**: Persistent pain in the breast or nipple area. 6. **Swelling**: Swelling in part or all of the breast, even if no lump is felt. 7. **Redness or Warmth**: Unusual redness or warmth in the breast. 8. **Lymph Node Changes**: Swelling or lumps in the underarm or collarbone area.   Not all lumps or changes indicate cancer, but any unusual symptoms should be evaluated by a healthcare professional. Regular screenings, like mammograms, are crucial for early detection. |  |
| 2 | What are the risk factors for breast cancer? | Breast cancer risk factors can be categorized into **modifiable** (lifestyle-related) and **non-modifiable** (unchangeable) factors. Understanding these can help in assessing risk and taking preventive measures.  **Non-Modifiable Risk Factors:**   1. **Gender**: Women are at significantly higher risk than men. 2. **Age**: Risk increases with age, particularly after 50. 3. **Family History**: A close relative (mother, sister, daughter) with breast cancer increases risk. 4. **Genetic Mutations**: Inherited mutations in genes like **BRCA1** and **BRCA2** significantly raise risk. 5. **Personal History**: Previous breast cancer or certain non-cancerous breast conditions (e.g., atypical hyperplasia). 6. **Dense Breast Tissue**: Denser breasts have a higher risk and can make detection harder. 7. **Reproductive History**: Early menstruation (before 12) or late menopause (after 55) increases exposure to hormones. 8. **Radiation Exposure**: Previous radiation therapy to the chest area, especially during adolescence.   **Modifiable Risk Factors:**   1. **Physical Inactivity**: Lack of regular exercise increases risk. 2. **Obesity**: Excess weight, especially after menopause, is linked to higher risk. 3. **Alcohol Consumption**: Regular alcohol use is associated with increased risk. 4. **Hormone Replacement Therapy (HRT)**: Long-term use of combined estrogen and progesterone HRT. 5. **Oral Contraceptives**: Slightly increased risk with current or recent use. 6. **Pregnancy History**: Never having been pregnant or having a first pregnancy after 30. 7. **Breastfeeding**: Not breastfeeding may slightly increase risk. 8. **Diet**: A diet high in saturated fats and low in fruits and vegetables may contribute.   **Other Factors:**   - **Environmental Exposures**: Prolonged exposure to certain chemicals or pollutants. - **Smoking**: Some studies suggest a link between smoking and breast cancer risk.   While some risk factors cannot be changed, lifestyle modifications like maintaining a healthy weight, exercising regularly, limiting alcohol, and avoiding smoking can help reduce risk. Regular screenings and genetic counseling (if there’s a family history) are also crucial for early detection and prevention.  For more detailed information, consult trusted sources like the **American Cancer Society** (cancer.org) or the **National Cancer Institute** (cancer.gov). |  |
|  | Repeat | Breast cancer risk factors can be categorized into **modifiable** (factors you can change) and **non-modifiable** (factors you cannot change). Here's a breakdown:  **Non-Modifiable Risk Factors**   1. **Gender**: Women are at significantly higher risk than men. 2. **Age**: Risk increases with age, especially after 50. 3. **Family History**: A close relative (mother, sister, daughter) with breast cancer increases risk. 4. **Genetic Mutations**: Inherited mutations in genes like **BRCA1** and **BRCA2** significantly increase risk. 5. **Personal History**: Previous breast cancer or certain non-cancerous breast conditions (e.g., atypical hyperplasia). 6. **Race/Ethnicity**: White women are slightly higher risk, but Black women are more likely to develop aggressive forms. 7. **Dense Breast Tissue**: Denser breasts increase risk and make detection harder. 8. **Reproductive History**:    - Early menstruation (before age 12).    - Late menopause (after age 55). 9. **Radiation Exposure**: Previous radiation therapy to the chest area, especially during adolescence.   **Modifiable Risk Factors**   1. **Physical Inactivity**: Lack of regular exercise increases risk. 2. **Obesity**: Being overweight or obese, especially after menopause, raises risk. 3. **Alcohol Consumption**: Regular alcohol use is linked to higher risk. 4. **Hormone Replacement Therapy (HRT)**: Long-term use of combined estrogen and progesterone HRT increases risk. 5. **Oral Contraceptives**: Slightly increased risk with current or recent use. 6. **Pregnancy History**:    - No full-term pregnancies.    - First pregnancy after age 30. 7. **Breastfeeding**: Not breastfeeding may slightly increase risk. 8. **Diet**: A diet high in saturated fats and low in fruits and vegetables may contribute. 9. **Smoking**: Long-term smoking is associated with increased risk.   **Other Factors**   - **Environmental Exposures**: Prolonged exposure to certain chemicals or pollutants. - **Night Shift Work**: Disruption of circadian rhythms may increase risk.   **Reducing Risk**   - Maintain a healthy weight. - Exercise regularly. - Limit alcohol. - Avoid smoking. - Breastfeed if possible. - Discuss hormone therapy risks with your doctor.   If you have significant risk factors, consult a healthcare provider for personalized screening and prevention strategies. |  |
| 3 | How to prevent breast cancer? | While there is no guaranteed way to prevent breast cancer, certain lifestyle changes and proactive measures can significantly reduce your risk. Here are some evidence-based strategies:  **1. Maintain a Healthy Weight**   - Obesity, especially after menopause, increases breast cancer risk. Aim for a balanced diet and regular physical activity to maintain a healthy weight.   **2. Stay Physically Active**   - Engage in at least **150 minutes of moderate exercise** (e.g., brisk walking) or **75 minutes of vigorous exercise** (e.g., running) per week. Regular activity helps regulate hormones and boosts immunity.   **3. Limit Alcohol Consumption**   - Alcohol is a known risk factor. If you drink, limit intake to **no more than one drink per day**.   **4. Eat a Healthy Diet**   - Focus on a diet rich in:   - **Fruits and vegetables** (high in antioxidants)   - **Whole grains**   - **Lean proteins**   - **Healthy fats** (e.g., omega-3 fatty acids from fish) - Limit processed foods, red meat, and saturated fats.   **5. Avoid Smoking**   - Smoking is linked to many cancers, including breast cancer. If you smoke, seek help to quit.   **6. Breastfeed if Possible**   - Breastfeeding for at least several months may reduce breast cancer risk by lowering estrogen levels.   **7. Limit Hormone Replacement Therapy (HRT)**   - Long-term use of combined estrogen and progesterone HRT increases risk. Discuss alternatives with your doctor if you’re considering HRT for menopause symptoms.   **8. Be Aware of Your Family History**   - If you have a family history of breast or ovarian cancer, consider **genetic counseling** to assess your risk for mutations like **BRCA1** or **BRCA2**.   **9. Regular Screenings**   - Early detection saves lives. Follow recommended guidelines:   - **Mammograms**: Start at age 40–50, depending on risk factors and doctor’s advice.   - **Clinical Breast Exams**: Have your doctor examine your breasts regularly.   - **Self-Exams**: Be familiar with how your breasts look and feel, and report any changes to your doctor.   **10. Consider Preventive Medications or Surgery (for High-Risk Individuals)**   - **Medications**: Drugs like tamoxifen or raloxifene may be prescribed for high-risk women. - **Prophylactic Surgery**: In rare cases, preventive mastectomy or ovary removal may be considered for those with very high genetic risk.   **11. Reduce Environmental Exposures**   - Minimize exposure to radiation and environmental pollutants, such as certain chemicals found in plastics or pesticides.   **12. Manage Stress and Mental Health**   - Chronic stress may weaken the immune system. Practice stress-reducing activities like yoga, meditation, or mindfulness.   **Key Takeaway:**  While some risk factors (like genetics) cannot be changed, adopting a healthy lifestyle and staying vigilant with screenings can significantly lower your risk. Always consult your healthcare provider for personalized advice.  For more information, refer to trusted sources like the **American Cancer Society** (cancer.org) or the **National Cancer Institute** (cancer.gov). |  |
|  | Repeat | While there is no guaranteed way to prevent breast cancer, there are several evidence-based strategies to **reduce your risk**. These include lifestyle changes, early detection, and medical interventions for those at higher risk. Here’s a comprehensive guide:  **Lifestyle Changes**   1. **Maintain a Healthy Weight**:    - Obesity, especially after menopause, increases breast cancer risk. Aim for a balanced diet and regular exercise to maintain a healthy weight. 2. **Exercise Regularly**:    - Engage in at least **150 minutes of moderate-intensity exercise** (e.g., brisk walking) or **75 minutes of vigorous exercise** (e.g., running) per week. 3. **Limit Alcohol Consumption**:    - The more alcohol you drink, the higher your risk. If you drink, limit it to **no more than 1 drink per day**. 4. **Avoid Smoking**:    - Smoking is linked to many cancers, including breast cancer. Seek help to quit if needed. 5. **Breastfeed if Possible**:    - Breastfeeding for at least several months may reduce risk, especially if done for a year or more. 6. **Eat a Healthy Diet**:    - Focus on a diet rich in fruits, vegetables, whole grains, and lean proteins.    - Limit processed foods, red meat, and saturated fats. 7. **Limit Hormone Therapy**:    - Avoid long-term use of combined estrogen and progesterone hormone replacement therapy (HRT) after menopause. Discuss alternatives with your doctor.   **Early Detection and Screening**   1. **Regular Mammograms**:    - Follow screening guidelines based on your age and risk level. For average-risk women, mammograms are typically recommended starting at age **40–50**. 2. **Breast Self-Exams**:    - Be familiar with how your breasts look and feel. Report any changes (e.g., lumps, nipple discharge, or skin changes) to your doctor. 3. **Clinical Breast Exams**:    - Have a healthcare provider examine your breasts during routine check-ups.   **Medical Interventions for High-Risk Individuals**   1. **Genetic Testing and Counseling**:    - If you have a strong family history of breast cancer or known genetic mutations (e.g., BRCA1/BRCA2), consider genetic testing and counseling. 2. **Preventive Medications**:    - For high-risk women, medications like **tamoxifen** or **raloxifene** may reduce the risk of hormone receptor-positive breast cancer. Discuss with your doctor. 3. **Prophylactic Surgery**:    - In extreme cases (e.g., BRCA mutation carriers), some women opt for preventive mastectomy or ovary removal to significantly reduce risk.   **Other Preventive Measures**   1. **Limit Exposure to Environmental Toxins**:    - Reduce exposure to chemicals linked to cancer, such as pesticides and industrial pollutants. 2. **Manage Stress**:    - Chronic stress may weaken the immune system. Practice stress-reducing techniques like yoga, meditation, or mindfulness. 3. **Get Enough Sleep**:    - Poor sleep and disrupted circadian rhythms may increase cancer risk. Aim for **7–9 hours of quality sleep** per night.   **For High-Risk Individuals**   - If you have a family history or genetic predisposition, work with your doctor to create a personalized prevention plan, which may include more frequent screenings or preventive treatments.   **Key Takeaway**  While you can’t eliminate all risk, adopting a healthy lifestyle, staying vigilant with screenings, and addressing high-risk factors can significantly reduce your chances of developing breast cancer. Always consult your healthcare provider for personalized advice. |  |
| 4 | Can breast cancer be inherited? | Yes, breast cancer can be inherited, but it accounts for only a small percentage of cases. Inherited genetic mutations play a significant role in increasing the risk of breast cancer. Here’s what you need to know:  **1. Inherited Genetic Mutations**   - Certain gene mutations passed down from parents can significantly increase the risk of breast cancer. The most well-known are:   - **BRCA1** and **BRCA2**: These mutations are responsible for the majority of hereditary breast and ovarian cancers.   - Other genes, such as **TP53**, **PTEN**, **PALB2**, **CHEK2**, and **ATM**, also increase risk but are less common.   **2. How Inheritance Works**   - These mutations are inherited in an **autosomal dominant** pattern, meaning only one copy of the mutated gene (from either parent) is needed to increase cancer risk. - If a parent carries a mutation, there’s a **50% chance** of passing it to their child.   **3. Risk Levels**   - Women with a **BRCA1** or **BRCA2** mutation have a **45–85% lifetime risk** of developing breast cancer, compared to about **12%** in the general population. - These mutations also increase the risk of **ovarian cancer** and, in men, **breast and prostate cancer**.   **4. Family History Red Flags**   - A family history of the following may suggest an inherited risk:   - Multiple relatives with breast or ovarian cancer.   - Breast cancer diagnosed at a young age (under 50).   - Male breast cancer in the family.   - Ashkenazi Jewish ancestry (higher prevalence of BRCA mutations).   **5. Genetic Testing and Counseling**   - If you have a strong family history, consider **genetic counseling** and **testing** to identify mutations. - Testing can help guide preventive measures, such as:   - Increased surveillance (e.g., more frequent mammograms or MRIs).   - Preventive medications (e.g., tamoxifen).   - Prophylactic surgery (e.g., mastectomy or ovary removal).   **6. Not All Familial Breast Cancer is Inherited**   - Even if no specific mutation is found, a family history of breast cancer can still increase risk due to shared environmental or lifestyle factors.   **Key Takeaway:**  While most breast cancers are not inherited, genetic mutations like **BRCA1** and **BRCA2** can significantly increase risk. If you have a family history of breast or ovarian cancer, consult a genetic counselor to assess your risk and explore preventive options.  For more information, refer to trusted sources like the **American Cancer Society** (cancer.org) or the **National Cancer Institute** (cancer.gov). |  |
|  | Repeat | Yes, breast cancer can be inherited, but it’s important to note that **only a small percentage of breast cancer cases (about 5–10%) are directly linked to inherited genetic mutations**. Most breast cancers are caused by a combination of genetic, environmental, and lifestyle factors.  **Inherited Breast Cancer and Genetic Mutations**   1. **BRCA1 and BRCA2 Mutations**:    - The most well-known genetic mutations linked to breast cancer are in the **BRCA1** and **BRCA2** genes.    - These mutations significantly increase the risk of both breast and ovarian cancer.    - Women with a BRCA1 mutation have a **55–65% lifetime risk** of developing breast cancer, and those with a BRCA2 mutation have a **45% lifetime risk**. 2. **Other Gene Mutations**:    - Mutations in other genes, such as **PALB2**, **TP53**, **PTEN**, **CHEK2**, and **ATM**, can also increase breast cancer risk, though to a lesser extent than BRCA mutations.   **Family History and Risk**   - A strong family history of breast or ovarian cancer can indicate an inherited risk, especially if:   - Multiple relatives on the same side of the family have had breast or ovarian cancer.   - Cancer occurs at a younger age (e.g., before menopause).   - There are cases of male breast cancer in the family.   - A family member has had both breast and ovarian cancer.   **What to Do if You Have a Family History**   1. **Genetic Counseling and Testing**:    - If you have a family history of breast or ovarian cancer, consider genetic counseling and testing to determine if you carry a mutation.    - A genetic counselor can help you understand your risk and guide you on next steps. 2. **Increased Surveillance**:    - If you’re at higher risk, your doctor may recommend more frequent screenings, such as:      - Annual mammograms starting at an earlier age.      - Breast MRI for more detailed imaging. 3. **Preventive Measures**:    - **Medications**: Drugs like tamoxifen or raloxifene may reduce risk in high-risk individuals.    - **Prophylactic Surgery**: Some high-risk women choose to undergo preventive mastectomy or ovary removal to significantly reduce their risk.   **Key Takeaway**  While most breast cancers are not inherited, a small percentage are linked to genetic mutations like BRCA1 and BRCA2. If you have a strong family history of breast or ovarian cancer, consult a genetic counselor or healthcare provider to assess your risk and explore preventive options. Early detection and proactive management can make a significant difference. |  |
| 5 | Why is breast cancer screening important? | Breast cancer screening is crucial because it can detect cancer early, often before symptoms appear, when treatment is most effective. Here are the key reasons why screening is important:  **1. Early Detection Saves Lives**   - Screening can identify breast cancer at an early stage, when it is most treatable. - Early detection is associated with a **higher survival rate** and less aggressive treatment options.   **2. Detects Cancer Before Symptoms Appear**   - Many breast cancers, especially in their early stages, do not cause noticeable symptoms. - Screening tools like mammograms can detect small tumors or abnormalities that cannot be felt during a physical exam.   **3. Reduces the Risk of Advanced Cancer**   - Regular screening helps catch cancer before it spreads to other parts of the body (metastasis), which is harder to treat.   **4. Identifies High-Risk Individuals**   - Screening can reveal conditions like **dense breast tissue** or precancerous changes (e.g., atypical hyperplasia), which may require closer monitoring or preventive measures.   **5. Provides Peace of Mind**   - For many women, regular screening offers reassurance and reduces anxiety about breast health.   **6. Tailored Screening for High-Risk Groups**   - Women with a family history of breast cancer or genetic mutations (e.g., BRCA1/BRCA2) may benefit from more frequent or advanced screening methods, such as **MRI** or **ultrasound**, in addition to mammograms.   **7. Cost-Effective and Accessible**   - Screening programs are widely available and often covered by insurance, making them accessible to many women.   **Screening Methods:**   1. **Mammography**: The most common screening tool, effective for detecting tumors in their early stages. 2. **Clinical Breast Exam (CBE)**: A physical exam performed by a healthcare provider. 3. **Breast Self-Exams (BSE)**: Encourages women to be familiar with their breasts and report any changes. 4. **MRI or Ultrasound**: Used for women with dense breasts or high risk.   **When to Start Screening:**   - Guidelines vary, but generally:   - **Average Risk**: Start mammograms at age **40–50**, depending on recommendations from your doctor or health organization.   - **High Risk**: Begin earlier (e.g., age 30) and consider additional screening methods.   **Key Takeaway:**  Breast cancer screening is a powerful tool for early detection, which significantly improves outcomes and reduces mortality. Regular screening, combined with awareness of personal risk factors, is essential for maintaining breast health.  For more information, consult trusted sources like the **American Cancer Society** (cancer.org) or the **National Cancer Institute** (cancer.gov). |  |
|  | Repeat | Breast cancer screening is crucial because it can **detect cancer early**, often before symptoms appear. Early detection significantly improves the chances of successful treatment and survival. Here are the key reasons why breast cancer screening is important:  **1. Early Detection Saves Lives**   - Breast cancer found at an **early stage** (when it’s small and hasn’t spread) is easier to treat and has a much higher survival rate. - Regular screening can detect cancer before it spreads to lymph nodes or other parts of the body.   **2. Reduces the Need for Aggressive Treatment**   - Early-stage breast cancer often requires less aggressive treatment (e.g., smaller surgeries, fewer rounds of chemotherapy) compared to advanced-stage cancer.   **3. Identifies Cancer Before Symptoms Develop**   - Many breast cancers, especially in their early stages, do not cause noticeable symptoms. - Screening tools like mammograms can detect abnormalities (e.g., small tumors or microcalcifications) that are not yet palpable or visible.   **4. Helps Monitor High-Risk Individuals**   - For women with a family history of breast cancer or genetic mutations (e.g., BRCA1/BRCA2), regular screening is essential for early detection and proactive management.   **5. Provides Peace of Mind**   - Regular screening can reassure women that they are taking proactive steps to monitor their health.   **Screening Methods**   1. **Mammograms**:    - The most common and effective screening tool for breast cancer.    - Can detect tumors that are too small to feel.    - Recommended for women aged **40–74**, depending on risk factors and guidelines. 2. **Clinical Breast Exams (CBE)**:    - A physical exam performed by a healthcare provider to check for lumps or other changes. 3. **Breast Self-Exams**:    - While not a substitute for mammograms, being familiar with your breasts can help you notice changes and report them to your doctor. 4. **Breast MRI**:    - Used for women at high risk (e.g., BRCA mutation carriers) or to further evaluate abnormalities found on a mammogram.   **Screening Guidelines**   - **Average-Risk Women**:   - Start mammograms at age **40–50** and continue every **1–2 years** until age **74**.   - Discuss the best schedule with your doctor based on your individual risk. - **High-Risk Women**:   - Begin screening earlier (e.g., age 30) and consider additional tests like breast MRI.   **Benefits vs. Risks of Screening**   - **Benefits**:   - Early detection of cancer.   - Improved treatment outcomes.   - Reduced mortality rates. - **Risks**:   - False positives (abnormal results that turn out not to be cancer).   - Overdiagnosis (detecting slow-growing cancers that may not have caused harm).   - Exposure to low-dose radiation (minimal risk with modern mammography).   **Key Takeaway**  Breast cancer screening is a vital tool for early detection, which can lead to more effective treatment and better outcomes. If you’re unsure about when to start screening or which tests are right for you, consult your healthcare provider to create a personalized plan based on your risk factors. |  |
| 6 | What are the risks of breast cancer screening? | While breast cancer screening is a vital tool for early detection, it is not without risks or limitations. Understanding these risks can help individuals make informed decisions about their screening choices. Here are the potential risks and downsides:  **1. False Positives**   - A false positive occurs when a screening test suggests cancer, but further testing reveals no cancer. - **Consequences**:   - Unnecessary anxiety and stress.   - Additional tests (e.g., biopsies, ultrasounds, MRIs), which can be invasive and costly. - **Frequency**: About **10% of women** undergoing mammograms will have a false positive result.   **2. Overdiagnosis and Overtreatment**   - Overdiagnosis occurs when screening detects cancers that would never have caused symptoms or become life-threatening (e.g., slow-growing or non-invasive cancers like ductal carcinoma in situ, or DCIS). - **Consequences**:   - Unnecessary treatments, such as surgery, radiation, or chemotherapy, which carry their own risks and side effects.   **3. False Negatives**   - A false negative occurs when screening fails to detect cancer that is present. - **Consequences**:   - Delayed diagnosis and treatment.   - False reassurance, leading to missed symptoms. - **Frequency**: Mammograms miss about **1 in 5 cancers**, especially in women with dense breast tissue.   **4. Radiation Exposure**   - Mammograms use low-dose radiation, which carries a small risk of increasing cancer risk over time. - **Consequences**:   - While the risk is minimal, repeated exposure over many years could theoretically contribute to cancer development.   **5. Discomfort or Pain**   - Mammograms involve compressing the breast, which can cause temporary discomfort or pain for some women.   **6. Psychological Impact**   - The process of screening and waiting for results can cause anxiety, stress, or fear, even if the results are ultimately negative.   **7. Financial Costs**   - While many insurance plans cover screening, additional tests (e.g., follow-up biopsies or MRIs) can lead to out-of-pocket expenses.   **8. Limited Effectiveness in Younger Women or Dense Breasts**   - Mammograms are less effective in younger women or those with dense breast tissue, as both factors can make it harder to detect abnormalities. - **Alternative Screening**: Women with dense breasts may need additional imaging, such as ultrasound or MRI.   **9. Overreliance on Screening**   - Relying solely on screening may lead to neglecting other preventive measures, such as lifestyle changes or awareness of symptoms.   **Key Takeaway:**  While breast cancer screening has undeniable benefits, it is important to weigh the potential risks and limitations. Discuss your personal risk factors, family history, and preferences with your healthcare provider to determine the best screening plan for you.  For more information, consult trusted sources like the **American Cancer Society** (cancer.org) or the **National Cancer Institute** (cancer.gov). |  |
|  | Repeat | Breast cancer screening, particularly mammograms, is a vital tool for early detection, but it does come with some potential risks and limitations. Understanding these risks can help you make informed decisions about screening. Here are the main risks associated with breast cancer screening:  **1. False Positives**   - **What it means**: A false positive occurs when a screening test suggests cancer, but further testing reveals no cancer. - **Impact**:   - Can cause anxiety and stress.   - Leads to additional tests, such as diagnostic mammograms, ultrasounds, or biopsies, which can be uncomfortable and costly.   **2. Overdiagnosis and Overtreatment**   - **What it means**: Overdiagnosis occurs when screening detects slow-growing cancers or non-invasive conditions (e.g., ductal carcinoma in situ or DCIS) that may never cause symptoms or become life-threatening. - **Impact**:   - May lead to unnecessary treatments, such as surgery, radiation, or chemotherapy, which carry their own risks and side effects.   **3. False Negatives**   - **What it means**: A false negative occurs when a screening test misses cancer that is present. - **Impact**:   - Can delay diagnosis and treatment, leading to a false sense of security.   - More common in women with dense breast tissue, as dense tissue can hide tumors on mammograms.   **4. Radiation Exposure**   - **What it means**: Mammograms use low-dose X-rays, which expose the breasts to a small amount of radiation. - **Impact**:   - The risk of radiation-induced cancer is very low, especially with modern mammography machines.   - However, repeated exposure over many years may slightly increase risk, particularly for younger women.   **5. Discomfort or Pain**   - **What it means**: Mammograms involve compressing the breast between two plates to get a clear image. - **Impact**:   - Some women find the process uncomfortable or even painful, though it is usually brief.   **6. Psychological Stress**   - **What it means**: The screening process, including waiting for results or dealing with false positives, can cause anxiety and stress. - **Impact**:   - Emotional distress, even if the results ultimately show no cancer.   **7. Limited Effectiveness in Dense Breasts**   - **What it means**: Women with dense breast tissue have a higher risk of breast cancer, but mammograms are less effective at detecting tumors in dense breasts. - **Impact**:   - May require additional screening methods, such as ultrasound or MRI, which can increase costs and the risk of false positives.   **8. Financial Costs**   - **What it means**: Screening and follow-up tests can be expensive, especially if not fully covered by insurance. - **Impact**:   - Out-of-pocket costs for additional tests or treatments can be a burden.   **Weighing the Risks and Benefits**  While these risks exist, the benefits of breast cancer screening—particularly early detection and improved survival rates—often outweigh the potential downsides for most women. The key is to make an informed decision based on your individual risk factors, age, and preferences.  **What You Can Do**   1. **Discuss with Your Doctor**:    - Talk about your personal risk factors and the best screening plan for you.    - Ask about the benefits and risks of screening in your specific case. 2. **Consider Supplemental Screening**:    - If you have dense breasts or are at high risk, ask about additional screening methods like ultrasound or MRI. 3. **Stay Informed**:    - Follow screening guidelines based on your age and risk level.    - Be aware of changes in your breasts and report them to your doctor.   **Key Takeaway**  Breast cancer screening is a powerful tool for early detection, but it’s important to understand its limitations and risks. By working with your healthcare provider, you can make informed decisions that balance the benefits and risks for your individual situation. |  |
| 7 | What are the common screening methods for breast cancer? | Breast cancer screening involves various methods to detect cancer early, often before symptoms appear. The choice of screening method depends on factors like age, risk level, and breast density. Here are the most common screening methods:  **1. Mammography**   - **What it is**: An X-ray of the breast used to detect tumors or abnormalities. - **Who it’s for**: Women aged **40 and older**, especially those at average risk. - **Benefits**:   - Effective for early detection of breast cancer.   - Reduces mortality by catching cancer before it spreads. - **Limitations**:   - Less effective in women with dense breast tissue.   - Can produce false positives or false negatives.   **2. Clinical Breast Exam (CBE)**   - **What it is**: A physical examination of the breasts performed by a healthcare provider. - **Who it’s for**: Women of all ages, often as part of routine check-ups. - **Benefits**:   - Helps identify lumps or changes that may not be visible on imaging.   - Complements other screening methods. - **Limitations**:   - Less effective than mammography for detecting small tumors.   **3. Breast Self-Exam (BSE)**   - **What it is**: Regular self-examination of the breasts to check for lumps, changes in size, or other abnormalities. - **Who it’s for**: Women of all ages, as a way to stay familiar with their breast health. - **Benefits**:   - Encourages awareness of breast changes.   - Easy to perform at home. - **Limitations**:   - Not a substitute for clinical exams or mammograms.   - May lead to unnecessary anxiety if changes are detected.   **4. Magnetic Resonance Imaging (MRI)**   - **What it is**: A imaging technique that uses magnetic fields and radio waves to create detailed images of the breast. - **Who it’s for**:   - Women at **high risk** (e.g., BRCA mutation carriers, strong family history).   - Women with **dense breast tissue** or unclear mammogram results. - **Benefits**:   - Highly sensitive for detecting cancer, especially in high-risk women.   - Often used in combination with mammograms. - **Limitations**:   - Higher rate of false positives.   - Expensive and not routinely recommended for average-risk women.   **5. Ultrasound**   - **What it is**: Uses sound waves to create images of the breast tissue. - **Who it’s for**:   - Women with dense breast tissue.   - Further evaluation of abnormalities found on mammograms. - **Benefits**:   - Helps distinguish between solid masses and fluid-filled cysts.   - No radiation exposure. - **Limitations**:   - Not a standalone screening tool for average-risk women.   - Less effective than mammography for detecting small tumors.   **6. Digital Breast Tomosynthesis (3D Mammography)**   - **What it is**: An advanced form of mammography that creates 3D images of the breast. - **Who it’s for**: Women of all risk levels, especially those with dense breasts. - **Benefits**:   - Reduces false positives and improves detection of small tumors.   - Provides clearer images compared to traditional mammography. - **Limitations**:   - May involve higher radiation exposure (though still within safe limits).   - Not universally available.   **7. Thermography**   - **What it is**: Uses infrared imaging to detect heat patterns in the breast. - **Who it’s for**: Not widely recommended as a standalone screening tool. - **Benefits**:   - No radiation exposure. - **Limitations**:   - Not as reliable as mammography or MRI.   - Not endorsed by major health organizations for routine screening.   **Key Takeaway:**  The most common and recommended screening method for average-risk women is **mammography**, often combined with clinical breast exams. High-risk women may benefit from additional methods like **MRI** or **ultrasound**. Discuss your personal risk factors and preferences with your healthcare provider to determine the best screening plan for you.  For more information, consult trusted sources like the **American Cancer Society** (cancer.org) or the **National Cancer Institute** (cancer.gov). |  |
|  | Repeat | Breast cancer screening involves methods to detect cancer before symptoms appear. Early detection improves treatment outcomes and survival rates. Here are the **common screening methods** for breast cancer:  **1. Mammogram**   - **What it is**: An X-ray of the breast used to detect tumors or abnormalities. - **Who it’s for**: Women aged **40 and older**, especially those at average risk. - **How often**: Typically every **1–2 years**, depending on age and risk factors. - **Benefits**:   - Most effective screening tool for early detection.   - Can detect small tumors or microcalcifications before they are palpable. - **Limitations**:   - Less effective in women with dense breast tissue.   - May produce false positives or false negatives.   **2. Clinical Breast Exam (CBE)**   - **What it is**: A physical examination of the breasts by a healthcare provider. - **Who it’s for**: Women of all ages, often as part of a routine check-up. - **How often**: Every **1–3 years** for women in their 20s and 30s, and annually for women 40 and older. - **Benefits**:   - Helps identify lumps or changes that may not be visible on imaging. - **Limitations**:   - Less effective than mammograms for detecting early-stage cancer.   **3. Breast Self-Exam (BSE)**   - **What it is**: A regular check of your own breasts for lumps, thickening, or other changes. - **Who it’s for**: Women of all ages. - **How often**: Monthly, ideally a few days after your menstrual period ends. - **Benefits**:   - Helps you become familiar with how your breasts normally look and feel.   - Encourages early reporting of changes to your doctor. - **Limitations**:   - Not a substitute for mammograms or clinical exams.   - May lead to unnecessary anxiety if changes are detected.   **4. Breast MRI (Magnetic Resonance Imaging)**   - **What it is**: A detailed imaging test that uses magnets and radio waves to create pictures of the breast. - **Who it’s for**:   - Women at **high risk** (e.g., BRCA mutation carriers, strong family history).   - Women with dense breast tissue or abnormal mammogram results. - **How often**: Annually, often in addition to mammograms. - **Benefits**:   - Highly sensitive for detecting cancer, especially in high-risk women. - **Limitations**:   - Higher rate of false positives compared to mammograms.   - Expensive and not routinely used for average-risk women.   **5. Ultrasound**   - **What it is**: An imaging test that uses sound waves to create pictures of the breast. - **Who it’s for**:   - Women with dense breast tissue.   - Further evaluation of abnormalities found on a mammogram or clinical exam. - **Benefits**:   - Helps distinguish between solid masses and fluid-filled cysts.   - No radiation exposure. - **Limitations**:   - Not a standalone screening tool for average-risk women.   - May miss small tumors detected by mammograms.   **6. 3D Mammography (Tomosynthesis)**   - **What it is**: An advanced type of mammogram that creates a 3D image of the breast. - **Who it’s for**: Women of all risk levels, especially those with dense breasts. - **Benefits**:   - More accurate than standard mammograms in detecting cancer.   - Reduces the need for follow-up tests. - **Limitations**:   - May not be available everywhere.   - Slightly higher radiation dose than standard mammograms (though still within safe limits).   **7. Genetic Testing**   - **What it is**: A blood or saliva test to identify mutations in genes like BRCA1 and BRCA2. - **Who it’s for**: Women with a strong family history of breast or ovarian cancer. - **Benefits**:   - Helps identify high-risk individuals who may need more frequent or specialized screening. - **Limitations**:   - Not a screening tool for cancer itself, but a way to assess risk.   **Screening Guidelines**   - **Average-Risk Women**:   - Start mammograms at age **40–50** and continue every **1–2 years** until age **74**.   - Discuss the best schedule with your doctor. - **High-Risk Women**:   - Begin screening earlier (e.g., age 30) and consider additional tests like MRI.   **Key Takeaway**  The most common screening method is the mammogram, but other tools like MRI, ultrasound, and clinical exams may be used depending on your risk factors and breast density. Talk to your doctor to determine the best screening plan for you. Early detection through regular screening is the best way to improve breast cancer outcomes. |  |
| 8 | What is the survival rate of breast cancer? | The survival rate for breast cancer varies depending on factors such as the stage at diagnosis, the type of breast cancer, the patient’s age, overall health, and access to treatment. Here’s an overview of survival rates based on the **stage of breast cancer**:  **1. 5-Year Relative Survival Rates (Based on SEER Data):**  The **5-year relative survival rate** compares the survival of breast cancer patients to that of the general population. It is categorized by the extent of cancer spread:   - **Localized (Early Stage)**: Cancer is confined to the breast.   - **5-Year Survival Rate**: **99%**   - This high rate highlights the importance of early detection through screening. - **Regional Spread**: Cancer has spread to nearby lymph nodes or tissues.   - **5-Year Survival Rate**: **86%** - **Distant Metastasis**: Cancer has spread to distant organs (e.g., lungs, liver, bones).   - **5-Year Survival Rate**: **30%**   **2. 10-Year Relative Survival Rates:**   - **Overall**: Approximately **84%** of women survive breast cancer for 10 years or more after diagnosis. - **Early-Stage**: Survival rates remain high for localized breast cancer, often exceeding **90%**.   **3. Factors Influencing Survival Rates:**   - **Stage at Diagnosis**: Early detection significantly improves survival. - **Subtype of Breast Cancer**:   - Hormone receptor-positive (ER+/PR+) cancers generally have better outcomes.   - HER2-positive cancers have improved survival with targeted therapies like trastuzumab.   - Triple-negative breast cancer (TNBC) is more aggressive and has a lower survival rate. - **Age and Overall Health**: Younger, healthier patients often have better outcomes. - **Access to Treatment**: Timely and effective treatment improves survival.   **4. Improvements Over Time:**   - Advances in screening, early detection, and treatment (e.g., targeted therapies, immunotherapy) have significantly improved survival rates over the past few decades. - For example, the 5-year survival rate for breast cancer in the 1970s was around **75%**, compared to **90%** today.   **Key Takeaway:**  The survival rate for breast cancer is highly favorable when detected early, with a **99% 5-year survival rate for localized cases**. However, survival drops significantly if the cancer has spread to distant organs. Regular screening, awareness of symptoms, and timely treatment are critical for improving outcomes.  For more detailed statistics and personalized information, consult trusted sources like the **American Cancer Society** (cancer.org) or the **National Cancer Institute** (cancer.gov). |  |
|  | Repeat | The survival rate for breast cancer varies significantly depending on the stage at which it is diagnosed, the type of breast cancer, the patient's age, overall health, and the treatments available. Here are some general statistics based on the stage of breast cancer:   1. **Stage 0 (DCIS - Ductal Carcinoma In Situ):** The 5-year relative survival rate is close to 100%. This is because DCIS is a non-invasive cancer where abnormal cells are found in the lining of the breast milk duct but have not spread outside the duct. 2. **Stage I:** The 5-year relative survival rate is also close to 100%. At this stage, the tumor is small (2 centimeters or less) and has not spread to the lymph nodes. 3. **Stage II:** The 5-year relative survival rate is about 93%. In this stage, the tumor may be larger (2 to 5 centimeters) or may have spread to a few nearby lymph nodes. 4. **Stage III:** The 5-year relative survival rate is about 72%. This stage includes larger tumors (more than 5 centimeters) and/or cancer that has spread to several lymph nodes or other tissues near the breast. 5. **Stage IV (Metastatic Breast Cancer):** The 5-year relative survival rate drops to about 22%. At this stage, the cancer has spread to distant parts of the body, such as the bones, liver, lungs, or brain.   It's important to note that these statistics are based on data from large groups of people and may not predict individual outcomes. Advances in treatment and early detection continue to improve survival rates. Always consult with healthcare professionals for the most accurate and personalized information. |  |
| 9 | What are the treatment methods of breast cancer? | Breast cancer treatment depends on the type, stage, and characteristics of the cancer, as well as the patient’s overall health and preferences. Treatment often involves a combination of therapies. Here are the primary treatment methods:  **1. Surgery**   - **Lumpectomy**: Removal of the tumor and a small margin of surrounding tissue. Often followed by radiation therapy. - **Mastectomy**: Removal of the entire breast. Options include:   - **Simple Mastectomy**: Removal of breast tissue only.   - **Modified Radical Mastectomy**: Removal of breast tissue and lymph nodes.   - **Double Mastectomy**: Removal of both breasts, often considered for high-risk patients. - **Lymph Node Removal**:   - **Sentinel Lymph Node Biopsy**: Removal of a few lymph nodes to check for cancer spread.   - **Axillary Lymph Node Dissection**: Removal of multiple lymph nodes if cancer is detected.   **2. Radiation Therapy**   - Uses high-energy beams to kill cancer cells or prevent their growth. - Often used after surgery (especially lumpectomy) to reduce the risk of recurrence. - **Types**:   - **External Beam Radiation**: Most common, targets the breast or chest wall.   - **Brachytherapy**: Internal radiation, often used for partial breast irradiation.   **3. Chemotherapy**   - Uses drugs to kill cancer cells or stop their growth. - **When used**:   - Before surgery (**neoadjuvant chemotherapy**) to shrink tumors.   - After surgery (**adjuvant chemotherapy**) to eliminate remaining cancer cells.   - For advanced or metastatic breast cancer. - **Administration**: Oral or intravenous.   **4. Hormone Therapy**   - Used for hormone receptor-positive (ER+/PR+) breast cancers. - Blocks hormones (estrogen or progesterone) that fuel cancer growth. - **Common Drugs**:   - **Tamoxifen**: Blocks estrogen receptors.   - **Aromatase Inhibitors** (e.g., letrozole, anastrozole): Reduce estrogen production in postmenopausal women.   - **Ovarian Suppression**: Shuts down ovaries in premenopausal women.   **5. Targeted Therapy**   - Targets specific molecules involved in cancer growth. - **HER2-Positive Breast Cancer**:   - **Trastuzumab (Herceptin)**: Targets HER2 protein.   - **Pertuzumab (Perjeta)**: Often used in combination with trastuzumab.   - **Ado-trastuzumab emtansine (Kadcyla)**: Combines targeted therapy with chemotherapy. - **Other Targeted Drugs**:   - **CDK4/6 Inhibitors** (e.g., palbociclib, ribociclib): For hormone receptor-positive, HER2-negative cancers.   - **PARP Inhibitors** (e.g., olaparib): For BRCA mutation carriers.   **6. Immunotherapy**   - Boosts the immune system to fight cancer. - **PD-1/PD-L1 Inhibitors** (e.g., pembrolizumab): Used for triple-negative breast cancer (TNBC) that expresses PD-L1.   **7. Treatment by Stage**   - **Early-Stage (I-II)**:   - Surgery (lumpectomy or mastectomy) + radiation.   - Chemotherapy or hormone therapy, depending on tumor characteristics. - **Locally Advanced (III)**:   - Neoadjuvant chemotherapy to shrink tumors, followed by surgery and radiation.   - Hormone or targeted therapy if applicable. - **Metastatic (Stage IV)**:   - Focus on controlling cancer growth and improving quality of life.   - Treatments include chemotherapy, hormone therapy, targeted therapy, and immunotherapy.   **8. Palliative Care**   - Focuses on relieving symptoms and improving quality of life for advanced-stage patients. - Can be used alongside other treatments.   **Key Takeaway:**  Breast cancer treatment is highly personalized and often involves a combination of surgery, radiation, chemotherapy, hormone therapy, and targeted therapies. Advances in precision medicine and immunotherapy continue to improve outcomes. Always discuss treatment options with your healthcare team to determine the best approach for your specific case.  For more information, consult trusted sources like the **American Cancer Society** (cancer.org) or the **National Cancer Institute** (cancer.gov). |  |
|  | Repeat | The treatment of breast cancer depends on the type, stage, and characteristics of the cancer, as well as the patient's overall health and preferences. Common treatment methods include:  **1. Surgery**   - **Lumpectomy:** Removal of the tumor and a small margin of surrounding tissue (breast-conserving surgery). - **Mastectomy:** Removal of the entire breast. This can be:   - **Simple mastectomy:** Removal of the breast tissue.   - **Modified radical mastectomy:** Removal of the breast tissue and nearby lymph nodes. - **Sentinel Lymph Node Biopsy:** Removal of a few lymph nodes to check for cancer spread. - **Axillary Lymph Node Dissection:** Removal of multiple lymph nodes if cancer is detected in the sentinel nodes.   **2. Radiation Therapy**   - Uses high-energy beams to kill cancer cells or prevent them from growing. - Often used after lumpectomy or mastectomy to reduce the risk of recurrence. - Can also be used to treat metastatic breast cancer in specific areas.   **3. Chemotherapy**   - Uses drugs to kill cancer cells or stop their growth. - May be given before surgery (neoadjuvant chemotherapy) to shrink tumors or after surgery (adjuvant chemotherapy) to kill remaining cancer cells. - Commonly used for aggressive or advanced breast cancer.   **4. Hormone Therapy (Endocrine Therapy)**   - Used for hormone receptor-positive breast cancers (ER+ or PR+). - Blocks hormones (estrogen or progesterone) that fuel cancer growth. - Common medications:   - **Tamoxifen:** Blocks estrogen receptors.   - **Aromatase inhibitors (e.g., letrozole, anastrozole):** Reduce estrogen production in postmenopausal women.   - **Ovarian suppression:** Shuts down ovaries in premenopausal women.   **5. Targeted Therapy**   - Targets specific molecules or pathways involved in cancer growth. - Common examples:   - **HER2-targeted therapies (e.g., trastuzumab, pertuzumab):** For HER2-positive breast cancer.   - **CDK4/6 inhibitors (e.g., palbociclib, ribociclib):** For hormone receptor-positive, HER2-negative breast cancer.   - **PARP inhibitors (e.g., olaparib):** For BRCA-mutated breast cancer.   **6. Immunotherapy**   - Boosts the immune system to fight cancer. - **Pembrolizumab (Keytruda):** Used for some triple-negative breast cancers (PD-L1 positive).   **7. Combination Therapies**   - Often, a combination of treatments (e.g., surgery + radiation + chemotherapy) is used for the best outcomes.   **8. Palliative Care**   - Focuses on relieving symptoms and improving quality of life, especially for advanced or metastatic breast cancer.   **Personalized Treatment**  Treatment plans are tailored based on:   - **Cancer subtype:** Hormone receptor status, HER2 status, and genetic mutations (e.g., BRCA). - **Stage of cancer:** Early-stage, locally advanced, or metastatic. - **Patient factors:** Age, overall health, and preferences.   Always consult with a healthcare team to determine the best treatment approach for individual cases. |  |
| 10 | Can breast cancer recur? | Yes, breast cancer can recur even after successful treatment. Recurrence happens when cancer cells that were not eliminated during initial treatment grow and cause new tumors. Here’s an overview of breast cancer recurrence, including types, risk factors, and prevention strategies:  **Types of Breast Cancer Recurrence**   1. **Local Recurrence**:    - Cancer returns in the same area as the original tumor (e.g., breast, chest wall, or surgical scar).    - Common after lumpectomy or mastectomy. 2. **Regional Recurrence**:    - Cancer returns in nearby lymph nodes or tissues (e.g., under the arm or near the collarbone). 3. **Distant Recurrence (Metastasis)**:    - Cancer spreads to distant organs, such as the bones, liver, lungs, or brain.    - This is considered advanced or metastatic breast cancer.   **Risk Factors for Recurrence**   - **Tumor Characteristics**:   - Larger tumor size.   - High-grade tumors (aggressive cancer cells).   - Hormone receptor-negative (ER-/PR-) or triple-negative breast cancer (TNBC).   - HER2-positive breast cancer (if not treated with targeted therapy). - **Lymph Node Involvement**:   - Cancer spread to lymph nodes increases recurrence risk. - **Incomplete Treatment**:   - Not completing recommended surgery, chemotherapy, radiation, or hormone therapy. - **Younger Age at Diagnosis**:   - Women under 35 may have a higher risk of recurrence. - **Genetic Mutations**:   - BRCA1 or BRCA2 mutations increase the risk of recurrence and new cancers. - **Lifestyle Factors**:   - Obesity, smoking, alcohol use, and lack of physical activity can contribute to higher recurrence risk.   **Signs and Symptoms of Recurrence**   - **Local or Regional Recurrence**:   - New lump in the breast, chest wall, or underarm.   - Skin changes (e.g., redness, dimpling, or thickening).   - Nipple discharge or inversion. - **Distant Recurrence**:   - Persistent pain (e.g., in bones, back, or joints).   - Shortness of breath or cough (lung involvement).   - Jaundice, abdominal pain, or swelling (liver involvement).   - Headaches, dizziness, or seizures (brain involvement).   **Preventing Recurrence**   1. **Complete Initial Treatment**:    - Follow through with all recommended therapies (surgery, radiation, chemotherapy, hormone therapy). 2. **Hormone Therapy**:    - For hormone receptor-positive cancers, take hormone-blocking medications (e.g., tamoxifen, aromatase inhibitors) as prescribed. 3. **Targeted Therapy**:    - For HER2-positive cancers, continue targeted therapies like trastuzumab. 4. **Lifestyle Changes**:    - Maintain a healthy weight.    - Exercise regularly.    - Limit alcohol and avoid smoking.    - Eat a balanced diet rich in fruits, vegetables, and whole grains. 5. **Regular Follow-Up Care**:    - Attend all scheduled check-ups, mammograms, and imaging tests.    - Report any new symptoms to your doctor immediately. 6. **Genetic Counseling**:    - If you have a BRCA mutation or strong family history, consider preventive measures like prophylactic surgery.   **Treatment for Recurrent Breast Cancer**   - **Local or Regional Recurrence**:   - Surgery, radiation, or additional systemic therapy (chemotherapy, hormone therapy, or targeted therapy). - **Distant Recurrence**:   - Focus on controlling cancer growth and improving quality of life.   - Treatments may include chemotherapy, hormone therapy, targeted therapy, immunotherapy, or palliative care.   **Key Takeaway:**  Breast cancer can recur, but the risk can be reduced through complete treatment, lifestyle changes, and regular follow-up care. Early detection of recurrence improves treatment outcomes, so it’s important to stay vigilant and report any new symptoms to your healthcare provider.  For more information, consult trusted sources like the **American Cancer Society** (cancer.org) or the **National Cancer Institute** (cancer.gov). |  |
|  | Repeat | Yes, breast cancer can recur even after successful treatment. Recurrence happens when cancer cells that were not completely eliminated during initial treatment begin to grow again. The risk of recurrence depends on several factors, including the type and stage of the original cancer, the treatments received, and individual patient characteristics.  **Types of Breast Cancer Recurrence**   1. **Local Recurrence:**    - Cancer returns in the same area as the original tumor (e.g., the breast or chest wall).    - More common in patients who underwent breast-conserving surgery (lumpectomy) rather than mastectomy. 2. **Regional Recurrence:**    - Cancer returns in nearby lymph nodes or tissues, such as the armpit or collarbone area. 3. **Distant Recurrence (Metastatic Recurrence):**    - Cancer spreads to distant organs, such as the bones, liver, lungs, or brain.    - This is the most serious type of recurrence and is considered stage IV breast cancer.   **Risk Factors for Recurrence**   - **Cancer Stage at Diagnosis:** Higher-stage cancers (e.g., stage III) have a greater risk of recurrence. - **Tumor Characteristics:**   - Hormone receptor-negative or HER2-positive cancers may have a higher risk.   - Triple-negative breast cancer (TNBC) is associated with a higher risk of early recurrence. - **Lymph Node Involvement:** Cancer that has spread to lymph nodes increases the risk. - **Treatment Received:** Incomplete treatment (e.g., skipping radiation or hormone therapy) can increase the risk. - **Genetic Mutations:** BRCA1 or BRCA2 mutations may increase the likelihood of recurrence. - **Age at Diagnosis:** Younger patients (under 35) may have a higher risk of recurrence.   **Reducing the Risk of Recurrence**   1. **Adjuvant Therapies:**    - **Chemotherapy:** Kills remaining cancer cells after surgery.    - **Radiation Therapy:** Reduces the risk of local recurrence.    - **Hormone Therapy:** For hormone receptor-positive cancers, it lowers the risk of recurrence for 5-10 years or more.    - **Targeted Therapy:** For HER2-positive cancers, drugs like trastuzumab (Herceptin) reduce recurrence risk. 2. **Lifestyle Changes:**    - Maintain a healthy weight.    - Exercise regularly.    - Limit alcohol consumption.    - Avoid smoking. 3. **Regular Follow-Up Care:**    - Routine mammograms, physical exams, and blood tests to monitor for signs of recurrence.   **Signs of Recurrence**   - New lump in the breast or surgical scar. - Changes in the breast skin or nipple. - Persistent pain in the breast, chest, or bones. - Unexplained weight loss, fatigue, or shortness of breath. - Swelling in the armpit or collarbone area.   **Managing Recurrence**  If breast cancer recurs, treatment options depend on the type and location of recurrence:   - **Local or Regional Recurrence:** Surgery, radiation, or additional systemic therapies (chemotherapy, hormone therapy, or targeted therapy). - **Distant Recurrence:** Focuses on controlling the cancer and improving quality of life, often with systemic therapies like chemotherapy, hormone therapy, or immunotherapy.   While recurrence is a concern, advances in treatment and early detection have significantly improved outcomes for many patients. Regular follow-up and adherence to treatment plans are crucial for reducing the risk and managing recurrence effectively. |  |
