# Supplementary material for: ChatGPT Versus DeepSeek for Breast Cancer Information Retrieval: Quantitative Comparative Study
Source: JMIR Cancer. 2026 Feb 27;12:e72839. doi: 10.2196/72839 (PMC12954694; doi:10.2196/72839)
Supplement: Multimedia Appendix 7 [file cancer-v12-e72839-s007.docx]

**Supporting Information: Supplementary Table 7**

**ChatGPT vs. DeepSeek: A Comparative Analysis of AI Models for Breast Cancer Information Retrieval**

Rima Hajjo^1,2,3*^, Dima A. Sabbah^1^, Sanaa K. Bardaweel^4^

*^1^Department of Pharmacy, Faculty of Pharmacy, Al-Zaytoonah University of Jordan, P.O. Box 130, Amman 11733, Jordan.*

*^2^Laboratory for Molecular Modeling, Division of Chemical Biology and Medicinal Chemistry, Eshelman School of Pharmacy, The University of North Carolina at Chapel Hill, Chapel Hill, NC, USA.*

*^3^Board Member, Jordan CDC, Amman, Jordan.*

*^4^Department of Pharmaceutical Sciences, School of Pharmacy, University of Jordan, Amman 11942, Jordan.*

*Correspondence

Rima Hajjo

E-mail: rhajjo@gmail.com; [r.hajjo@zuj.edu.jo](mailto:r.hajjo@zuj.edu.jo)

ORCID: https://orcid.org/0000-0002-7090-5425

**Supplementary Table 7.** Information sources for ChatGPT-4.0 and DeepSeek-V3.

| **ChatGPT 4.0** | | | | |  |
| --- | --- | --- | --- | --- | --- |
|  | **Question** | | | **Answer** |  |
| 1 | What are the common symptoms of breast cancer? | | | References: Cancer.org. Breast Cancer Signs and Symptoms; available from: <https://www.cancer.org/cancer/types/breast-cancer/screening-tests-and-early-detection/breast-cancer-signs-and-symptoms.html?utm_source=chatgpt.com> (accessed on 1 February 2025)   1. CDC.gov. Symptoms of Breast Cancer; available from:   <https://www.cdc.gov/breast-cancer/symptoms/index.html?utm_source=chatgpt.com>  (accessed on 1 February 2025)   1. Komen.org. Warning Signs of Breast Cancer; available from: <https://www.komen.org/breast-cancer/signs-symptoms/warning-signs/?utm_source=chatgpt.com>   (accessed on 4 February 2025)   1. National Breast Cancer Foundation, Inc. Breast Cancer Signs and Symptoms; available from: <https://www.nationalbreastcancer.org/breast-cancer-symptoms-and-signs/>   (accessed on 4 February 2025)   1. MedicalNewsToday. What are the symptoms of breast cancer? available from: <https://www.medicalnewstoday.com/articles/327488>   (accessed on 4 February 2025)   1. WebMD. Breast Cancer Symptoms; available from: <https://www.webmd.com/breast-cancer/understanding-breast-cancer-symptoms>   (accessed on 4 February 2025)   1. VeryWellHealth. Breast Cancer Signs and Symptoms; available from: <https://www.verywellhealth.com/symptoms-of-breast-cancer-430640>   (accessed on 4 February 2025)     1. The University of Kansas Cancer Center. Early Signs of Breast Cancer; available from: <https://www.kucancercenter.org/news-room/blog/2020/07/8-early-signs-of-breast-cancer>   (accessed on 4 February 2025)   1. Myoclinic.org. Breast Cancer Symptoms and Causes; available from: <https://www.mayoclinic.org/diseases-conditions/breast-cancer/symptoms-causes/syc-20352470>   (accessed on 4 February 2025)   1. MD Anderson Cancer Center. Breast cancer symptoms you shouldn't ignore; available from: <https://www.mdanderson.org/publications/focused-on-health/breast-cancer-symptoms-you-shouldn-t-ignore.h10-1592991.html>   (accessed on 4 February 2025) |  |
| 2 | What are the risk factors for breast cancer? | | | National Breast Cancer Foundation, Inc. Risk Factors; available from: <https://www.nationalbreastcancer.org/breast-cancer-risk-factors/?utm_source=chatgpt.com> (accessed on 4 February 2025)   1. CDC.gov. Breast Cancer Risk Factors; available from: <https://www.cdc.gov/breast-cancer/risk-factors/index.html?utm_source=chatgpt.com>   (accessed on 4 February 2025)   1. American Cancer Society. Breast Cancer Risk and Prevention Methods; available from: <https://www.cancer.org/cancer/types/breast-cancer/risk-and-prevention.html?utm_source=chatgpt.com>   (accessed on 1 February 2025) Wikipedia.org. Risk factors for breast cancer; available from: <https://en.wikipedia.org/wiki/Risk_factors_for_breast_cancer?utm_source=chatgpt.com> (accessed on 1 February 2025)  1. Komen.org. Breast Cancer Risk Factors Table; available from: <https://www.komen.org/breast-cancer/risk-factor/table/>  (accessed on 4 February 2025)  1. Healthline.com. What Are Your Possible Risk Factors for Breast Cancer? available from: <https://www.healthline.com/health/breast-cancer/risk-factors-for-breast-cancer>   (accessed on 4 February 2025)   1. BCRF. What are the Major Risk Factors for Breast Cancer? available from: <https://www.bcrf.org/blog/risk-factors-for-breast-cancer/>   (accessed on 4 February 2025)   1. City of Hope. Breast cancer causes and risk factors; available from: <https://www.cancercenter.com/cancer-types/breast-cancer/risk-factors> (accessed on 4 February 2025) 2. BreastCancer.org. Breast Cancer Risk Factors; available from: <https://www.breastcancer.org/risk/risk-factors> (accessed on 4 February 2025) 3. American Cancer Society. Breast Cancer Risk Factors You Cannot Change ; available from: <https://www.cancer.org/cancer/types/breast-cancer/risk-and-prevention/breast-cancer-risk-factors-you-cannot-change.html> (accessed on 4 February 2025) 4. Komen.org. Factors Linked to Breast Cancer Risk; available from: <https://www.komen.org/breast-cancer/risk-factor/factors-that-affect-risk/>  (accessed on 4 February 2025)  1. Myoclinic.org. Breast Cancer Symptoms and Causes; available from: <https://www.mayoclinic.org/diseases-conditions/breast-cancer/symptoms-causes/syc-20352470>   (accessed on 4 February 2025) |  |
| 3 | How to prevent breast cancer? | | | 1. CDC.gov. Breast Cancer; Reducing Risk for Breast Cancer; available from:   <https://www.cdc.gov/breast-cancer/prevention/index.html?utm_source=chatgpt.com>  (accessed on 1 February 2025)   1. Cancer.org. Five Ways to Help Reduce Your Breast Cancer Risk; available from:   <https://www.cancer.org/cancer/latest-news/five-ways-to-reduce-your-breast-cancer-risk.html?utm_source=chatgpt.com>  (accessed on 1 February 2025)   1. Myoclinic.org. Healthy Lifestyle Women’s Health. Breast cancer prevention: How to reduce your risk; available from: Breast Cancer Symptoms and Causes; available from:   <https://www.mayoclinic.org/healthy-lifestyle/womens-health/in-depth/breast-cancer-prevention/art-20044676?utm_source=chatgpt.com>  (accessed on 1 February 2025)   1. American Cancer Society. Can I Lower My Risk of Breast Cancer? available from: <https://www.cancer.org/cancer/types/breast-cancer/risk-and-prevention/can-i-lower-my-risk.html?utm_source=chatgpt.com>   (accessed on 4 February 2025)   1. BCRF. 10Ways to Help Reduce Breast Cancer Risk; available from: <https://www.bcrf.org/blog/breast-cancer-prevention-breast-cancer-risk-reduction/>   (accessed on 4 February 2025)   1. MD Anderson Cancer Center. How to reduce your breast cancer risk; available from: <https://www.mdanderson.org/cancerwise/how-to-reduce-your-breast-cancer-risk.h00-159696756.html>   (accessed on 4 February 2025)   1. WebMD. Understanding Breast Cancer – Prevention; available from: <https://www.webmd.com/breast-cancer/understanding-breast-cancer-prevention>   (accessed on 4 February 2025)   1. Myoclinic.org. Breast cancer prevention: How to reduce your risk; available from: <https://www.mayoclinic.org/healthy-lifestyle/womens-health/in-depth/breast-cancer-prevention/art-20044676>   (accessed on 4 February 2025)   1. Myoclinic.org. How to reduce the risk of breast cancer; available from: <https://mcpress.mayoclinic.org/women-health/how-to-reduce-the-risk-of-breast-cancer/>   (accessed on 4 February 2025)   1. National Cancer Society. Breast Cancer Prevention (PDQ®)–Patient Version; available from: <https://www.cancer.gov/types/breast/patient/breast-prevention-pdq>   (accessed on 4 February 2025)   1. Healthline. How to Reduce Your Risk of Breast Cancer: 10 Lifestyle Recommendations; available from: <https://www.healthline.com/health/breast-cancer/prevention-for-breast-cancer>   (accessed on 4 February 2025)   1. American Cancer Society. Breast Cancer Risk and Prevention Methods; available from: <https://www.cancer.org/cancer/types/breast-cancer/risk-and-prevention.html> (accessed on 4 February 2025) 2. SITEMAN Cancer Center. 8IGHTWAYS® to Prevent Breast Cancer; available from: <https://siteman.wustl.edu/prevention/8-ways/8-ways-to-prevent-breast-cancer/>   (accessed on 4 February 2025) |  |
| 4 | Can breast cancer be inherited? | | | 1. Cancer.org. Breast Cancer Risk Factors You Cannot Change; available from: <https://www.cancer.org/cancer/types/breast-cancer/risk-and-prevention/breast-cancer-risk-factors-you-cannot-change.html?utm_source=chatgpt.com>   (accessed on 1 February 2025)   1. Cancer.org. BRCA Gene Changes: Cancer Risk and Genetic Testing; available from: <https://www.cancer.gov/about-cancer/causes-prevention/genetics/brca-fact-sheet?utm_source=chatgpt.com>   (accessed on 1 February 2025)   1. John Hopkins Medicine. Hereditary Breast Cancer**;** available from: <https://www.hopkinsmedicine.org/health/conditions-and-diseases/breast-cancer/hereditary-breast-cancer?utm_source=chatgpt.com>   (accessed on 1 February 2025)   1. Komen.org. Breast Cancer Risk Factors: Inherited Gene Mutations; available from: <https://www.komen.org/breast-cancer/risk-factor/gene-mutations-genetic-testing/inherited-genetic-mutations/?utm_source=chatgpt.com>   (accessed on 5 February 2025)   1. MD Anderson Cancer Center. Is breast cancer genetic? <https://www.mdanderson.org/cancerwise/does-breast-cancer-run-in-families.h00-159695967.html?utm_source=chatgpt.com>   (accessed on 5 February 2025)   1. Komen.org. Breast Cancer Risk Factors: Inherited Gene Mutations; available from: <https://www.komen.org/breast-cancer/risk-factor/gene-mutations-genetic-testing/inherited-genetic-mutations/>   (accessed on 5 February 2025)   1. BCRF. Is Breast Cancer Hereditary? Here’s What to Know; available from: <https://www.bcrf.org/blog/is-breast-cancer-hereditary/>   (accessed on 5 February 2025)   1. Cleveland Clinic. Breast Cancer Can Be Genetic: Here’s What To Know; available from: <https://health.clevelandclinic.org/is-breast-cancer-hereditary>   (accessed on 5 February 2025)   1. MedicalNewsToday. Can breast cancer genes skip a generation? available from: <https://www.medicalnewstoday.com/articles/breast-cancer-skip-generation>   (accessed on 5 February 2025)   1. Healthline. Is Breast Cancer Genetic? available from: <https://www.healthline.com/health/breast-cancer/is-breast-cancer-genetic>   (accessed on 5 February 2025)   1. WebMD. What Are Genetic Breast Cancer Mutations? available from: <https://www.webmd.com/breast-cancer/genetic-mutations>   (accessed on 5 February 2025)   1. HealthPartners. Hereditary breast cancer: Understanding your personal risk; available from: <https://www.healthpartners.com/blog/is-breast-cancer-genetic/>   (accessed on 5 February 2025)   1. Johns Hopkins Medicine. Hereditary Breast Cancer; available from:   <https://www.hopkinsmedicine.org/health/conditions-and-diseases/breast-cancer/hereditary-breast-cancer>  (accessed on 5 February 2025)   1. BreastCancer.org. Is Breast Cancer Hereditary? ; available from: <https://www.breastcancer.org/risk/risk-factors/genetics>   (accessed on 5 February 2025)   1. CancerResearch UK.org. Family history of breast cancer and inherited genes; available from: <https://www.cancerresearchuk.org/about-cancer/breast-cancer/risks-causes/family-history-and-inherited-genes>   (accessed on 5 February 2025)   1. American Cancer Society. Breast Cancer Risk Factors You Cannot Change; available from: <https://www.cancer.org/cancer/types/breast-cancer/risk-and-prevention/breast-cancer-risk-factors-you-cannot-change.html>   (accessed on 5 February 2025) |  |
| 5 | Why is breast cancer screening important? | | | 1. CDC Breast Cancer; Screening for Breast Cancer; available from: <https://www.cdc.gov/breast-cancer/screening/index.html?utm_source=chatgpt.com>   (accessed on 1 February 2025) UC Davis Health; Why it’s so important to get regular breast cancer screenings; available from: <https://health.ucdavis.edu/blog/cultivating-health/why-its-so-important-to-get-regular-breast-cancer-screenings/2022/10?utm_source=chatgpt.com>  (accessed on 2 February 2025)   1. [Mayo Clinic Comprehensive Cancer Center Blog](https://cancerblog.mayoclinic.org/). The importance of breast cancer screening; available from: <https://cancerblog.mayoclinic.org/2025/01/02/the-importance-of-breast-cancer-screening/> (accessed on 2 February 2025) 2. Wikipedia. Mammography; available from: <https://en.wikipedia.org/wiki/Mammography?utm_source=chatgpt.com>   (accessed on 5 February 2025)   1. VeryWellHealth. Ask the Expert: Why Is Breast Cancer Screening So Important?; available from: <https://www.verywellhealth.com/ask-the-expert-what-is-the-importance-of-breast-cancer-screening-6740783>   (accessed on 5 February 2025)   1. Mayo Clinic News Network. Mayo Clinic Q and A: How breast cancer screening can save lives; available from: <https://newsnetwork.mayoclinic.org/discussion/mayo-clinic-q-and-a-how-breast-cancer-screening-can-save-lives/>   (accessed on 5 February 2025)   1. Healthline. The Importance of Breast Cancer Screening and Early Detection; available from: <https://www.healthline.com/health/breast-cancer/early-detection-of-breast-cancer>   (accessed on 5 February 2025)   1. Mayo Clinic News Network. Mayo Clinic Minute: The importance of breast cancer screening; available from: <https://newsnetwork.mayoclinic.org/discussion/mayo-clinic-minute-the-importance-of-breast-cancer-screening/>   (accessed on 5 February 2025)   1. New York Health. Why Breast Screenings are so Important; available from: <https://nyhealth.com/news/why_breast_screenings_are_so_important>   (accessed on 5 February 2025)   1. American Cancer Society. Why We Screen for Some Cancers and Not Others; available from: <https://www.cancer.org/cancer/latest-news/why-we-screen-for-some-cancers-and-not-others.html>   (accessed on 5 February 2025)   1. City of Hope. 5 reasons why breast cancer screenings are crucial to women’s health; available from: <https://www.cityofhope.org/orange-county/blog/five-reasons-why-breast-cancer-screenings-are-crucial-womens-health>   (accessed on 5 February 2025)   1. Cancerscreenweek.org. Why is cancer screening important? available from: chrome -extension://efaidnbmnnnibpcajpcglclefindmkaj/https://www.cancerscreenweek.org/content/dam/gene/cancerscreenweek/resources/cancer-screening-fact-sheet-english.pdf   (accessed on 5 February 2025) |  |
| 6 | What are the risks of breast cancer screening? | | | 1. NHS. How to decide if you want breast screening; available from: <https://www.nhs.uk/conditions/breast-screening-mammogram/how-to-decide-if-you-want-breast-screening/?utm_source=chatgpt.com>   (accessed on 2 February 2025)   1. National Breast Cancer Foundation, Inc. Breast Cancer screening; available from:   <https://www.nationalbreastcancer.org/breast-cancer-screening/>  (accessed on 2 February 2025)   1. Komen.org. Weighing the Benefits and Risks of Screening Mammography; available from: <https://www.komen.org/breast-cancer/screening/mammography/benefits-risks/?utm_source=chatgpt.com>   (accessed on 2 February 2025)   1. CDC. Facts about Mammograms; available from: <https://www.cdc.gov/radiation-health/data-research/facts-stats/mammograms.html?utm_source=chatgpt.com>   (accessed on 5 February 2025)   1. National Breast Cancer Foundation, Inc. Breast Cancer Screening; available from: <https://www.nationalbreastcancer.org/breast-cancer-screening/> (accessed on 5 February 2025) 2. Mayo Clinic News Network. Mayo Clinic Q and A: How breast cancer screening can save lives; available from: <https://newsnetwork.mayoclinic.org/discussion/mayo-clinic-q-and-a-how-breast-cancer-screening-can-save-lives/>   (accessed on 5 February 2025)   1. Komen.org. What is a mammogram? available from: <https://www.komen.org/breast-cancer/screening/mammography/>   (accessed on 5 February 2025)   1. National Cancer Institute. Mammograms; available from: <https://www.cancer.gov/types/breast/mammograms-fact-sheet>   (accessed on 5 February 2025)   1. American Family Physicians. Breast Cancer Screening: Common Questions and Answers; available from: <https://www.aafp.org/pubs/afp/issues/2021/0101/p33.html>   (accessed on 5 February 2025)   1. American Cancer Society. American Cancer Society Recommendations for the Early Detection of Breast Cancer; available from: <https://www.cancer.org/cancer/types/breast-cancer/screening-tests-and-early-detection/american-cancer-society-recommendations-for-the-early-detection-of-breast-cancer.html>   (accessed on 5 February 2025)   1. The American College of Obstetricians and Gynecologists (ACOG). Breast Cancer Risk Assessment and Screening in Average-Risk Women; available from: <https://www.acog.org/clinical/clinical-guidance/practice-bulletin/articles/2017/07/breast-cancer-risk-assessment-and-screening-in-average-risk-women>   (accessed on 5 February 2025)   1. National Cancer Institute. Breast Cancer Screening (PDQ®)–Patient Version; available from: <https://www.cancer.gov/types/breast/patient/breast-screening-pdq>   (accessed on 5 February 2025) |  |
| 7 | What are the common screening methods for breast cancer? | | | American Cancer Society; Recommendations for the Early Detection of Breast Cancer; available from: <https://www.cancer.org/cancer/types/breast-cancer/screening-tests-and-early-detection/american-cancer-society-recommendations-for-the-early-detection-of-breast-cancer.html?utm_source=chatgpt.com> (accessed on 2 February 2025)   1. Harvard Health Publishing; What type of Breast screening do you need? available from: <https://www.health.harvard.edu/womens-health/what-type-of-breast-screening-do-you-need?utm_source=chatgpt.com>   (accessed on 2 February 2025)   1. National Cancer Institute. Screening Tests; available from: <https://www.cancer.gov/about-cancer/screening/screening-tests?utm_source=chatgpt.com>   (accessed on 6 February 2025)   1. CDC. Screening for Breast Cancer; available from: <https://www.cdc.gov/breast-cancer/screening/index.html?utm_source=chatgpt.com>   (accessed on 6 February 2025)   1. National Cancer Institute Seer Training Module. Screening; available from: <https://training.seer.cancer.gov/breast/screening.html>   (accessed on 6 February 2025)   1. Breast Cancer Research Foundation (BCRF). Mammograms: Everything You Need to Know; available from: <https://www.bcrf.org/blog/mammogram-breast-cancer-screening-research/>   (accessed on 6 February 2025)   1. National Breast Cancer Foundation, Inc. Breast Cancer Screening; available from: <https://www.nationalbreastcancer.org/breast-cancer-screening/>   (accessed on 6 February 2025)   1. National Cancer Institute. Breast Cancer Screening (PDQ®)–Health Professional Version; available from: <https://www.cancer.gov/types/breast/hp/breast-screening-pdq>   (accessed on 6 February 2025)   1. Dana-Farber Cancer Institute. What to Know About the Types of Breast Cancer Screening; available from: <https://blog.dana-farber.org/insight/2023/08/what-to-know-about-the-types-of-breast-cancer-screening/>   (accessed on 6 February 2025)   1. American Cancer Society. American Cancer Society Recommendations for the Early Detection of Breast Cancer;   ; available from: <https://www.cancer.org/cancer/types/breast-cancer/screening-tests-and-early-detection/american-cancer-society-recommendations-for-the-early-detection-of-breast-cancer.html>  (accessed on 6 February 2025)   1. Komen.org. **Breast Cancer Screening & Early Detection;** available from: <https://www.komen.org/breast-cancer/screening/>   (accessed on 6 February 2025)   1. Health Images. What Are All the Breast Cancer Screening Options? available from: <https://www.healthimages.com/what-are-all-the-breast-cancer-screening-options/>   (accessed on 6 February 2025) |  |
| 8 | What is the survival rate of breast cancer? | | | 1. National Breast Cancer Foundation, Inc.; Breast Cancer Facts & Stats; available from: <https://www.nationalbreastcancer.org/breast-cancer-facts/?utm_source=chatgpt.com>   (accessed on 2 February 2025) Komen.org. Understanding Breast Cancer Survival Rates; available from: <https://www.komen.org/breast-cancer/facts-statistics/breast-cancer-statistics/survival-rates/?utm_source=chatgpt.com> (accessed on 2 February 2025)   1. People.com. 1 in 8 Women Will Develop Breast Cancer in Their Lifetime, New Report Says ; available from: <https://people.com/breast-cancer-rates-rising-young-women-1-in-8-8721317?utm_source=chatgpt.com>   (accessed on 2 February 2025)   1. Komen.org. Breast Cancer Statistics; available from: <https://www.komen.org/breast-cancer/facts-statistics/breast-cancer-statistics/>   (accessed on 6 February 2025)   1. CDC. Breast Cancer Statistics; available from: <https://www.cdc.gov/breast-cancer/statistics/index.html>   (accessed on 6 February 2025)   1. WebMD. Breast Cancer Survival Rates; available from: <https://www.webmd.com/breast-cancer/breast-cancer-survival-rates>   (accessed on 6 February 2025)   1. Healthline. All About Breast Cancer Survival Rates and Outlook; available from: <https://www.healthline.com/health/breast-cancer/survival-facts-statistics>   (accessed on 6 February 2025)   1. Medical News Today. Breast cancer: Is it curable? available from: <https://www.medicalnewstoday.com/articles/is-breast-cancer-curable>   (accessed on 6 February 2025)   1. Living Beyond Breast Cancer (LBBC). Breast cancer life expectancy: Statistics and outcomes;   available from: <https://www.lbbc.org/about-breast-cancer/what-is-breast-cancer/life-expectancy>  (accessed on 6 February 2025)   1. VeryWellHealth. Most People With Early Stage Breast Cancer Will Be Long-Term Survivors: Study; available from: <https://www.verywellhealth.com/early-stage-breast-cancer-has-high-survival-rates-7555121>   (accessed on 6 February 2025)   1. Breast Cancer- Moose and Doc. Breast Cancer Survival Rates for all types of breast cancers; available from: <https://breast-cancer.ca/survicanc/>   (accessed on 6 February 2025)   1. American Cancer Society. Survival Rates for Breast Cancer; available from: <https://www.cancer.org/cancer/types/breast-cancer/understanding-a-breast-cancer-diagnosis/breast-cancer-survival-rates.html>   (accessed on 6 February 2025)   1. MOFFITT Cancer Center. Breast Cancer Survival Rate; available from: <https://www.moffitt.org/cancers/breast-cancer/survival-rate/>   (accessed on 6 February 2025) |  |
| 9 | What are the treatment methods of breast cancer? | | | Mayo Clinic Press. Breast Cancer Diagnosis and treatment; available from: <https://www.mayoclinic.org/diseases-conditions/breast-cancer/diagnosis-treatment/drc-20352475?utm_source=chatgpt.com> (accessed on 2 February 2025)   1. CDC Breast Cancer. Treatment of Breast Cancer; available from: <https://www.cdc.gov/breast-cancer/treatment/index.html?utm_source=chatgpt.com>     (accessed on 2 February 2025)   1. American Cancer Society. Treating Breast Cancer; available from: <https://www.cancer.org/cancer/types/breast-cancer/treatment.html?utm_source=chatgpt.com>   (accessed on 2 February 2025)   1. National Cancer Institute. Breast Cancer Treatment (PDQ®)–Patient Version; available from: <https://www.cancer.gov/types/breast/patient/breast-treatment-pdq?utm_source=chatgpt.com>   (accessed on 6 February 2025)   1. Memorial Sloan Kettering Cancer Center. Breast Cancer Treatment; available from: <https://www.mskcc.org/cancer-conditions/breast-cancer/treatment?utm_source=chatgpt.com>   (accessed on 6 February 2025)   1. [Mayo Clinic Comprehensive Cancer Center Blog](https://cancerblog.mayoclinic.org/). The 4 types of systemic therapy for breast cancer; available from: <https://cancerblog.mayoclinic.org/2024/10/01/the-4-types-of-systemic-therapy-for-breast-cancer/?utm_source=chatgpt.com>   (accessed on 6 February 2025)   1. WebMD. Breast Cancer Treatment; available from: <https://www.webmd.com/BREAST-CANCER/BREAST-CANCER-TREATMENT>   (accessed on 6 February 2025)   1. Healthline. Breast Cancer Treatment Options; available from: <https://www.healthline.com/health/breast-cancer/treatments-for-breast-cancer?utm_source=chatgpt.com>   (accessed on 6 February 2025)   1. Up To Date.com. Patient education: Breast cancer guide to diagnosis and treatment (Beyond the Basics); available from: <https://www.uptodate.com/contents/breast-cancer-guide-to-diagnosis-and-treatment-beyond-the-basics>   (accessed on 6 February 2025)   1. City of Hope Cancer Treatment Centers. Breast cancer treatments; available from: <https://www.cancercenter.com/cancer-types/breast-cancer/treatments>   (accessed on 6 February 2025)   1. MD Anderson Cancer Centers. Breast Cancer Treatment;   available from: <https://www.mdanderson.org/cancer-types/breast-cancer/breast-cancer-treatment.html>  (accessed on 6 February 2025)   1. National Breast Cancer Foundation, Inc. Treatment; available from: <https://www.nationalbreastcancer.org/breast-cancer-treatment/>   (accessed on 6 February 2025) |  |
| 10 | Can breast cancer recur? | | | 1. Mayo Clinic Press. Commonly asked questions about breast cancer recurrence; available from: <https://mcpress.mayoclinic.org/early-breast-cancer/commonly-asked-questions-about-breast-cancer-recurrence/?utm_source=chatgpt.com>   (accessed on 2 February 2025)   1. The Sunday Times. Menipausal Cancer Survivors offered HRT under New Guidance; available from: <https://www.thetimes.com/uk/healthcare/article/menopausal-cancer-survivors-offered-hrt-under-new-guidance-z08bgldlj?utm_source=chatgpt.com&region=global>   (accessed on 2 February 2025)   1. Healthline. A Guide to Understanding Breast Cancer Recurrence Rates; available from: <https://www.healthline.com/health/breast-cancer/recurrence-rates>   (accessed on 6 February 2025)   1. Mayo Clinic. [Recurrent breast cancer](https://www.mayoclinic.org/diseases-conditions/recurrent-breast-cancer/symptoms-causes/syc-20377135) ; available from:   <https://www.mayoclinic.org/diseases-conditions/recurrent-breast-cancer/symptoms-causes/syc-20377135>  (accessed on 6 February 2025)   1. Komen.org. Survival and Risk of Breast Cancer Recurrence After Treatment; available from: <https://www.komen.org/breast-cancer/treatment/recurrence/survival-and-risk-of-recurrence/>   (accessed on 6 February 2025)   1. Komen.org. Breast Cancer Recurrence; available from: <https://www.komen.org/breast-cancer/treatment/recurrence/>   (accessed on 6 February 2025)   1. MedicineNet. Breast Cancer Recurrence; available from:   <https://www.medicinenet.com/breast_cancer_recurrence/article.htm>  (accessed on 6 February 2025)   1. City of Hope Cancer Treatment Centers. Breast cancer recurrence; available from: <https://www.cancercenter.com/cancer-types/breast-cancer/types/rare-breast-cancer-types/recurrent-breast-cancer>   (accessed on 6 February 2025)   1. Verywell health. The Risks of Breast Cancer Recurrence; available from: <https://www.verywellhealth.com/breast-cancer-recurrence-3576787>   (accessed on 6 February 2025)   1. Cleveland Clinic. Breast Cancer Recurrence; available from: <https://my.clevelandclinic.org/health/diseases/8328-breast-cancer-recurrence>   (accessed on 6 February 2025)   1. Johns Hopkins Medicine. Health; available from: <https://www.hopkinsmedicine.org/health/conditions-and-diseases/breast-cancer/breast-cancer-recurrence>   (accessed on 6 February 2025)   1. American Cancer Society. Treatment of Recurrent Breast Cancer; available from: <https://www.cancer.org/cancer/types/breast-cancer/treatment/treatment-of-breast-cancer-by-stage/treatment-of-recurrent-breast-cancer.html>   (accessed on 6 February 2025) |  |
| **DeepSeek** | | | | | |
|  | | Question | Answer | | |
| 1 | | What are the common symptoms of breast cancer? | 1. American Cancer Society. (2023). Breast Cancer Signs and Symptoms. Available from: <https://www.cancer.org/>   (accessed on 7 February 2025)   1. Mayo Clinic. (2023). Breast Cancer Symptoms and Causes. Available from: <https://www.mayoclinic.org/>   (accessed on 7 February 2025)   1. National Breast Cancer Foundation. Inc. (2023). Symptoms of Breast Cancer. Available from: [https://www.nationalbreastcancer.org](https://www.nationalbreastcancer.org/)   (accessed on 7 February 2025) | | |
| 2 | | What are the risk factors for breast cancer? | 1. American Cancer Society. (2023). "Breast Cancer Risk Factors." Available from: <https://www.cancer.org/cancer/types/breast-cancer/risk-and-prevention/breast-cancer-risk-factors-you-cannot-change.html>   (accessed on 7 February 2025)   1. National Cancer Institute. (2023). "Breast Cancer Risk in American Women." Available from: <https://www.cancer.gov/types/breast/risk-fact-sheet>   (accessed on 7 February 2025)   1. World Health Organization (WHO). (2023). "Breast Cancer: Prevention and Control." Available from: <https://www.who.int/activities/preventing-cancer>   (accessed on 7 February 2025)   1. Mayo Clinic. (2023). "Breast Cancer: Risk Factors." Available from: <https://www.mayoclinic.org/diseases-conditions/breast-cancer/symptoms-causes/syc-20352470>   (accessed on 7 February 2025) | | |
| 3 | | How to prevent breast cancer? | 1. Mayo Clinic.org. Breast cancer prevention: How to reduce your risk; available from:   <https://www.mayoclinic.org/healthy-lifestyle/womens-health/in-depth/breast-cancer-prevention/art-20044676>  (accessed on 7 February 2025)   1. Sauter ER. Breast Cancer Prevention: Current Approaches and Future Directions. Eur J Breast Health. 2018 Apr 1;14(2):64-71. doi: 10.5152/ejbh.2018.3978. PMID: 29774312; PMCID: PMC5939980. 2. National Cancer Institute. Breast Cancer Prevention (PDQ®)–Patient Version; available from:   <https://www.cancer.gov/types/breast/patient/breast-prevention-pdq>  (accessed on 7 February 2025)   1. Britt, K.L., Cuzick, J. & Phillips, KA. Key steps for effective breast cancer prevention. *Nat Rev Cancer* **20**, 417–436 (2020). <https://doi.org/10.1038/s41568-020-0266-x> 2. CDC.org. Reducing Risk for Breast Cancer; available from:   <https://www.cdc.gov/breast-cancer/prevention/index.html>  (accessed on 7 February 2025)   1. Healthline. How to Reduce Your Risk of Breast Cancer: 10 Lifestyle Recommendations; available from: <https://www.healthline.com/health/breast-cancer/prevention-for-breast-cancer>   (accessed on 7 February 2025)   1. SITEMAN Cancer Center. 8IGHTWAYS® to Prevent Breast Cancer; available from: <https://siteman.wustl.edu/prevention/8-ways/8-ways-to-prevent-breast-cancer/>   (accessed on 7 February 2025)   1. Howell, A., Anderson, A.S., Clarke, R.B. *et al.* Risk determination and prevention of breast cancer. *Breast Cancer Res* **16**, 446 (2014). <https://doi.org/10.1186/s13058-014-0446-2> 2. Vogel VG. Breast cancer prevention: a review of current evidence. CA Cancer J Clin. 2000 May-Jun;50(3):156-70. doi: 10.3322/canjclin.50.3.156. PMID: 10901739. 3. Breast Cancer UK. 25 tips to reduce your risk of breast cancer;   available from: <https://www.breastcanceruk.org.uk/25-tips-to-reduce-your-risk-of-breast-cancer/>  (accessed on 7 February 2025)   1. The Pan American Health Organization (PAHO).org. Breast Cancer Risk Factors And Prevention; available from: chrome-extension://efaidnbmnnnibpcajpcglclefindmkaj/https://www3.paho.org/hq/   dmdocuments/2016/KNOWLEDGE-SUMMARY---PREVENTION.pdf  (accessed on 7 February 2025)   1. Memorial Sloan Kettering Cancer Center. What You Can Do to Prevent Breast Cancer; available from: <https://www.mskcc.org/cancer-care/patient-education/your-guide-preventing-breast-cancer>   (accessed on 7 February 2025)   1. Vidali, S.; Susini, T. Breast Cancer Risk and Prevention: A Step Forward. Cancers **2023**, 15, 5559. <https://doi.org/10.3390/cancers15235559> 2. [Julie R. Nangia et al.](https://ascopubs.org/action/doSearch?ContribAuthorRaw=Nangia%2C+Julie+R), Breast Cancer Prevention: Where Are We?. *JCO Oncol Pract* **17**, 720-722(2021). DOI:[10.1200/OP.21.00605](https://doi.org/10.1200/OP.21.00605) 3. Prevent Cancer Foundation. Breast Cancer; available from: <https://preventcancer.org/zh/preventable-cancer/breast-cancer/>   (accessed on 7 February 2025)   1. Primary and secondary prevention of breast cancer in China: a scoping review. Tang, Yingyue et al. The Lancet, Volume 394, S76. DOI: [10.1016/S0140-6736(19)32412-2](https://doi.org/10.1016/S0140-6736(19)32412-2) 2. American Cancer Society. Five Ways to Help Reduce Your Breast Cancer Risk; available from: <https://www.cancer.org/cancer/latest-news/five-ways-to-reduce-your-breast-cancer-risk.html>   (accessed on 7 February 2025)   1. [Sandhya Pruthi et al.](https://ascopubs.org/action/doSearch?ContribAuthorRaw=Pruthi%2C+Sandhya), Reducing Breast Cancer Incidence and Mortality: Rethinking an Approach to Risk Assessment and Prevention. *JCO Oncol Pract* **17**, 717-719(2021). DOI:[10.1200/OP.21.00551](https://doi.org/10.1200/OP.21.00551) 2. Breast Cancer Research Foundation (BCRF). 10 Ways to Help Reduce Breast Cancer Risk; available from: <https://www.bcrf.org/blog/breast-cancer-prevention-breast-cancer-risk-reduction/> (accessed on 7 February 2025) 3. American Cancer Society. Can I Lower My Risk of Breast Cancer? available from: <https://www.cancer.org/cancer/types/breast-cancer/risk-and-prevention/can-i-lower-my-risk.html>   (accessed on 7 February 2025)   1. Zaluzec, E.K.; Sempere, L.F. Systemic and Local Strategies for Primary Prevention of Breast Cancer. Cancers **2024**, 16, 248. <https://doi.org/10.3390/cancers16020248> 2. Reeder, J.G., Vogel, V.G. (2008). Breast Cancer Prevention. In: Gradishar, W.J., Wood, W.C. (eds) Advances in Breast Cancer Management, Second Edition. Cancer Treatment and Research, vol 141. Springer, Boston, MA. <https://doi.org/10.1007/978-0-387-73161-2_10> 3. Mayo Clinic.org. How to reduce the risk of breast cancer risk; available from: <https://mcpress.mayoclinic.org/women-health/how-to-reduce-the-risk-of-breast-cancer/>   (accessed on 7 February 2025)   1. Li, X., Zhang, H., Yang, H., Song, Y., Zhang, F. and Wang, A. (2025), Modifiable Risk Factors for Breast Cancer: Insights from Systematic Reviews. Public Health Nurs. <https://doi.org/10.1111/phn.13504> 2. WikiHow. [How to Prevent Breast Cancer](https://www.wikihow.health/Prevent-Breast-Cancer); available from: <https://www.wikihow.health/Prevent-Breast-Cancer> (accessed on 7 February 2025) 3. Advani, P., & Moreno-Aspitia, A. (2014). Current strategies for the prevention of breast cancer. *Breast Cancer: Targets and Therapy*, *6*, 59–71. <https://doi.org/10.2147/BCTT.S39114> 4. WebMD. Understanding Breast Cancer – Prevention; available from: <https://www.webmd.com/breast-cancer/understanding-breast-cancer-prevention> (accessed on 7 February 2025) 5. Woman’sWorld.com. Doctors Reveal 8 Easy Ways to Lower Your Breast Cancer Risk—With These Simple Habits; available from: <https://www.womansworld.com/wellness/cancer/habits-to-decrease-risk-of-breast-cancer> (accessed on 7 February 2025) 6. Prevent Cancer Foundation. 6 Things You Can Do to Reduce Your Risk of Breast Cancer; available from: <https://preventcancer.org/zh/article/6-things-you-can-do-now-to-reduce-your-breast-cancer-risk/> (accessed on 7 February 2025) 7. Farina S, Osti T, Russo L, Maio A, Scarsi N, Savoia C, et al. (2025) The current landscape of personalised preventive approaches for non-communicable diseases: A scoping review. PLoS ONE 20(1): e0317379. <https://doi.org/10.1371/journal.pone.0317379> 8. National Cancer Institute. Breast Cancer Screening (PDQ®)– Health Professional Version; available from: <https://www.cancer.gov/types/breast/hp/breast-prevention-pdq>   (accessed on 7 February 2025)   1. Atale, N.; Wells, A. Statins as Secondary Preventive Agent to Limit Breast Cancer Metastatic Outgrowth. Int. J. Mol. Sci. **2025**, 26, 1300. <https://doi.org/10.3390/ijms26031300> 2. DOCTOR NDTV. Lifestyle Tips For Women To Prevent Cancer Risk; available from: <https://doctor.ndtv.com/cancer/lifestyle-tips-for-women-to-prevent-cancer-risk-7655694>   (accessed on 7 February 2025)   1. Noman, S.M., Fadel, Y.M., Henedak, M.T. *et al.* Leveraging survival analysis and machine learning for accurate prediction of breast cancer recurrence and metastasis. *Sci Rep* **15**, 3728 (2025). <https://doi.org/10.1038/s41598-025-87622-3> 2. American Cancer Society. Breast Cancer Risk and Prevention; available from: <https://www.cancer.org/cancer/types/breast-cancer/risk-and-prevention.html>   (accessed on 7 February 2025)   1. The Healthy. 50 Everyday Habits That Reduce Your Risk of Breast Cancer; available from: <https://www.thehealthy.com/cancer/breast-cancer/habits-that-reduce-risk-of-breast-cancer/> (accessed on 7 February 2025) 2. Kuszaj, O., Day, M., Tse, S.S.W. *et al.* A critical review of randomized controlled trials on topical corticosteroids for the prevention of radiation dermatitis in breast cancer. *Support Care Cancer* **33**, 147 (2025). <https://doi.org/10.1007/s00520-025-09178-2> 3. Canadian Living.com. 12 ways to help reduce your breast cancer risk; available from: <https://www.canadianliving.com/health-fitness/prevention-and-recovery/article/12-ways-to-prevent-breast-cancer> (accessed on 7 February 2025) 4. Thorat MA, Balasubramanian R. Breast cancer prevention in high-risk women. Best Pract Res Clin Obstet Gynaecol. 2020 May;65:18-31. doi: 10.1016/j.bpobgyn.2019.11.006. Epub 2019 Nov 21. PMID: 31862315. 5. Jiao, D., Chen, X., Sun, X. *et al.* A comparative study of mitoxantrone hydrochloride injection and technetium-99 m for tracing axillary sentinel nodes in patients with breast cancer. *BMC Cancer* **25**, 209 (2025). <https://doi.org/10.1186/s12885-025-13618-x> 6. Chlebowski RT, Aragaki AK, Pan K, et al. Breast cancer incidence and mortality by metabolic syndrome and obesity: The Women’s Health Initiative. *Cancer*. 2024; 130(18): 3147-3156. doi:[10.1002/cncr.35318](https://doi.org/10.1002/cncr.35318) 7. Ferraro E, Reiner AS, Bou Nassif R, et al. Survival Among Patients With *ERBB2*-Positive Metastatic Breast Cancer and Central Nervous System Disease. JAMA Netw Open. 2025;8(1):e2457483. doi:10.1001/jamanetworkopen.2024.57483 8. Kolak A, Kamińska M, Sygit K, Budny A, Surdyka D, Kukiełka-Budny B, Burdan F. Primary and secondary prevention of breast cancer. Ann Agric Environ Med. 2017 Dec 23;24(4):549-553. doi: 10.26444/aaem/75943. Epub 2017 Jul 18. PMID: 29284222.   Goonaratne, E., Ahad, A., Al-Khaifi, M. (2025). Survivorship Needs in Breast Cancer Patients with Disabilities. In: Bennett, G., Goodall, E. (eds) The Palgrave Encyclopedia of Disability. Palgrave Macmillan, Cham. <https://doi.org/10.1007/978-3-031-40858-8_377-1> | | |
| 4 | | Can breast cancer be inherited? | 1. Cancer Research UK. Family history of breast cancer and inherited genes; available from: <https://www.cancerresearchuk.org/about-cancer/breast-cancer/risks-causes/family-history-and-inherited-genes>   (accessed on 7 February 2025)   1. Larsen MJ, Thomassen M, Gerdes AM, Kruse TA. Hereditary breast cancer: clinical, pathological and molecular characteristics. Breast Cancer (Auckl). 2014 Oct 15;8:145-55. doi: 10.4137/BCBCR.S18715. PMID: 25368521; PMCID: PMC4213954. 2. Cleveland Clinic. Breast Cancer Can Be Genetic: Here’s What To Know; available from: <https://health.clevelandclinic.org/is-breast-cancer-hereditary>   (accessed on 7 February 2025)   1. Jia, G., Chen, Z., Ping, J. *et al.* Refining breast cancer genetic risk and biology through multi-ancestry fine-mapping analyses of 192 risk regions. *Nat Genet* **57**, 80–87 (2025). <https://doi.org/10.1038/s41588-024-02031-y> 2. MD Anderson Cancer Centers. Is breast cancer genetic? <https://www.mdanderson.org/cancerwise/does-breast-cancer-run-in-families.h00-159695967.html>   (accessed on 7 February 2025)   1. Pal M, Das D, Pandey M. Understanding genetic variations associated with familial breast cancer. World J Surg Oncol. 2024 Oct 10;22(1):271. doi: 10.1186/s12957-024-03553-9. PMID: 39390525; PMCID: PMC11465949. 2. Gauvin, K., Allain, V., Bouhamdani, N., Williams, C., Saheb, Y., Savoie, C., Macrae, L., Hodson, K., Zhu, Y.A., Allain, E. and Amor, M.B. (2025), Retrospective Study of Genetic Testing Results Reveals Pathogenic Variants Beyond BRCA1/2 in Hereditary Breast and Ovarian Cancer Cases in New Brunswick: Implications for Future Care. Cancer Med, 14: e70640. <https://doi.org/10.1002/cam4.70640> 3. Cancer Research UK. Family history and inherited cancer genes; available from:   <https://www.cancerresearchuk.org/about-cancer/causes-of-cancer/inherited-cancer-genes-and-increased-cancer-risk/family-history-and-inherited-cancer-genes> (accessed on 7 February 2025)   1. HealthPartners.com. Hereditary breast cancer: Understanding your personal risk; available from: <https://www.healthpartners.com/blog/is-breast-cancer-genetic/>   (accessed on 7 February 2025)   1. Australian Government Cancer Australia. Family history and genetics; available from: <https://www.canceraustralia.gov.au/breast-cancer-risk-factors/risk-factors/family-history-and-genetics>   (accessed on 7 February 2025)   1. Breast Cancer Research Foundation (BCRF). Hereditary Breast Cancer and Inherited Risk Factors: Ongoing Areas of Focus for BCRF; available from: <https://www.bcrf.org/blog/inherited-risk-factors-and-hereditary-breast-cancer-ongoing-areas-of-focus-for-bcrf/>   (accessed on 7 February 2025)   1. Barili, V.; Ambrosini, E.; Bortesi, B.; Minari, R.; De Sensi, E.; Cannizzaro, I.R.; Taiani, A.; Michiara, M.; Sikokis, A.; Boggiani, D.; et al. Genetic Basis of Breast and Ovarian Cancer: Approaches and Lessons Learnt from Three Decades of Inherited Predisposition Testing. Genes **2024**, 15, 219. <https://doi.org/10.3390/genes15020219> 2. Li, T., Zhang, H., Lian, M. *et al.* Global status and attributable risk factors of breast, cervical, ovarian, and uterine cancers from 1990 to 2021. *J Hematol Oncol* **18**, 5 (2025). <https://doi.org/10.1186/s13045-025-01660-y> 3. Australian Government Cancer Australia. What are the risk factors for breast cancer? available from: <https://www.canceraustralia.gov.au/cancer-types/breast-cancer/what-are-risk-factors-breast-cancer> (accessed on 7 February 2025) 4. Apostolou P, Fostira F. Hereditary breast cancer: the era of new susceptibility genes. Biomed Res Int. 2013;2013:747318. doi: 10.1155/2013/747318. Epub 2013 Mar 21. PMID: 23586058; PMCID: PMC3618918. 5. Walsh, T.; King, M.-C. Ten Genes for Inherited Breast Cancer. *Cancer Cell* **2007**, *11*, 103-105, doi:10.1016/j.ccr.2007.01.010. 6. Ding, L., Chen, B., Zhou, Z. *et al.* Exploring the genetic correlation and causal relationships between breast cancer and meningioma using bidirectional Mendelian randomization. *Sci Rep* **15**, 4271 (2025). <https://doi.org/10.1038/s41598-025-88829-0> 7. Yoshida, R. Hereditary breast and ovarian cancer (HBOC): review of its molecular characteristics, screening, treatment, and prognosis. *Breast Cancer* **28**, 1167–1180 (2021). <https://doi.org/10.1007/s12282-020-01148-2> 8. Wendt, C.; Margolin, S. Identifying breast cancer susceptibility genes – a review of the genetic background in familial breast cancer. *Acta Oncologica* **2019**, *58*, 135-146, doi:10.1080/0284186X.2018.1529428. 9. Singh, T., Rastogi, M. & Thakur, K. Network pharmacology and in silico approach to study the mechanism of quercetin against breast cancer. *In Silico Pharmacol.* **13**, 22 (2025). <https://doi.org/10.1007/s40203-025-00306-8> 10. Hartman, M.; Lindström, L.; Dickman, P.W.; Adami, H.O.; Hall, P.; Czene, K. Is breast cancer prognosis inherited? *Breast Cancer Res* **2007**, *9*, R39, doi:10.1186/bcr1737. 11. Radford DM, Zehnbauer BA. Inherited breast cancer. Surg Clin North Am. 1996 Apr;76(2):205-20. doi: 10.1016/s0039-6109(05)70434-5. PMID: 8610259. 12. Apessos, A., Nasioulas, G. (2021). Breast Cancer Genetics. In: Rezai, M., Kocdor, M.A., Canturk, N.Z. (eds) Breast Cancer Essentials. Springer, Cham. <https://doi.org/10.1007/978-3-030-73147-2_3> 13. Zhang, LY., Chen, XT., Li, RT. *et al.* Overcoming hypoxia-induced breast cancer drug resistance: a novel strategy using hollow gold-platinum bimetallic nanoshells. *J Nanobiotechnol* **23**, 85 (2025). <https://doi.org/10.1186/s12951-025-03132-4> 14. Guo Y, Arciero CA, Jiang R, Behera M, Peng L, Li X. Different Breast Cancer Subtypes Show Different Metastatic Patterns: A Study from A Large Public Database. Asian Pac J Cancer Prev. 2020 Dec 1;21(12):3587-3593. doi: 10.31557/APJCP.2020.21.12.3587. PMID: 33369456; PMCID: PMC8046324. 15. American Cancer Society. Breast Cancer Risk Factors You Cannot Change; available from: <https://www.cancer.org/cancer/types/breast-cancer/risk-and-prevention/breast-cancer-risk-factors-you-cannot-change.html>   (accessed on 7 February 2025)   1. Haddad, C.F., 2020. Hereditary breast cancer: review and current approach. *Mastology*, *30*, pp.1-11. <http://doi.org/10.29289/25945394202020200042> 2. Chen, Z., Liu, Y., Lyu, M. *et al.* Classifications of triple-negative breast cancer: insights and current therapeutic approaches. *Cell Biosci* **15**, 13 (2025). <https://doi.org/10.1186/s13578-025-01359-0> 3. Cupertino, S.E.S.; Gonçalves, A.C.A.; Gusmão Lopes, C.V.; Gradia, D.F.; Beltrame, M.H. The Current State of Breast Cancer Genetics in Populations of African Ancestry. Genes **2025**, 16, 199. <https://doi.org/10.3390/genes16020199> 4. van der Groep, P., van der Wall, E. & van Diest, P.J. Pathology of hereditary breast cancer. *Cell Oncol.* **34**, 71–88 (2011). <https://doi.org/10.1007/s13402-011-0010-3> 5. Militello, C. AI Applied to Breast Cancer: Early Detection and Explainable Predictive Models as the Basis of Precision Medicine. *Academic Radiology*, doi:10.1016/j.acra.2025.01.045. 6. AstraZeneca. Enhertu approved in the US as first HER2-directed therapy for patients with HER2-low or HER2-ultralow metastatic breast cancer following disease progression after one or more endocrine therapies; available from: <https://www.astrazeneca.com/media-centre/press-releases/2025/enhertu-approved-in-us-for-breast-cancer-post-et.html>   (accessed on 7 February 2025)   1. Muller, L., Fauvet, F., Chassot, C. *et al.* EMT-driven plasticity prospectively increases cell–cell variability to promote therapeutic adaptation in breast cancer. *Cancer Cell Int* **25**, 32 (2025). <https://doi.org/10.1186/s12935-025-03637-w> 2. American Cancer Society. Causes, Risk Factors, and Prevention of Cancer in Children; available from: <https://www.cancer.org/cancer/childhood-cancer/causes-risk-factors-prevention.html> (accessed on 7 February 2025) 3. Myers, C., Bennett, K., Cahir, C. *et al.* Exploring health related quality of life for women with breast cancer in Ireland and Québec, Canada throughout the COVID-19 pandemic. *Sci Rep* **15**, 4010 (2025). <https://doi.org/10.1038/s41598-024-84852-9> 4. Urology Times. Pathogenic variants of WNT9B gene linked to hereditary prostate cancer; available from:   <https://www.urologytimes.com/view/pathogenic-variants-of-wnt9b-gene-linked-to-hereditary-prostate-cancer>  (accessed on 7 February 2025) | | |
| 5 | | Why is breast cancer screening important? | 1. Fuller MS, Lee CI, Elmore JG. Breast cancer screening: an evidence-based update. Med Clin North Am. 2015 May;99(3):451-68. doi: 10.1016/j.mcna.2015.01.002. Epub 2015 Mar 5. PMID: 25841594; PMCID: PMC5064844. 2. Ren W, Chen M, Qiao Y, Zhao F. Global guidelines for breast cancer screening: A systematic review. Breast. 2022 Aug;64:85-99. doi: 10.1016/j.breast.2022.04.003. Epub 2022 Apr 19. PMID: 35636342; PMCID: PMC9142711. 3. Narayan AK, Lee CI, Lehman CD. Screening for Breast Cancer. Med Clin North Am. 2020 Nov;104(6):1007-1021. doi: 10.1016/j.mcna.2020.08.003. PMID: 33099447. 4. Nayyar S, Chakole S, Taksande AB, Prasad R, Munjewar PK, Wanjari MB. From Awareness to Action: A Review of Efforts to Reduce Disparities in Breast Cancer Screening. Cureus. 2023 Jun 20;15(6):e40674. doi: 10.7759/cureus.40674. PMID: 37485176; PMCID: PMC10359048. 5. Berg WA. Benefits of screening mammography. JAMA. 2010 Jan 13;303(2):168-9. doi: 10.1001/jama.2009.1993. PMID: 20068213; PMCID: PMC3891888. 6. Ren, W.; Chen, M.; Qiao, Y.; Zhao, F. Global guidelines for breast cancer screening: A systematic review. *Breast* **2022**, *64*, 85-99, doi:10.1016/j.breast.2022.04.003. 7. Tomlinson-Hansen SE, Budh DP, Sapra A. Breast Cancer Screening in the Average-Risk Patient. [Updated 2024 Oct 3]. In: StatPearls [Internet]. Treasure Island (FL): StatPearls Publishing; 2025 Jan-. Available from: <https://www.ncbi.nlm.nih.gov/books/NBK556050/> 8. Gemignani ML. Breast cancer screening: why, when, and how many? Clin Obstet Gynecol. 2011 Mar;54(1):125-32. doi: 10.1097/GRF.0b013e318208020d. PMID: 21278511. 9. Katsika L, Boureka E, Kalogiannidis I, Tsakiridis I, Tirodimos I, Lallas K, Tsimtsiou Z, Dagklis T. Screening for Breast Cancer: A Comparative Review of Guidelines. Life (Basel). 2024 Jun 19;14(6):777. doi: 10.3390/life14060777. PMID: 38929759; PMCID: PMC11204612. 10. [Mayo Clinic Comprehensive Cancer Center Blog](https://cancerblog.mayoclinic.org/). The importance of breast cancer screening; available from: <https://cancerblog.mayoclinic.org/2025/01/02/the-importance-of-breast-cancer-screening/> (accessed on 7 February 2025) 11. Hernström, V.; Josefsson, V.; Sartor, H.; Schmidt, D.; Larsson, A.-M.; Hofvind, S.; Andersson, I.; Rosso, A.; Hagberg, O.; Lång, K. Screening performance and characteristics of breast cancer detected in the Mammography Screening with Artificial Intelligence trial (MASAI): a randomised, controlled, parallel-group, non-inferiority, single-blinded, screening accuracy study. *The Lancet Digital Health*, doi:10.1016/S2589-7500(24)00267-X. 12. Gordon PB. Breast cancer screening. Can Fam Physician. 2019 Jul;65(7):457-459. PMID: 31300424; PMCID: PMC6738458. 13. De Jesus, C., Moseley, T.W., Diaz, V. *et al.* The Benefits of Screening Mammography. *Curr Breast Cancer Rep* **15**, 103–107 (2023). <https://doi.org/10.1007/s12609-023-00479-1> 14. IARC Working Group on the Evaluation of Cancer-Preventive Interventions. Breast cancer screening. Lyon (FR): International Agency for Research on Cancer; 2016. 4. Efficacy of Breast Cancer Screening. Available from: <https://www.ncbi.nlm.nih.gov/books/NBK546555/> 15. Katsika, L.; Boureka, E.; Kalogiannidis, I.; Tsakiridis, I.; Tirodimos, I.; Lallas, K.; Tsimtsiou, Z.; Dagklis, T. Screening for Breast Cancer: A Comparative Review of Guidelines. Life **2024**, 14, 777. <https://doi.org/10.3390/life14060777> 16. Fuller MS, Lee CI, Elmore JG. Breast cancer screening: an evidence-based update. Med Clin North Am. 2015 May;99(3):451-68. doi: 10.1016/j.mcna.2015.01.002. Epub 2015 Mar 5. PMID: 25841594; PMCID: PMC5064844. 17. Tavakoli, B., Feizi, A., Zamani-Alavijeh, F. *et al.* Factors influencing breast cancer screening practices among women worldwide: a systematic review of observational and qualitative studies. *BMC Women's Health* **24**, 268 (2024). <https://doi.org/10.1186/s12905-024-03096-x> 18. Cleveland Clinic. Breast Cancer Screenings; available from: <https://my.clevelandclinic.org/health/diagnostics/breast-cancer-screenings> (accessed on 7 February 2025) 19. Cancer Research UK. Breast Screening; available from: <https://www.cancerresearchuk.org/health-professional/cancer-screening/breast-cancer-screening>   (accessed on 7 February 2025)   1. Swapana M, Padmavathy C. A critical review on breast cancer literature: Screening, awareness and preventive measures. Mediterranean Journal of Social Sciences. 2015 Aug;6(4):256. 2. American Cancer Society. American Cancer Society Recommendations for the Early Detection of Breast Cancer; available from: <https://www.cancer.org/cancer/types/breast-cancer/screening-tests-and-early-detection/american-cancer-society-recommendations-for-the-early-detection-of-breast-cancer.html>   (accessed on 7 February 2025)   1. Mayo Clinic.org. Mayo Clinic Minute: Why breast cancer screening is important; available from: <https://newsnetwork.mayoclinic.org/discussion/mayo-clinic-minute-why-breast-cancer-screening-is-important/>   (accessed on 7 February 2025)   1. Onishi, N., Kataoka, M. Breast cancer screening for women at high risk: review of current guidelines from leading specialty societies. *Breast Cancer* **28**, 1195–1211 (2021). <https://doi.org/10.1007/s12282-020-01157-1> 2. Verywell Health. Ask the Expert: Why Is Breast Cancer Screening So Important? available from: <https://www.verywellhealth.com/ask-the-expert-what-is-the-importance-of-breast-cancer-screening-6740783> 3. Glasziou P, Houssami N. The evidence base for breast cancer screening. Prev Med. 2011 Sep;53(3):100-2. doi: 10.1016/j.ypmed.2011.05.011. Epub 2011 Jun 2. PMID: 21658406. 4. Saldanha, O.L., Zhu, J., Müller-Franzes, G. *et al.* Swarm learning with weak supervision enables automatic breast cancer detection in magnetic resonance imaging. *Commun Med* **5**, 38 (2025). <https://doi.org/10.1038/s43856-024-00722-5> 5. Cancerscreenweek.org. Why is cancer screening important? available from: <https://www.cancerscreenweek.org/content/dam/gene/cancerscreenweek/>   resources/cancer-screening-fact-sheet-english.pdf   1. Ferraro E, Reiner AS, Bou Nassif R, et al. Survival Among Patients With *ERBB2*-Positive Metastatic Breast Cancer and Central Nervous System Disease. JAMA Netw Open. 2025;8(1):e2457483. doi:10.1001/jamanetworkopen.2024.57483 2. Smith RA, Oeffinger KC. The Importance of Cancer Screening. Med Clin North Am. 2020 Nov;104(6):919-938. doi: 10.1016/j.mcna.2020.08.008. Epub 2020 Sep 16. PMID: 33099452. 3. Abdullah, K.A., Marziali, S., Nanaa, M. *et al.* Deep learning-based breast cancer diagnosis in breast MRI: systematic review and meta-analysis. *Eur Radiol* (2025). <https://doi.org/10.1007/s00330-025-11406-6> 4. PR Newswire. Early detection is a game changer: Novartis makes bold play to prioritize breast health and inspire a new wave of breast cancer screenings 5. Rjoop, W., Rjoop, A., Almohtaseb, A. *et al.* Pathological and radiological assessment of benign breast lesions with BIRADS IVc/V subtypes. should we repeat the biopsy?. *BMC Women's Health* **25**, 47 (2025). <https://doi.org/10.1186/s12905-025-03569-7> 6. Chen, Z., Liu, Y., Lyu, M. *et al.* Classifications of triple-negative breast cancer: insights and current therapeutic approaches. *Cell Biosci* **15**, 13 (2025). <https://doi.org/10.1186/s13578-025-01359-0> 7. Independent UK Panel on Breast Cancer Screening. The benefits and harms of breast cancer screening: an independent review. Lancet. 2012 Nov 17;380(9855):1778-86. doi: 10.1016/S0140-6736(12)61611-0. Epub 2012 Oct 30. PMID: 23117178. | | |
| 6 | | What are the risks of breast cancer screening? | 1. CDC.org. Screening for breast cancer; available from: <https://www.cdc.gov/breast-cancer/screening/index.html>   (accessed on 7 February 2025)   1. Xu H, Xu B. Breast cancer: Epidemiology, risk factors and screening. Chin J Cancer Res. 2023 Dec 30;35(6):565-583. doi: 10.21147/j.issn.1000-9604.2023.06.02. PMID: 38204449; PMCID: PMC10774137. 2. National Cancer Institute. Breast Cancer Screening (PDQ®)–Patient Version; available from: <https://www.cancer.gov/types/breast/patient/breast-screening-pdq>   (accessed on 7 February 2025)   1. Ren W, Chen M, Qiao Y, Zhao F. Global guidelines for breast cancer screening: A systematic review. Breast. 2022 Aug;64:85-99. doi: 10.1016/j.breast.2022.04.003. Epub 2022 Apr 19. PMID: 35636342; PMCID: PMC9142711. 2. Clift, A.K., Dodwell, D., Lord, S. *et al.* The current status of risk-stratified breast screening. *Br J Cancer* **126**, 533–550 (2022). <https://doi.org/10.1038/s41416-021-01550-3> 3. Fuller MS, Lee CI, Elmore JG. Breast cancer screening: an evidence-based update. Med Clin North Am. 2015 May;99(3):451-68. doi: 10.1016/j.mcna.2015.01.002. Epub 2015 Mar 5. PMID: 25841594; PMCID: PMC5064844. 4. Tomlinson-Hansen SE, Budh DP, Sapra A. Breast Cancer Screening in the Average-Risk Patient. [Updated 2024 Oct 3]. In: StatPearls [Internet]. Treasure Island (FL): StatPearls Publishing; 2025 Jan-. Available from: <https://www.ncbi.nlm.nih.gov/books/NBK556050/> 5. Autier P, Jørgensen KJ, Smans M, Støvring H. Effect of screening mammography on the risk of breast cancer deaths and of all-cause deaths: a systematic review with meta-analysis of cohort studies. J Clin Epidemiol. 2024 Aug;172:111426. doi: 10.1016/j.jclinepi.2024.111426. Epub 2024 Jun 13. PMID: 38878837. 6. Ren W, Chen M, Qiao Y, Zhao F. Global guidelines for breast cancer screening: A systematic review. Breast. 2022 Aug;64:85-99. doi: 10.1016/j.breast.2022.04.003. Epub 2022 Apr 19. PMID: 35636342; PMCID: PMC9142711. 7. Pace LE, Keating NL. A systematic assessment of benefits and risks to guide breast cancer screening decisions. JAMA. 2014 Apr 2;311(13):1327-35. doi: 10.1001/jama.2014.1398. PMID: 24691608. 8. Up to Date.cn. Screening for breast cancer: Evidence for effectiveness and harms; available from:   <https://www.uptodate.cn/contents/screening-for-breast-cancer-evidence-for-effectiveness-and-harms>  (accessed on 7 February 2025)   1. Lee CI, Chen LE, Elmore JG. Risk-based Breast Cancer Screening: Implications of Breast Density. Med Clin North Am. 2017 Jul;101(4):725-741. doi: 10.1016/j.mcna.2017.03.005. PMID: 28577623; PMCID: PMC5458625. 2. Tomlinson-Hansen SE, Budh DP, Sapra A. Breast Cancer Screening in the Average-Risk Patient. 2024 Oct 3. In: StatPearls [Internet]. Treasure Island (FL): StatPearls Publishing; 2025 Jan–. PMID: 32310510. 3. American Cancer Society. American Cancer Society Recommendations for the Early Detection of Breast Cancer; available from: <https://www.cancer.org/cancer/types/breast-cancer/screening-tests-and-early-detection/american-cancer-society-recommendations-for-the-early-detection-of-breast-cancer.html>   (accessed on 7 February 2025)   1. Onishi, N., Kataoka, M. Breast cancer screening for women at high risk: review of current guidelines from leading specialty societies. *Breast Cancer* **28**, 1195–1211 (2021). <https://doi.org/10.1007/s12282-020-01157-1> 2. Usher-Smith, J.A., Hindmarch, S., French, D.P. *et al.* Proactive breast cancer risk assessment in primary care: a review based on the principles of screening. *Br J Cancer* **128**, 1636–1646 (2023). <https://doi.org/10.1038/s41416-023-02145-w> 3. National Breast Cancer Foundation, Inc. Breast Cancer Screening; available from: <https://www.nationalbreastcancer.org/breast-cancer-screening/>   (accessed on 7 February 2025)   1. Diwanji, D.; Joe, B. An Evidence-based and Inclusive Breast Cancer Screening Strategy: Summary of Current Guidelines from the American College of Radiology and Society of Breast Imaging. *Radiology: Imaging Cancer* **2022**, *4*, e229003, doi:10.1148/rycan.229003. 2. Cleveland Clinic. Breast Cancer Screenings; available from: <https://my.clevelandclinic.org/health/diagnostics/breast-cancer-screenings> (accessed on 7 February 2025)   Swapana M, Padmavathy C. A critical review on breast cancer literature: Screening, awareness and preventive measures. Mediterranean Journal of Social Sciences. 2015 Aug;6(4):256   1. Monticciolo, D.L.; Newell, M.S.; Moy, L.; Lee, C.S.; Destounis, S.V. Breast Cancer Screening for Women at Higher-Than-Average Risk: Updated Recommendations From the ACR. *Journal of the American College of Radiology* **2023**, *20*, 902-914, doi:10.1016/j.jacr.2023.04.002. 2. The American College of Obstetricians and Gynecologists (ACOG). Breast Cancer Risk Assessment and Screening in Average-Risk Women; available from: <https://www.acog.org/clinical/clinical-guidance/practice-bulletin/articles/2017/07/breast-cancer-risk-assessment-and-screening-in-average-risk-women>   (accessed on 7 February 2025)   1. Hernström, V.; Josefsson, V.; Sartor, H.; Schmidt, D.; Larsson, A.-M.; Hofvind, S.; Andersson, I.; Rosso, A.; Hagberg, O.; Lång, K. Screening performance and characteristics of breast cancer detected in the Mammography Screening with Artificial Intelligence trial (MASAI): a randomised, controlled, parallel-group, non-inferiority, single-blinded, screening accuracy study. *The Lancet Digital Health*, doi:10.1016/S2589-7500(24)00267-X. 2. National Institute for Health and Care Excellence (NICE). Familial breast cancer: classification, care and managing breast cancer and related risks in people with a family history of breast cancer; available from: <https://www.nice.org.uk/guidance/cg164> (accessed on 7 February 2025) 3. McWilliams, L., Roux, A., Hawkes, R., Cholerton, R., Delattre, H., … Bernoux, A. (2024). Women’s experiences of risk-stratified breast cancer screening in the MyPeBS trial: a qualitative comparative study across two European countries. *Psychology & Health*, 1–23. <https://doi.org/10.1080/08870446.2024.2395856> 4. Xu H, Xu B. Breast cancer: Epidemiology, risk factors and screening. Chin J Cancer Res. 2023 Dec 30;35(6):565-583. doi: 10.21147/j.issn.1000-9604.2023.06.02. PMID: 38204449; PMCID: PMC10774137. 5. Katsika, L.; Boureka, E.; Kalogiannidis, I.; Tsakiridis, I.; Tirodimos, I.; Lallas, K.; Tsimtsiou, Z.; Dagklis, T. Screening for Breast Cancer: A Comparative Review of Guidelines. Life **2024**, 14, 777. <https://doi.org/10.3390/life14060777>   NHS. How to decide if you want breast screening; available from: <https://www.nhs.uk/conditions/breast-screening-mammogram/how-to-decide-if-you-want-breast-screening/> (accessed on 7 February 2025)   1. National Cancer Institute. Breast Cancer Risk Assessment Tool: Online Calculator (The Gail Model); available from: <https://bcrisktool.cancer.gov/>   (accessed on 7 February 2025)   1. Canelo-Aybar C, Ferreira DS, Ballesteros M, Posso M, Montero N, Solà I, Saz-Parkinson Z, Lerda D, Rossi PG, Duffy SW, Follmann M, Gräwingholt A, Alonso-Coello P. Benefits and harms of breast cancer mammography screening for women at average risk of breast cancer: A systematic review for the European Commission Initiative on Breast Cancer. J Med Screen. 2021 Dec;28(4):389-404. doi: 10.1177/0969141321993866. Epub 2021 Feb 25. PMID: 33632023. 2. Aliu, A.E., Kerrison, R.S. and Marcu, A. (2025), A Systematic Review of Barriers to Breast Cancer Screening, and of Interventions Designed to Increase Participation, Among Women of Black African and Black Caribbean Descent in the UK. Psycho-Oncology, 34: e70093. <https://doi.org/10.1002/pon.70093> 3. Foshag, K.; Tsiouris, A.J.; Prince, M.; Reichman, M. A review of gadolinium-based contrast agents in the setting of repeated MRI for high risk breast cancer screening. *Clinical Imaging*, doi:10.1016/j.clinimag.2025.110420. 4. Tan, N.Q.P.; Nargund, R.S.; Douglas, E.E.; Lopez-Olivo, M.A.; Resong, P.J.; Ishizawa, S.; Nofal, S.; Krause, K.; Volk, R.J.; Toumazis, I. Acceptability and perceptions of personalised risk-based cancer screening among health-care professionals and the general public: a systematic review and meta-analysis. *The Lancet Public Health* **2025**, *10*, e85-e96, doi:10.1016/S2468-2667(24)00278-0. 5. Mann, R.M. Rethinking surveillance after breast cancer. *The Lancet* **2025**, *405*, 356-358, doi:10.1016/S0140-6736(25)00093-5. 6. Medical Xpress. AI-supported breast cancer screening—new results suggest even higher accuracy; available from: <https://medicalxpress.com/news/2025-02-ai-breast-cancer-screening-results.html> (accessed on 7 February 2025) 7. The Royal Australian College of General Practitioners (RACGP). **Cancer** \| Breast cancer Screening and case finding age bar; available from: <https://www.racgp.org.au/clinical-resources/clinical-guidelines/key-racgp-guidelines/view-all-racgp-guidelines/preventive-activities-in-general-practice/cancer/breast-cancer> (accessed on 7 February 2025) 8. Komen.org. 2025 Super Bowl Ad Highlights Breast Screening Importance; available from: <https://www.komen.org/blog/super-bowl-ad-highlights-breast-cancer-screening-importance/> (accessed on 7 February 2025) 9. Neal CH, Helvie MA. Overdiagnosis and Risks of Breast Cancer Screening. Radiol Clin North Am. 2021 Jan;59(1):19-27. doi: 10.1016/j.rcl.2020.09.005. PMID: 33222997. 10. Naturopathic Doctor News & Review (NDNR). Advancing Breast Cancer Prevention: The Shift to Risk-Based Screening; available from: <https://ndnr.com/womens-health/individualized-breast-cancer-screening-prevention/> (accessed on 7 February 2025) 11. Firstpost. India screens 146 mn women for breast cancer; health minister says 57,000 diagnosed, 50,000 receiving treatment; available from: <https://www.firstpost.com/india/india-screens-146-mn-women-for-breast-cancer-health-minister-says-57000-diagnosed-50000-receiving-treatment-13860663.html> (accessed on 7 February 2025) 12. Australian Government Cancer Australia. What are the risk factors for breast cancer? available from: <https://www.canceraustralia.gov.au/cancer-types/breast-cancer/what-are-risk-factors-breast-cancer> (accessed on 7 February 2025) 13. Science Daily. New screening device shows promise for early lung cancer detection; available from: <https://www.sciencedaily.com/releases/2025/02/250206113328.htm>   (accessed on 7 February 2025)   1. Saldanha, O.L., Zhu, J., Müller-Franzes, G. *et al.* Swarm learning with weak supervision enables automatic breast cancer detection in magnetic resonance imaging. *Commun Med* **5**, 38 (2025). https://doi.org/10.1038/s43856-024-00722-5 | | |
| 7 | | What are the common screening methods for breast cancer? | 1. National Cancer Institute. Breast Cancer Prevention (PDQ®)–Patient Version; available from:   <https://www.cancer.gov/types/breast/patient/breast-prevention-pdq>  (accessed on 7 February 2025)   1. Ren W, Chen M, Qiao Y, Zhao F. Global guidelines for breast cancer screening: A systematic review. Breast. 2022 Aug;64:85-99. doi: 10.1016/j.breast.2022.04.003. Epub 2022 Apr 19. PMID: 35636342; PMCID: PMC9142711. 2. Cleveland Clinic. Breast Cancer Screenings; available from: <https://my.clevelandclinic.org/health/diagnostics/breast-cancer-screenings> (accessed on 7 February 2025) 3. CDC.org. Screening for breast cancer; available from: <https://www.cdc.gov/breast-cancer/screening/index.html>   (accessed on 7 February 2025)   1. Fuller MS, Lee CI, Elmore JG. Breast cancer screening: an evidence-based update. Med Clin North Am. 2015 May;99(3):451-68. doi: 10.1016/j.mcna.2015.01.002. Epub 2015 Mar 5. PMID: 25841594; PMCID: PMC5064844. 2. He Z, Chen Z, Tan M, Elingarami S, Liu Y, Li T, Deng Y, He N, Li S, Fu J, Li W. A review on methods for diagnosis of breast cancer cells and tissues. Cell Prolif. 2020 Jul;53(7):e12822. doi: 10.1111/cpr.12822. Epub 2020 Jun 12. PMID: 32530560; PMCID: PMC7377933. 3. Narayan AK, Lee CI, Lehman CD. Screening for Breast Cancer. Med Clin North Am. 2020 Nov;104(6):1007-1021. doi: 10.1016/j.mcna.2020.08.003. PMID: 33099447. 4. Tomlinson-Hansen SE, Budh DP, Sapra A. Breast Cancer Screening in the Average-Risk Patient. [Updated 2024 Oct 3]. In: StatPearls [Internet]. Treasure Island (FL): StatPearls Publishing; 2025 Jan-. Available from: <https://www.ncbi.nlm.nih.gov/books/NBK556050/> 5. Nelson HD, Cantor A, Humphrey L, et al. Screening for Breast Cancer: A Systematic Review to Update the 2009 U.S. Preventive Services Task Force Recommendation [Internet]. Rockville (MD): Agency for Healthcare Research and Quality (US); 2016 Jan. (Evidence Syntheses, No. 124.) 2, Methods. Available from: <https://www.ncbi.nlm.nih.gov/books/NBK343821/> 6. Bennett A, Shaver N, Vyas N, Almoli F, Pap R, Douglas A, Kibret T, Skidmore B, Yaffe M, Wilkinson A, Seely JM, Little J, Moher D. Screening for breast cancer: a systematic review update to inform the Canadian Task Force on Preventive Health Care guideline. Syst Rev. 2024 Dec 19;13(1):304. doi: 10.1186/s13643-024-02700-3. PMID: 39702409; PMCID: PMC11656969. 7. National Breast Cancer Foundation, Inc. Breast Cancer Screening; available from: <https://www.nationalbreastcancer.org/breast-cancer-screening/>   (accessed on 7 February 2025)   1. Tomlinson-Hansen SE, Budh DP, Sapra A. Breast Cancer Screening in the Average-Risk Patient. 2024 Oct 3. In: StatPearls [Internet]. Treasure Island (FL): StatPearls Publishing; 2025 Jan–. PMID: 32310510. 2. Katsika L, Boureka E, Kalogiannidis I, Tsakiridis I, Tirodimos I, Lallas K, Tsimtsiou Z, Dagklis T. Screening for Breast Cancer: A Comparative Review of Guidelines. Life (Basel). 2024 Jun 19;14(6):777. doi: 10.3390/life14060777. PMID: 38929759; PMCID: PMC11204612. 3. Hernström, V.; Josefsson, V.; Sartor, H.; Schmidt, D.; Larsson, A.-M.; Hofvind, S.; Andersson, I.; Rosso, A.; Hagberg, O.; Lång, K. Screening performance and characteristics of breast cancer detected in the Mammography Screening with Artificial Intelligence trial (MASAI): a randomised, controlled, parallel-group, non-inferiority, single-blinded, screening accuracy study. *The Lancet Digital Health*, doi:10.1016/S2589-7500(24)00267-X. 4. Henderson JT, Webber EM, Weyrich M, et al. Screening for Breast Cancer: A Comparative Effectiveness Review for the U.S. Preventive Services Task Force [Internet]. Rockville (MD): Agency for Healthcare Research and Quality (US); 2024 Apr. (Evidence Synthesis, No. 231.) Available from: <https://www.ncbi.nlm.nih.gov/books/NBK603789/> 5. American Cancer Society. American Cancer Society Recommendations for the Early Detection of Breast Cancer; available from: <https://www.cancer.org/cancer/types/breast-cancer/screening-tests-and-early-detection/american-cancer-society-recommendations-for-the-early-detection-of-breast-cancer.html>   (accessed on 7 February 2025)   1. Katsika, L.; Boureka, E.; Kalogiannidis, I.; Tsakiridis, I.; Tirodimos, I.; Lallas, K.; Tsimtsiou, Z.; Dagklis, T. Screening for Breast Cancer: A Comparative Review of Guidelines. Life **2024**, 14, 777. <https://doi.org/10.3390/life14060777> 2. Katsika L, Boureka E, Kalogiannidis I, Tsakiridis I, Tirodimos I, Lallas K, Tsimtsiou Z, Dagklis T. Screening for Breast Cancer: A Comparative Review of Guidelines. Life (Basel). 2024 Jun 19;14(6):777. doi: 10.3390/life14060777. PMID: 38929759; PMCID: PMC11204612. 3. Ding, R.; Xiao, Y.; Mo, M.; Zheng, Y.; Jiang, Y.-Z.; Shao, Z.-M. Breast cancer screening and early diagnosis in Chinese women. *Cancer Biology &amp; Medicine* **2022**, *19*, 450-467, doi:10.20892/j.issn.2095-3941.2021.0676. 4. Swapana M, Padmavathy C. A critical review on breast cancer literature: Screening, awareness and preventive measures. Mediterranean Journal of Social Sciences. 2015 Aug;6(4):256. 5. National Cancer Institute SEER Training Modules. Screening. <https://training.seer.cancer.gov/breast/screening.html> (accessed on 7 February 2025) 6. Covington, M.F. Maximizing Breast Cancer Detection Through Screening: A Comparative Analysis of Imaging-Based Approaches. *Clinical Breast Cancer* **2025**, *25*, 117-121.e111, doi:10.1016/j.clbc.2024.09.012. 7. National Breast Cancer Foundation. HOW TO DETECT BREAST CANCER; available from: <https://nbcf.org.au/about-breast-cancer/detection-and-awareness/detection/> (accessed on 7 February 2025) 8. Nebraska Cancer Specialists. Screening Tools for Detecting Breast Cancer; available from: https://nebraskacancer.com/screening-tools-for-detecting-breast-cancer/ (accessed on 7 February 2025) 9. Australian Government Cancer Australia. Screening; available from: <https://www.canceraustralia.gov.au/cancer-types/breast-cancer/health-professionals/screening> (accessed on 7 February 2025) 10. Vahabi, M. Breast cancer screening methods: a review of the evidence. *Health Care Women Int* **2003**, *24*, 773-793, doi:10.1080/07399330390229957. 11. Mustapha MT, Ozsahin DU, Ozsahin I, Uzun B. Breast Cancer Screening Based on Supervised Learning and Multi-Criteria Decision-Making. Diagnostics (Basel). 2022 May 27;12(6):1326. doi: 10.3390/diagnostics12061326. PMID: 35741136; PMCID: PMC9221649. 12. Abdullah, K.A., Marziali, S., Nanaa, M. *et al.* Deep learning-based breast cancer diagnosis in breast MRI: systematic review and meta-analysis. *Eur Radiol* (2025). <https://doi.org/10.1007/s00330-025-11406-6> 13. Mann, R.M. Rethinking surveillance after breast cancer. *The Lancet* **2025**, *405*, 356-358, doi:10.1016/S0140-6736(25)00093-5. 14. GOV.UK. World-leading AI trial to tackle breast cancer launched; available from: <https://www.gov.uk/government/news/world-leading-ai-trial-to-tackle-breast-cancer-launched> (accessed on 7 February 2025) 15. Saldanha, O.L., Zhu, J., Müller-Franzes, G. *et al.* Swarm learning with weak supervision enables automatic breast cancer detection in magnetic resonance imaging. *Commun Med* **5**, 38 (2025). <https://doi.org/10.1038/s43856-024-00722-5> 16. Barba D, León-Sosa A, Lugo P, Suquillo D, Torres F, Surre F, Trojman L, Caicedo A. Breast cancer, screening and diagnostic tools: All you need to know. Crit Rev Oncol Hematol. 2021 Jan;157:103174. doi: 10.1016/j.critrevonc.2020.103174. Epub 2020 Nov 11. PMID: 33249359. 17. Getz, K.R., Jeon, M.S., Liu, L. *et al.* Metabolites and lipid species mediate the associations of adiposity in childhood and early adulthood with mammographic breast density in premenopausal women. *Breast Cancer Res* **27**, 18 (2025). <https://doi.org/10.1186/s13058-025-01970-6> 18. Oakes AH, Boyce K, Patton C, Jain S. Rates of Routine Cancer Screening and Diagnosis Before vs After the COVID-19 Pandemic. JAMA Oncol. 2023;9(1):145–146. doi:10.1001/jamaoncol.2022.5481 19. University of Central Lancashire (UCLan). Behind the Screen: an exploratory study of factors influencing breast screening uptake in Lancashire (UK); available from: <https://clok.uclan.ac.uk/54394/> (accessed on 7 February 2025) 20. Pande, Bhanupriya; Shaikh, Mohammed U.. Mixed method analysis of mobile screening clinic for cancer in a tribal community of Maharashtra. Journal of Education and Health Promotion 14(1):41, January 2025. \| DOI: 10.4103/jehp.jehp_707_24 21. HealthWorld.com. More than 14 crore women screened for breast cancer across country: Nadda; available from: <https://health.economictimes.indiatimes.com/news/policy/more-than-14-crore-women-screened-for-breast-cancer-across-country-nadda/118015502> (accessed on 7 February 2025) 22. Breast Cancer in India: 14.6 crore women screened, says health minister; available from: <https://www.cnbctv18.com/webstories/healthcare/breast-cancer-in-india-14-6-crore-women-screened-50000-receiving-treatment-21691.htm> (accessed on 7 February 2025) 23. Rjoop, W., Rjoop, A., Almohtaseb, A. *et al.* Pathological and radiological assessment of benign breast lesions with BIRADS IVc/V subtypes. Should we repeat the biopsy? *BMC Women's Health* **25**, 47 (2025). <https://doi.org/10.1186/s12905-025-03569-7> 24. Xu, T., Chu, C., Xue, S. *et al.* Identification and validation of a prognostic signature of drug resistance and mitochondrial energy metabolism-related differentially expressed genes for breast cancer. *J Transl Med* **23**, 131 (2025). <https://doi.org/10.1186/s12967-025-06080-7> 25. ITV NEWS. NHS to use AI in breast cancer screening in new major trial; available from: <https://www.itv.com/news/2025-02-03/major-trial-launched-to-explore-how-ai-could-transform-breast-cancer-screening> (accessed on 7 February 2025) 26. Chen, Z., Liu, Y., Lyu, M. *et al.* Classifications of triple-negative breast cancer: insights and current therapeutic approaches. *Cell Biosci* **15**, 13 (2025). <https://doi.org/10.1186/s13578-025-01359-0> 27. Firstpost. India screens 146 mn women for breast cancer; health minister says 57,000 diagnosed, 50,000 receiving treatment; available from: <https://www.firstpost.com/india/india-screens-146-mn-women-for-breast-cancer-health-minister-says-57000-diagnosed-50000-receiving-treatment-13860663.html> (accessed on 7 February 2025) 28. Ntekim, A., Folasire, A., Odukoya, O.A. *et al.* Pathway to care among adolescents and young adults with breast cancer in Nigeria: a mixed methods study. *BMC Cancer* **25**, 11 (2025). <https://doi.org/10.1186/s12885-024-13420-1> | | |
| 8 | | What is the survival rate of breast cancer? | 1. Giaquinto, A.N.; Sung, H.; Newman, L.A.; Freedman, R.A.; Smith, R.A.; Star, J.; Jemal, A.; Siegel, R.L. Breast cancer statistics 2024. *CA: A Cancer Journal for Clinicians* **2024**, *74*, 477-495, doi:<https://doi.org/10.3322/caac.21863>. 2. National Breast Cancer Foundation. Inc. Breast Cancer Facts & Stats; available from: <https://www.nationalbreastcancer.org/breast-cancer-facts/> (accessed on 7 February 2025) 3. National Cancer Institute. Cancer Stat Facts: Female Breast Cancer; available from: <https://seer.cancer.gov/statfacts/html/breast.html> (accessed on 7 February 2025) 4. Komen.org. Understanding Breast Cancer Survival Rates; available from: <https://www.komen.org/breast-cancer/facts-statistics/breast-cancer-statistics/survival-rates/> (accessed on 7 February 2025) 5. Giaquinto AN, Sung H, Newman LA, Freedman RA, Smith RA, Star J, Jemal A, Siegel RL. Breast cancer statistics 2024. CA Cancer J Clin. 2024 Nov-Dec;74(6):477-495. doi: 10.3322/caac.21863. Epub 2024 Oct 1. PMID: 39352042. 6. Arnold M, Morgan E, Rumgay H, Mafra A, Singh D, Laversanne M, Vignat J, Gralow JR, Cardoso F, Siesling S, Soerjomataram I. Current and future burden of breast cancer: Global statistics for 2020 and 2040. Breast. 2022 Dec;66:15-23. doi: 10.1016/j.breast.2022.08.010. Epub 2022 Sep 2. PMID: 36084384; PMCID: PMC9465273. 7. Giaquinto, A.N.; Sung, H.; Miller, K.D.; Kramer, J.L.; Newman, L.A.; Minihan, A.; Jemal, A.; Siegel, R.L. Breast Cancer Statistics, 2022. *CA Cancer J Clin* **2022**, *72*, 524-541, doi:10.3322/caac.21754. 8. American Cancer Society. [*Cancer Facts & Figures 2024*](https://www.cancer.org/content/dam/cancer-org/research/cancer-facts-and-statistics/annual-cancer-facts-and-figures/2024/2024-cancer-facts-and-figures-acs.pdf). Atlanta: American Cancer Society; 2024; available from: <https://www.cancer.org/research/cancer-facts-statistics/all-cancer-facts-figures/2024-cancer-facts-figures.html> (accessed on 8 February 2025) 9. CDC.org. Breast Cancer Statistics; available from: <https://www.cdc.gov/breast-cancer/statistics/index.html> (accessed on 8 February 2025) 10. CancerNetwork. Breast Cancer Breakthroughs: 2024 ESMO Highlights; available from: <https://www.cancernetwork.com/view/breast-cancer-breakthroughs-2024-esmo-highlights> (accessed on 8 February 2025) 11. American Cancer Society.  Breast Cancer Facts and Figures; available from: <https://www.cancer.org/content/dam/cancer-org/research/cancer-facts-and-statistics/breast-cancer-facts-and-figures/2024/breast-cancer-facts-and-figures-2024.pdf> (accessed on 8 February 2025) 12. Breast Cancer Research Foundation (BCRF). Breast Cancer Statistics And Resources; available from: <https://www.bcrf.org/breast-cancer-statistics-and-resources/> (accessed on 8 February 2025) 13. American Cancer Society.  Breast Cancer Facts and Figures 2024-2025; available from: https://www.cancer.org/content/dam/cancer-org/cancer-control/en/booklets-flyers/breast-cancer-facts-and-figures-2425.pdf (accessed on 8 February 2025) 14. Komen.org. Breast Cancer Statistics; available from: <https://www.komen.org/breast-cancer/facts-statistics/breast-cancer-statistics/> (accessed on 8 February 2025) 15. Canadian Cancer Society. Cancer-specific stats 2024; available from: <https://cdn.cancer.ca/-/media/files/research/cancer-statistics/2024-statistics/2024-cmaj/2024_cancer-specific-stats.pdf?rev=-1&hash=AB4A55266C9E3E89F32D4B921451F446> (accessed on 8 February 2025) 16. Cancer Research UK. Breast cancer survival statistics; available from: <https://www.cancerresearchuk.org/health-professional/cancer-statistics/statistics-by-cancer-type/breast-cancer/survival> (accessed on 8 February 2025) 17. Women on Guard. Breast Cancer Statistics 2024: What the Latest Data Reveals? available from: <https://womenonguard.com/statistics/breast-cancer/> (accessed on 8 February 2025) 18. Tao X, Li T, Gandomkar Z, Brennan PC, Reed WM. Incidence, mortality, survival, and disease burden of breast cancer in China compared to other developed countries. *Asia-Pac J Clin Oncol*. 2023; 19: 645–654. <https://doi.org/10.1111/ajco.13958> 19. Breast Cancer UK. Facts and Figures; available from: <https://www.breastcanceruk.org.uk/about-breast-cancer/facts-figures-and-qas/facts-and-figures/> (accessed on 8 February 2025) 20. Clinical Advisor. com. 10 Critical Breast Cancer Statistics; available from: <https://www.clinicaladvisor.com/features/breast-cancer-statistics/>   (accessed on 8 February 2025)   1. American Cancer Society.  Survival Rates for Breast Cancer; available from: <https://www.cancer.org/cancer/types/breast-cancer/understanding-a-breast-cancer-diagnosis/breast-cancer-survival-rates.html> (accessed on 8 February 2025) 2. Siegel RL, Giaquinto AN, Jemal A. Cancer statistics, 2024. CA Cancer J Clin. 2024 Jan-Feb;74(1):12-49. doi: 10.3322/caac.21820. Epub 2024 Jan 17. Erratum in: CA Cancer J Clin. 2024 Mar-Apr;74(2):203. doi: 10.3322/caac.21830. PMID: 38230766. 3. Tang DD, Ye ZJ, Liu WW, Wu J, Tan JY, Zhang Y, Xu Q, Xiang YB. Survival feature and trend of female breast cancer: A comprehensive review of survival analysis from cancer registration data. Breast. 2024 Dec 15;79:103862. doi: 10.1016/j.breast.2024.103862. Epub ahead of print. PMID: 39701013; PMCID: PMC11722932. 4. Kaklamani, V.G. and Arteaga, C.L. (2024), Breast cancer: The good, the bad, and an important call to effective risk reduction strategies. CA Cancer J Clin, 74: 471-474. <https://doi.org/10.3322/caac.21867> 5. WebMD. Breast Cancer Survival Rates; available from: <https://www.webmd.com/breast-cancer/breast-cancer-survival-rates> (accessed on 8 February 2025) 6. Healthline. All About Breast Cancer Survival Rates and Outlook; available from: <https://www.healthline.com/health/breast-cancer/survival-facts-statistics> (accessed on 8 February 2025) 7. Canadian Cancer Society. Survival statistics for breast cancer; <https://cancer.ca/en/cancer-information/cancer-types/breast/prognosis-and-survival/survival-statistics> (accessed on 8 February 2025) 8. Cancer Today. Breast Cancer Survival Rates Continue to Rise; available from: <https://www.cancertodaymag.org/spring-2024/breast-cancer-survival-rates-continue-to-rise/> (accessed on 8 February 2025) 9. Noman, S.M., Fadel, Y.M., Henedak, M.T. *et al.* Leveraging survival analysis and machine learning for accurate prediction of breast cancer recurrence and metastasis. *Sci Rep* **15**, 3728 (2025). <https://doi.org/10.1038/s41598-025-87622-3> 10. Cancer Research UK. Survival for breast cancer; available from: <https://www.cancerresearchuk.org/about-cancer/breast-cancer/survival> (accessed on 8 February 2025) 11. AstraZeneca. Enhertu approved in the US as first HER2-directed therapy for patients with HER2-low or HER2-ultralow metastatic breast cancer following disease progression after one or more endocrine therapies; available from: <https://www.astrazeneca.com/media-centre/press-releases/2025/enhertu-approved-in-us-for-breast-cancer-post-et.html>   (accessed on 8 February 2025)   1. American Cancer Society.  Annual Report to the Nation 2022: Overall Cancer Statistics; available from: <https://seer.cancer.gov/report_to_nation/statistics.html?cid=soc_tw_en_sharedlink_arn2023_statistics1> (accessed on 8 February 2025) 2. Chen, Z., Liu, Y., Lyu, M. *et al.* Classifications of triple-negative breast cancer: insights and current therapeutic approaches. *Cell Biosci* **15**, 13 (2025). <https://doi.org/10.1186/s13578-025-01359-0> 3. Healthline. Statistics to Know About Male Breast Cancer; available from: <https://www.healthline.com/health/breast-cancer/male-breast-cancer-statistics> (accessed on 8 February 2025) 4. Ferraro E, Reiner AS, Bou Nassif R, et al. Survival Among Patients With *ERBB2*-Positive Metastatic Breast Cancer and Central Nervous System Disease. JAMA Netw Open. 2025;8(1):e2457483. doi:10.1001/jamanetworkopen.2024.57483 5. American Cancer Society.  Cancer Incidence Rate for Women Under 50 Rises Above Men's; available from: <https://www.cancer.org/research/acs-research-news/cancer-incidence-rate-for-women-under-50-rises-above-mens.html>    (accessed on 8 February 2025)   1. Zheng Y, Yuan Y, Jin M, Wu C. Nomogram prediction of overall survival in breast cancer patients post-surgery: integrating SEER database and multi-center evidence from China. Front Oncol. 2025 Jan 22;14:1470515. doi: 10.3389/fonc.2024.1470515. PMID: 39911819; PMCID: PMC11795552. 2. Medical Xpress. Phase III trial shows new treatment boosts cure rate for most common form of breast cancer; available from: <https://medicalxpress.com/news/2025-01-phase-iii-trial-treatment-boosts.html> (accessed on 8 February 2025) 3. Cure. FDA Approves Enhertu for HER2-Low and -Ultralow Breast Cancer; available from: <https://www.curetoday.com/view/fda-approves-enhertu-for-her2-low-and--ultralow-breast-cancer> (accessed on 8 February 2025) 4. BioSpace. Metastatic HER2-Positive Breast Cancer Market to Reach USD 3,108.1 Million by 2035, Impelled by Significant Progress in Monoclonal Antibodies, Antibody-Drug Conjugates (ADCs), and Tyrosine Kinase Inhibitors (TKIs); available from: <https://www.biospace.com/press-releases/metastatic-her2-positive-breast-cancer-market-to-reach-usd-3-108-1-million-by-2035-impelled-by-significant-progress-in-monoclonal-antibodies-antibody-drug-conjugates-adcs-and-tyrosine-kinase-inhibitors-tkis> (accessed on 8 February 2025) 5. Hernström, V.; Josefsson, V.; Sartor, H.; Schmidt, D.; Larsson, A.-M.; Hofvind, S.; Andersson, I.; Rosso, A.; Hagberg, O.; Lång, K. Screening performance and characteristics of breast cancer detected in the Mammography Screening with Artificial Intelligence trial (MASAI): a randomised, controlled, parallel-group, non-inferiority, single-blinded, screening accuracy study. *The Lancet Digital Health*, doi:10.1016/S2589-7500(24)00267-X. 6. Medical Xpress. Fear of breast cancer recurrence: Survivors describe impacts and coping in study; available from: <https://www.msn.com/en-us/health/other/fear-of-breast-cancer-recurrence-survivors-describe-impacts-and-coping-in-study/ar-AA1yxkxB> (accessed on 8 February 2025) 7. The Times of India. Breast cancer most common among state’s women in 2024: Govt data; available from: <https://timesofindia.indiatimes.com/city/ranchi/breast-cancer-most-common-among-states-women-in-2024-govt-data/articleshow/117924667.cms> (accessed on 8 February 2025) 8. Health World.com. Breast Cancer in India: Tackling early detection and societal barriers for improved survival rates; available from: <https://health.economictimes.indiatimes.com/news/industry/breast-cancer-in-india-tackling-early-detection-and-societal-barriers-for-improved-survival-rates/117922247> (accessed on 8 February 2025) | | |
| 9 | | What are the treatment methods of breast cancer? | 1. Targeted Oncology. Top 10 Breast Cancer Breakthroughs in 2024; available from: <https://www.targetedonc.com/view/top-10-breast-cancer-breakthroughs-in-2024> (accessed on 8 February 2025) 2. Liu Zhen, Yuan Yang, Jiang Zefei. Interpretation of the key points of the 2024 CSCO guideline update for advanced breast cancer [J]. Chinese Journal of Clinical Oncology, 2024, 51(23): 1223-1226.DOI: 10.12354/j.issn.1000-8179.2024.20240987 3. City of Hope. 3 breast cancer treatment innovations to look for in 2024; available from: <https://www.cityofhope.org/3-breast-cancer-treatment-innovations-to-look-for-in-2024> (accessed on 8 February 2025) 4. Outcomes4Me. 2024 Breakthroughs in Breast Cancer Treatment; available from: <https://outcomes4me.com/article/year-in-review-2024-breast-cancer-breakthroughs/> (accessed on 8 February 2025) 5. Gradishar WJ, Moran MS, Abraham J, Abramson V, Aft R, Agnese D, Allison KH, Anderson B, Bailey J, Burstein HJ, Chen N, Chew H, Dang C, Elias AD, Giordano SH, Goetz MP, Jankowitz RC, Javid SH, Krishnamurthy J, Leitch AM, Lyons J, McCloskey S, McShane M, Mortimer J, Patel SA, Rosenberger LH, Rugo HS, Santa-Maria C, Schneider BP, Smith ML, Soliman H, Stringer-Reasor EM, Telli ML, Wei M, Wisinski KB, Yeung KT, Young JS, Schonfeld R, Kumar R. Breast Cancer, Version 3.2024, NCCN Clinical Practice Guidelines in Oncology. J Natl Compr Canc Netw. 2024 Jul;22(5):331-357. doi: 10.6004/jnccn.2024.0035. PMID: 39019058. 6. Liu, B., Zhou, H., Tan, L. *et al.* Exploring treatment options in cancer: tumor treatment strategies. *Sig Transduct Target Ther* 9, 175 (2024). <https://doi.org/10.1038/s41392-024-01856-7> 7. Liu, B., Zhou, H., Tan, L. *et al.* Exploring treatment options in cancer: tumor treatment strategies. *Sig Transduct Target Ther* 9, 175 (2024). <https://doi.org/10.1038/s41392-024-01856-7> 8. Breast Cancer Research Foundation (BCRF). SABCS 2024 Showcases Promising Breast Cancer Treatments and Approaches; available from: <https://www.bcrf.org/blog/sabcs-2024-breast-cancer-treatments-bcrf/> (accessed on 8 February 2025) 9. Cancer Therapy Advisor. SABCS 2024: Improving Treatment for Early Breast Cancer; available from: <https://www.cancertherapyadvisor.com/indepth/sabcs-2024-improving-treatment-for-early-breast-cancer/> (accessed on 8 February 2025) 10. OncLive.com. Insights From SABCS 2024 and Future Directions in Breast Cancer; available from: <https://www.onclive.com/view/insights-from-sabcs-2024-and-future-directions-in-breast-cancer> (accessed on 8 February 2025) 11. Liu, Guozheng, Zhang, Yanwen, Huang, Yong, Triple-Negative Breast Cancer Treatment Advancements: A Review of Evolving Strategies, *European Journal of Cancer Care*, 2024, 8299502, 12 pages, 2024. <https://doi.org/10.1155/2024/8299502> 12. World Economic Forum. 12 new breakthroughs in the fight against cancer; available from: <https://www.weforum.org/stories/2024/10/cancer-treatment-and-diagnosis-breakthroughs/> (accessed on 8 February 2025) 13. National Cancer Comprehensive Network. NCCN Guidelines; available from: <https://www.nccn.org/guidelines/guidelines-detail?category=1&id=1419> (accessed on 8 February 2025) 14. Cancernetwork. Top 10 Findings Shaping the Future of Breast Cancer Care From SABCS 2024; available from: <https://www.cancernetwork.com/view/top-10-findings-shaping-the-future-of-breast-cancer-care-from-sabcs-2024> (accessed on 8 February 2025) 15. Giaquinto AN, Sung H, Newman LA, et al. Breast cancer statistics 2024. *CA Cancer J Clin*. 2024; 74(6): 477-495. doi:[10.3322/caac.21863](https://doi.org/10.3322/caac.21863) 16. Dolgin, E. Cancer drug approvals and setbacks in 2024. *Nat Cancer* 5, 1756–1758 (2024). https://doi.org/10.1038/s43018-024-00873-3 17. Cancer Therapy Advisor. Top Breast Cancer Research of 2024; available from: <https://www.cancertherapyadvisor.com/features/top-breast-cancer-research-of-2024/> (accessed on 8 February 2025) 18. Nave, O., Shor, Y., Bar, R. *et al.* A new treatment for breast cancer using a combination of two drugs: AZD9496 and palbociclib. *Sci Rep* 14, 1307 (2024). <https://doi.org/10.1038/s41598-023-48305-z> 19. ScienceDaily. Strongest contender in decades in fight against breast cancer; available from: <https://www.sciencedaily.com/releases/2024/02/240212133206.htm> (accessed on 8 February 2025) 20. PatientPower. SABCS 2024: the Latest Updates for Breast Cancer Treatments; available from: <https://www.patientpower.info/video/breast-cancer/sabcs-2024-the-latest-updates-for-breast-cancer-treatments> (accessed on 8 February 2025) 21. Heater, N.K.; Warrior, S.; Lu, J. Current and future immunotherapy for breast cancer. *J Hematol Oncol* 2024, *17*, 131, doi:10.1186/s13045-024-01649-z. 22. Marra, A., Chandarlapaty, S. & Modi, S. Management of patients with advanced-stage HER2-positive breast cancer: current evidence and future perspectives. *Nat Rev Clin Oncol* 21, 185–202 (2024). <https://doi.org/10.1038/s41571-023-00849-9> 23. Breast Cancer Expert Committee of National Cancer Quality Control Center; Breast Cancer Expert Committee of China Anti-Cancer Association; Cancer Drug Clinical Research Committee of China Anti-Cancer Association. [Guidelines for diagnosis and treatment of advanced breast cancer in China (2024 edition)]. Zhonghua Zhong Liu Za Zhi. 2024 Dec 23;46(12):1079-1106. Chinese. doi: 10.3760/cma.j.cn112152-20241009-00435. PMID: 39632259. 24. Mukherjee, A.; Bandyopadhyay, D. Targeted Therapy in Breast Cancer: Advantages and Advancements of Antibody-Drug Conjugates, a Type of Chemo-Biologic Hybrid Drugs. *Cancers (Basel)* 2024, *16*, doi:10.3390/cancers16203517. 25. Johns Hopkins Medicine. Novel Drug Combination Shows Promise for Advanced HER2-Negative Breast Cancer; available from: <https://www.hopkinsmedicine.org/news/newsroom/news-releases/2024/02/novel-drug-combination-shows-promise-for-advanced-her2-negative-breast-cancer> (accessed on 8 February 2025) 26. Burguin A, Diorio C, Durocher F. Breast Cancer Treatments: Updates and New Challenges. J Pers Med. 2021 Aug 19;11(8):808. doi: 10.3390/jpm11080808. PMID: 34442452; PMCID: PMC8399130. 27. Medical Xpress.A more effective treatment for aggressive breast cancer: Two inhibitor drugs show potential; available from: <https://medicalxpress.com/news/2025-02-effective-treatment-aggressive-breast-cancer.html> (accessed on 8 February 2025) 28. Wang J, Wu SG. Breast Cancer: An Overview of Current Therapeutic Strategies, Challenge, and Perspectives. Breast Cancer (Dove Med Press). 2023 Oct 20;15:721-730. doi: 10.2147/BCTT.S432526. PMID: 37881514; PMCID: PMC10596062. 29. Marc Thill, Wolfgang Janni, Ute-Susann Albert, Maggie Banys-Paluchowski, Ingo Bauerfeind, Jens Blohmer, Wilfried Budach, Peter Dall, Nina Ditsch, Eva Maria Fallenberg, Peter A. Fasching, Tanja Fehm, Michael Friedrich, Bernd Gerber, Oleg Gluz, Nadia Harbeck, Andreas Hartkopf, Jörg Heil, Jens Huober, Christian Jackisch, Cornelia Kolberg-Liedtke, Hans-Heinrich Kreipe, David Krug, Thorsten Kühn, Sherko Kümmel, Sibylle Loibl, Diana Lüftner, Michael Patrick Lux, Nicolai Maass, Christoph Mundhenke, Toralf Reimer, Kerstin Rhiem, Achim Rody, Marcus Schmidt, Andreas Schneeweiss, Florian Schütz, Hans-Peter Sinn, Christine Solbach, Erich-Franz Solomayer, Elmar Stickeler, Christoph Thomssen, Michael Untch, Isabell Witzel, Achim Wöckel, Rachel Würstlein, Volkmar Müller, Tjoung-Won Park-Simon; Arbeitsgemeinschaft Gynäkologische Onkologie Recommendations for the Diagnosis and Treatment of Patients with Locally Advanced and Metastatic Breast Cancer: Update 2024. Breast Care 17 June 2024; 19 (3): 183–191. <https://doi.org/10.1159/000538753> 30. Rhodes S, Miller DG, Chino F. "When Less is More": Paradigm Shifts in Radiation Treatment for Early-Stage Breast Cancer. Curr Treat Options Oncol. 2024 Dec;25(12):1495-1505. doi: 10.1007/s11864-024-01253-w. Epub 2024 Nov 25. PMID: 39585586. 31. Medical Xpress. Phase III trial shows new treatment boosts cure rate for most common form of breast cancer; available from: <https://medicalxpress.com/news/2025-01-phase-iii-trial-treatment-boosts.html> (accessed on 8 February 2025) 32. Australian Government Cancer Australia. Treatment options; available from: <https://www.canceraustralia.gov.au/cancer-types/breast-cancer/treatment-options> (accessed on 8 February 2025) 33. NOVARTIS. Novartis Kisqali® (ribociclib) receives MHRA authorisation for the adjuvant treatment of HR+/HER2- early breast cancer at high risk of recurrence in a broad population of patients; available from: <https://www.novartis.com/uk-en/news/media-releases/novartis-kisqali-ribociclib-receives-mhra-authorisation-adjuvant-treatment-hrher2-early-breast-cancer-high-risk-recurrence-broad-population-patients> (accessed on 8 February 2025) 34. ROCHE. Roche receives FDA approval for the first companion diagnostic to identify patients with HER2-ultralow metastatic breast cancer eligible for ENHERTU; available from: <https://www.roche.com/media/releases/med-cor-2025-01-31>(accessed on 8 February 2025) 35. Mitri, Z., Gelmon, K.A. Immunotherapy boosts responses in hormone receptor-positive breast cancers. *Nat Med* (2025). <https://doi.org/10.1038/s41591-024-03441-5> 36. Baum, E., Bernhardsgrütter, D., Engst, R. *et al.* The meaning of trust along the treatment pathway of women with breast cancer: a mixed-methods study among cancer survivors. *BMC Women's Health* 25, 25 (2025). <https://doi.org/10.1186/s12905-024-03540-y> 37. Al Sukhun, S.; Temin, S.; Barrios, C.H.; Antone, N.Z.; Guerra, Y.C.; Chavez-MacGregor, M.; Chopra, R.; Danso, M.A.; Gomez, H.L.; Homian, N.M.; Kandil, A.; Kithaka, B.; Koczwara, B.; Moy, B.; Nakigudde, G.; Petracci, F.E.; Rugo, H.S.; El Saghir, N.S.; Arun, B.K. Systemic Treatment of Patients With Metastatic Breast Cancer: ASCO Resource-Stratified Guideline. *JCO Glob Oncol* 2024, *10*, e2300285, doi:10.1200/go.23.00285. 38. AstraZeneca. Enhertu approved in the US as first HER2-directed therapy for patients with HER2-low or HER2-ultralow metastatic breast cancer following disease progression after one or more endocrine therapies; available from: <https://www.astrazeneca.com/media-centre/press-releases/2025/enhertu-approved-in-us-for-breast-cancer-post-et.html>   (accessed on 8 February 2025)   1. Zhang, Y.; Chen, H.; Mo, H.; Zhao, N.; Sun, X.; Liu, B.; Gao, R.; Xu, B.; Zhang, Z.; Liu, Z.; Ma, F. Distinct cellular mechanisms underlie chemotherapies and PD-L1 blockade combinations in triple-negative breast cancer. *Cancer Cell*, doi:10.1016/j.ccell.2025.01.007. 2. BioSpace. Metastatic HER2-Positive Breast Cancer Market to Reach USD 3,108.1 Million by 2035, Impelled by Significant Progress in Monoclonal Antibodies, Antibody-Drug Conjugates (ADCs), and Tyrosine Kinase Inhibitors (TKIs); available from: <https://www.biospace.com/press-releases/metastatic-her2-positive-breast-cancer-market-to-reach-usd-3-108-1-million-by-2035-impelled-by-significant-progress-in-monoclonal-antibodies-antibody-drug-conjugates-adcs-and-tyrosine-kinase-inhibitors-tkis> (accessed on 8 February 2025) 3. Wali, A.F.; Pillai, J.R.; Talath, S.; Shivappa, P.; Sridhar, S.B.; El-Tanani, M.; Rangraze, I.R.; Mohamed, O.I.; Al Ani, N.N. Phytochemicals in Breast Cancer Prevention and Treatment: A Comprehensive Review. Curr. Issues Mol. Biol. 2025, 47, 30. <https://doi.org/10.3390/cimb47010030> 4. Ferraro E, Reiner AS, Bou Nassif R, et al. Survival Among Patients With *ERBB2*-Positive Metastatic Breast Cancer and Central Nervous System Disease. JAMA Netw Open. 2025;8(1):e2457483. doi:10.1001/jamanetworkopen.2024.57483 5. Henricks, J., Haddad, T., Ahmed, O. *et al.* Evaluating risk factors for Trastuzumab-Deruxtecan Pneumonitis in patients with metastatic breast cancer. *Breast Cancer Res* 27, 16 (2025). <https://doi.org/10.1186/s13058-025-01967-1> 6. Li, Z.-Y.; Wu, S.-N.; Lin, P.; Jiang, M.-C.; Chen, C.; Lin, W.-J.; Xue, E.-S.; Liang, R.-X.; Lin, Z.-H. Habitat-based radiomics for revealing tumor heterogeneity and predicting residual cancer burden classification in breast cancer. *Clinical Breast Cancer*, doi:10.1016/j.clbc.2025.01.014. 7. Science Blog. Manuka Honey Shows Promise in Breast Cancer Treatment, UCLA Study Finds; available from: <https://scienceblog.com/manuka-honey-shows-promise-in-breast-cancer-treatment-ucla-study-finds/> (accessed on 8 February 2025)   Li J, Hao C, Wang K, Zhang J, Chen J, Liu Y, Nie J, Yan M, Liu Q, Geng C, Wang X, Wang H, Wang S, Wu J, Yin Y, Song E, Jiang Z. Chinese Society of Clinical Oncology (CSCO) Breast Cancer guidelines 2024. Transl Breast Cancer Res. 2024 Jul 25;5:18. doi: 10.21037/tbcr-24-31. PMID: 39184927; PMCID: PMC11341997. | | |
| 10 | | Can breast cancer recur? | 1. Riggio, A.I., Varley, K.E. & Welm, A.L. The lingering mysteries of metastatic recurrence in breast cancer. *Br J Cancer* 124, 13–26 (2021). <https://doi.org/10.1038/s41416-020-01161-4> 2. Courtney D, Davey MG, Moloney BM, Barry MK, Sweeney K, McLaughlin RP, Malone CM, Lowery AJ, Kerin MJ. Breast cancer recurrence: factors impacting occurrence and survival. Ir J Med Sci. 2022 Dec;191(6):2501-2510. doi: 10.1007/s11845-022-02926-x. Epub 2022 Jan 25. PMID: 35076871; PMCID: PMC9671998. 3. Pan, H.; Gray, R.; Braybrooke, J.; Davies, C.; Taylor, C.; McGale, P.; Peto, R.; Pritchard, K.I.; Bergh, J.; Dowsett, M.; Hayes, D.F. 20-Year Risks of Breast-Cancer Recurrence after Stopping Endocrine Therapy at 5 Years. *New England Journal of Medicine* 2017, *377*, 1836-1846, doi:doi:10.1056/NEJMoa1701830. 4. Haji, H.E.; Souadka, A.; Patel, B.N.; Sbihi, N.; Ramasamy, G.; Patel, B.K.; Ghogho, M.; Banerjee, I. Evolution of Breast Cancer Recurrence Risk Prediction: A Systematic Review of Statistical and Machine Learning–Based Models. *JCO Clinical Cancer Informatics* 2023, e2300049, doi:10.1200/cci.23.00049. 5. Courtney, D.; Davey, M.G.; Moloney, B.M.; Barry, M.K.; Sweeney, K.; McLaughlin, R.P.; Malone, C.M.; Lowery, A.J.; Kerin, M.J. Breast cancer recurrence: factors impacting occurrence and survival. *Ir J Med Sci* 2022, *191*, 2501-2510, doi:10.1007/s11845-022-02926-x. 6. Lafourcade, A., His, M., Baglietto, L. *et al.* Factors associated with breast cancer recurrences or mortality and dynamic prediction of death using history of cancer recurrences: the French E3N cohort. *BMC Cancer* 18, 171 (2018). <https://doi.org/10.1186/s12885-018-4076-4> 7. Rueda, O.M., Sammut, SJ., Seoane, J.A. *et al.* Dynamics of breast-cancer relapse reveal late-recurring ER-positive genomic subgroups. *Nature* 567, 399–404 (2019). <https://doi.org/10.1038/s41586-019-1007-8> 8. Lafourcade, A., His, M., Baglietto, L. *et al.* Factors associated with breast cancer recurrences or mortality and dynamic prediction of death using history of cancer recurrences: the French E3N cohort. *BMC Cancer* 18, 171 (2018). <https://doi.org/10.1186/s12885-018-4076-4> 9. Chen, R., Qarmali, M., Siegal, G.P. *et al.* Receptor conversion in metastatic breast cancer: analysis of 390 cases from a single institution. *Mod Pathol* 33, 2499–2506 (2020). <https://doi.org/10.1038/s41379-020-0615-z> 10. Noman, S.M., Fadel, Y.M., Henedak, M.T. *et al.* Leveraging survival analysis and machine learning for accurate prediction of breast cancer recurrence and metastasis. *Sci Rep* 15, 3728 (2025). https://doi.org/10.1038/s41598-025-87622-3 11. Keogan, A., Nguyen, T.N.Q., Bouzy, P. *et al.* Prediction of post-treatment recurrence in early-stage breast cancer using deep-learning with mid-infrared chemical histopathological imaging. *npj Precis. Onc.* 9, 18 (2025). <https://doi.org/10.1038/s41698-024-00772-x> 12. Yang J, Ju J, Guo L, Ji B, Shi S, Yang Z, Gao S, Yuan X, Tian G, Liang Y, Yuan P. Prediction of HER2-positive breast cancer recurrence and metastasis risk from histopathological images and clinical information via multimodal deep learning. Comput Struct Biotechnol J. 2021 Dec 23;20:333-342. doi: 10.1016/j.csbj.2021.12.028. PMID: 35035786; PMCID: PMC8733169. 13. Asaoka, M.; Narui, K.; Suganuma, N.; Chishima, T.; Yamada, A.; Sugae, S.; Kawai, S.; Uenaka, N.; Teraoka, S.; Miyahara, K.; Kawate, T.; Sato, E.; Nagao, T.; Matsubara, Y.; Gandhi, S.; Takabe, K.; Ishikawa, T. Clinical and pathological predictors of recurrence in breast cancer patients achieving pathological complete response to neoadjuvant chemotherapy. *Eur J Surg Oncol* 2019, *45*, 2289-2294, doi:10.1016/j.ejso.2019.08.001. 14. Chen, Z., Liu, Y., Lyu, M. *et al.* Classifications of triple-negative breast cancer: insights and current therapeutic approaches. *Cell Biosci* 15, 13 (2025). <https://doi.org/10.1186/s13578-025-01359-0> 15. Gao, L., Medford, A., Spring, L. *et al.* Searching for the “Holy Grail” of breast cancer recurrence risk: a narrative review of the hunt for a better biomarker and the promise of circulating tumor DNA (ctDNA). *Breast Cancer Res Treat* 205, 211–226 (2024). <https://doi.org/10.1007/s10549-024-07253-6> 16. Foldi, J.; O’Meara, T.; Marczyk, M.; Sanft, T.; Silber, A.; Pusztai, L. Defining Risk of Late Recurrence in Early-Stage Estrogen Receptor–Positive Breast Cancer: Clinical Versus Molecular Tools. *Journal of Clinical Oncology* 2019, *37*, 1365-1369, doi:10.1200/jco.18.01933. 17. Kumari, S., Kumar, C. (2024). Current Evidence in the Management of Recurrent Breast Cancer. In: Mallick, S., Sharma, C.K. (eds) Evidence in Breast Cancer. Springer, Singapore. <https://doi.org/10.1007/978-981-97-7755-6_38> 18. Riggio AI, Varley KE, Welm AL. The lingering mysteries of metastatic recurrence in breast cancer. Br J Cancer. 2021 Jan;124(1):13-26. doi: 10.1038/s41416-020-01161-4. Epub 2020 Nov 26. PMID: 33239679; PMCID: PMC7782773. 19. Ahmad, Aamir, Pathways to Breast Cancer Recurrence, *International Scholarly Research Notices*, 2013, 290568, 16 pages, 2013. <https://doi.org/10.1155/2013/290568> 20. Fillon M. Breast cancer recurrence risk can remain for 10 to 32 years. *CA Cancer J Clin*. 2022. <https://doi.org/10.3322/caac.21724> 21. Myller, S., Jukkola, A., Jääskeläinen, A., Roininen, N., & Karihtala, P. (2022). How breast cancer recurrences are found – a real-world, prospective cohort study. *Acta Oncologica*, *61*(4), 417–424. <https://doi.org/10.1080/0284186X.2021.2023756> 22. Sparano, J.A. Development and Validation of Data-Driven Estimates of Recurrence Risk and Treatment Benefit in Early Breast Cancer. *Journal of Clinical Oncology 0*, JCO-24-02452, doi:10.1200/jco-24-02452. 23. Pedersen, R.N.; Esen, B.Ö.; Mellemkjær, L.; Christiansen, P.; Ejlertsen, B.; Lash, T.L.; Nørgaard, M.; Cronin-Fenton, D. The Incidence of Breast Cancer Recurrence 10-32 Years After Primary Diagnosis. *JNCI: Journal of the National Cancer Institute* 2021, *114*, 391-399, doi:10.1093/jnci/djab202. 24. A. Kaushik, B. Madhuranath, D. Rao, S. R. Dey and G. S. Sampatrao, "Interpreting Breast Cancer Recurrence Prediction Models: Exploring Feature Importance with Explainable AI," 2024 3rd International Conference on Artificial Intelligence For Internet of Things (AIIoT), Vellore, India, 2024, pp. 1-6, doi: 10.1109/AIIoT58432.2024.10574760. keywords: {Reviews;Explainable AI;Surgery;Predictive models;Feature extraction;Genetics;Breast cancer;Breast Cancer;Feature extraction;Machine Learning;SHAP;LIME}, 25. Stanford Medicine. Breast cancers broadly defined by their genome architecture in Stanford Medicine study; available from: <https://med.stanford.edu/news/all-news/2025/02/breast-cancer-mutation.html> (accessed on 8 February 2025) 26. Lee YJ, Jung SP, Bae JW, Yang SM, You JY, Bae SY. Prognosis according to the timing of recurrence in breast cancer. Ann Surg Treat Res. 2023 Jan;104(1):1-9. doi: 10.4174/astr.2023.104.1.1. Epub 2023 Jan 2. PMID: 36685773; PMCID: PMC9830048. 27. Izci H, Tambuyzer T, Tuand K, Depoorter V, Laenen A, Wildiers H, Vergote I, Van Eycken L, De Schutter H, Verdoodt F, Neven P. A Systematic Review of Estimating Breast Cancer Recurrence at the Population Level With Administrative Data. J Natl Cancer Inst. 2020 Oct 1;112(10):979-988. doi: 10.1093/jnci/djaa050. PMID: 32259259; PMCID: PMC7566328. 28. Hui, C., Dirbas, F.M. & Horst, K.C. Management of Local–Regional Recurrence of Breast Cancer. *Curr Breast Cancer Rep* 15, 185–195 (2023). <https://doi.org/10.1007/s12609-023-00498-y> 29. Mann, R.M. Rethinking surveillance after breast cancer. *The Lancet* 2025, *405*, 356-358, doi:10.1016/S0140-6736(25)00093-5. 30. O'Shaughnessy, J.; Gradishar, W.; O'Regan, R.; Gadi, V. Risk of Recurrence in Patients With HER2+ Early-Stage Breast Cancer: Literature Analysis of Patient and Disease Characteristics. *Clinical Breast Cancer* 2023, *23*, 350-362, doi:10.1016/j.clbc.2023.03.007. 31. OncLive. Managing Unseen Risks: Understanding Recurrence in Early Breast Cancer With No or Limited Nodal Involvement; available from: <https://www.onclive.com/view/managing-unseen-risks-understanding-recurrence-in-early-breast-cancer-with-no-or-limited-nodal-involvement> (accessed on 8 February 2025) 32. Hanker, A.B.; Sudhan, D.R.; Arteaga, C.L. Overcoming Endocrine Resistance in Breast Cancer. *Cancer Cell* **2020**, *37*, 496-513, doi:10.1016/j.ccell.2020.03.009. 33. Jang, H.M.; Bae, K.; Lee, T.Y.; Lim, S.; Bang, M. Contrast-Enhanced Chest Computed Tomography for *In-Breast* Recurrence Detection: Clinical and Imaging Predictors of Visibility. Diagnostics **2025**, 15, 407. <https://doi.org/10.3390/diagnostics15040407> 34. OncLive. FDA Grants Breakthrough Device Designation to DCISionRT for Breast Cancer With DCIS; available from: <https://www.onclive.com/view/fda-grants-breakthrough-device-designation-to-dcisionrt-for-breast-cancer-with-dcis> (accessed on 8 February 2025) 35. American Cancer Society. Treatment of recurrent breast cancer; available from: <https://www.cancer.org/cancer/types/breast-cancer/treatment/treatment-of-breast-cancer-by-stage/treatment-of-recurrent-breast-cancer.html> (accessed on 8 February 2025) 36. Medical Xpress. Phase III trial shows new treatment boosts cure rate for most common form of breast cancer; available from: <https://medicalxpress.com/news/2025-01-phase-iii-trial-treatment-boosts.html> (accessed on 8 February 2025) 37. News Medical Life Sciences. Fear of cancer recurrence disrupts many aspects of breast cancer survivors' lives; available from: <https://www.news-medical.net/news/20250207/Fear-of-cancer-recurrence-disrupts-many-aspects-of-breast-cancer-survivors-lives.aspx>   (accessed on 8 February 2025)   1. AstraZeneca. Enhertu approved in the US as first HER2-directed therapy for patients with HER2-low or HER2-ultralow metastatic breast cancer following disease progression after one or more endocrine therapies; available from: <https://www.astrazeneca.com/media-centre/press-releases/2025/enhertu-approved-in-us-for-breast-cancer-post-et.html>   (accessed on 8 February 2025)   1. Medical Xpress. Fear of breast cancer recurrence: Survivors describe impacts and coping in study; available from: <https://www.msn.com/en-us/health/other/fear-of-breast-cancer-recurrence-survivors-describe-impacts-and-coping-in-study/ar-AA1yxkxB> (accessed on 8 February 2025) 2. NOVARTIS. Novartis Kisqali® (ribociclib) receives MHRA authorisation for the adjuvant treatment of HR+/HER2- early breast cancer at high risk of recurrence in a broad population of patients; available from: <https://www.novartis.com/uk-en/news/media-releases/novartis-kisqali-ribociclib-receives-mhra-authorisation-adjuvant-treatment-hrher2-early-breast-cancer-high-risk-recurrence-broad-population-patients> (accessed on 8 February 2025) 3. Shen, G., Liu, Z., Wang, M. *et al.* Neoadjuvant apatinib addition to sintilimab and carboplatin-taxane based chemotherapy in patients with early triple-negative breast cancer: the phase 2 NeoSAC trial. *Sig Transduct Target Ther* **10**, 41 (2025). <https://doi.org/10.1038/s41392-025-02137-7> 4. AuntMinnie.com. Cryoablation leads to low breast cancer recurrence rates; available from: <https://www.auntminnie.com/clinical-news/interventional/article/15736955/cryoablation-leads-to-low-breast-cancer-recurrence-rates> (accessed on 8 February 2025) 5. Beatty JD, Sun Q, Markowitz D, Chubak J, Huang B, Etzioni R. Identifying breast cancer recurrence histories via patient-reported outcomes. J Cancer Surviv. 2022 Apr;16(2):388-396. doi: 10.1007/s11764-021-01033-7. Epub 2021 Apr 14. PMID: 33852139; PMCID: PMC8525779. 6. Regenstein Institute. Fear of breast cancer recurrence: Impact and coping with being in a dark place; available from: <https://www.regenstrief.org/article/breast-cancer-survivors-getting-out-of-a-dark-place/> (accessed on 8 February 2025) | | |
